# Synthesis of Fluorinated 3,6-Dihydropyridines and 2-(Fluoromethyl)pyridines by Electrophilic Fluorination of 1,2-Dihydropyridines with Selectfluor^®^

**DOI:** 10.3390/molecules25143143

**Published:** 2020-07-09

**Authors:** Nadiia V. Pikun, Arkadij Sobolev, Aiva Plotniece, Martins Rucins, Brigita Vigante, Marina Petrova, Ruslan Muhamadejev, Karlis Pajuste, Yuriy G. Shermolovich

**Affiliations:** 1 Latvian Institute of Organic Synthesis, Aizkraukles Str. 21, LV-1006 Riga, Latvia; arkady@osi.lv (A.S.); aiva@osi.lv (A.P.); rucins@osi.lv (M.R.); vigante@osi.lv (B.V.); marina@osi.lv (M.P.); muhamadejev@osi.lv (R.M.); kpajuste@osi.lv (K.P.); 2 Institute of Organic Chemistry NAS of Ukraine, Murmanska Str. 5, 02660 Kyiv, Ukraine; sherm@ioch.kiev.ua

**Keywords:** dihydropyridines, electrophilic fluorination, Selectfluor^®^, homoallyl long-range coupling, fluorine-containing heterocycles

## Abstract

New fluorinated 3,6-dihydropyridines were obtained by the electrophilic fluorination of 1,2-dihydropyridines with Selectfluor^®^. These 3-fluoro-3,6-dihydropyridines were easily converted to corresponding pyridines by the elimination of hydrogen fluoride under mild conditions. A new approach to the synthesis of methyl 2-(fluoromethyl)-5-nitro-6-arylnicotinates by the fluorination of 3-fluoro-2-methyl-5-nitro-3,6-dihydropyridines or 1,2-dihydropyridines with Selectfluor^®^ has been developed.

## 1. Introduction

The incorporation of fluorine into organic compounds has become a commonly used tool in medicinal chemistry and agrochemistry. The presence of fluorine may result in substantial changes in the biological, as well as physicochemical, properties of organic compounds. The incorporation of fluorine into a drug structure can drastically influence its physicochemical properties, such as bond strength, lipophilicity, bioavailability, conformation, electrostatic potential, dipole moment, pKa, etc. The fluorination of organic compounds also significantly affects their pharmacological properties and toxicology [1,2,3]. The majority of currently marketed modern drugs have heterocyclic fragments in their structures. The fluorinated heterocycles are becoming more significant in a number of disciplines, in particular, the pharmaceutical industry, materials science and agriculture [1,2,3]. 

The electrophilic fluorination of aromatic heterocycles has been less studied than the fluorination of arenes. However, a number of fluoropyrroles [4,5], furans [6], thiophenes [7], pyrrolo[2,3-d]pyrimidines [8,9], quinolines [10,11,12] and indoles [13,14,15,16,17,18,19,20,21,22] have been prepared either by direct fluorination or by fluorodecarboxylation using mainly Selectfluor^®^ and *N*-fluorobenzenesulfonimide.

The chemistry of the pyridines has been extended by the development of many significant transformations, such as addition, addition-elimination, elimination-addition and ring opening, as well as proton abstraction reactions followed by nucleophilic substitution. The nature of the pyridine rings and bases employed plays an important role in the course of reactions [23].

It should also be noted that, despite the fact that fluorine- and trifluoromethyl-substituted pyridines have been very extensively studied, there are very limited data on 2- and 4-(fluoromethyl)pyridines in the literature [24,25,26,27,28,29], which makes these compounds attractive for investigations.

In our previous works, we reported a new electrophilic fluorination reaction of 1,4-dihydropyridines, providing an approach to previously unknown fluorinated 2,6-heptanediones as useful synthons for the preparation of fluorine-containing compounds. The use of 2,6-heptanediones in reactions with ammonia, amines or hydrazine hydrate offered new fluorine-containing carbocycles and heterocycles, such as 3-acetyl-5-(alkyloxycarbonyl)-3,5-difluoro-2,6-dioxo-4-phenylcyclohexan-1-ides, 2-oxa-6-azabicyclo[2.2.2]octanes and pyrazolinone derivatives [30,31]. 

The 1,2-dihydropyridines are known in particular for their ability to readily undergo oxidation to the pyridines [32]. Thus, isomerisation involving hydride transfer was shown to convert 1,2-dihydropyridines into their 1,4-isomers in the presence of the transition metal complex RhCl(PPh_3_)_2_ [33]. Polysubstituted 1,2-dihydropyridines have been found to undergo [4+2] cycloaddition as reactive dienes with maleic anhydride, dimethyl fumarate and methyl acrylate, leading to the formation of 2-azabicyclo[2.2.2]oct-7-enes [34]. The regiospecific 1,3-dipolar cycloaddition reaction of 1,2-dihydropyridines with cyanogen azide and per(poly)fluoroalkanesulfonyl azides has afforded corresponding 2,7-diazabicyclo[4.1.0]hept-4-enes and *N*-(1,2,3,6-tetrahydropyridylidene)fluoroalkanesulfonylamides [35,36]. The alkylation and acylation reactions of 2-alkyl(phenyl)-1-lithio-1,2-dihydropyridines has also been investigated [37,38,39,40].

It has to be noted that, to the best of our knowledge, there are no previous studies on the fluorination reactions of 1,2-dihydropyridines. In this paper, we studied the electrophilic fluorination reaction of 1,2-dihydropyridines **1a**–**k** with Selectfluor^®^ (Table 1).

## 2. Results and Discussion

Starting 2-methyl-5-nitro-1,2-dihydropyridines **1** were obtained by the cyclisation of corresponding nitrodienamines with aldehydes [41]. We show that 1,2-dihydropyridines **1a**–**k** reacted with Selectfluor^®^ at 0 °C in dry acetonitrile under an argon atmosphere to form a series of new 3-fluoro-3,6-dihydropyridines **2a**–**k** (Table 1).

Products **2a**–**k** were isolated as mixtures of two diastereomers. The ratio of diastereomers was determined according to the ^19^F, ^1^H-NMR spectra of compounds **2a**–**k** (Table 1). The attempts to separate the diastereomers by column chromatography failed, as during separation the formation of corresponding pyridines **3a**–**k** occurred as a result of the removal of hydrogen fluoride (according to LC–MS data) (Table 2).

It was found that after the storage of 3-fluoro-3,6-dihydropyridines **2a**–**k** in deuterochloroform solution at room temperature for 2−4 days, hydrogen fluoride was eliminated, leading to the formation of corresponding pyridines **3a**–**k** (Table 2). Compounds **3a**–**k** were isolated by column chromatography in 72−91% yields (Table 2).

The effect of various factors on the course of this reaction, such as the solvent, the order of addition of reagents, temperature and dilution, was studied. Acetonitrile turned out to be the best solvent for the preparation of 3-fluoro-3,6-dihydropyridines **2a**–**k**. Finally, the optimal reaction conditions for the synthesis of fluorinated 3,6-dihydropyridines **2a**–**k** were when the solution of Selectfluor^®^ in acetonitrile was slowly added to the solution of 1,2-dihydropyridines **1a**–**k** in acetonitrile under an argon atmosphere at 0 °C. Compounds **2a**–**k** obtained by this method were further used without additional purification. In other cases, a mixture of 3-fluoro-3,6-dihydropyridines **2a**–**k** and pyridines **3a**–**k** as products of the elimination of hydrogen fluoride from 3-fluoro-3,6-dihydropyridines **2** was formed, according to LC–MS data.

The structures of compounds **2a**–**k** were established and confirmed on the base of one-dimensional ^1^H, ^19^F, ^13^C and two-dimensional {^1^H−^1^H} COSY, {^13^C−^1^H} HSQC and {^13^C−^1^H} HMBC NMR spectral data (Table 3).

The correlations of the protons at 5.4−6.2 ppm in the {^13^C−^1^H} HMBC spectra with the *ipso* and *orto* carbon atoms and aryl substituent for both diastereomers of compounds **2a**–**k** indicate that the mentioned proton and aryl substituent are located at the same C6 carbon atom.

In both diastereomers of compounds **2a**–**k**, the fluorine atom and ester moiety are located at the same C3 carbon atom, as is evidenced by the large values of the constants ^1^*J*_C3-F_ = 189.1−194.2 Hz for the C3 carbon atom at 84.7−87.0 ppm and ^2^*J*_C-F_ = 26.2−30.7 Hz for the carbonyl carbon atom at 165.5−166.7 ppm according to the ^13^C-NMR spectra.

In the ^19^F-NMR spectra of compounds **2a**–**k**, a signal of the fluorine atom appears as a doublet doublet in the range of −139.9 ppm to −145.9 ppm with the constants ^5^*J*_F-H_ = 14.0−17.0 Hz and ^3^*J*_F-H_ = 9.0−9.5 Hz for one diastereomer and in the field of −141.5 ppm to −150.4 ppm with the constants ^5^*J*_F-H_ = 11.4−12.6 Hz and ^3^*J*_F-H_ = 5.4−6.5 Hz for another diastereomer (Table 3).

An interesting fact is that compounds **2a**–**k** in the ^1^H and ^19^F-NMR spectra have unusually large coupling constants between the fluorine atom and C6H proton, which are separated by five bonds (^5^*J* (^19^F,^1^H)). The case of considerably large coupling across five bonds is unusual, however, it can be observed under favourable circumstances [42]. The value of this constant is almost two times higher than the constant between the fluorine nuclei and C4H protons separated by only three bonds (^3^*J*_F-H_ = 5.4−9.5 Hz) (^3^*J* (^19^F,^1^H) in compounds **2a**–**h** (Table 3).

**Figure 1:**
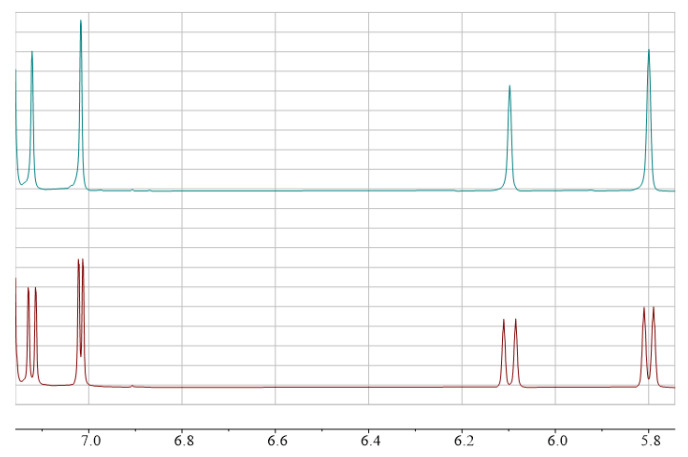
The 600 MHz ^1^H-NMR spectrum of **2a** in CDCl_3_ (red—spectra without decoupling, cyan—with ^19^F decoupled irradiated ^19^F atoms (^1^H-{^19^F}).

To prove the coupling between the fluorine atom and C6H proton, heteronuclear spin decoupling was used. Figure 1 shows the effect of the heteronuclear spin decoupling of the ^19^F nucleus on the C6H and C4H proton signals of compound **2a**. In the below spectrum (Figure 1), the signals of the C4H protons are presented as two doublet doublets coupled with the fluorine atom and C6H proton (^4^*J*_H-H_ ~ 1.3 Hz). The C6H proton is coupled with the fluorine atom through five bonds (Table 3), as well as with the C4H and C2CH_3_ protons (^4^*J*_H-H_ < 1.9 Hz). In the above spectrum (Figure 1), the fluorine atom is irradiated and the doublet of doublets of the C4H and the multiplet of C6H are collapsed to broaden singlets. The C4H and C6H proton signal broadening results from long-range coupling with the mentioned protons. The main information obtained from the ^19^F decoupling is the confirmation of the coupling between the fluorine atom and C4H and C6H protons. 

Our attempts to explain the origin of the large coupling constant ^5^*J* (^19^F,^1^H) value in compounds **2a**–**k** were initially based on the hypothesis of through-space coupling for the spatially proximate C6H proton and F nuclei. The evidence for the through-space mechanism in HF couplings was known previously [43,44,45]. The through-space mechanism is possible for compounds where the fluorine and a coupled proton are separated by a distance which does not exceed the sum of their van der Waals radii (~2.6 Å). 

To ascertain the possibility of through-space proton–fluorine interactions in compounds **2a**–**k**, ab initio calculations were carried out and the data are presented in Table 4.

**Figure 2:**
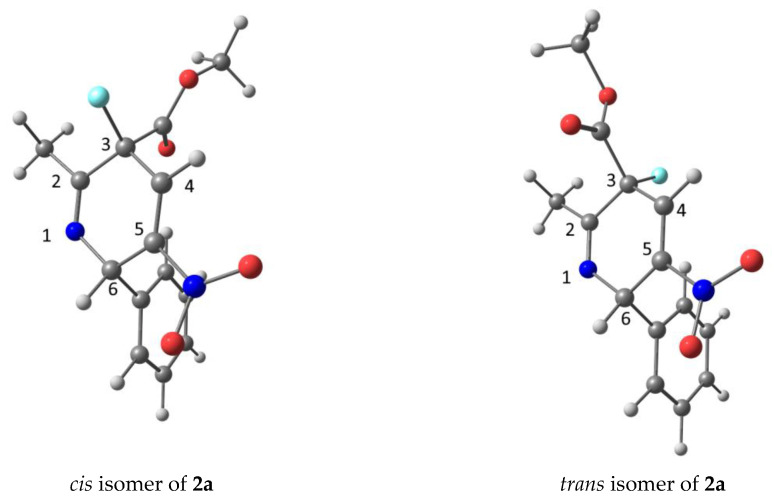
The structure of **2a** optimised by density−functional theory (DFT) calculations. The optimised structures of **2b**–**k** are provided in the Supplementary Materials as Gaussian output files.

According to the quantum chemical calculations, the angles between the C6-N1-C2-C3 and C6-C5-C4-C3 planes are 167.8−178.5° for compounds **2a**–**k** (Figure 2, Table 4), which indicate that the 3,6-dihydropyridine ring is planar or near planar. According to Table 3, the only non-planar conformer was **2k** *cis* (α = 148.2°). It has already been established that the 1,4-cyclohexadiene ring is also relatively flat and for various cyclohexadiene derivatives α = 166−172° [46,47,48,49].

The distance between the proton and the fluorine in *cis* conformer is shorter than in *trans* for 5-nitro-substituted 3,6-dihydropyridines **2a**–**h**. The distance from the C3 and C6 carbons to the N1-C2-C4-C5 plane is larger in its *cis* conformer (except **2a** and **2d** compounds) (Table 4). 

The graphical representation of the optimised molecular geometry for both diastereomers of compound **2a**, obtained with density−functional theory (DFT) calculations, is shown in Figure 2.

The data show that 3,6-dihydropyridines **2** are nearly planar and the distance between the two coupled atoms (C6H proton and F) greatly exceeds the sum of the van der Waals radii varying in the interval 4.6−5.0 Å for compounds **2a**–**k** (Table 4). Consequently, we can claim that the transfer of spin–spin interaction through-space is rather problematic for compounds **2**. The hypothesis about homoallyl long-range coupling transmitted through π-electrons across a double bond of the heterocycle in compounds **2a**–**k** seems to be more likely as compared with through-space interaction. The homoallylic coupling occurs over five bonds and attains a maximum value when the bonds to the coupled atoms are nearly parallel. The coupling is optimal when both C-H and C-F bonds are aligned with the π-orbital of an intervening double bond (perpendicular to the plane of the double bond) [49]. 

Extremally large homoallyl long-range HH couplings have been previously observed for 1,4-cyclohexadienes and related structures, where there were two paths for the coupling [50]. For planar 1,4-cyclohexadienes and their derivatives, the value of the ^5^*J*_H-H*cis*_ is greater (9.6−11.0 Hz) than the ^5^*J*_H-H*trans*_ (7.5−8.4 Hz) [49]. Based on these data, we can also suggest that the *cis* diastereomer of compounds **2a**–**k** has a larger homoallylic coupling constant ^5^*J*_F-H_ than the corresponding *trans* diastereomer.

**Scheme 1:**
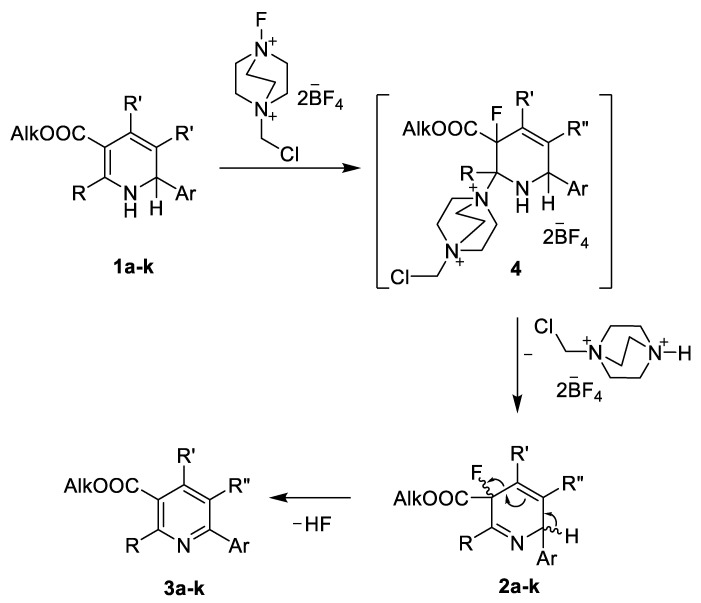
Supposed scheme for the formation of 3-fluoro-3,6-dihydropyridines **2a**–**k** and pyridines **3a**–**k**.

We can propose the following scheme for the formation of 3-fluoro-3,6-dihydropyridines **2a**–**k**. At the first stage of the reaction, Selectfluor^®^ is probably attached to the double bond of 1,2-dihydropyridines **1a**–**k** with the formation of ammonium salts **4**. The decomposition of these salts **4** under mild conditions results in the formation of 3-fluoro-3,6-dihydropyridines **2a**–**k** (Scheme 1).

The reaction of 3-fluoro-2-methyl-5-nitro-3,6-dihydropyridines **2a,c,f,g** with Selectfluor^®^ was also investigated. It was found that this reaction with 2 equiv. of Selectfluor^®^ led to the formation of a mixture of new methyl 2-(fluoromethyl)-5-nitro-6-arylnicotinates **5a**–**d** and 2-methylpyridines **3a,c,f,g** (Table 5, Method A). The compounds **5a**–**d** and compounds **3a,c,f,g** were isolated by column chromatography in 21−43% and 10–52% yields, respectively. Our attempts to optimise the reaction conditions, such as the solvent, the order of addition of reagents, temperature and dilution, did not lead to an increase in the yields of compounds **5a**–**d**. The one-pot reaction of 1,2-dihydropyridines **1a,c,f,g** with 3 equiv. of Selectfluor^®^, in this case omitting the isolation of 3,6-dihydropyridines **2a,c,f,g**, directly formed 2-(fluoromethyl)pyridines **5a**–**d** and 2-methylpyridines **3a,c,f,g** (Table 5, Method B). The ratios of compounds **3a,c,f,g** and **5a**–**d** were determined according to the ^1^H-NMR spectra and the LC–MS data of the reaction mixture were similar to those obtained by Method A (Table 5).

**Scheme 2:**
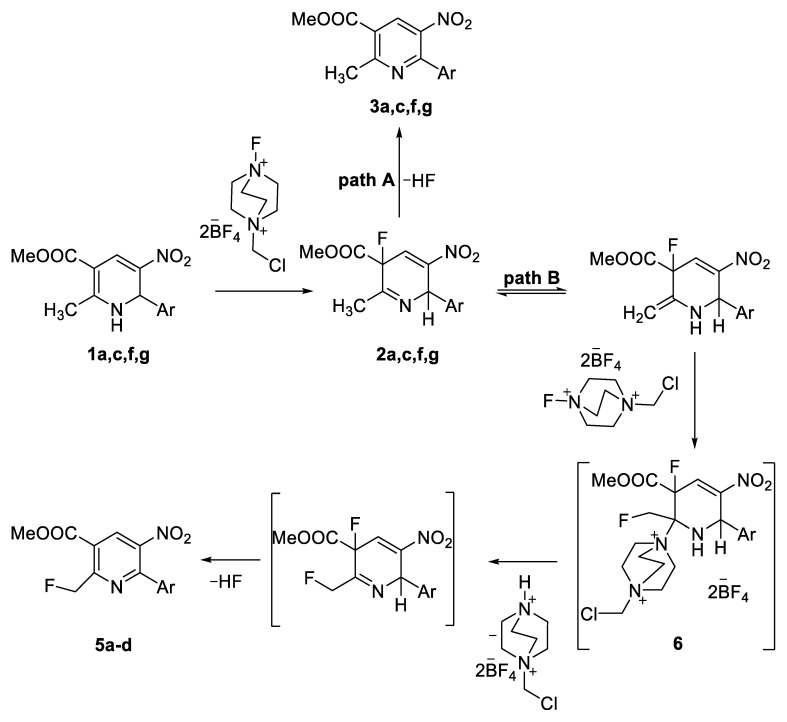
Supposed scheme for the formation of methyl 2-(fluoromethyl)-5-nitro-6-arylnicotinates **5a–d**.

It is known that N-F fluorinating agents and, in particular Selectfluor^®^, are also strong oxidants, and competition between fluorofunctionalisation and oxidation can occur. The selectivity of fluorination reactions may thereby be decreased if the structure of the substrate contains oxidisable functional groups or heteroatoms. In our case, the reaction of 3-fluoro-2-methyl-5-nitro-3,6-dihydropyridines **2a,c,f,g** with Selectfluor^®^ can proceed in two competitive directions. Tautomers of 3-fluoro-3,6-dihydropyridines **2** with an enamine-like structure are probably fluorinated by Selectfluor^®^ on the methylene site of the enamine to form ammonium salts **6** (Scheme 2, Path B). The decomposition of these salts **6**, followed by the elimination of hydrogen fluoride, leads to the formation of 2-(fluoromethyl)pyridines **5a**–**d** (Scheme 2).

On the other hand, 2-methylpyridines **3a,c,f,g** can be formed as a result of the elimination of hydrogen fluoride from 3-fluoro-2-methyl-5-nitro-3,6-dihydropyridines **2a,c,f,g**. (Scheme 2, Path A). Under similar conditions, pyridines **3** did not react with Selectfluor^®^.

There are very limited data in the literature about straightforward methods for the preparation of 2- or 4-(fluoromethyl)pyridines. When direct fluorination was performed on 4-picoline by F_2_/N2, 2-fluoro-4-methylpyridine was the main product [24]. Additionally, 4-(fluoromethyl)pyridine was prepared from 4-(chloromethyl)pyridine using activated tetrabutylammonium fluoride (TBAF) [25].

The preparation of 2- or 4-(fluoromethyl)pyridines was realised with *N*-fluorobis(trifluoromethanesulfonyl)imide from corresponding methylpyridines in dichloromethane at room temperature. In these reactions, the presence of sodium carbonate is necessary to suppress the formation of unreactive pyridinium salts, which are generated by the strongly acidic bis(trifluoromethanesulfonyl)imide co-product [26,27].

*N*-Fluorobis(trifluoromethanesulfonyl)imide, (CF_3_SO_2_*)*_2_NF, is a very attractive fluorinating agent because of its favourable physical properties and high reactivity, but it is not commercially available. In the reaction of 2-(chloromethyl)pyridine with potassium fluoride, 2-(fluoromethyl)pyridines can be also obtained (Finkelstein reaction, S_N_2 reaction that involves the exchange of one halogen atom for another) [28] or in the reaction of 2-pyridinylmethanol derivatives with (diethylamino)sulfur trifluoride [29].

Thus, the electrophilic fluorination reactions of 1,2-dihydropyridines **1** and 3-fluoro-2-methyl-5-nitro-3,6-dihydropyridines **2** with a commercially available Selectfluor^®^ can be used as a convenient approach for the preparation of 2-(fluoromethyl)pyridines **5**.

## 3. Materials and Methods 

### 3.1. General Methods

All reagents were purchased from Acros Organics (Geel, Belgium), Sigma-Aldrich/Merck KGaA (Darmstadt, Germany), or Alfa Aesar (Lancashire, UK) and used without further purification. TLC was performed on silica gel 60 F_254_ aluminium sheets 20 × 20 cm (Merck KGaA, Darmstadt, Germany). Silica gel of particle size 35−70 µm (Merck KGaA, Darmstadt, Germany) was used for column chromatography. Melting points were recorded on an OptiMelt digital melting point apparatus (Stanford Research Systems, Sunnyvale, CA, USA) and were uncorrected. One-dimensional ^1^H, ^13^C, ^19^F and two-dimensional {^1^H-^1^H} COSY, {^13^C-^1^H} HMBC and {^13^C-^1^H} HSQC-NMR spectra were recorded on a Bruker Avance Neo 400 MHz (Bruker Biospin Gmbh, Rheinstetten, Germany) with a double resonance broadband CryoProbe Prodigy (^1^H 399.96 MHz, ^13^C 100.58 MHz, ^19^F 376.30 MHz). Inverse gate decoupling experiments for ^19^F on ^1^H were recorded on a Bruker Avance Neo 600 MHz (Bruker Biospin Gmbh, Rheinstetten, Germany) with a quadrupole resonance CryoProbe (CP QCI 600S3 H/F-C/N-D-05 Z) (^1^H 599.93 MHz, ^19^F 564.44 MHz). Chemical shifts of the hydrogen, carbon and fluorine atoms are presented in parts per million (ppm) and refer to the residual signals of the deuterated CDCl_3_ (δ: 7.26) solvent for the ^1^H-NMR spectra and CDCl_3_ (δ: 77.16) solvent for the ^13^C-NMR, respectively. For the ^19^F-NMR experiments, indirect referencing (Bruker standard referencing) was used. Coupling constants *J* are reported in hertz (Hz). Multiplicities are abbreviated as s = singlet; d = doublet; t = triplet; q = quartet, m = multiplet; br = broad; dd = double doublet; dm = double multiplet; td = triple doublet; ddd = double double doublet. Low resolution mass spectra (MS) were determined on an Acquity UPLC system (Waters, Milford, MA, USA) connected to a Waters SQ Detector-2 operating in the electrospray ionisation (ESI) positive or negative ion mode on a Waters Acquity UPLC^®^ BEH C18 column (1.7 µm, 2.1 × 50 mm, using gradient elution with acetonitrile (0.01% formic acid) in water (0.01% formic acid). High resolution mass spectra (HRMS) were determined on an Acquity UPLC H-Class system (Waters, Milford, MA, USA) connected to a Waters Synapt G2-Si operating in the ESI positive or negative ion mode on a Waters Acquity UPLC^®^ BEH C18 column (1.7 µm, 2.1 × 50 mm, using gradient elution with acetonitrile (0.01% formic acid) in water (0.01% formic acid). Infrared spectra were recorded with a Prestige-21 FTIR spectrometer (Shimadzu, Kyoto, Japan). Elemental analyses were determined on an Elemental Combustion System ECS 4010 (Costech International S.p.A., Milano, Italy) at the Laboratory of Chromatography of the Latvian Institute of Organic Synthesis. All quantum chemical calculations were performed with Gaussian 09 [51]. Geometry optimisation and vibrational calculations were performed with 6-311++G(d2,p2) basis set using DFT B97D3 functional with Grimme’s empirical dispersion D3 correction [52]. All geometries were optimised using the polarisable continuum model (PCM) solvation model for chloroform. For all optimised geometries, vibrational harmonic frequencies were calculated at the same level of theory, all the obtained harmonic frequencies were positive. Planes, distances to planes and angles between planes were calculated with the Mercury 3.1 program by Cambridge Crystallographic Data Centre (CCDC) (https://www.ccdc.cam.ac.uk/solutions/csd-system/components/mercury/).

### 3.2. General Procedure for the Synthesis of 3-Fluoro-3,6-dihydropyridines **2a**–**k**

A solution of Selectfluor^®^ (0.170 g, 0.5 mmol) in dry acetonitrile (5 mL) was slowly added dropwise to a solution of 1,2-dihydropyridines **1a**–**k** (0.5 mmol) in dry acetonitrile (5 mL) in the presence of 3 Å molecular sieves at 0°C under an argon atmosphere. The reaction mixture was stirred for 10 min at 0 °C, after which the temperature was slowly raised to room temperature. The reaction mixture was concentrated in vacuo to dryness, then diluted with diethyl ether (15 mL) and filtered. The filtrate was evaporated in vacuo to give compounds **2a**–**k** as oils in 82−97% yields. Compounds **2a**–**k** obtained by this method were further used without additional purification.

#### 3.2.1. Methyl 3-fluoro-2-methyl-5-nitro-6-phenyl-3,6-dihydropyridine-3-carboxylate (**2a**)

Pale yellow oil. Yield 0.137 g (94%). Mixture of diastereomers (the ratio is 45:55 according to the ^19^F and ^1^H-NMR spectra). ^1^H-NMR (CDCl_3_): δ 2.20 (d, ^4^*J*_H-F_ = 2.1 Hz, 3H, CH_3_ of major diastereomer), 2.22 (dd, ^4^*J*_H-F_ = 1.8 Hz, ^5^*J*_H-H_ = 1.5 Hz, 3H, CH_3_ of minor diastereomer), 3.87 (s, 3H, COOCH_3_ of minor diastereomer), 3.89 (s, 3H, COOCH_3_ of major diastereomer), 5.77−5.81 (dm, ^5^*J*_H-F_ = 12.1 Hz, 1H, C6H of major diastereomer), 6.06−6.11 (dm, ^5^*J*_H-F_ = 15.4 Hz, 1H, C6H of minor diastereomer), 7.09 (dd, ^3^*J*_H-F_ = 5.6 Hz, ^4^*J*_H-H_ = 1.2 Hz, 1H, C4H of major diastereomer), 7.18 (dd, ^3^*J*_H-F_ = 9.4 Hz, ^4^*J*_H-H_ = 1.3 Hz, 1H, C4H of minor diastereomer), 7.22−7.24 (m, 1H, CH, Ph), 7.24−7.26 (m, 1H, CH, Ph), 7.30−7.38 (m, 8H, CH, Ph of both diastereomers). ^19^F-NMR (CDCl_3_): δ −140.6 (dd, ^5^*J*_F-H_ = 15.4 Hz, ^3^*J*_F-H_ = 9.4 Hz, 1F, CF of minor diastereomer), −142.4 (dd, ^5^*J*_F-H_ = 12.1 Hz, ^3^*J*_F-H_ = 5.6 Hz, 1F, CF of major diastereomer). ^13^C-NMR (CDCl_3_): δ 20.7 (s, CH_3_ of minor diastereomer), 20.9 (s, CH_3_ of major diastereomer), 54.1 (s, COOCH_3_ of minor diastereomer), 54.2 (s, COOCH_3_ of major diastereomer), 61.9 (d, ^4^*J*_C-F_ = 3.2 Hz, C6H of major diastereomer), 62.5 (d, ^4^*J*_C-F_ = 2.7 Hz, C6H of minor diastereomer), 84.8 (d, ^1^*J*_C-F_ = 189.9 Hz, CF of major diastereomer), 87.0 (d, ^1^*J*_C-F_ = 191.2 Hz, CF of minor diastereomer), 120.9 (d, ^2^*J*_C-F_ = 27.3 Hz, C4H of major diastereomer), 122.3 (d, ^2^*J*_C-F_ = 25.9 Hz, C4H of minor diastereomer), 128.0 (s, 2×CH, Ph of major diastereomer), 128.2 (s, 2×CH, Ph of minor diastereomer), 128.8 (s, CH, Ph of minor diastereomer), 128.9 (s, CH, Ph of major diastereomer), 129.0 (s, 2×CH, Ph of minor diastereomer), 129.3 (s, 2×CH, Ph of major diastereomer), 135.5 (d, ^5^*J*_C-F_ = 6.9 Hz, C_q_, Ph of major diastereomer), 135.8 (d, ^5^*J*_C-F_ = 4.1 Hz, C_q_, Ph of minor diastereomer), 154.7 (d, ^3^*J*_C-F_ = 8.7 Hz, C5 of minor diastereomer), 155.7 (d, ^3^*J*_C-F_ = 8.4 Hz, C5 of major diastereomer), 156.8 (d, ^2^*J*_C-F_ = 20.9 Hz, C2 of major diastereomer), 157.3 (d, ^2^*J*_C-F_ = 19.8 Hz, C2 of minor diastereomer), 165.5 (d, ^2^*J*_C-F_ = 28.8 Hz, C=O of minor diastereomer), 165.9 (d, ^2^*J*_C-F_ = 29.3 Hz, C=O of major diastereomer). HRMS (ESI^+^): Calcd for C_14_H_13_FN_2_O_4_ [M + H]: 293.0946; found 293.0938.

#### 3.2.2. Methyl 6-([1,1′-biphenyl]-4-yl)-3-fluoro-2-methyl-5-nitro-3,6-dihydropyridine-3-carboxylate (**2b**) 

Pale yellow oil. Yield 0.177 g (96%). Mixture of diastereomers (the ratio is 40:60 according to the ^19^F and ^1^H-NMR spectra). ^1^H-NMR (CDCl_3_): δ 2.23−2.24 (dm, ^4^*J*_H-F_ = 1.8 Hz, 3H, CH_3_ of major diastereomer), 2.24−2.25 (dm, ^4^*J*_H-F_ = 1.5 Hz, 3H, CH_3_ of minor diastereomer), 3.91 (s, 3H, COOCH_3_ of minor diastereomer), 3.92 (s, 3H, COOCH_3_ of major diastereomer), 5.91−5.96 (dm, ^5^*J*_H-F_ = 12.1 Hz, 1H, C6H of major diastereomer), 6.20−6.25 (dm, ^5^*J*_H-F_ = 15.4 Hz, 1H, C6H of minor diastereomer), 7.13 (dd, ^3^*J*_H-F_ = 5.6 Hz, ^4^*J*_H-H_ = 1.2 Hz, 1H, C4H of major diastereomer), 7.23 (dd, ^3^*J*_H-F_ = 9.3 Hz, ^4^*J*_H-H_ = 1.2 Hz, 1H, C4H of minor diastereomer), 7.23−7.37 (m, 4H, CH, Ar of both diastereomers), 7.41−7.46 (m, 6H, CH, Ar of both diastereomers), 7.54−7.60 (m, 8H, CH, Ar of both diastereomers). ^19^F-NMR (CDCl_3_): δ −140.3 (dd, ^5^*J*_F-H_ = 15.4 Hz, ^3^*J*_F-H_ = 9.3 Hz, 1F, CF of minor diastereomer), −142.4 (dd, ^5^*J*_F-H_ = 12.1 Hz, ^3^*J*_F-H_ = 5.6 Hz, 1F, CF of major diastereomer). ^13^C-NMR (CDCl_3_): δ 20.9 (s, CH_3_ of minor diastereomer), 21.0 (s, CH_3_ of major diastereomer), 54.2 (s, COOCH_3_ of minor diastereomer), 54.3 (s, COOCH_3_ of major diastereomer), 61.7 (d, ^4^*J*_C-F_ = 3.2 Hz, C6H of major diastereomer), 62.3 (d, ^4^*J*_C-F_ = 2.7 Hz, C6H of minor diastereomer), 84.8 (d, ^1^*J*_C-F_ = 189.1 Hz, CF of major diastereomer), 86.1 (d, ^1^*J*_C-F_ = 191.4 Hz, CF of minor diastereomer), 121.2 (d, ^2^*J*_C-F_ = 24.3 Hz, C4H of major diastereomer), 122.5 (d, ^2^*J*_C-F_ = 26.3 Hz, C4H of minor diastereomer), 127.25 (s, Ar of minor diastereomer), 127.29 (s, Ar of major diastereomer), 127.7 (s, Ar of minor diastereomer), 127.9 (s, Ar of minor diastereomer), 128.1 (s, Ar of major diastereomer), 128.5 (s, Ar of major diastereomer), 128.6 (s, Ar of minor diastereomer), 128.9 (s, Ar of major diastereomer), 134.4 (d, ^5^*J*_C-F_ = 7.0 Hz, C_q_, Ar of major diastereomer), 134.8 (d, ^5^*J*_C-F_ = 3.8 Hz, C_q_, Ar of minor diastereomer), 140.5 (s, C_q_, Ar of both diastereomers), 141.8 (s, C_q_, Ar of minor diastereomer), 142.0 (s, C_q_, Ar of major diastereomer), 154.7 (d, ^3^*J*_C-F_ = 9.0 Hz, C5 of minor diastereomer), 155.7 (d, ^3^*J*_C-F_ = 8.7 Hz, C5 of major diastereomer), 157.1 (d, ^2^*J*_C-F_ = 20.8 Hz, C2 of major diastereomer), 157.6 (d, ^2^*J*_C-F_ = 20.0 Hz, C2 of minor diastereomer), 165.8 (d, ^2^*J*_C-F_ = 28.8 Hz, C=O of minor diastereomer), 166.1 (d, ^2^*J*_C-F_ = 29.4 Hz, C=O of major diastereomer). HRMS (ESI^+^): Calcd for C_20_H_17_FN_2_O_4_ [M + H]: 369.1251; found 369.1264.

#### 3.2.3. Methyl 3-fluoro-6-(2-methoxyphenyl)-2-methyl-5-nitro-3,6-dihydropyridine-3-carboxylate (**2c**)

Yellow oil. Yield 0.145 g (90%). Mixture of diastereomers (the ratio is 45:55 according to the ^19^F and ^1^H-NMR spectra). ^1^H-NMR (CDCl_3_): δ 2.18−2.20 (m, 6H, CH_3_ of both diastereomers), 3.85 (s, 3H, COOCH_3_ of minor diastereomer), 3.89 (s, 3H, COOCH_3_ of major diastereomer), 3.91 (s, 3H, OCH_3_ of minor diastereomer), 3.96 (s, 3H, OCH_3_ of major diastereomer), 6.21−6.26 (dm, ^5^*J*_H-F_ = 12.6 Hz, 1H, C6H of major diastereomer), 6.69−6.75 (dm, ^5^*J*_H-F_ = 16.6 Hz, 1H, C6H of minor diastereomer), 6.84−6.89 (m, 1H, CH, Ar), 6.90−6.94 (m, 2H, 2 × CH, Ar of both diastereomers), 6.96−7.00 (m, 2H, CH, Ar of both diastereomers), 7.03−7.05 (m, 1H, CH, Ar), 7.07 (dd, ^3^*J*_H-F_ = 6.2 Hz, ^4^*J*_H-H_ = 1.6 Hz, 1H, C4H of major diastereomer), 7.22 (dd, ^3^*J*_H-F_ = 9.0 Hz, ^4^*J*_H-H_ = 1.4 Hz, 1H, C4H of minor diastereomer); 7.27−7.31 (m, 2H, CH, Ar of both diastereomers). ^19^F-NMR (CDCl_3_): δ −139.9 (dd, ^5^*J*_F-H_ = 16.6 Hz, ^3^*J*_F-H_ = 9.0 Hz, 1F, CF of minor diastereomer), −144.3 (dd, ^5^*J*_F-H_ = 12.6 Hz, ^3^*J*_F-H_ = 6.2 Hz, 1F, CF of major diastereomer). ^13^C-NMR (CDCl_3_): δ 20.8 (s, 2 × CH_3_ of both diastereomers), 54.2 (s, COOCH_3_ of minor diastereomer), 54.3 (s, COOCH_3_ of major diastereomer), 55.7 (s, OCH_3_ of major diastereomer), 55.9 (d, ^4^*J*_C-F_ = 2.6 Hz, C6H of minor diastereomer), 56.2 (s, OCH_3_ of minor diastereomer), 56.8 (d, ^4^*J*_C-F_ = 3.2 Hz, C6H of major diastereomer), 86.16 (d, ^1^*J*_C-F_ = 190.1 Hz, CF of major diastereomer), 86.23 (d, ^1^*J*_C-F_ = 191.3 Hz, CF of minor diastereomer), 120.86 (s, Ar of minor diastereomer), 120.90 (d, ^2^*J*_C-F_ = 24.8 Hz, C4H of major diastereomer), 121.3 (s, CH, Ar of major diastereomer), 122.6 (d, ^2^*J*_C-F_ = 26.6 Hz, C4H of minor diastereomer), 123.6 (s, Ar of major diastereomer), 125.6 (s, Ar of minor diastereomer), 127.6 (s, Ar of minor diastereomer), 129.03−129.06 (m, C_q_, Ar of minor diastereomer), 130.52−130.56 (m, C_q_, Ar of major diastereomer), 144.7 (s, C-OCH_3_, Ar of both diastereomers), 155.13−155.31 (m, C5 of both diastereomers), 157.3 (s, C2 of major diastereomer), 157.4 (s, C2 of minor diastereomer), 166.0 (d, ^2^*J*_C-F_ = 30.7 Hz, C=O of both diastereomers). MS: *m/z* = 323 [M + 1] (100%). 

#### 3.2.4. Methyl 3-fluoro-2-methyl-5-nitro-6-(3,4,5-trimethoxyphenyl)-3,6-dihydropyridine- 3-carboxylate (**2d**)

Yellow brown oil. Yield 0.172 g (90%). Mixture of diastereomers (the ratio is 20:80 according to the ^19^F and ^1^H-NMR spectra). ^1^H-NMR (CDCl_3_): δ 2.21 (d, ^4^*J*_H-F_ = 1.7 Hz, 3H, CH_3_ of minor diastereomer), 2.24 (t, ^4^*J*_H-F_ = 1.4 Hz, 3H, CH_3_ of major diastereomer), 3.81 (s, 6H, OCH_3_, Ar of both diastereomers), 3.83 (s, 6H, 2 × OCH_3_, Ar of minor diastereomer), 3.85 (s, 6H, 2 × OCH_3_, Ar of major diastereomer), 3.91 (s, 3H, COOCH_3_ of minor diastereomer), 3.94 (s, 3H, COOCH_3_ of major diastereomer), 5.77−5.81 (dm, ^5^*J*_H-F_ = 12.2 Hz, 1H, C6H of minor diastereomer), 6.02−6.07 (dm, ^5^*J*_H-F_ = 15.8 Hz, 1H, C6H of major diastereomer), 6.43 (s, 2H, 2 × CH, Ar of minor diastereomer), 6.62 (s, 2H, 2 × CH, Ar of major diastereomer), 7.08 (dd, ^3^*J*_H-F_ = 5.4 Hz, ^4^*J*_H-H_ = 1.1 Hz, 1H, C4H of minor diastereomer), 7.14 (dd, ^3^*J*_H-F_ = 9.2 Hz, ^4^*J*_H-H_ = 1.2 Hz, 1H, C4H of major diastereomer). ^19^F-NMR (CDCl_3_): δ −140.3 (dd, ^5^*J*_F-H_ = 15.8 Hz, ^3^*J*_F-H_ = 9.2 Hz, 1F, CF of major diastereomer), −141.6 (dd, ^5^*J*_F-H_ = 12.2 Hz, ^3^*J*_F-H_ = 5.4 Hz, 1F, CF of minor diastereomer). ^13^C-NMR (CDCl_3_): δ 21.0 (s, CH_3_ of major diastereomer), 21.1 (s, CH_3_ of minor diastereomer), 54.3 (s, COOCH_3_, Ar of minor diastereomer), 54.4 (s, COOCH_3_, Ar of major diastereomer), 56.27 (s, 2 × OCH_3_ of minor diastereomer), 56.33 (s, 2 × OCH_3_ of minor diastereomer), 60.9 (s, OCH_3_ of both diastereomers), 62.1 (d, ^4^*J*_C-F_ = 3.4 Hz, C6H of minor diastereomer), 62.7 (d, ^4^*J*_C-F_ = 2.8 Hz, C6H of major diasteeomer), 85.7 (d, ^1^*J*_C-F_ = 189.2 Hz, CF of minor diastereomer), 86.1 (d, ^1^*J*_C-F_ = 194.2 Hz, CF of major diastereomer), 105.2 (s, CH, Ar of minor diasrereomer), 105.7 (s, CH, Ar of major diastereomer), 121.0 (d, ^2^*J*_C-F_ = 24.1 Hz, C4H of minor diastereomer), 121.8 (d, ^2^*J*_C-F_ = 25.4 Hz, C4H of major diastereomer), 130.8 (d, ^5^*J*_C-F_ = 7.0 Hz, C_q_, Ar of minor diastereomer), 131.0 (d, ^5^*J*_C-F_ = 4.3 Hz, C_q_, Ar of major diastereomers), 138.4 (s, C-OCH_3_, Ar of major diastereomer), 138.5 (s, C-OCH_3_, Ar of minor diastereomer), 153.7 (s, 2 × C-OCH_3_, Ar of major diastereomer), 153.9 (s, 2 × C-OCH_3_, Ar of minor diastereomer), 155.1 (d, ^3^*J*_C-F_ = 9.0 Hz, C5 of major diastereomer), 155.8 (d, ^3^*J*_C-F_ = 8.4 Hz, C5 of minor diastereomer), 157.2 (d, ^2^*J*_C-F_ = 20.5 Hz, C2 of minor diastereomers), 157.5 (d, ^2^*J*_C-F_ = 19.6 Hz, C2 of major diastereomer), 166.0 (d, ^2^*J*_C-F_ = 29.0 Hz, C=O of minor diastereomer); 166.4 (d, ^2^*J*_C-F_ = 27.5 Hz, C=O of minor diastereomer). MS: *m/z* = 383 [M + 1] (100%). 

#### 3.2.5. Methyl 2-ethyl-3-fluoro-6-(2-methoxyphenyl)-5-nitro-3,6-dihydropyridine-3-carboxylate (**2e**) 

Yellow oil. Yield 0.163 g (97%). Mixture of diastereomers (the ratio is 50:50 according to the ^19^F and ^1^H-NMR spectra). ^1^H-NMR (CDCl_3_): δ 1.08−1.14 (m, 6H, 2 × CH_2_CH_3_ of both diastereomers); 2.34−2.44 (m, 2H, CH_2_CH_3_), 2.51−2.64 (m, 2H, CH_2_CH_3_), 3.84 (s, 3H, OCH_3_), 3.87 (s, 3H, COOCH_3_), 3.89 (s, 3H, OCH_3_), 3.95 (s, 3H, COOCH_3_), 6.20−6.25 (dm, ^5^*J*_H-F_ = 12.6 Hz, 1H, C6H), 6.71−6.77 (dm, ^5^*J*_H-F_ = 17.0 Hz, 1H, C6H), 6.84−7.06 (m, 6H, 6 × CH, Ar), 7.00 (dd, ^3^*J*_H-F_ = 6.5 Hz, ^4^*J*_H-H_ = 1.6 Hz, 1H, C4H), 7.16 (dd, ^3^*J*_H-F_ = 9.1 Hz, ^4^*J*_H-H_ = 1.2 Hz, 1H, C4H), 7.26−7.30 (m, 2H, 2 × CH, Ar of both diastereomers). ^19^F-NMR (CDCl_3_): δ −140.5 (dd, ^5^*J*_F-H_ = 17.0 Hz, ^3^*J*_F-H_ = 9.1 Hz, 1F, CF), −145.1 (dd, ^5^*J*_F-H_ = 12.6 Hz, ^3^*J*_F-H_ = 6.5 Hz, 1F, CF). ^13^C-NMR (CDCl_3_): δ 10.8 (s, CH_2_CH_3_), 10.9 (s, CH_2_CH_3_), 27.0 (s, CH_2_CH_3_), 27.1 (s, CH_2_CH_3_), 54.0 (s, COOCH_3_), 54.1 (s, COOCH_3_), 55.7 (s, OCH_3_, Ar), 55.9 (d, ^4^*J*_C-F_ = 2.6 Hz, C6H), 56.3 (s, OCH_3_, Ar), 56.9 (d, ^4^*J*_C-F_ = 3.4 Hz, C6H), 86.3 (d, ^1^*J*_C-F_ = 190.0 Hz, CF), 86.4 (d, ^1^*J*_C-F_ = 190.3 Hz, CF), 111.85 (s, CH, Ar), 111.91 (s, CH, Ar), 120.7 (s, CH, Ar), 121.0 (d, ^2^*J*_C-F_ = 25.4 Hz, C4H), 121.2 (s, CH, Ar), 122.4 (d, ^2^*J*_C-F_ = 25.7 Hz, C4H), 124.3 (d, ^5^*J*_C-F_ = 6.3 Hz, C_q_, Ar), 124.9 (d, ^5^*J*_C-F_ = 3.9 Hz, C_q_, Ph), 127.8 (s, CH, Ar), 129.2 (s, CH, Ar), 130.1 (s, CH, Ar), 130.2 (s, CH, Ar), 155.7 (d, ^3^*J*_C-F_ = 10.1 Hz, C5), 157.5 (d, ^3^*J*_C-F_ = 5.8 Hz, C5), 160.4 (d, ^2^*J*_C-F_ = 19.4 Hz, C2 of both diastereomers), 166.2 (d, ^2^*J*_C-F_ = 29.1 Hz, C=O), 166.7 (d, ^2^*J*_C-F_ = 30.2 Hz, C=O). MS: *m/z* = 337 [M + 1] (100%). 

#### 3.2.6. Methyl 3-fluoro-2-methyl-5-nitro-6-(4-nitrophenyl)-3,6-dihydropyridine-3-carboxylate (**2f**)

Yellow oil. Yield 0.169 g (94%). Mixture of diastereomers (the ratio is 50:50 according to the ^19^F and ^1^H-NMR spectra). ^1^H-NMR (CDCl_3_): 2.22 (dd, ^4^*J*_H-F_ = 1.9 Hz, ^4^*J*_H-H_ =0.9 Hz, 3H, CH_3_), 2.23−2.24 (dm, ^4^*J*_H-F_ = 1.6 Hz, 3H, CH_3_), 3.91 (s, 3H, COOCH_3_), 3.92 (s, 3H, COOCH_3_), 5.91−5.96 (dm, ^5^*J*_H-F_ = 11.4 Hz, 1H, C6H), 6.24−6.28 (dm, ^5^*J*_H-F_ = 14.4 Hz, 1H, C6H), 7.21 (dd, ^3^*J*_H-F_ = 5.7 Hz, ^4^*J*_H-H_ = 1.3 Hz, 1H, C4H), 7.30 (dd, ^3^*J*_H-F_ = 9.5 Hz, ^4^*J*_H-H_ = 1.1 Hz, 1H, C4H), 7.41−7.44 (dm, ^3^*J*_H-H_ = 8.7 Hz, 2H, 2×CH, Ar), 7.54−7.58 (dm, ^3^*J*_H-H_ = 8.7 Hz, 2H, 2×CH, Ar), 8.20−8.25 (m, 4H, 2 × CH, Ar of both diastereomers). ^19^F-NMR (CDCl_3_): δ −141.6 (dd, ^5^*J*_F-H_ = 14.4 Hz, ^3^*J*_F-H_ = 9.5 Hz, 1F, CF), −143.0 (dd, ^5^*J*_F-H_ = 11.4 Hz, ^3^*J*_F-H_ = 5.7 Hz, 1F, CF). ^13^C-NMR (CDCl_3_): δ 20.9 (s, CH_3_), 21.1 (s, CH_3_), 54.5 (s, 2 × COOCH_3_ of both diastereomers), 61.1 (d, ^4^*J*_C-F_ = 3.3 Hz, C6H), 61.9 (d, ^4^*J*_C-F_ = 2.7 Hz, C6H), 85.8 (d, ^1^*J*_C-F_ = 190.2 Hz, CF), 86.2 (d, ^1^*J*_C-F_ = 194.1 Hz, CF), 122.7 (d, ^2^*J*_C-F_ = 24.6 Hz, C4H), 123.9 (d, ^2^*J*_C-F_ = 26.4 Hz, C4H), 124.3 (s, Ar), 124.5 (s, Ar), 129.2 (s, Ar), 129.4 (s, Ar), 142.86 (d, ^5^*J*_C-F_ = 6.9 Hz, C_q_, Ar), 142.92 (d, ^5^*J*_C-F_ = 4.0 Hz, C_q_, Ar), 148.1 (s, C−NO_2_, Ar), 148.2 (s, C−NO_2_, Ar), 153.4 (d, ^3^*J*_C-F_ = 8.6 Hz, C5), 154.4 (d, ^3^*J*_C-F_ = 8.4 Hz, C5), 158.6 (d, ^2^*J*_C-F_ = 20.9 Hz, C2), 159.0 (d, ^2^*J*_C-F_ = 20.0 Hz, C_2_), 165.6 (d, ^2^*J*_C-F_ = 28.6 Hz, C=O of both diastereomers). MS: *m/z* = 338 [M + 1] (100%). 

#### 3.2.7. Methyl 3-fluoro-6-(3-fluorophenyl)-2-methyl-5-nitro-3,6-dihydropyridine-3-carboxylate (**2g**)

Yellow oil. Yield 0.136 g (88%). Mixture of diastereomers (the ratio is 50:50 according to the ^19^F and ^1^H-NMR spectra). ^1^H-NMR (CDCl_3_): δ 2.20−2.21 (dm, ^4^*J*_H-F_ = 1.8 Hz, 3H, CH_3_), 2.22−2.23 (dm, ^4^*J*_H-F_ = 1.5 Hz, 3H, CH_3_), 3.90 (s, 3H, COOCH_3_), 3.91 (s, 3H, COOCH_3_), 5.82−5.87 (dm, ^5^*J*_H-F_ = 11.8 Hz, 1H, C6H), 6.14−6.18 (dm, ^5^*J*_H-F_ = 14.9 Hz, 1H, C6H), 6.93−6.96 (dm, ^3^*J*_H-F_ = 9.5 Hz, 1H, CH, Ar), 7.00−7.06 (m, 4H, CH, Ar of both diastereomers), 7.12 (dd, ^3^*J*_H-F_ = 5.6 Hz, ^4^*J*_H-H_ = 1.2 Hz, 1H, C4H), 7.17−7.20 (dm, ^3^*J*_H-F_ = 7.7 Hz, 1H, CH, Ar ), 7.22 (dd, ^3^*J*_H-F_ = 9.4 Hz, ^4^*J*_H-H_ = 1.1 Hz, 1H, C4H), 7.31−7.37 (m, 2H, CH, Ar of both diastereomers). ^19^F-NMR (CDCl_3_): δ −111.46 −(−111.52) (m, 1F, CF, Ar), −111.73−(−111.79) (m, 1F, CF, Ar), −140.7 (dd, ^5^*J*_F-H_ = 14.9 Hz, ^3^*J*_F-H_ = 9.5 Hz, 1F, CF), −142.8 (dd, ^5^*J*_F-H_ = 11.8 Hz, ^3^*J*_F-H_ = 5.6 Hz, 1F, CF). ^13^C-NMR (CDCl_3_): δ 20.8 (s, CH_3_), 21.0 (s, CH_3_), 54.3 (s, COOCH_3_), 54.4 (s, COOCH_3_), 61.4 (dd, ^4^*J*_C-F_ = 3.2 Hz, ^4^*J*_C-F_ = 2.0 Hz, C6H), 62.0 (t, ^4^*J*_C-F_ = 2.2 Hz, C6H), 84.7 (d, ^1^*J*_C-F_ = 190.7 Hz, CF), 86.0 (d, ^1^*J*_C-F_ = 191.9 Hz, CF), 115.2 (d, ^2^*J*_C-F_ = 23.2 Hz, CH, Ar), 115.3 (d, ^2^*J*_C-F_ = 22.4 Hz, CH, Ar), 115.9 (d, ^2^*J*_C-F_ = 21.1 Hz, CH, Ar), 116.1 (d, ^2^*J*_C-F_ = 21.1 Hz, CH, Ar), 121.7 (d, ^2^*J*_C-F_ = 24.2 Hz, C4H), 122.9 (d, ^2^*J*_C-F_ = 26.4 Hz, C4H), 123.8 (d, ^4^*J*_C-F_ = 3.0 Hz, CH, Ar), 124.3 (d, ^4^*J*_C-F_ = 3.1 Hz, CH, Ar), 130.7 (d, ^3^*J*_C-F_ = 8.5 Hz, CH, Ar), 130.9 (d, ^3^*J*_C-F_ = 8.2 Hz, CH, Ar), 137.91−138.06 (dm, ^5^*J*_C-F_ = 7.0 Hz, C_q_, Ar), 138.2 (dd, ^5^*J*_C-F_ = 4.1 Hz, ^3^*J*_C-F_ = 7.4 Hz, C_q_, Ar), 154.2 (d, ^3^*J*_C-F_ = 9.0 Hz, C5), 155.2 (d, ^3^*J*_C-F_ = 8.5 Hz, C5), 157.7 (d, ^2^*J*_C-F_ = 20.7 Hz, C2), 158.1 (d, ^2^*J*_C-F_ = 19.8 Hz, C2), 163.0 (d, ^1^*J*_C-F_ = 246.2 Hz, CF, Ar), 163.1 (d, ^1^*J*_C-F_ = 247.6 Hz, CF, Ar), 165.5 (d, ^2^*J*_C-F_ = 29.0 Hz, C=O), 165.9 (d, ^2^*J*_C-F_ = 29.6 Hz, C=O). MS: *m/z* = 311 [M + 1] (100%). 

#### 3.2.8. Methyl 6-(1,3-diphenyl-1H-pyrazol-4-yl)-3-fluoro-2-methyl-5-nitro-3,6-dihydropyridine- 3-carboxylate (**2h**)

Yellow brown oil. Yield 0.150 g (89%). Mixture of diastereomers (the ratio is 10:90 according to the ^19^F and ^1^H-NMR spectra). ^1^H-NMR (CDCl_3_): δ 2.18−2.20 (dm, ^4^*J*_H-F_ = 1.8 Hz, 3H, CH_3_), 2.25−2.26 (dm, ^4^*J*_H-F_ = 1.5 Hz, 3H, CH_3_), 3.97 (s, 3H, COOCH_3_), 3.98 (s, 3H, COOCH_3_), 6.03−6.07 (dm, ^5^*J*_H-F_ = 11.9 Hz, 1H, C6H of minor diastereomer), 6.27−6.32 (dm, ^5^*J*_H-F_ = 14.0 Hz, 1H, C6H of major diastereomer), 7.02 (dd, ^3^*J*_H-F_ = 5.8 Hz, ^4^*J*_H-H_ = 1.5 Hz, 1H, C4H), 7.19 (dd, ^3^*J*_H-F_ = 9.5 Hz, ^4^*J*_H-H_ = 1.1 Hz, 1H, C4H), 7.42−7.48 (m, 8H, CH, Ph of both diastereomers), 7.50−7.55 (m, 4H, CH, Ph of both diastereomers), 7.66−7.69 (m, 4H, CH, Ph of both diastereomers), 7.87 (s, 2H, CH, pyrazolyl of both diastereomers), 8.01−8.04 (m, 4H, CH, Ph of both diastereomers). ^19^F-NMR (CDCl_3_): δ −142.6 (dd, ^5^*J*_F-H_ = 14.0 Hz, ^3^*J*_F-H_ = 9.5 Hz, 1F, CF of major diastereomer), −144.2 (dd, ^5^*J*_F-H_ = 11.9 Hz, ^3^*J*_F-H_ = 5.8 Hz, 1F, CF of minor diastereomer). ^13^C-NMR (CDCl_3_): δ 20.8 (s, CH_3_ of major diastereomer), 21.1 (s, CH_3_ of minor diastereomer), 53.7 (d, ^4^*J*_C-F_ = 2.4 Hz, C6H of major diastereomer), 54.3 (s, COOCH_3_ of minor diastereomer), 54.5 (s, COOCH_3_ of major diastereomer), 86.6 (d, ^1^*J*_C-F_ = 193.8 Hz, CF of major diastereomer), 117.2 (d, ^5^*J*_C-F_ = 4.1 Hz, pyrazolyl of major diastereomer), 119.2 (s, Ph, of major diastereomer) 122.1 (d, ^2^*J*_C-F_ = 26.3 Hz, C4H of major diastereomer), 126.9 (s, Ph of both diastereomers), 128.7 (s, CH, pyrazolyl of both diastereomers), 128.8 (s, Ph of both diastereomers), 128.9 (s, Ph of both diastereomers), 129.6 (s, Ph of both diastereomers), 132.6 (s, C_q_, Ph of both diastereomers), 132.6 (s, C_q_, Ph of both diastereomers), 139.9 (s, C_q_, Ph of both diastereomers), 153.0 (s, C−Ph, pyrazolyl of both diastereomers), 154.8 (d, ^3^*J*_C-F_ = 8.7 Hz, C_5_ of major diastereomer), 157.1 (d, ^2^*J*_C-F_ = 20.2 Hz, C2 of major diastereomer), 166.2 (d, ^2^*J*_C-F_ = 29.6 Hz, C=O of major diastereomer). MS: *m/z* = 338 [M + 1] (100%). 

#### 3.2.9. Diethyl 5-fluoro-4,6-dimethyl-2-phenyl-2,5-dihydropyridine-3,5-dicarboxylate (**2k**) 

Pale yellow oil. Yield 0.142 g (82%). Mixture of diastereomers (the ratio is 15:85 according to the ^19^F and ^1^H-NMR spectra). ^1^H-NMR (CDCl_3_): δ 0.89 (t, ^3^*J*_H-H_ = 7.4 Hz, 3H, COOCH_2_CH_3_ of minor diastereomer), 1.01 (t, ^3^*J*_H-H_ = 7.4 Hz, 3H, COOCH_2_CH_3_ of major diastereomer), 1.13 (t, ^3^*J*_H-H_ = 6.9 Hz, 3H, FCOOCH_2_CH_3_ of minor diastereomer), 1.23 (t, ^3^*J*_H-H_ = 7.4 Hz, 3H, FCOOCH_2_CH_3_ of major diastereomer), 1.94 (d, ^4^*J*_H-F_ = 2.1 Hz, 3H, CH_3_ of minor diastereomer), 2.02 (d, ^4^*J*_H-F_ = 1.9 Hz, 3H, CH_3_ of major diastereomer), 2.07 (dd, ^4^*J*_H-F_ = 1.8 Hz, ^5^*J*_H-H_ = 1.4 Hz, 3H, CH_3_C=N of major diastereomer), 2.09 (dd, ^4^*J*_H-F_ = 2.4 Hz, ^5^*J*_H-H_ = 1.9 Hz, 3H, CH_3_C=N of minor diastereomer), 3.84−3.93 (m, 4H, 2 × COOCH_2_CH_3_ of both diastereomers), 4.07−4.16 (m, 4H, 2 × COOCH_2_CH_3_ of both diastereomers), 5.24−5.30 (dm, ^5^*J*_H-F_ = 12.0 Hz, 1H, C6H of minor diastereomer), 5.57−5.62 (dm, ^5^*J*_H-F_ = 15.5 Hz, 1H, C6H of major diastereomer), 7.11−7.34 (m, 10H, CH, Ph of both diastereomers). ^19^F-NMR (CDCl_3_): δ −145.9 (d, ^5^*J*_F-H_ = 15.6 Hz, 1F, CF of major diastereomer), −150.4 (d, ^5^*J*_F-H_ = 12.2 Hz, 1F, CF of minor diastereomer). ^13^C-NMR (CDCl_3_): δ 13.5 (d, ^3^*J*_C-H_ = 4.5 Hz, CH_3_ of both diastereomers), 14.0 (s, COOCH_2_CH_3_ of both diastereomers), 14.1 (s, COOCH_2_CH_3_ of both diastereomers), 21.2 (s, CH_3_C=N of both diastereomers), 61.1 (s, COOCH_2_CH_3_ of both diastereomers), 63.2 (s, COOCH_2_CH_3_ of both diastereomers), 64.5 (d, ^4^*J*_C-F_ = 2.5 Hz, C6H of minor diastereomer), 65.3 (d, ^4^*J*_C-F_ = 1.8 Hz, C6H of major diastereomer), 84.65 (d, ^1^*J*_C-F_ = 183.8 Hz, CF of major diastereomer), 127.8 (s, CH, Ph), 128.0 (s, CH, Ph), 128.3 (s, 2×CH, Ph), 128.4 (s, 2×CH, Ph), 128.5 (s, 2×CH, Ph), 128.6 (s, 2×CH, Ph), 130.7 (d, ^2^*J*_C-F_ = 22.1 Hz, C4 of minor diastereomer), 132.0 (d, ^2^*J*_C-F_ = 21.6 Hz, C4 of major diastereomer), 133.8 (d, ^5^*J*_C-F_ = 4.8 Hz, C_q_, Ph of major diastereomer), 134.2 (d, ^5^*J*_C-F_ = 5.1 Hz, C_q_, Ph of minor diastereomer), 138.8 (d, ^3^*J*_C-F_ = 4.8 Hz, C5 of major diastereomer), 139.2 (d, ^3^*J*_C-F_ = 5.4 Hz, C5 of minor diastereomer), 157.4 (d, ^2^*J*_C-F_ = 22.7 Hz, C2 of major diastereomer), 158.5 (d, ^2^*J*_C-F_ = 20.4 Hz, C2 of minor diastereomer), 165.9 (s, C=O), 166.2 (s, C=O), 166.5 (d, ^2^J_C-F_ = 29.7 Hz, C=O, CH_3_CH_2_COOCF). MS: *m/z* = 348 [M + 1] (100%). 

### 3.3. General Procedure for the Synthesis of Pyridines **3a**–**k**

A deuterochloroform solution (3 mL) of 3-fluoro-3,6-dihydropyridines **2a**–**k** (0.3 mmol) was maintained for 2 days in the case of compounds **2c,d,e,h,k** and for 4 days in the case of compounds **2a,b,f,g** at room temperature, after which the solvent was evaporated in vacuo. Pyridines **3a**–**k** were isolated by column chromatography in 72–91% yields.

#### 3.3.1. Methyl 2-methyl-5-nitro-6-phenylnicotinate (**3a**)

White powder. Yield 0.074 g (91%). Mp 93−94 °C (from ethanol). R_f_: 0.54 (petroleum ether – ethyl acetate 4:1). Anal. calcd for C_14_H_12_N_2_O_4_: C, 61.76; H, 4.44; N, 10.29; found: C, 61.85; H, 4.55; N, 10.16. ^1^H-NMR (CDCl_3_): δ 2.98 (s, 3H, CH_3_), 3.98 (s, 3H, COOCH_3_), 7.47−7.49 (m, 3H, 3×CH, Ph), 7.59−7.61 (m, 2H, 2×CH, Ph), 8.69 (s, 1H, C4H). ^13^C-NMR (CDCl_3_): δ 25.4 (s, CH_3_), 53.0 (s, COOCH_3_), 123.8 (s, C3), 128.5 (s, CH, Ph), 129.0 (s, CH, Ph), 130.5 (s, CH, Ph), 135.1 (s, C_q_, Ph), 135.9 (s, C4H), 143.9 (s, C5), 154.5 (s, C6), 163.5 (s, C2), 165.0 (s, C=O). MS: *m/z* = 273 [M + 1] (100%). 

#### 3.3.2. Methyl 6-([1,1′-biphenyl]-4-yl)-2-methyl-5-nitronicotinate (**3b**) 

Colorless viscous oil. Yield 0.089 g (85%). R_f_: 0.50 (petroleum ether – ethyl acetate 4:1). Anal. calcd for C_20_H_16_N_2_O_4_: C, 68.96; H, 4.63; N, 8.04; found: C, 69.02; H, 4.71; N, 7.96. ^1^H-NMR (CDCl_3_): δ 3.00 (s, 3H, CH_3_), 4.00 (s, 3H, COOCH_3_), 7.36−7.50 (m, 3H, Ar), 7.62−7.65 (m, 2H, Ar), 7.70 (s, 4H, Ar), 8.71 (s, 1H, C4H). MS: *m/z* = 349 [M + 1] (100%). 

#### 3.3.3. Methyl 6-(2-methoxyphenyl)-2-methyl-5-nitronicotinate (**3c**)

Colorless viscous oil. Yield 0.065 g (72%). R_f_: 0.63 (petroleum ether–ethyl acetate 4:1). ^1^H-NMR (CDCl_3_): δ 2.98 (s, 3H, CH_3_), 3.71 (s, 3H, OCH_3_), 3.98 (s, 3H, COOCH_3_), 6.91 (d, ^3^*J*_H-H_ = 8.4 Hz, 1H, CH, Ar), 7.16 (td, ^3^*J*_H-H_ = 7.6 Hz, ^4^*J*_H-H_ = 1.0 Hz, 1H, CH, Ar), 7.46 (ddd, ^3^*J*_H-H_ = 7.6 Hz, ^3^*J*_H-H_ = 8.4 Hz, ^4^*J*_H-H_ = 1.7 Hz, 1H, CH, Ar), 7.73 (dd, ^3^*J*_H-H_ = 7.6 Hz, ^4^*J*_H-H_ = 1.7 Hz, 1H, CH, Ar), 8.74 (s, 1H, C4H). HRMS (ESI^+^): Calcd for C_15_H_14_N_2_O_5_ [M + H]: 303.0981; found 303.0985.

#### 3.3.4. Methyl 2-methyl-5-nitro-6-(3,4,5-trimethoxyphenyl)nicotinate (**3d**)

Red brown powder. Yield 0.091 g (84%). Mp 172−173°C (from ethanol). R_f_: 0.29 (petroleum ether – ethyl acetate 4:1). IR ν_max_ (Film) 3006, 2945, 2840, 1731, 1592, 1554, 1455, 1424, 1334. ^1^H-NMR (CDCl_3_): δ 2.97 (s, 3H, CH_3_), 3.89 (s, 6H, 2×OCH_3_, Ar), 3.90 (s, 3H, OCH_3_, Ar), 3.98 (s, 3H, COOCH_3_), 6.82 (s, 2H, 2×CH, Ar), 8.63 (s, 1H, C4H). ^13^C-NMR (CDCl_3_): δ 25.4 (s, CH_3_), 53.0 (s, COOCH_3_), 56.4 (s, 2 × OCH_3_, Ar), 61.1 (s, OCH_3_, Ar), 105.8 (s, 2 × CH, Ar), 123.7 (s, C3), 130.8 (s, C_q_, Ar), 134.9 (s, CH), 140.3 (s, C−OCH_3_, Ar), 144.0 (s, C5), 153.7 (s, 2 × OCH_3_, Ar), 153.8 (s, C6), 163.3 (s, C2), 164.9 (s, C=O). HRMS (ESI^+^): Calcd for C_17_H_18_N_2_O_7_ [M + H]: 363.1192; found 363.1201.

#### 3.3.5. Methyl 2-ethyl-6-(2-methoxyphenyl)-5-nitronicotinate (**3e**) 

Pale yellow oil. Yield 0.078 g (82%). R_f_: 0.61 (petroleum ether–ethyl acetate 4:1). IR ν_max_ (Film) 3082, 2977, 2840, 1733, 1598, 1456, 1354. Anal. calcd for C_16_H_16_N_2_O_5_: C, 60.76; H, 5.10; N, 8.86; found: C, 60.84; H, 5.19; N, 8.75. ^1^H-NMR (CDCl_3_): δ 1.36 (t, ^3^*J*_H-H_ = 7.4 Hz, 3H, CH_2_CH_3_), 3.33 (t, ^3^*J*_H-H_ = 7.4 Hz, 2H, CH_2_CH_3_), 3.72 (s, 3H, OCH_3_), 3.98 (s, 3H, COOCH_3_), 6.92 (d, ^3^*J*_H-H_ = 8.6 Hz, 1H, CH, Ar), 7.13−7.18 (m, 1H, CH, Ar), 7.43−7.50 (m, 1H, CH, Ar), 7.76 (dd, ^3^*J*_H-H_ = 7.6 Hz, ^3^*J*_H-H_ = 1.7 Hz, 1H, CH, Ar), 8.70 (s, 1H, C4H). MS: *m/z* = 317 [M + 1] (100%). 

#### 3.3.6. Methyl 2-methyl-5-nitro-6-(4-nitrophenyl)nicotinate (**3f**)

White powder. Yield 0.088 g (93%). Mp 167−168 °C (from ethanol). R_f_: 0.39 (petroleum ether–ethyl acetate 4:1). IR ν_max_ (Film) 3086, 2995, 1719, 1553, 1517, 1443, 1358, 1345. ^1^H-NMR (CDCl_3_): δ 2.99 (s, 3H, CH_3_), 4.01 (s, 3H, COOCH_3_), 7.73−7.75 (dm, ^3^*J*_H-H_ = 9.1 Hz, 2H, 2×CH, Ar), 8.32−8.34 (dm, ^3^*J*_H-H_ = 9.1 Hz, 2H, 2×CH, Ar), 8.80 (s, 1H, C4H). ^13^C-NMR (CDCl_3_): δ 25.4 (s, CH_3_), 53.2 (s, COOCH_3_), 124.0 (s, 2×CH, Ar), 125.2 (s, C−COOCH_3_), 129.7 (s, 2×CH, Ar), 135.5 (s, CH), 142.2 (s, C_q_, Ar), 143.7 (s, C5), 148.9 (s, C−NO_2_, Ar), 152.5 (s, C6), 164.2 (s, C2), 164.6 (s, C=O). HRMS (ESI^+^): Calcd for C_14_H_11_N_3_O_6_ [M + H]: 318.0726; found 318.0731.

#### 3.3.7. Methyl 6-(3-fluorophenyl)-2-methyl-5-nitronicotinate (**3g**) 

White powder. Yield 0.076 g (87%). Mp 105−107 °C (from ethanol). R_f_: 0.61 (petroleum ether–ethyl acetate 4:1). IR ν_max_ (Film) 3094, 2963, 2848, 1726, 1595, 1533, 1515, 1442, 1304. ^1^H-NMR (CDCl_3_): δ 2.98 (s, 3H, CH_3_), 4.00 (s, 3H, COOCH_3_), 7.20 (tdd, ^3^*J*_H-F_ = 8.4 Hz, ^3^*J*_H-H_ = 2.6 Hz, ^4^*J*_H-H_ = 1.0 Hz, 1H, CH, Ar), 7.30−7.33 (dm, ^3^*J*_H-H_ = 7.7 Hz, 1H, CH, Ar), 7.35−7.38 (dm, ^3^*J*_H-F_ = 9.4 Hz, 1H, CH, Ar), 7.43 (td, ^3^*J*_H-H_ = 8.0 Hz, ^4^*J*_H-H_ = 5.6 Hz, 1H, CH, Ar), 8.71 (s, 1H, C4H); ^19^F-NMR (CDCl_3_): δ −111.8− (−111.9) (m, 1F, CF, Ar). ^13^C-NMR (CDCl_3_): δ 25.3 (s, CH_3_), 53.1 (s, COOCH_3_), 115.8 (d, ^2^*J*_C-F_ = 23.7 Hz, CH, Ar), 117.5 (d, ^2^*J*_C-F_ = 21.5 Hz, CH, Ar), 124.1 (d, ^4^*J*_C-F_ = 3.1 Hz, CH, Ar), 124.4 (s, C3), 130.5 (d, ^3^*J*_C-F_ = 8.1 Hz, CH, Ar), 135.2 (s, C4H), 137.9 (s, d, ^3^*J*_C-F_ = 7.8 Hz, C_q_, Ar), 143.8 (s, C5), 153.1 (d, ^4^*J*_C-F_ = 2.6 Hz, C6), 162.9 (d, ^1^*J*_C-F_ = 247.4 Hz, CF, Ar), 163.7 (s, C2), 164.8 (s, C=O). HRMS (ESI^+^): Calcd for C_14_H_11_FN_2_O_4_ [M + H]: 291.0781; found 291.0788.

#### 3.3.8. Methyl 6-(1,3-diphenyl-1H-pyrazol-4-yl)-2-methyl-5-nitronicotinate (**3h**)

Red yellow powder. Yield 0.098 g (79%). Mp 160−162 °C (from ethanol). R_f_: 0.41 (petroleum ether–ethyl acetate 4:1). ^1^H-NMR (CDCl_3_): δ 2.95 (s, 3H, CH_3_), 3.98 (s, 3H, COOCH_3_), 7.32−7.40 (m, 6H, CH, Ph), 7.48−7.52 (m, 2H, CH, Ph), 7.82−7.85 (m, 2H, CH, Ph), 8.39 (s, 1H, CH, pyrazolyl), 8.67 (s, 1H, C4H); ^13^C-NMR (CDCl_3_): δ 25.3 (s, CH_3_), 53.0 (s, COOCH_3_), 118.4 (s, C_q_, pyrazolyl), 119.6 (s, Ph), 122.6 (s, C3), 127.3 (s, Ph), 127.9 (s, Ph), 128.7 (s, Ph), 128.8 (s, Ph), 129.4 (s, CH, pyrazolyl), 129.70 (s, Ph), 132.53 (s, C_q_, Ph), 135.5 (s, C4H), 139.7 (s, C_q_, Ph), 143.3 (s, C5), 148.3 (s, C6), 152.4 (s, C−Ph, pyrazolyl), 164.1 (s, C2), 164.9 (s, C=O). HRMS (ESI^+^): Calcd for C_23_H_18_N_4_O_4_ [M + H]: 415.1406; found 415.1408.

#### 3.3.9. Diethyl 2,4-dimethyl-6-phenylpyridine-3,5-dicarboxylate (**3k**)

Pale yellow oil. Yield 0.093 g (85%). R_f_: 0.60 (petroleum ether–ethyl acetate 4:1). ^1^H-NMR (CDCl_3_): δ 0.99 (t, ^3^*J*_H-H_ = 7.1 Hz, 3H, COOCH_2_CH_3_), 1.42 (t, ^3^*J*_H-H_ = 7.1 Hz, 3H, COOCH_2_CH_3_), 2.36 (s, 3H, CH_3_), 2.61 (s, 3H, CH_3_), 4.11 (q, ^3^*J*_H-H_ = 7.1 Hz, 2H, COOCH_2_CH_3_), 4.45 (q, ^3^*J*_H-H_ = 7.1 Hz, 2H, COOCH_2_CH_3_), 7.40−7.42 (m, 3H, 3×CH, Ph), 7.55−7.57 (m, 2H, 2×CH, Ph). ^13^C-NMR (CDCl_3_): δ 13.7 (s, COOCH_2_CH_3_), 14.4 (s, COOCH_2_CH_3_), 17.0 (s, CH_3_), 23.3 (s, CH_3_), 61.7 (s, COOCH_2_CH_3_), 61.9 (s, COOCH_2_CH_3_), 127.4 (s, C−COOCH_2_CH_3_), 128.4 (s, 2×CH, Ph), 128.5 (s, 2×CH, Ph), 128.6 (s, C−COOCH_2_CH_3_), 129.0 (s, CH, Ph), 139.8 (s, C_q_, Ph), 143.0 (s, C−CH_3_), 155.4 (s, C6), 156.6 (s, C−CH_3_), 168.50 (s, C=O), 168.53 (s, C=O); MS: *m/z* = 364 [M + 1] (100%). HRMS (ESI^+^): Calcd for C_19_H_19_F_2_NO_4_ [M + H]: 364.1360; found 364.1366.

### 3.4. General Procedure for the Synthesis of Methyl 2-(fluoromethyl)-5-nitro-6-arylnicotinates **5a**–**d**

**Method A:** To a stirred solution of 3-fluoro-5-nitro-3,6-dihydropyridines **2a,c,f,g** (0.5 mmol) in dry acetonitrile (5 mL) in the presence of 3 Å molecular sieves cooled to 0 °C, a solution of Selectfluor^®^ (0.340 g, 1 mmol) in dry acetonitrile (10 mL) was added in portions under an argon atmosphere. The reaction mixture was stirred for 10 min at 0 °C, after which the temperature was slowly raised to room temperature. The reaction mixture was stirred for 48 h at room temperature under an argon atmosphere, then volatile compounds were evaporated in vacuo, diethyl ether (15 mL) was added to the residue and the precipitate was filtered. According to the LC–MS data, the filtrate contained a mixture of 2-(fluoromethyl)pyridines **5a**–**d** and pyridines **3a,c,f,g**. The filtrate was evaporated in vacuo and the residue was separated by column chromatography to give methyl 2-(fluoromethyl)-5-nitro-6-arylnicotinates **5a**–**d** in 21−43% yields and 2-methylpyridines **3a,c,f,g** in 10–52% yields.

**Method B:** To a stirred solution of 1,2-dihydropyridines **1a,c,f,g** (0.5 mmol) in dry acetonitrile (5 mL) in the presence of 3 Å molecular sieves cooled to 0 °C, a solution of Selectfluor^®^ (0.53 g, 1.5 mmol) in dry acetonitrile (10 mL) was added dropwise under an argon atmosphere. The reaction mixture was stirred for 10 min at 0 °C, after which the temperature was slowly raised to room temperature. The reaction mixture was stirred for 48 h at room temperature under an argon atmosphere. Then the reaction mixture was evaporated in vacuo, diluted with diethyl ether (15 mL) and the insoluble precipitate was filtered. The filtrate was evaporated in vacuo to give a mixture of 2-(fluoromethyl)pyridines **5a**–**d** and pyridines **3a,c,f,g** in similar ratios as in Method A according to the ^1^H-NMR spectra and LC–MS data (Table 5).

Isolated yields for 2-(fluoromethyl)pyridines **5a–d** and pyridines **3a,c,f,g** obtained by Method A are given.

#### 3.4.1. Methyl 2-(fluoromethyl)-5-nitro-6-phenylnicotinate (**5a**) 

Pale yellow oil. Yield 0.046 g (32%). R_f_: 0.45 (petroleum ether–ethyl acetate 4:1). Anal. calcd for C_14_H_11_FN_2_O_4_: C, 57.93; H, 3.82; N, 9.65; found: C, 58.06; H, 3.93; N, 9.52. ^1^H-NMR (CDCl_3_): δ 4.02 (s, 3H, COOCH_3_), 5.93 (d, ^2^*J*_H-F_ = 46.9 Hz, 2H, CH_2_F), 7.49−7.51 (m, 3H, 3×CH, Ph), 7.65−6.67 (m, 2H, 2×CH, Ph), 8.71 (d, ^5^*J*_H-F_ = 1.1 Hz, 1H, C4H). ^19^F-NMR (CDCl_3_): δ −219.4 (t, ^2^*J*_F-H_ = 46.9 Hz, 1F, FCH_2_). ^13^C-NMR (CDCl_3_): δ 53.4 (s, COOCH_3_), 82.9 (d, ^1^*J*_C-F_ = 174.4 Hz, FCH_2_), 123.1 (s, C−COOCH_3_), 128.7 (s, Ph), 129.1 (s, Ph), 131.0 (s, Ph), 135.15 (s, C_q_, Ph), 135.20 (s, C4H), 144.9 (s, C5), 154.8 (s, C6), 159.2−156-4 (m, C2), 164.1 (s, C=O). MS: *m/z* = 291 [M + 1] (100%).

#### 3.4.2. Methyl 2-(fluoromethyl)-6-(2-methoxyphenyl)-5-nitronicotinate (**5b**)

White powder. Yield 0.034 g (21%). Mp 143−145 °C (from ethanol). R_f_: 0.38 (petroleum ether–ethyl acetate 4:1). IR ν_max_ (Film) 3095, 2953, 2924, 2848, 1736, 1595, 1562, 1521, 1494, 1466, 1437, 1351, 1304. Anal. calcd for C_15_H_13_FN_2_O_5_: C, 56.25; H, 4.09; N, 8.75; found: C, 56.39; H, 4.19; N, 8.64. ^1^H-NMR (CDCl_3_): δ 3.72 (s, 3H, OCH_3_), 4.01 (s, 3H, COOCH_3_), 5.92 (d, ^2^*J*_H-F_ = 46.9 Hz, 2H, CH_2_F), 6.90−6.92 (m, 1H, CH, Ar), 7.15−7.19 (m, 1H, CH, Ar), 7.46−7.50 (m, 1H, CH, Ar), 7.80−7.82 (m, 1H, CH, Ar), 8.77 (d, ^5^*J*_H-F_ = 1.1 Hz, 1H, C4H). ^19^F-NMR (CDCl_3_): δ −219.1 (t, ^2^*J*_F-H_ = 46.9 Hz, 1F, FCH_2_); ^13^C-NMR (CDCl_3_): 53.3 (s, COOCH_3_), 55.10 (s, OCH_3_), 82.9 (d, ^1^*J*_C-F_ = 173.91 Hz, FCH_2_), 110.8 (s, CH, Ar), 121.8 (s, CH, Ar), 123.1 (s, C3), 125.3 (s, C_q_, Ar), 131.4 (s, CH, Ar), 131.5 (s, CH, Ar), 134.6 (s, C4H), 145.9 (s, C5), 152.6 (s, C6), 156.7 (s, C−OMe, Ar), 159.2 (d, ^2^*J*_C-F_ = 15.4 Hz, C2), 164.3 (s, C=O). MS: *m/z* = 321 [M + 1] (100%). 

#### 3.4.3. Methyl 2-(fluoromethyl)-5-nitro-6-(4-nitrophenyl)nicotinate (**5c**)

White powder. Yield 0.049 g (38%). Mp 150−151 °C (from ethanol). R_f_: 0.43 (petroleum ether–ethyl acetate 4:1). IR ν_max_ (Film) 3101, 2958, 2860, 1728, 1596, 1555, 1522, 1437, 1351, 1300. ^1^H-NMR (CDCl_3_): δ 4.04 (s, 3H, COOCH_3_), 5.94 (d, ^2^*J*_H-F_ = 46.6 Hz, 2H, CH_2_F), 7.79−7.82 (m, 2H, 2×CH, Ar), 8.33−8.37 (m, 2H, 2×CH, Ar), 8.83 (d, ^5^*J*_H-F_ = 1.1 Hz, 1H, CH). ^19^F-NMR (CDCl_3_): δ −220.1 (t, ^2^*J*_F-H_ = 46.6 Hz, 1F, FCH_2_). ^13^C-NMR (CDCl_3_): δ 53.6 (s, COOCH_3_), 82.6 (d, ^1^*J*_C-F_ = 175.8 Hz, FCH_2_), 124.1 (s, 2×CH, Ar), 124.6 (s, C3), 129.9 (s, 2×CH, Ar), 135.6 (s, C4H), 141.4 (s, C5), 145.0 (s, C_q_, Ar), 149.1 (s, C−NO_2_, Ar), 152.8 (s, C6), 159.8 (d, ^2^*J*_C-F_ = 15.0 Hz, C2), 163.6 (s, C=O); MS: *m/z* = 336 [M + 1] (100%). HRMS (ESI^+^): Calcd for C_14_H_10_FN_3_O_6_ [M + H]: 336.0632; found 336.0634.

#### 3.4.4. Methyl 2-(fluoromethyl)-6-(3-fluorophenyl)-5-nitronicotinate (**5d**) 

White powder. Yield 0.057 g, (43%). Mp 107−109 °C (from ethanol). R_f_: 0.42 (petroleum ether–ethyl acetate 4:1). IR ν_max_ (Film) 3098, 2976, 2919, 2861, 1714, 1597, 1557, 1519, 1436, 1351, 1309. ^1^H-NMR (CDCl_3_): δ 4.02 (s, 3H, COOCH_3_), 5.93 (d, ^2^*J*_H-F_ = 46.6 Hz, 2H, CH_2_F), 7.20−7.25 (m, 1H, CH, Ar), 7.36−7.48 (m, 3H, 3×CH, Ar), 8.73 (d, ^5^*J*_H-F_ = 0.6 Hz, 1H, CH). ^19^F-NMR (CDCl_3_): δ −111.45–−111.51 (m, 1F, CF, Ar), −219.6 (t, ^2^*J*_F-H_ = 46.6 Hz, 1F, FCH_2_). ^13^C-NMR (CDCl_3_): δ 54.5 (s, COOCH_3_), 82.7 (d, ^1^*J*_C-F_ = 174.7 Hz, FCH_2_), 116.0 (d, ^2^*J*_C-F_ = 23.5 Hz, CH, Ar), 118.0 (d, ^2^*J*_C-F_ = 20.9 Hz, CH, Ar), 123.8 (s, CH, Ar), 124.3 (d, ^3^*J*_C-F_ = 3.2 Hz, C3), 130.7 (d, ^3^*J*_C-F_ = 8.2 Hz, CH, Ar), 135.3 (s, CH), 137.2 (d, ^3^*J*_C-F_ = 7.1 Hz, C_q_, Ar), 145.0 (s, C5), 153.4 (d, ^4^*J*_C-F_ = 3.1 Hz, C6), 159.3 (d, ^2^*J*_C-F_ = 15.9 Hz, C2), 163.0 (d, ^1^*J*_C-F_ = 247.4 Hz, CF, Ar), 163.9 (s, C=O). MS: *m/z* = 309 [M + 1] (100%). HRMS (ESI^+^): Calcd for C_14_H_10_F_2_N_2_O_4_ [M + H]: 309.0687; found 309.0692.

## 4. Conclusions

A series of new fluorinated 3,6-dihydropyridines **2a**–**k** can be obtained by the reaction of 1,2-dihydropyridines **1a**–**k** with Selectfluor^®^. According to quantum chemical calculations, the 3,6-dihydropyridine ring of 3-fluoro-3,6-dihydropyridines **2a**–**k** was shown to be planar or near planar. The large values of the long-range ^5^*J* (^1^H,^19^F) coupling constants registered in the ^1^H and ^19^F-NMR spectra of compounds **2** were apparently due to the homoallyl long-range coupling transmitted through *π*-electrons across a double bond of the heterocycle rather than by through-space interaction. The elimination of hydrogen fluoride under mild conditions can easily convert 3-Fluoro-3,6-dihydropyridines **2a**–**k** to corresponding pyridines **3a**–**k**. A new approach to the synthesis of methyl 2-(fluoromethyl)-5-nitro-6-arylnicotinates **5a**–**d** by the reaction of 3-fluoro-2-methyl-5-nitro-3,6-dihydropyridines **2a,c,f,g** or 1,2-dihydropyridines **1a,c,f,g** with Selectfluor^®^ has been also proposed.

## Figures and Tables

**Table 1:**
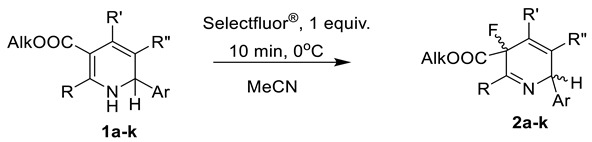
The reaction of 1,2-dihydropyridines **1a**–**k** with Selectfluor^®^.

Comp.	R	Alk	R′	R″	Ar	The Ratio of Diastereomers, % ^1^	Yield, %^2^
**2a**	Me	Me	H	NO_2_	Ph	45:55	94
**2b**	Me	Me	H	NO_2_	Ph-Ph	40:60	96
**2c**	Me	Me	H	NO_2_	*o*-OMe-Ph	45:55	90
**2d**	Me	Me	H	NO_2_	3,4,5-OMe-Ph	20:80	90
**2e**	Et	Me	H	NO_2_	*o*-OMe-Ph	50:50	97
**2f**	Me	Me	H	NO_2_	*p*-NO_2_-Ph	50:50	94
**2g**	Me	Me	H	NO_2_	*m*-F-Ph	50:50	88
**2h**	Me	Me	H	NO_2_	1,3-Ph-*1H*-pyrazol-4-yl	10:90	89
**2k**	Me	Et	Me	COOEt	Ph	15:85	82

^1^ The ratio of diastereomers is determined according to the ^19^F, ^1^H-NMR spectra of compounds **2a**–**k**. ^2^ The yields of **2a**–**k** after extraction with diethyl ether are given.

**Table 2:**
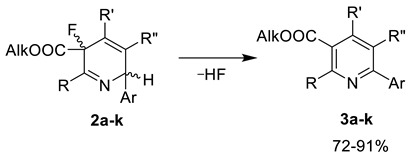
The formation of pyridines **3a**–**k** from 3-fluoro-3,6-dihydropyridines **2a**–**k** after the elimination of hydrogen fluoride.

Comp.	R	Alk	R′	R″	Ar	Yield, %^1^
**3a**	Me	Me	H	NO_2_	Ph	91
**3b**	Me	Me	H	NO_2_	Ph-Ph	85
**3c**	Me	Me	H	NO_2_	*o*-OMe-Ph	72
**3d**	Me	Me	H	NO_2_	3,4,5-OMe-Ph	84
**3e**	Et	Me	H	NO_2_	*o*-OMe-Ph	82
**3f**	Me	Me	H	NO_2_	*p*-NO_2_-Ph	93
**3g**	Me	Me	H	NO_2_	*m*-F-Ph	87
**3h**	Me	Me	H	NO_2_	1,3-Ph-*1H*-pyrazol-4-yl	79
**3k**	Me	Et	Me	COOEt	Ph	85

^1^ Isolated yields are given.

**Table 3:** The general NMR spectral characteristics of 3-fluoro-3,6-dihydropyridines **2a**–**k**.

Comp.	The Ratio of Diastereo-mers, %^1^	δ C6H Proton for Both Diastereomers in ^1^H-NMR Spectra, ppm		δ F for both Diastereomers in ^19^F-NMR Spectra, ppm		Homoallylic Coupling Constant ^5^*J*_H-F(F-H)_ for Both Diastereomers, Hz^2^		^3^*J*_H-F(F-H)_ for Both Diastereomers, Hz^2^		Allylic Coupling Constant ^4^*J*_H-H_ for both Diastereomers, Hz^3^	
		Minor	Major	Minor	Major	Minor	Major	Minor	Major	Minor	Major
**2a**	45:55	6.09	5.79	−140.6	−142.4	15.4	12.1	9.4	5.6	1.3	1.2
**2b**	40:60	6.22	5.94	−140.3	−142.4	15.4	12.1	9.3	5.6	1.2	1.2
**2c**	45:55	6.70	6.22	−139.9	−144.3	16.6	12.6	9.0	6.2	1.4	1.6
**2d**	20:80	5.79	6.02	−141.5	−140.4	12.2	15.8	5.4	9.2	1.1	1.2
**2e**	50:50	6.72	6.21	−140.5	−145.1	17.0	12.6	9.1	6.5	1.2	1.6
**2f**	50:50	6.26	5.94	−141.6	−143.0	14.4	11.4	9.5	5.7	1.1	1.3
**2g**	50:50	6.16	5.85	−140.7	−142.8	14.9	11.8	9.4	5.6	1.1	1.2
**2h**	10:90	6.05	6.30	−144.2	−142.6	11.9	14.0	5.8	9.5	1.5	1.1
**2k**	15:85	5.38	5.70	−150.4	−145.9	12.0	15.5	-	-	-	-

^1^ The ratio of diastereomers is determined according to the ^19^F, ^1^H-NMR spectra of compounds **2a**–**k**. ^2^ Determined according to the ^19^F, ^1^H-NMR spectra of compounds **2a**–**k**. ^3^ Determined according to the ^1^H-NMR spectra of compounds **2a**–**k**.

**Table 4:** Quantum chemical calculation data for compounds **2a**–**k**.

Comp.	d(H…F), Å		d(C6 to N1-C2-C4-C5 plane), Å		d(C3 to N1-C2-C4-C5 plane), Å		The angle between C6-N1-C2-C3 and C6-C5-C4-C3 Planes, °	
	*cis*	*trans*	*cis*	*trans*	*cis*	*trans*	*cis*	*trans*
**2a**	4.60	4.73	0.11	0.11	0.12	0.13	169.38	168.88
**2b**	4.60	4.85	0.11	0.02	0.13	0.05	169.18	178.46
**2c**	4.59	4.85	0.10	0.01	0.13	0.08	169.72	175.66
**2d**	4.61	4.72	0.12	0.12	0.13	0.15	168.90	167.86
**2e**	4.57	4.77	0.09	0.07	0.12	0.07	170.45	173.73
**2f**	4.59	4.85	0.10	0.03	0.13	0.10	169.74	174.07
**2g**	4.60	4.85	0.11	0.01	0.13	0.08	169.40	175.94
**2h**	4.63	4.77	0.13	0.08	0.14	0.08	167.81	172.97
**2k**	4.98	4.80	0.30	0.02	0.42	0.05	148.20	176.79

**Table 5:**
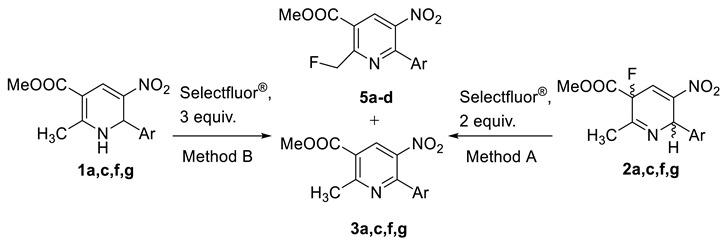
Reactions of 3-fluoro-2-methyl-5-nitro-3,6-dihydropyridines **2a,c,f,g** or 1,2-dihydropyridines **1a,c,f,g** with Selectfluor^®^, leading to the formation of a mixture of methyl 2-(fluoromethyl)-5-nitro-6-arylnicotinates **5a**–**d** and 2-methylpyridines **3a,c,f,g**.

Comp.	Ar	Ratio 3:5, %^1^		Yields, %^2^	
		Method A	Method B	Comp. 3	Comp. 5
**3a, 5a**	Ph	50:50	55:45	44	32
**3c, 5b**	*o*-OMe-Ph	60:40	55:45	52	21
**3f, 5c**	*p*-NO_2_-Ph	15:75	20:80	10	38
**3g, 5d**	*m*-F-Ph	50:50	50:50	36	43

^1^ The ratio of compounds **3a,c,f,g** to **5a–d** were determined according to the ^1^H-NMR spectra and LC–MS data of the reaction mixture. ^2^ Isolated yields obtained by Method A are given.

## Supplementary Materials

The following are available online: one-dimensional ^1^H, ^19^F, ^13^C and two-dimensional {^1^H−^1^H} COSY, {^13^C−^1^H} HSQC, {^13^C−^1^H} HMBC-NMR spectral data of compounds **2a**–**k**, **3a**–**k** and **5a**–**d** (file type pdf), the optimised structures of compounds **2a**–**k** are provided in Gaussian output files.
