# Supplementary material for: Synthesis of Fluorinated 3,6-Dihydropyridines and 2-(Fluoromethyl)pyridines by Electrophilic Fluorination of 1,2-Dihydropyridines with Selectfluor®
Source: Molecules. 2020 Jul 9;25(14):3143. doi: 10.3390/molecules25143143 (PMC7397266; doi:10.3390/molecules25143143)

Synthesis of fluorinated 3,6-dihydropyridines and 2-(fluoromethyl)pyridines by electrophilic fluorination of 1,2-dihydropyridines with Selectfluor®

Nadiia V. Pikun [1,*], Arkadij Sobolev [1], Aiva Plotniece [1], Martins Rucins [1], Brigita Vigante [1], Marina Petrova [1], Ruslan Muhamadejev [1], Karlis Pajuste [1], and Yuriy G. Shermolovich [2]

[1] Latvian Institute of Organic Synthesis, Aizkraukles Str. 21, Riga, LV-1006, Latvia; arkady@osi.lv (A.S.); aiva@osi.lv (A.P.); rucins@osi.lv (M.R.); vigante@osi.lv (B.V.); marina@osi.lv (M.P.); muhamadejev@osi.lv (R.M.); kpajuste@osi.lv (K.P.)

[2] Institute of Organic Chemistry NAS of Ukraine, Murmanska Str. 5, 02660, Kyiv, Ukraine; sherm@ioch.kiev.ua (Y.S.)

* Correspondence: nadiia@osi.lv; Tel.: +371-67014928 (N.P.)

Abstract: New fluorinated 3,6-dihydropyridines were obtained by electrophilic fluorination of 1,2-dihydropyridines with Selectfluor®. These 3-fluoro-3,6-dihydropyridines were easily converted to corresponding pyridines by the elimination of hydrogen fluoride under mild conditions. A new approach to the synthesis of methyl 2-(fluoromethyl)-5-nitro-6-arylnicotinates by fluorination of 3-fluoro-2-methyl-5-nitro-3,6-dihydropyridines or 1,2-dihydropyridines with Selectfluor® has been developed.

Table of Contents

NMR spectra 2

^{1}H NMR spectrum of compound **2a**

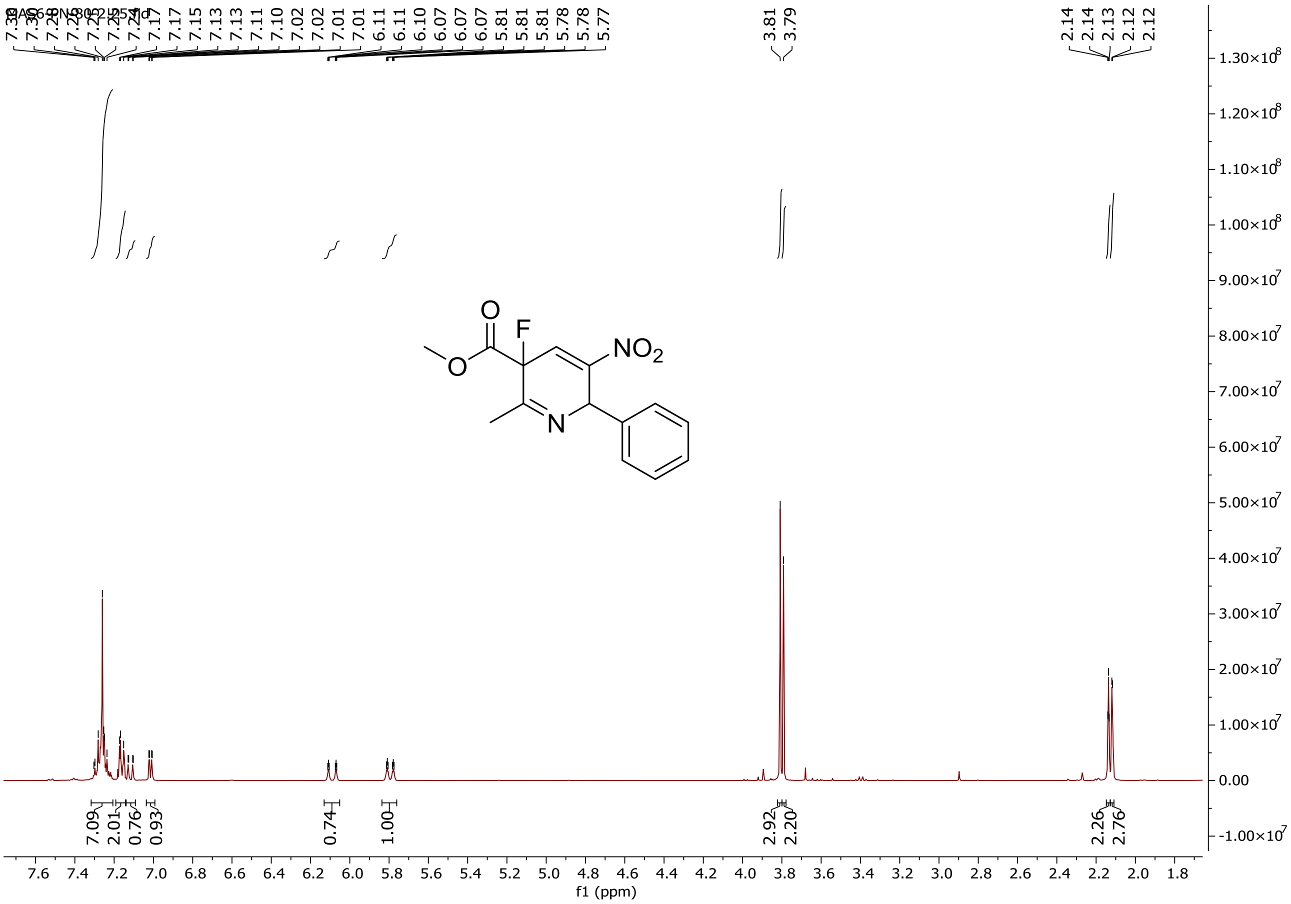

^{19}F NMR spectrum of compound **2a**

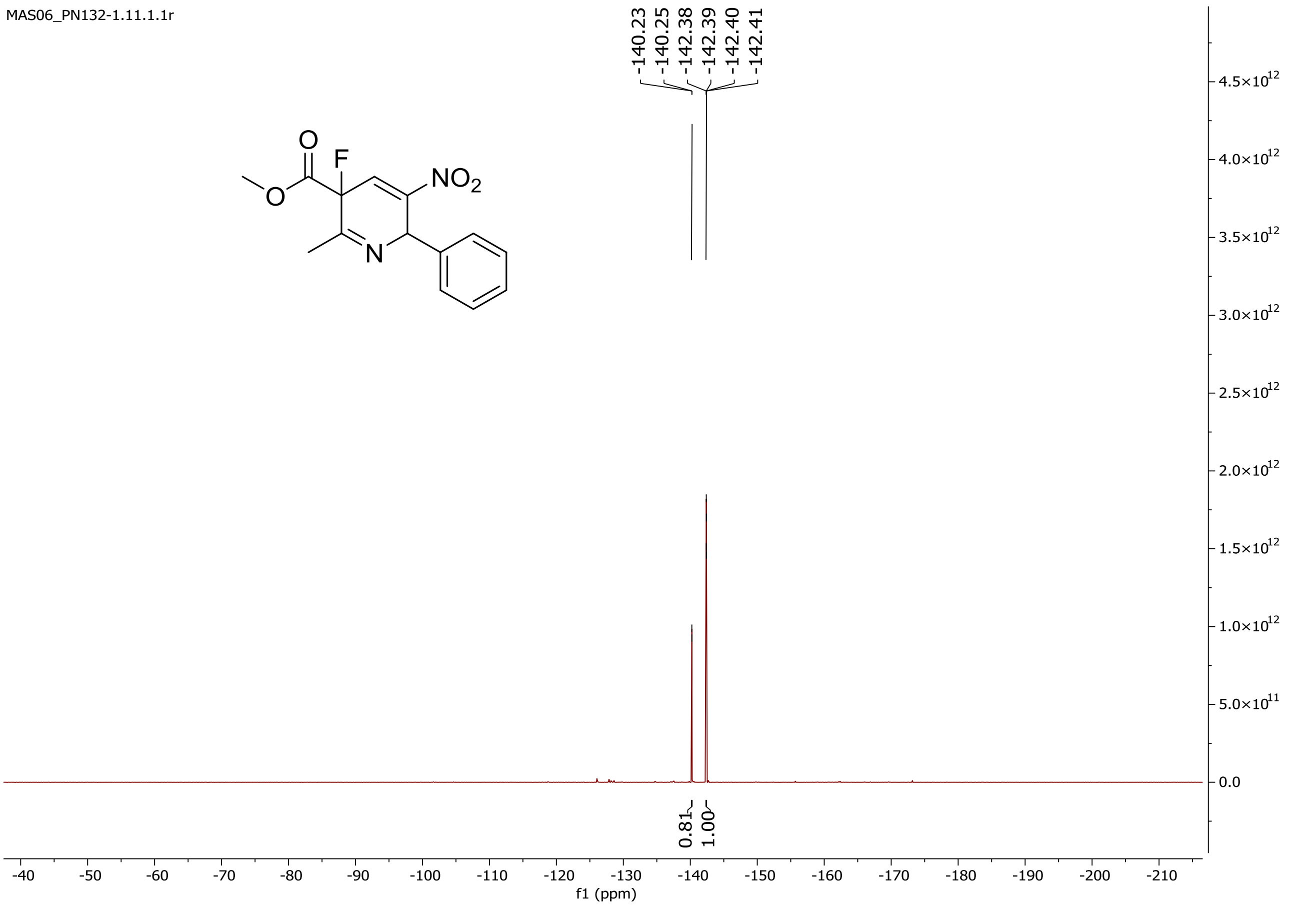

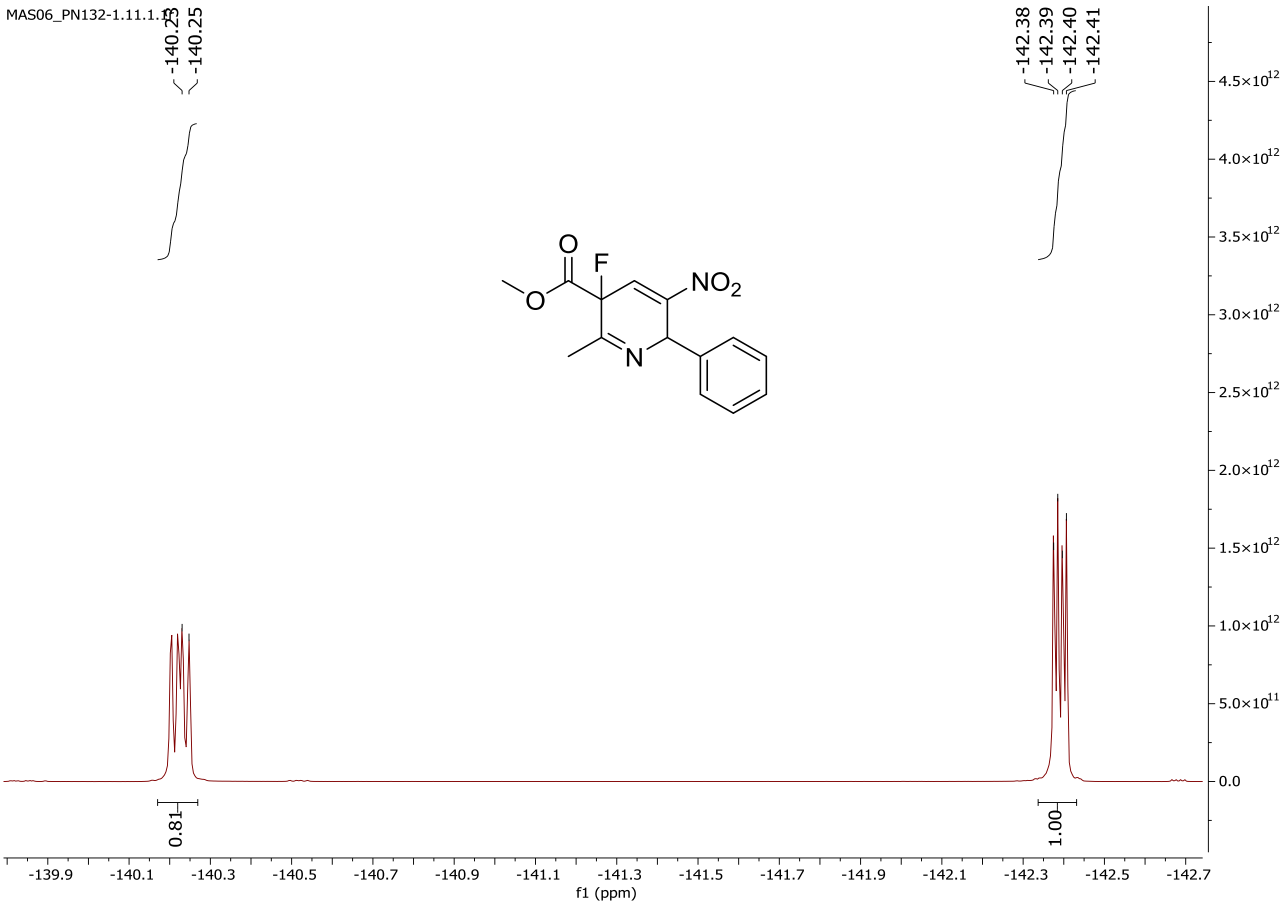

^{19}F NMR spectrum with ^{1}H decoupling of compound **2a**

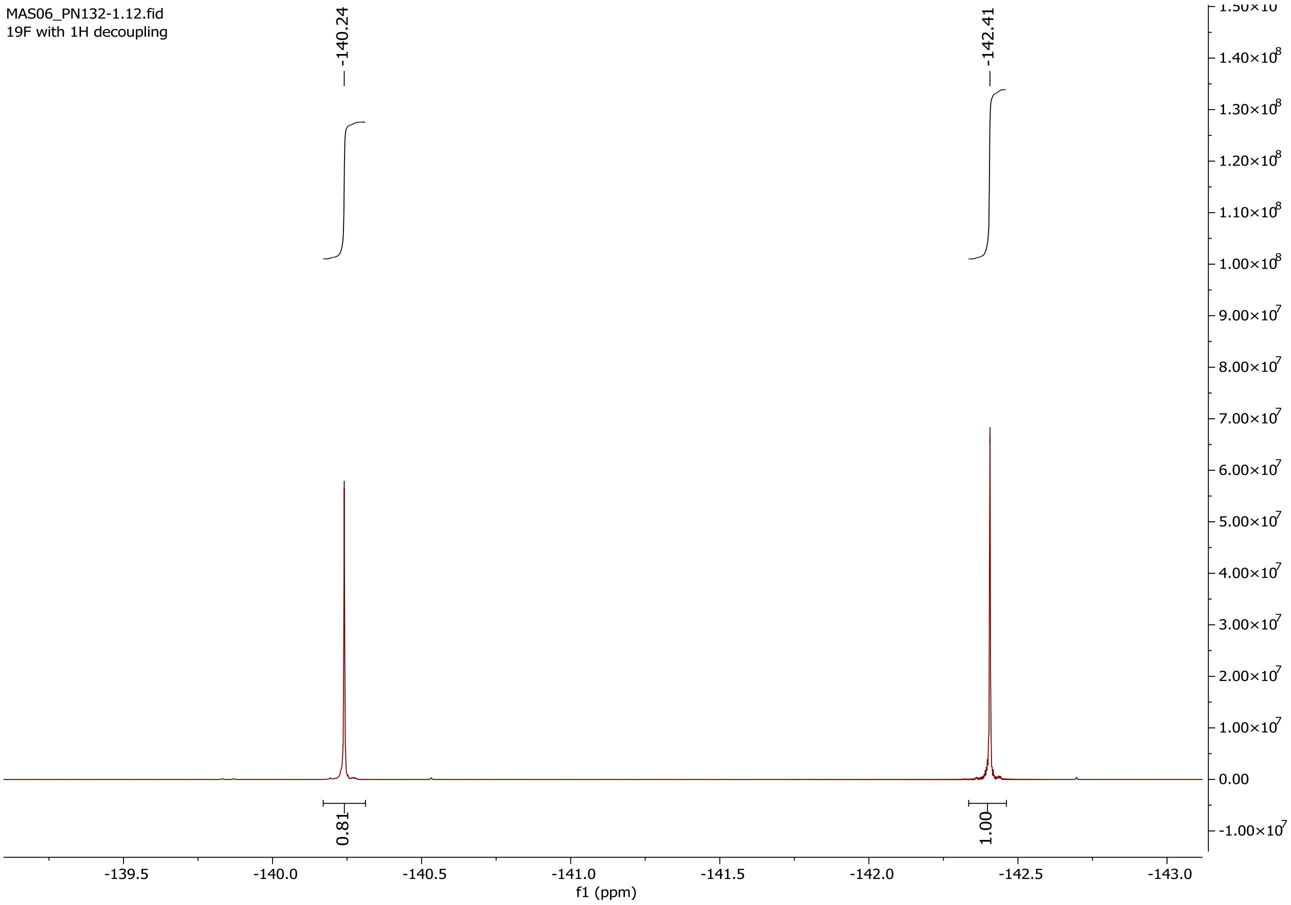

^{13}C NMR spectrum of compound **2a**

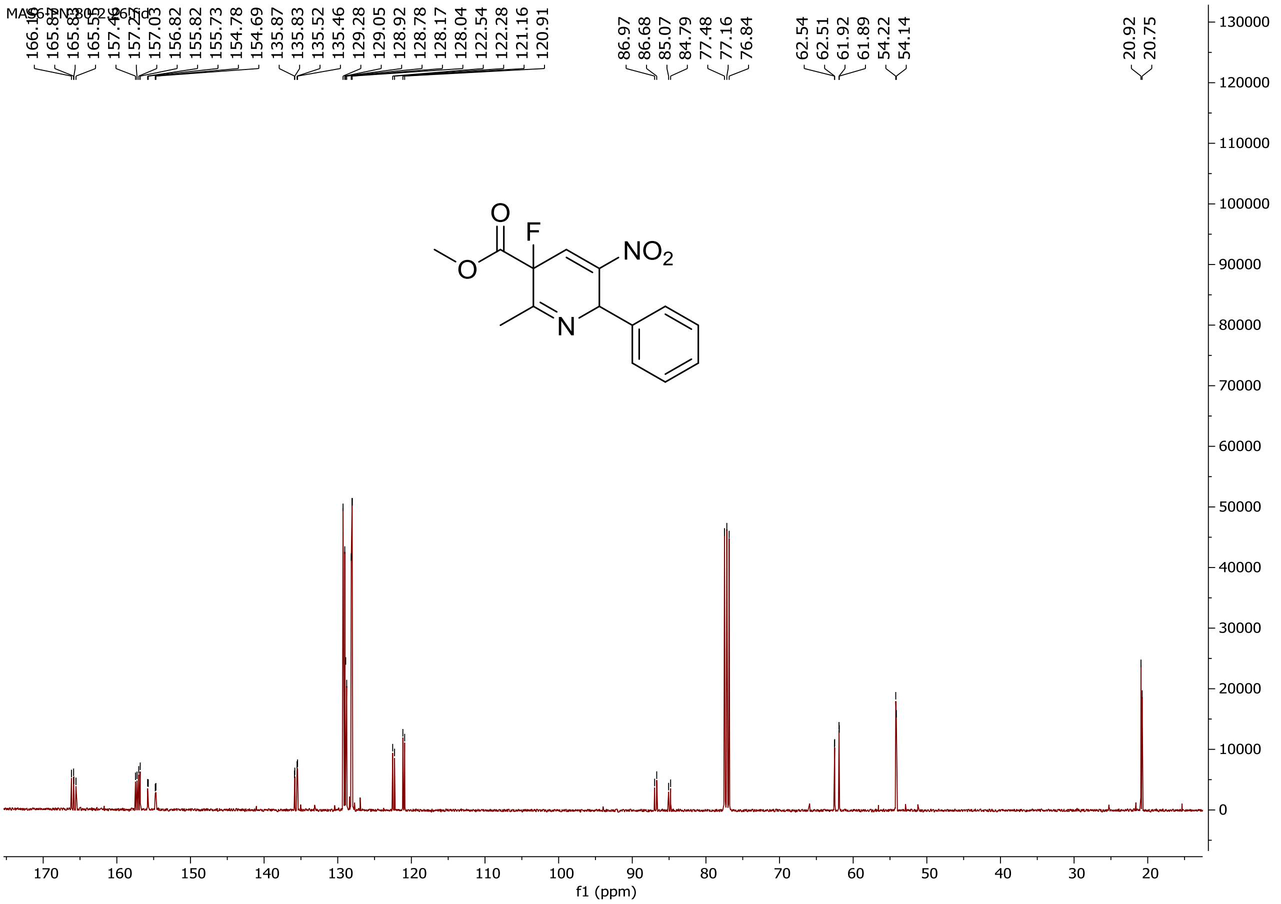

{^{13}C–^{1}H} HSQC spectrum of compound **2a**

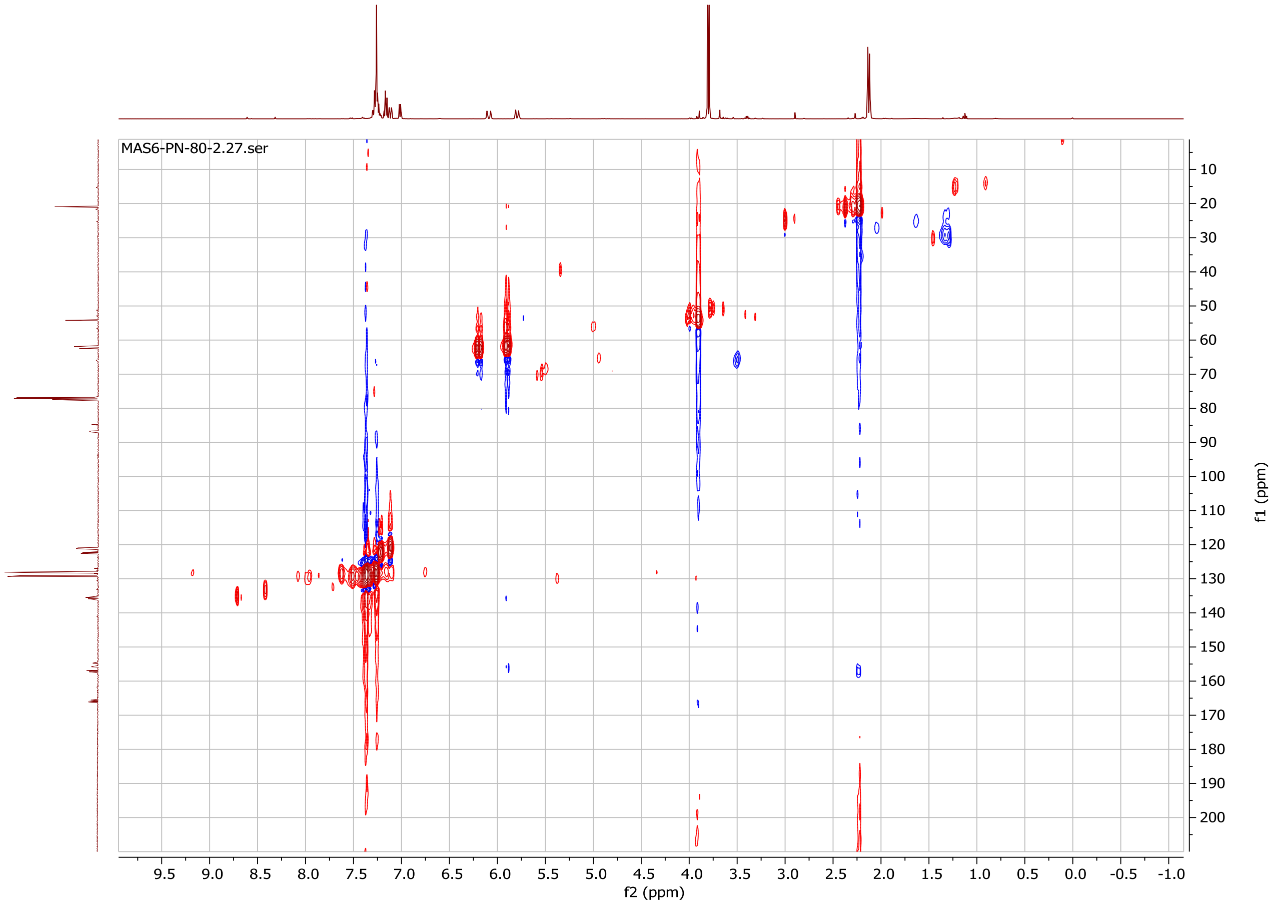

{^{13}C–^{1}H} HMBC spectrum of compound **2a**

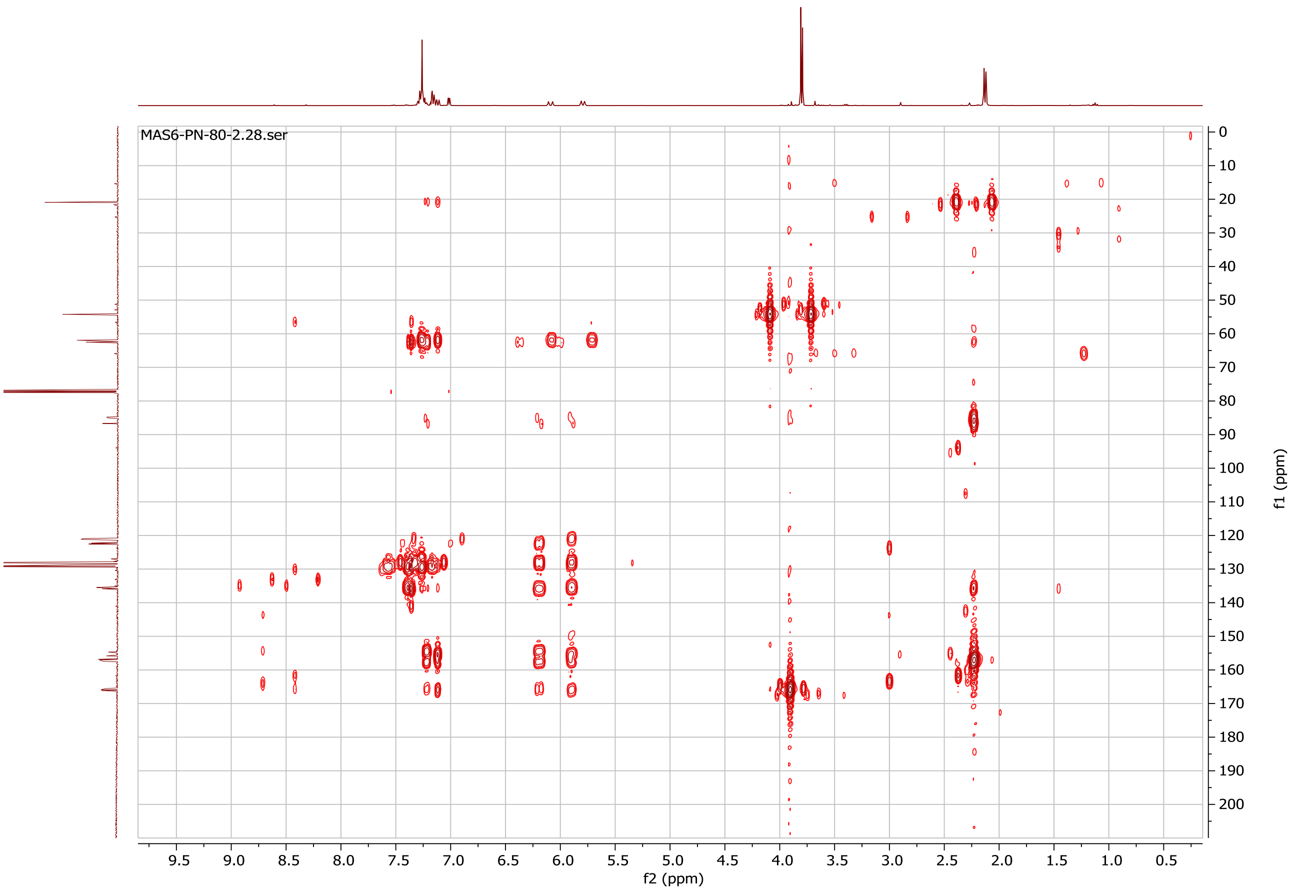

^{1}H NMR spectrum of compound **2b**

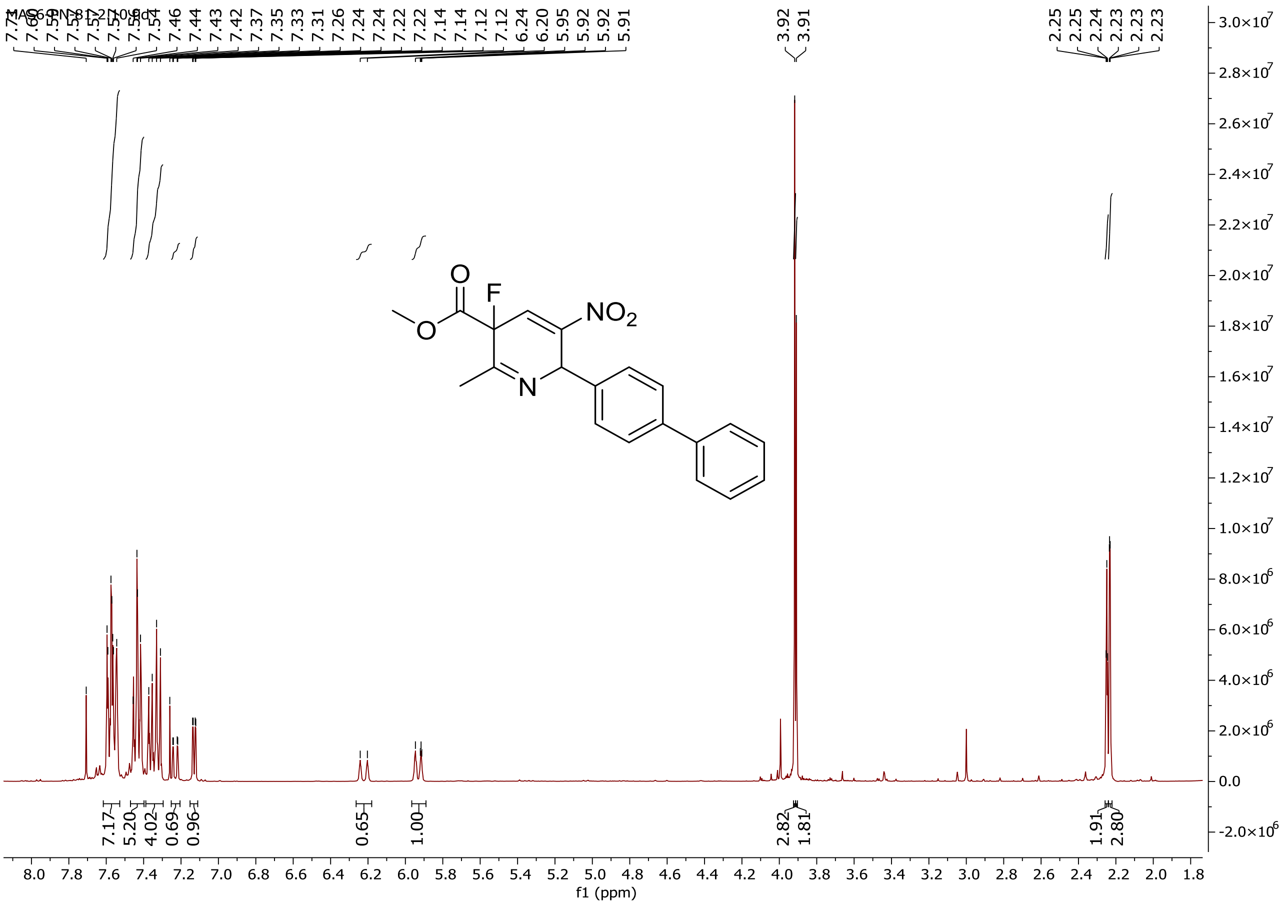

^{19}F NMR spectrum of compound **2b**

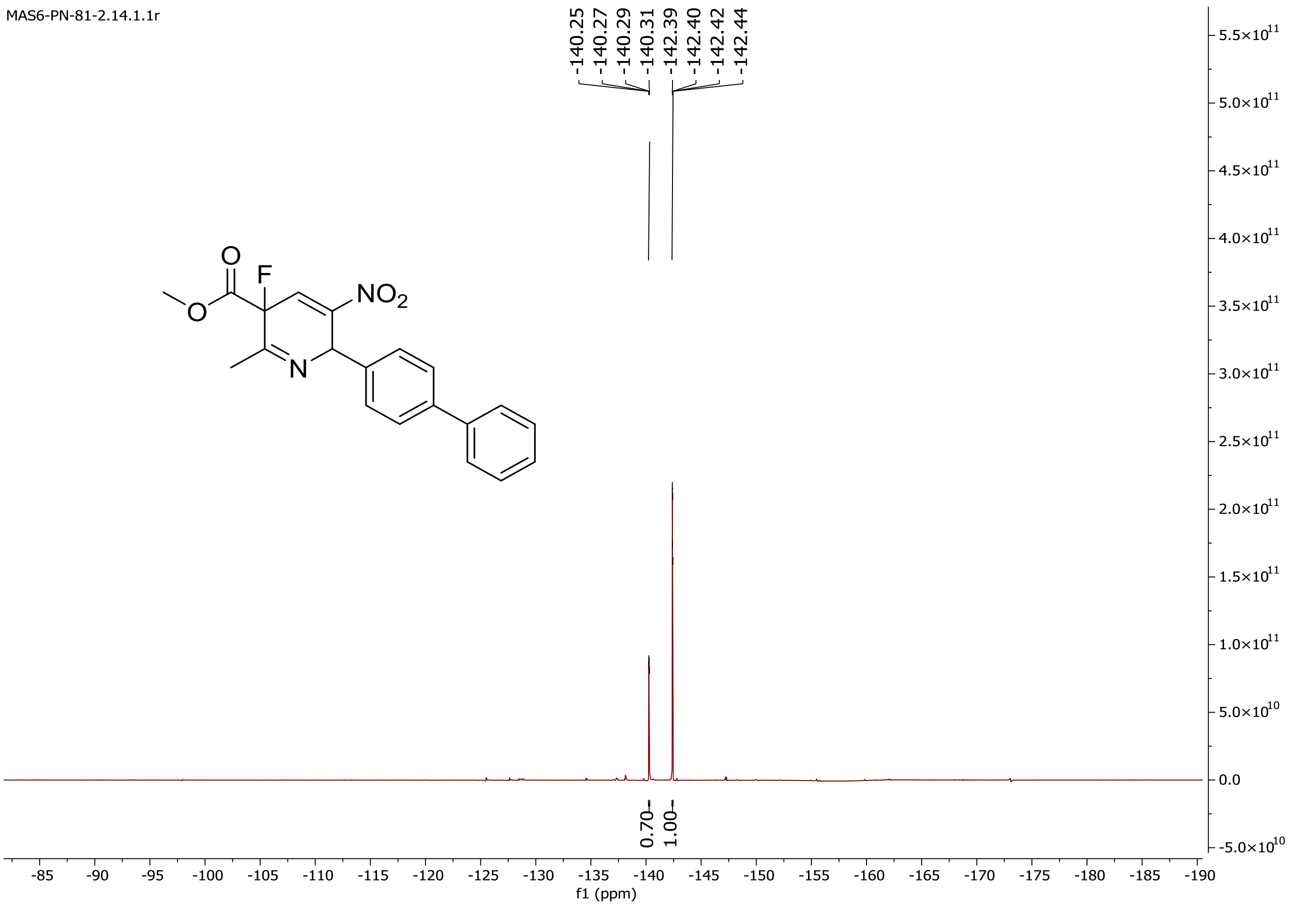

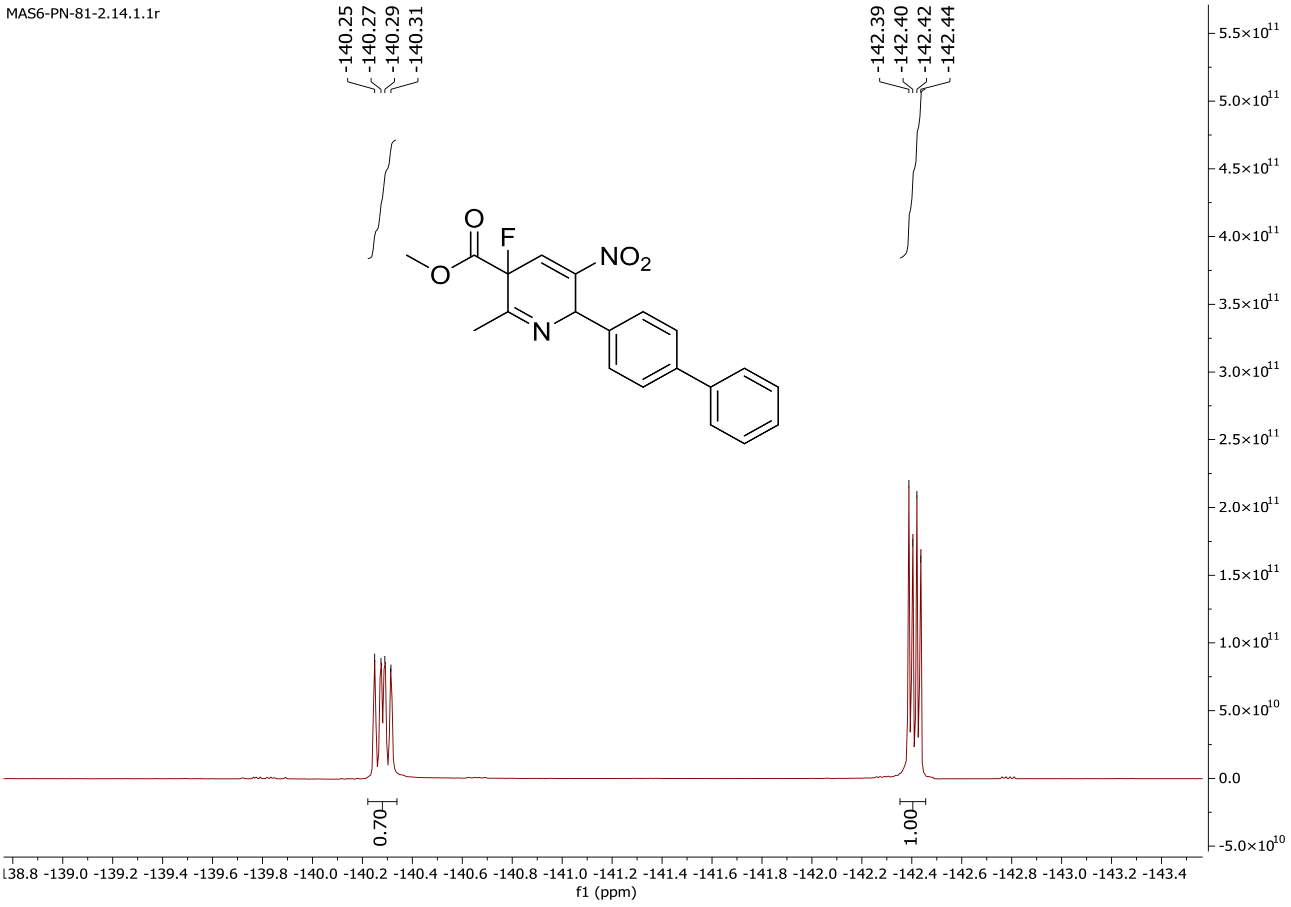

^{13}C NMR spectrum of compound **2b**

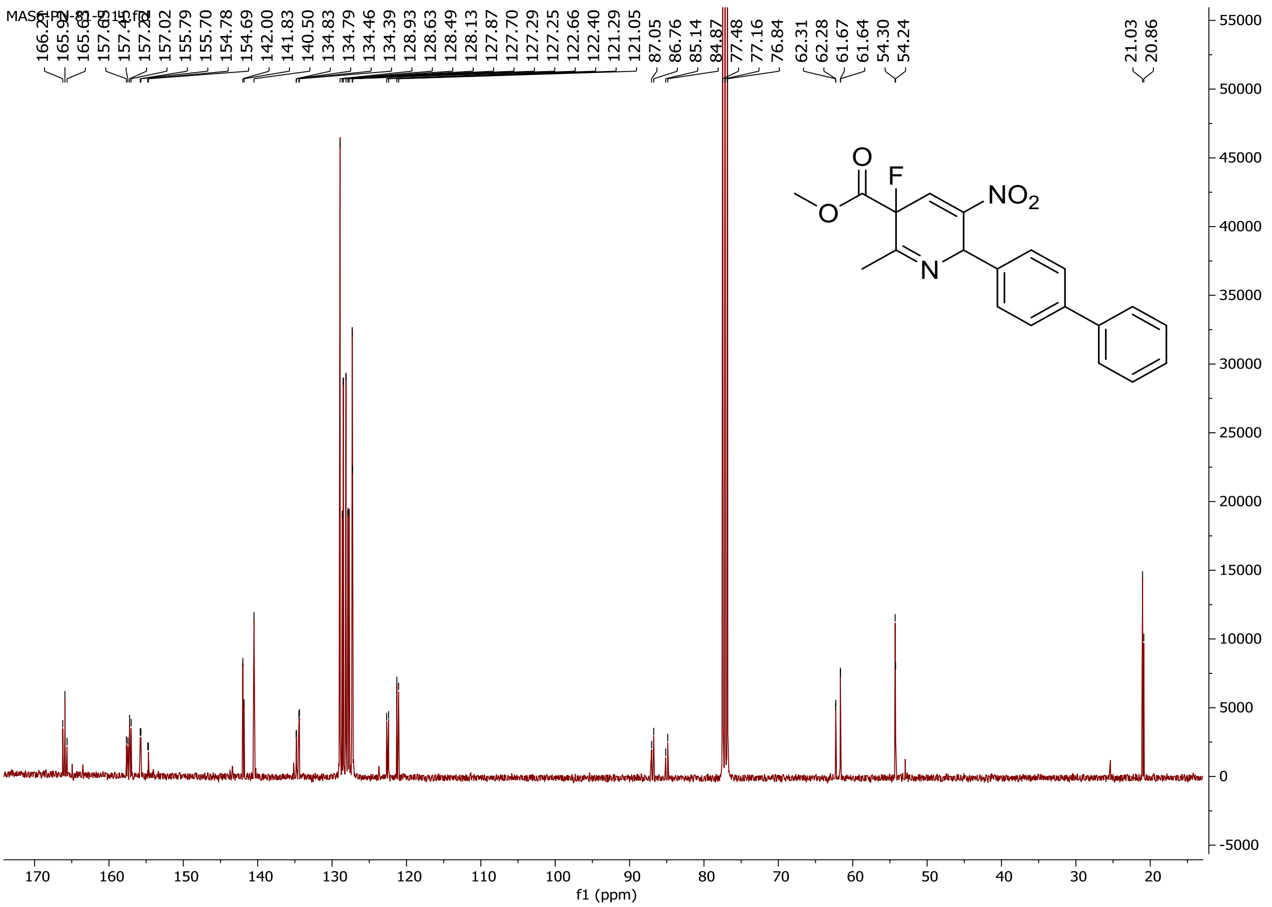

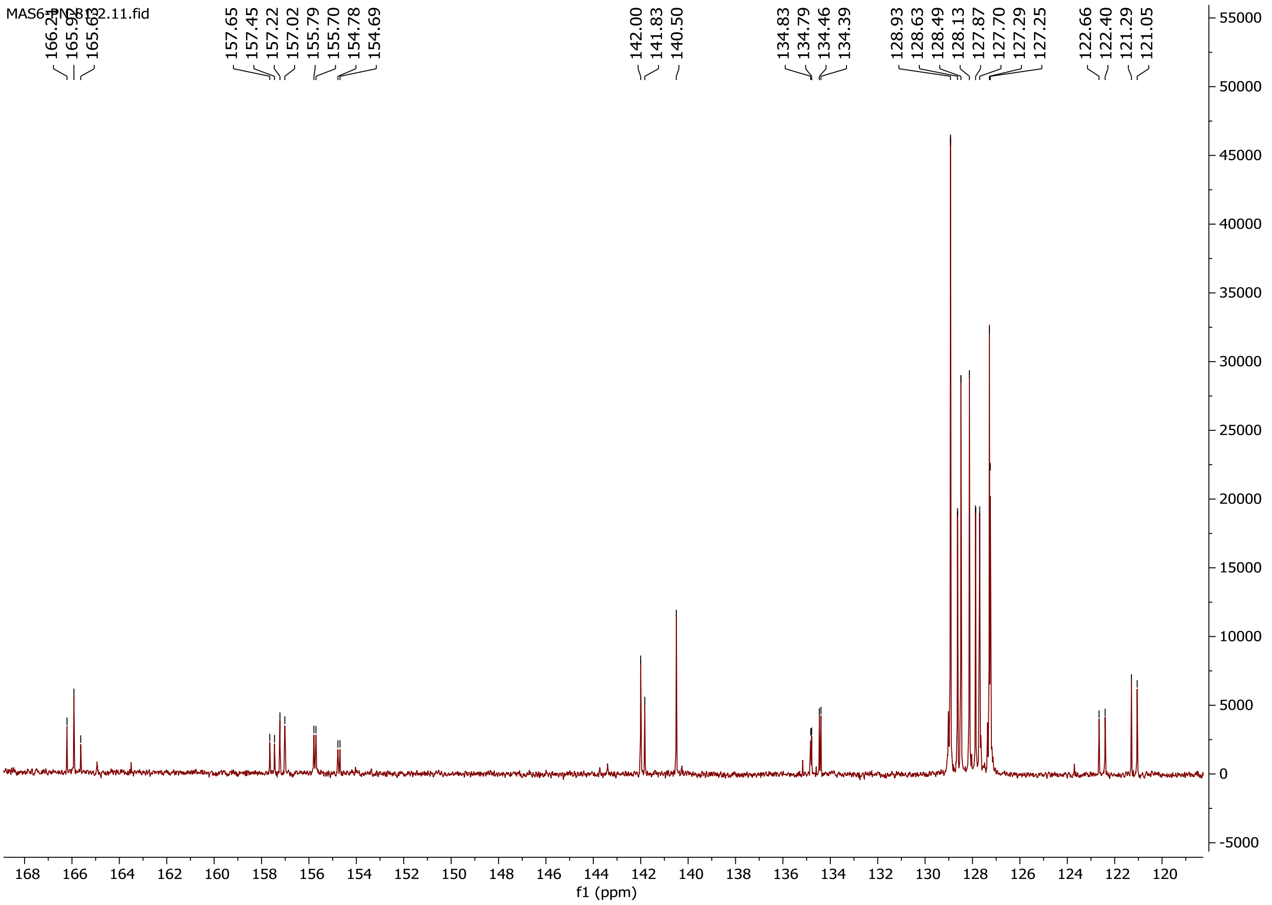

{^{13}C–^{1}H} HSQC spectrum of compound **2b**

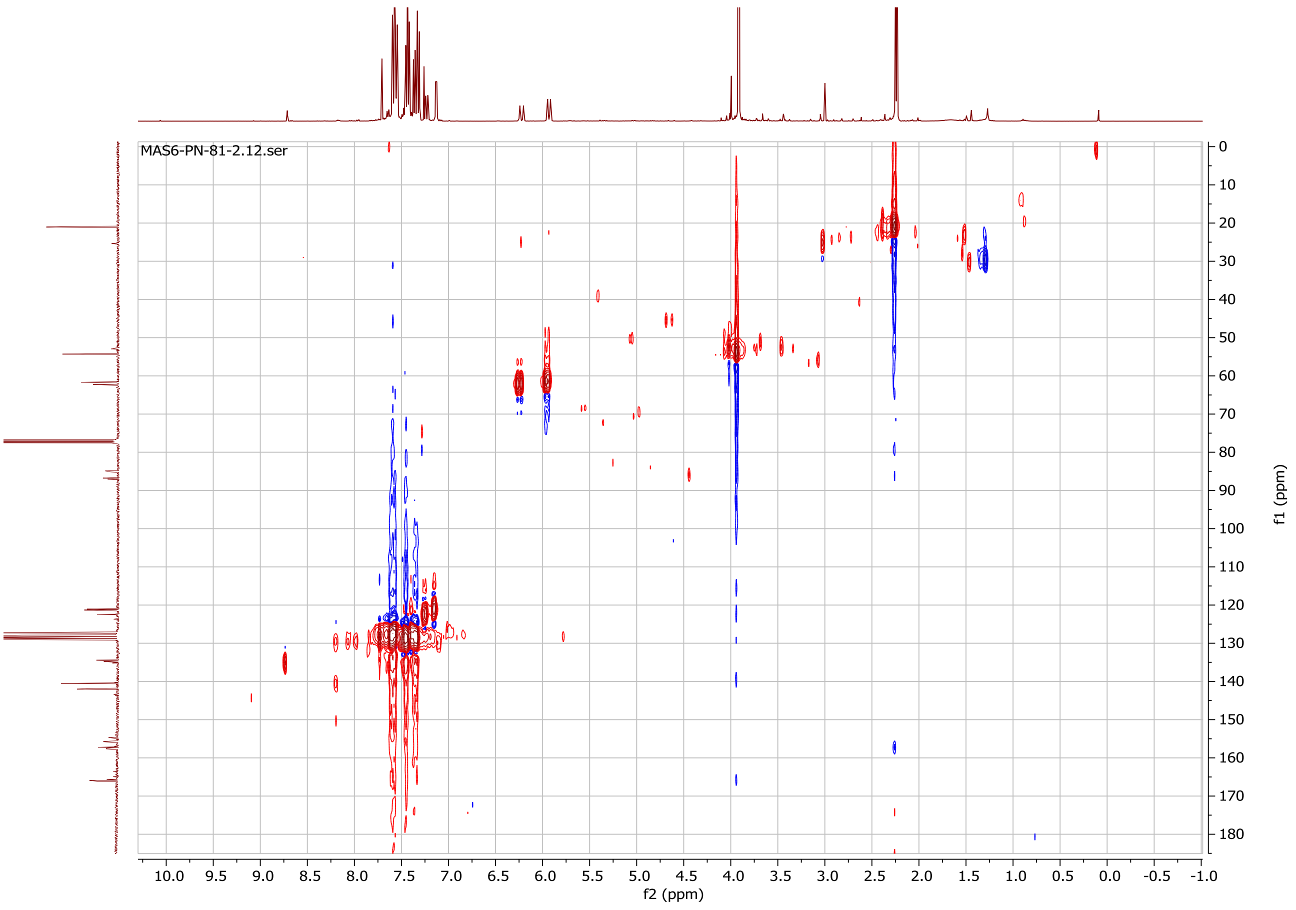

{^{13}C–^{1}H} HMBC spectrum of compound **2b**

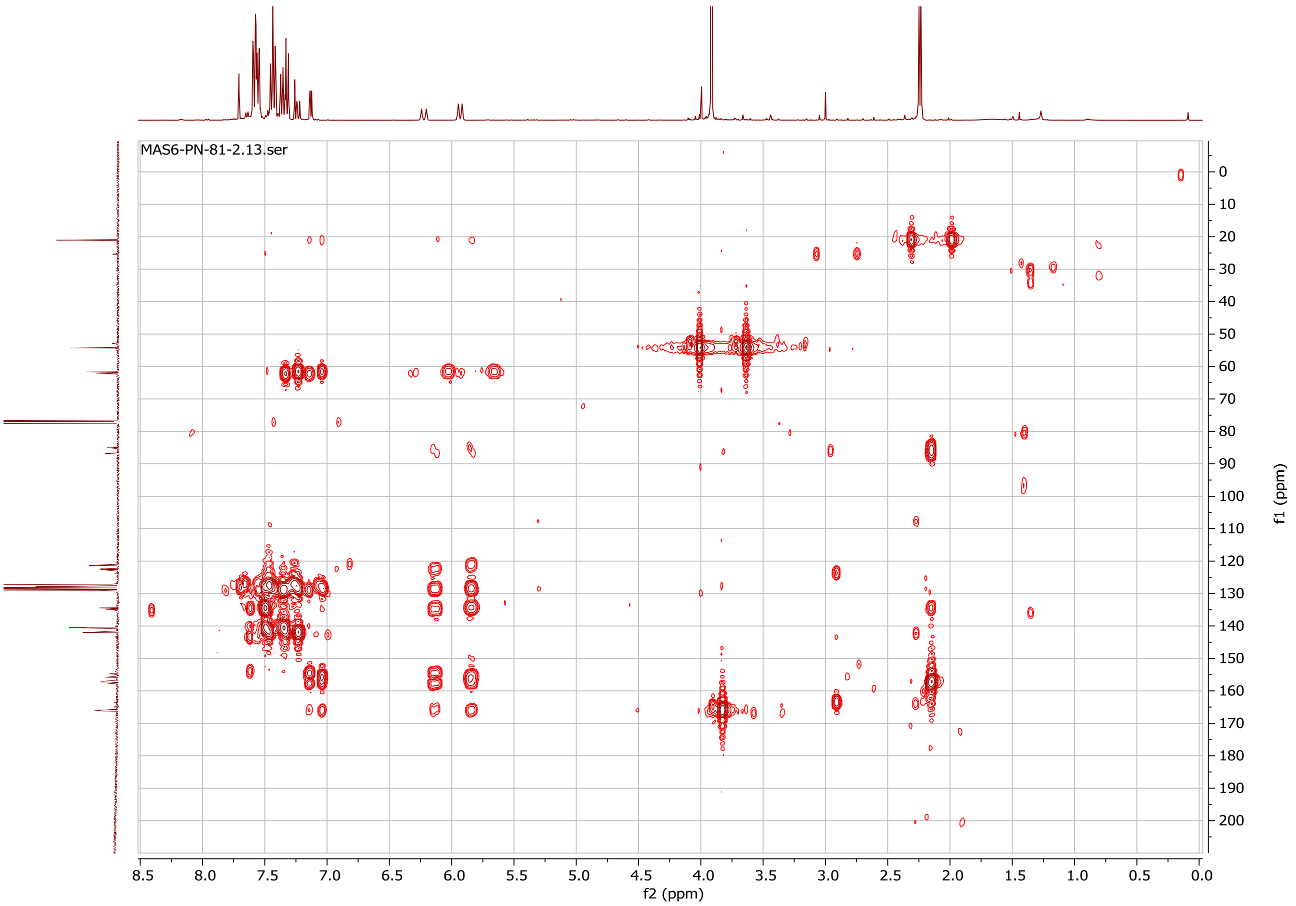

^{1}H NMR spectrum of compound **2c** (+ 25% pyridine **3c**)

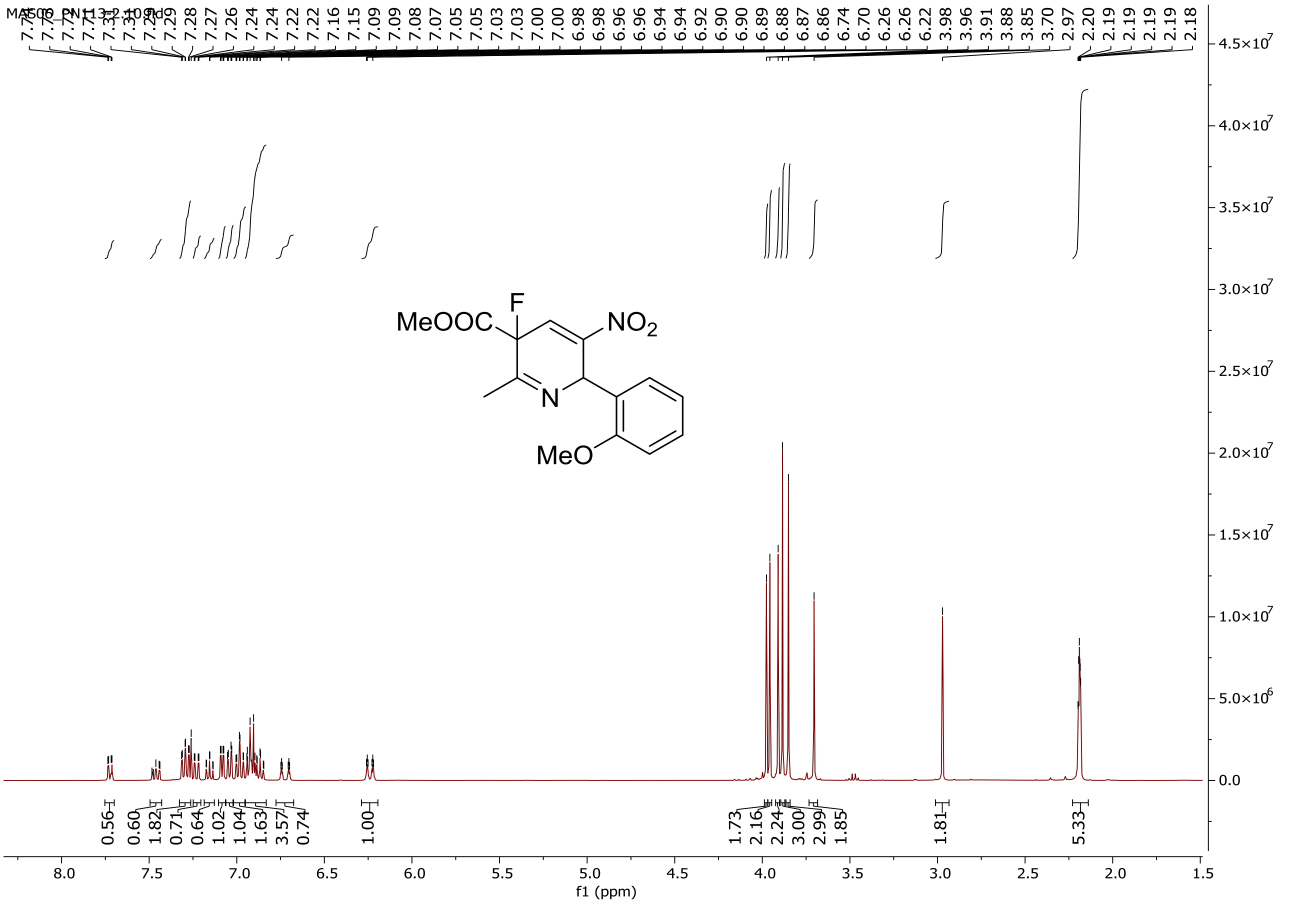

^{19}F NMR spectrum of compound **2c**

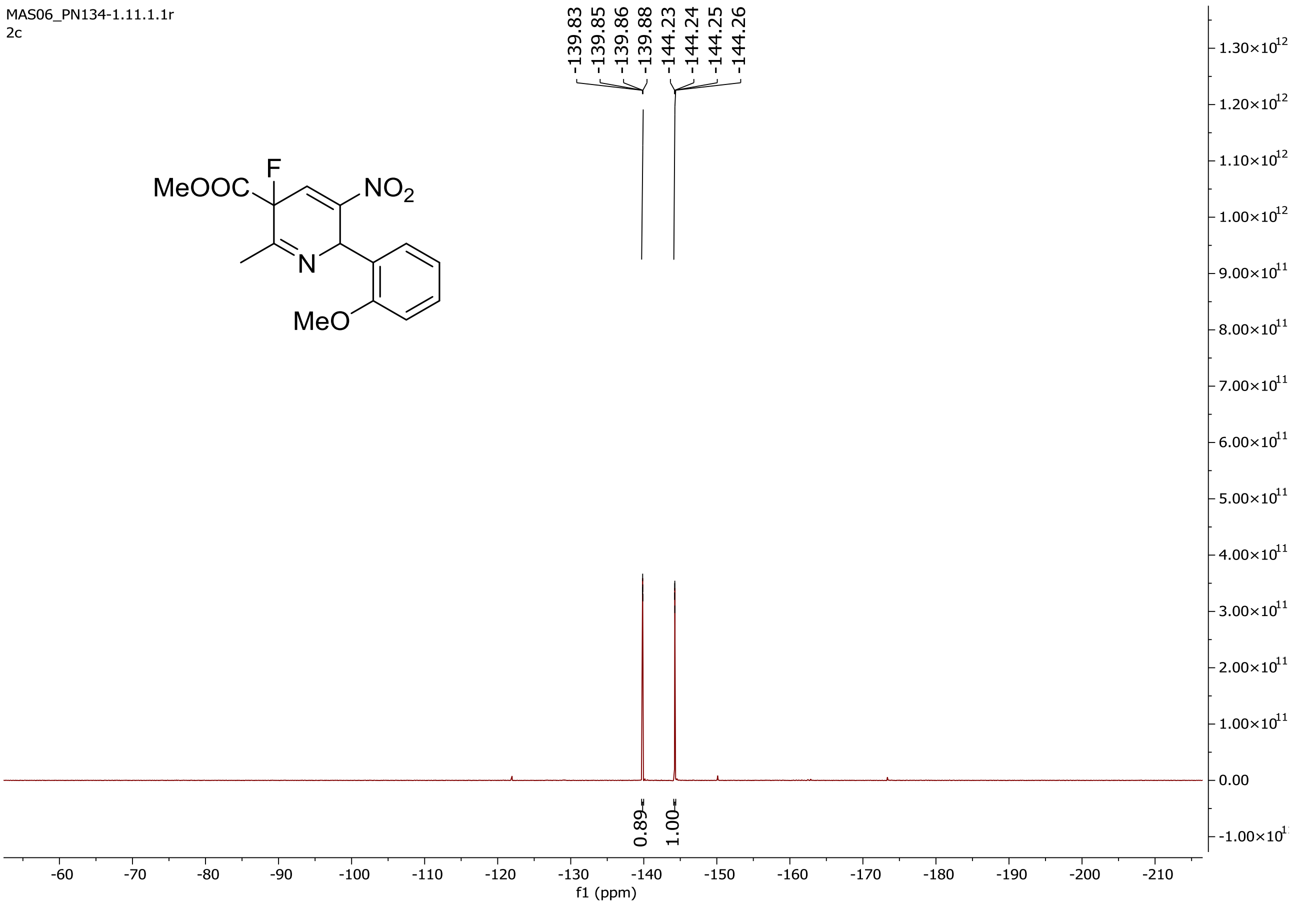

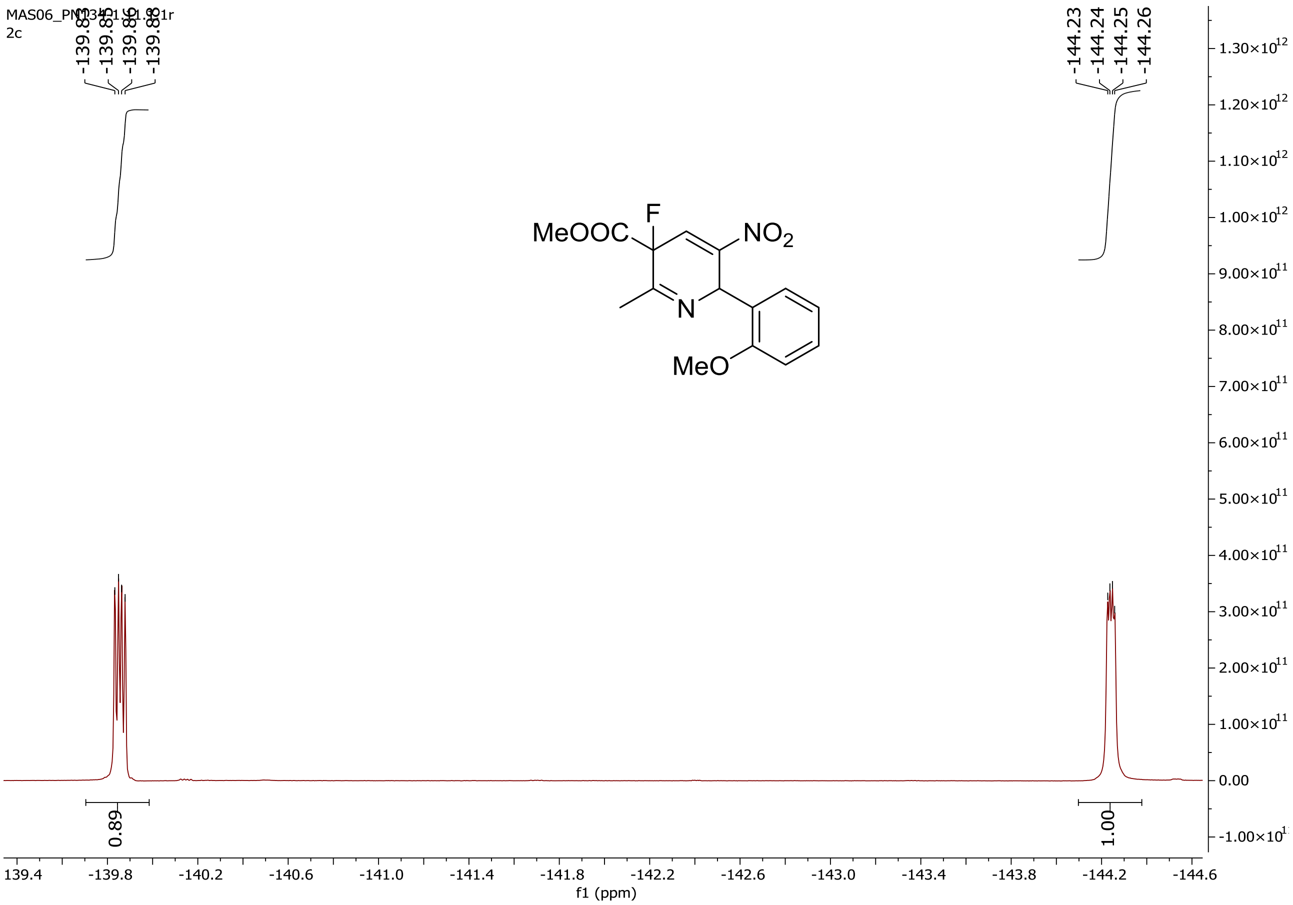

^{19}F NMR spectrum with ^{1}H decoupling of compound **2c**

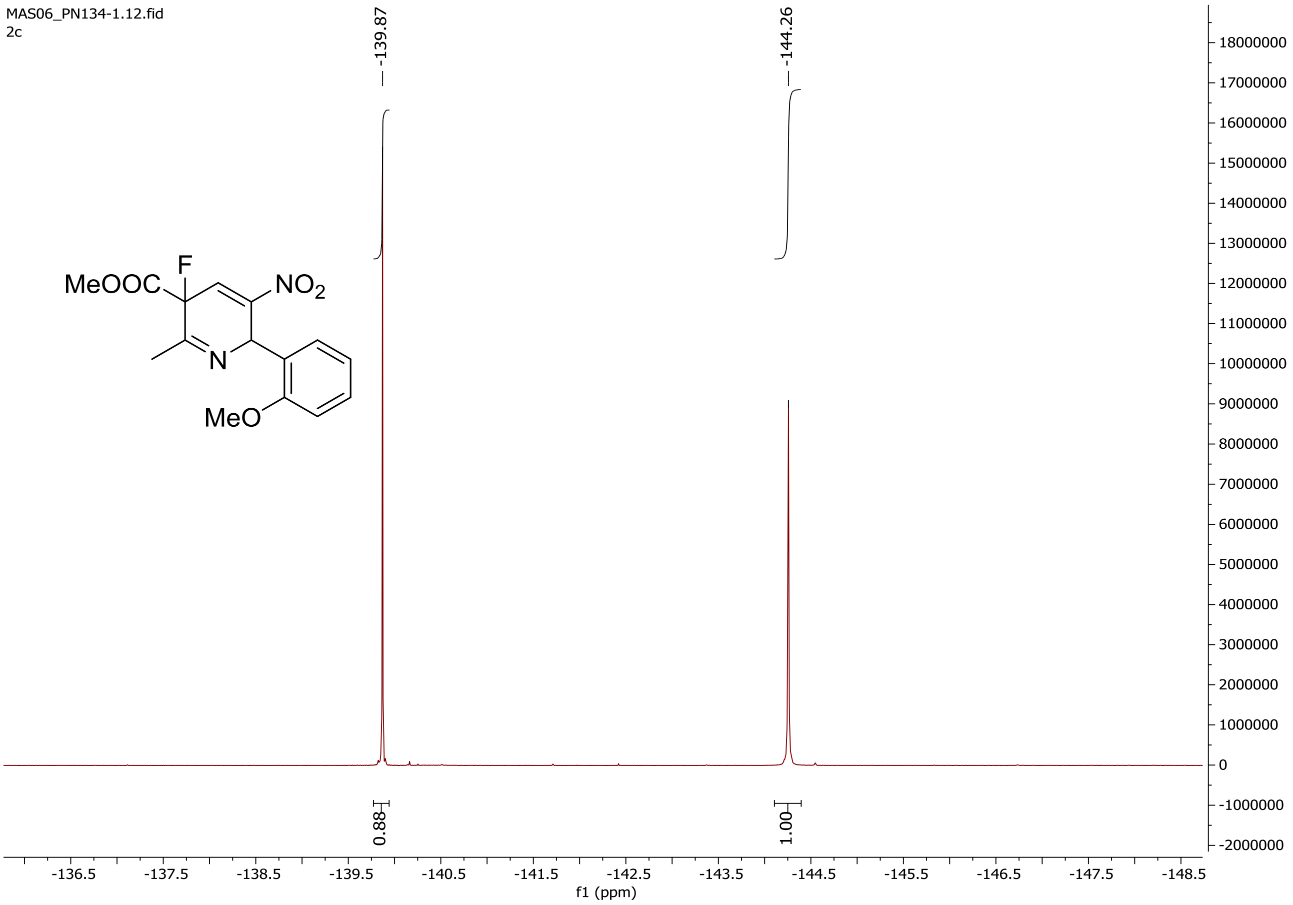

^{13}C NMR spectrum of compound **2c** (+ 25% pyridine **3c**)

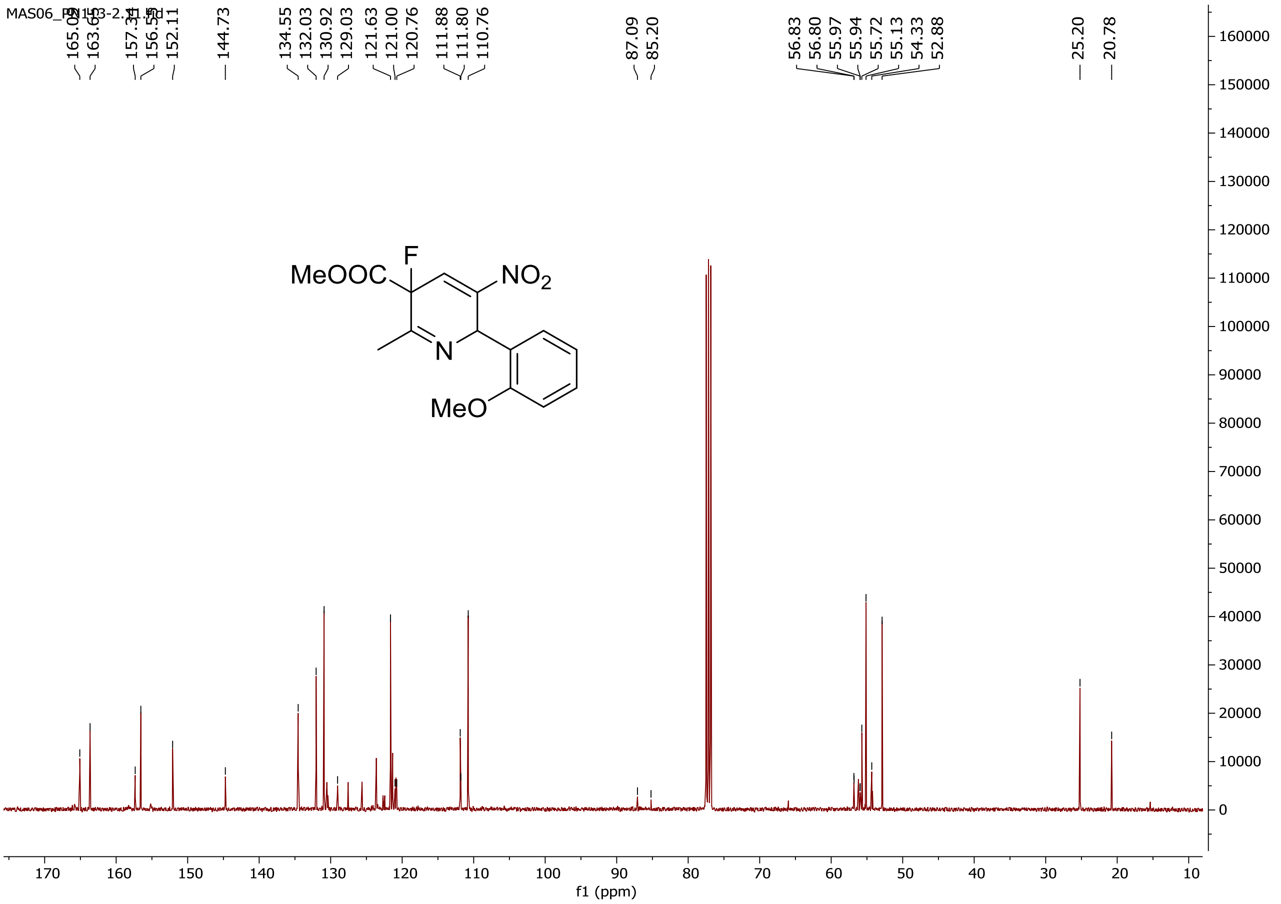

{^{13}C–^{1}H} HSQC spectrum of compound **2c**

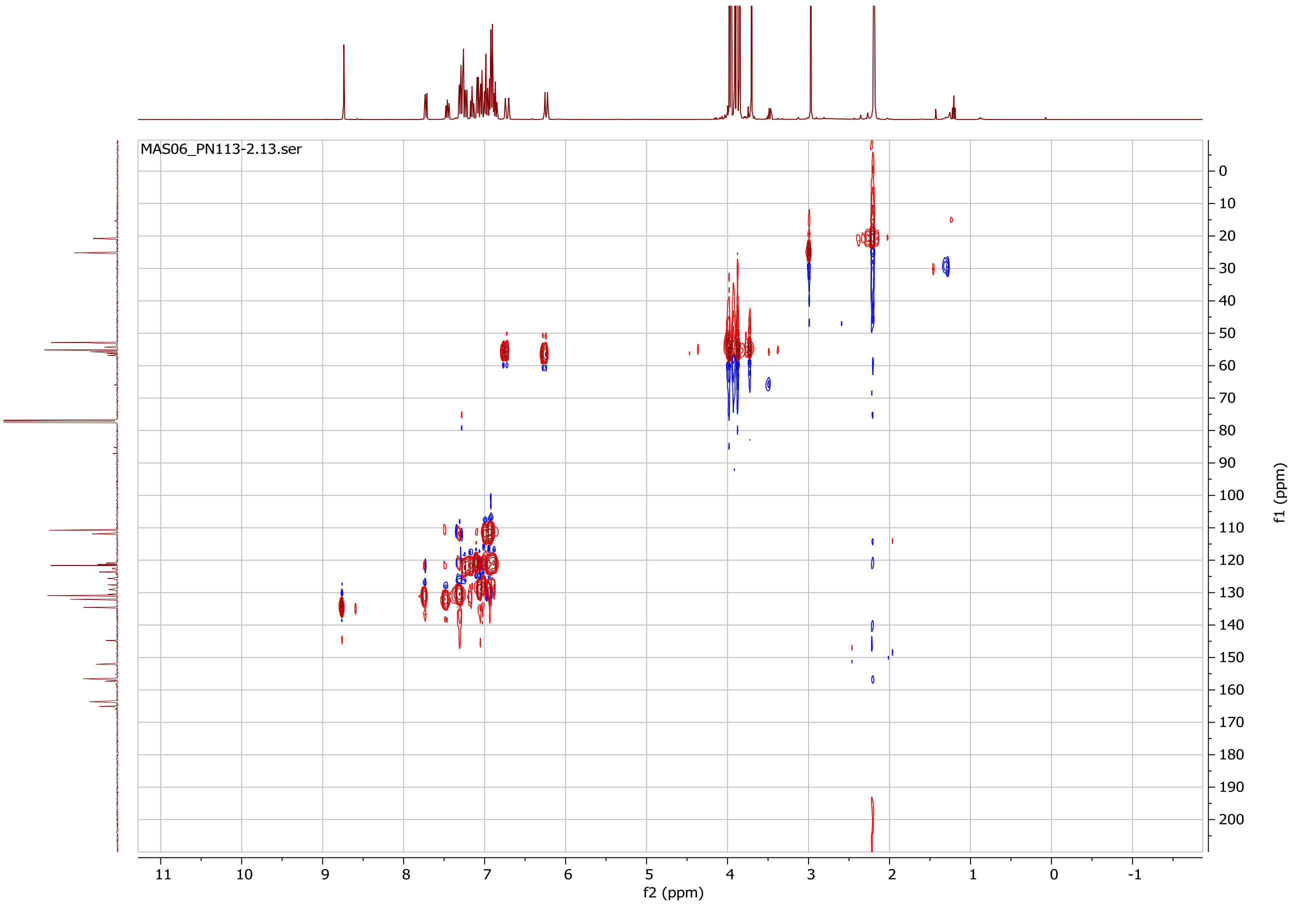

{^{13}C–^{1}H} HMBC spectrum of compound **2c**

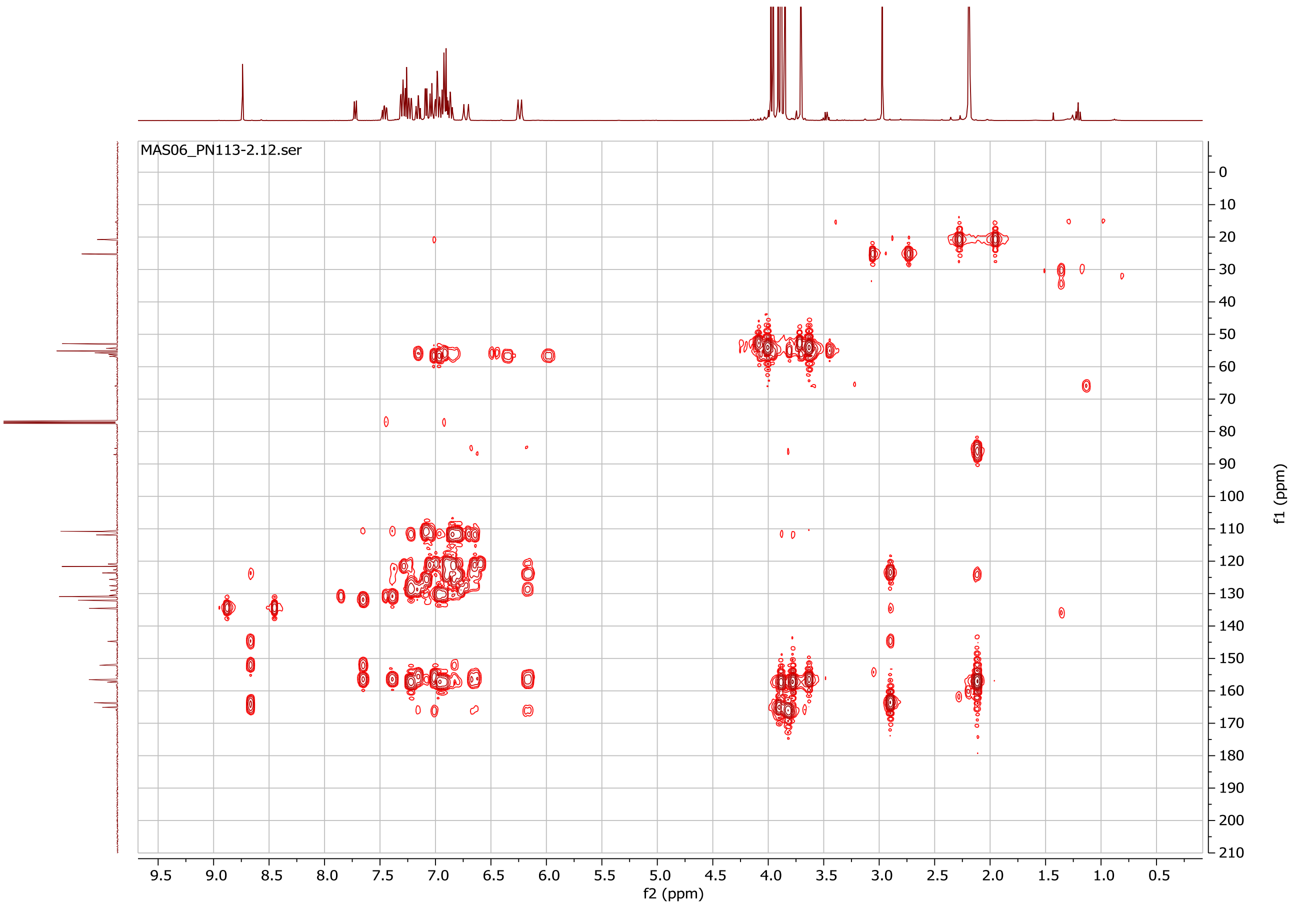

^{1}H NMR spectrum of compound **2d**

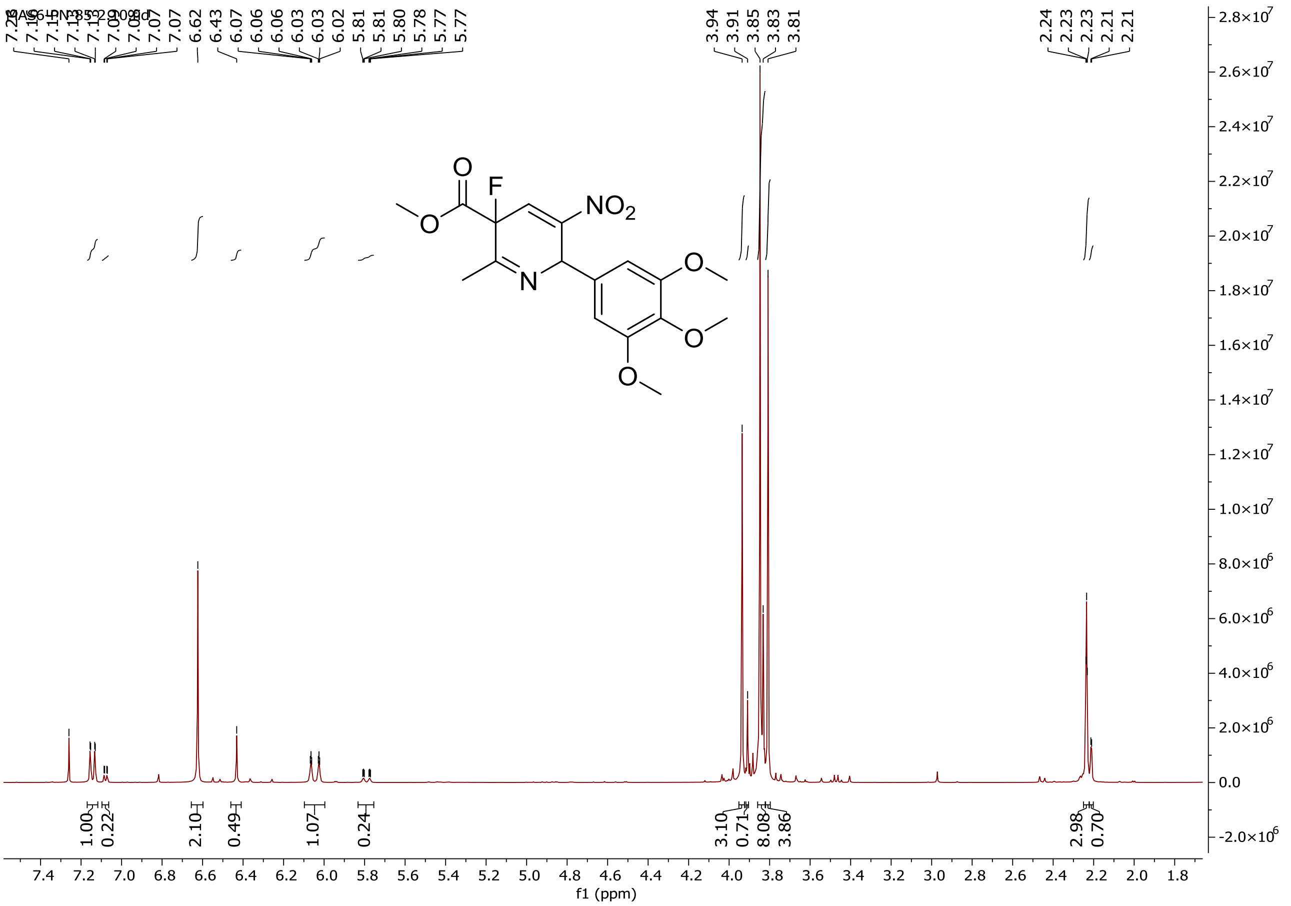

^{19}F NMR spectrum of compound **2d**

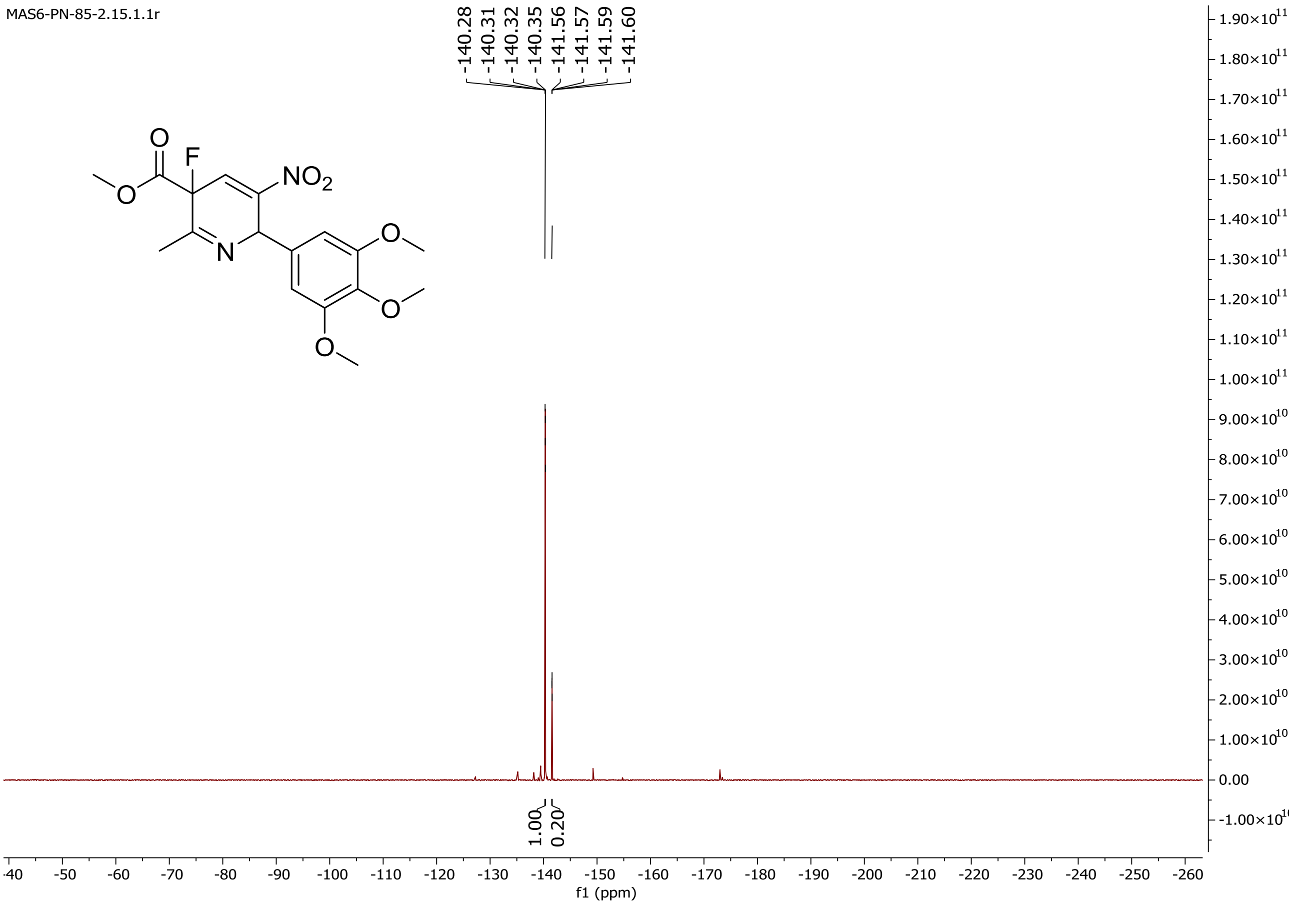

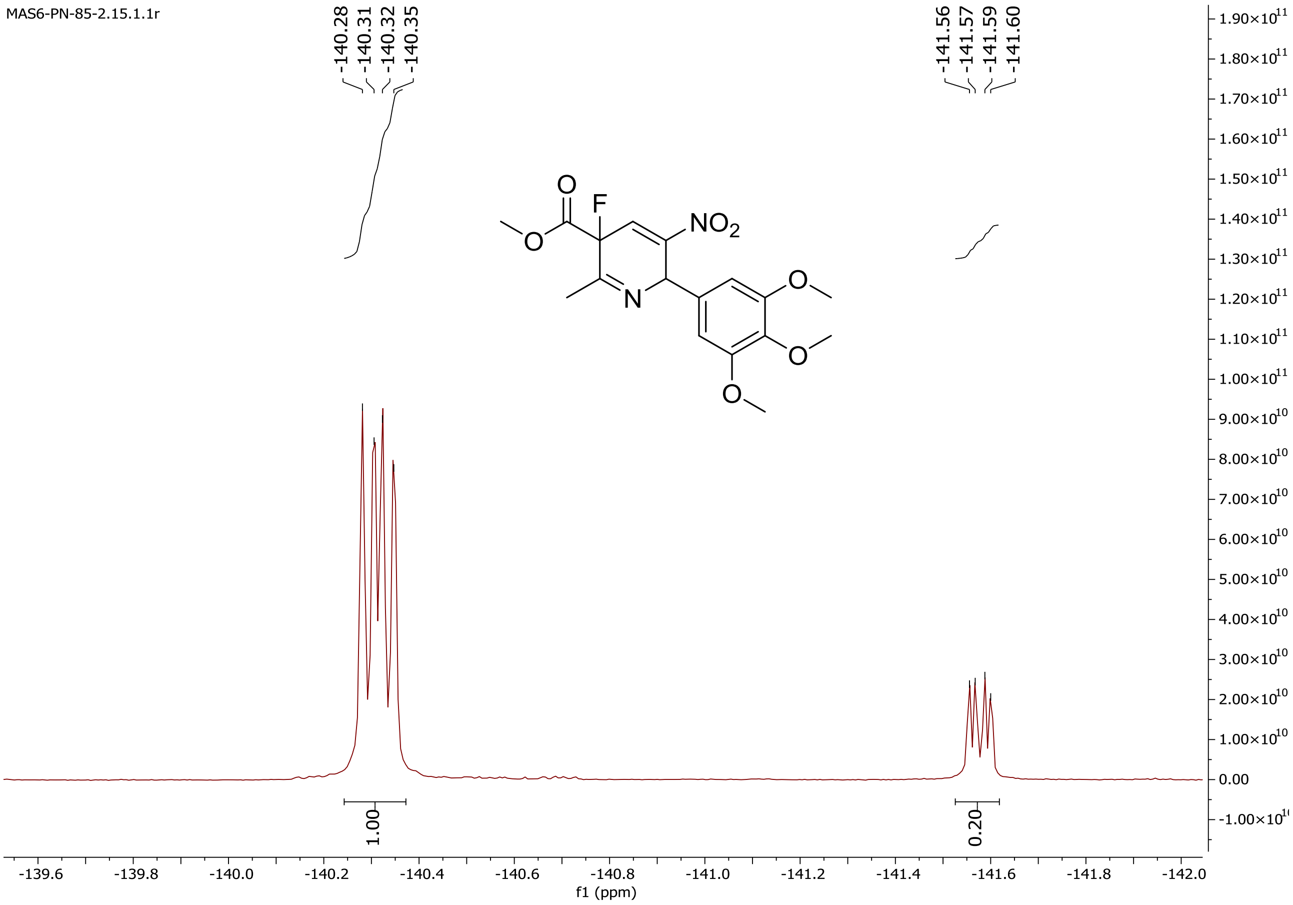

^{13}C NMR spectrum of compound **2d**

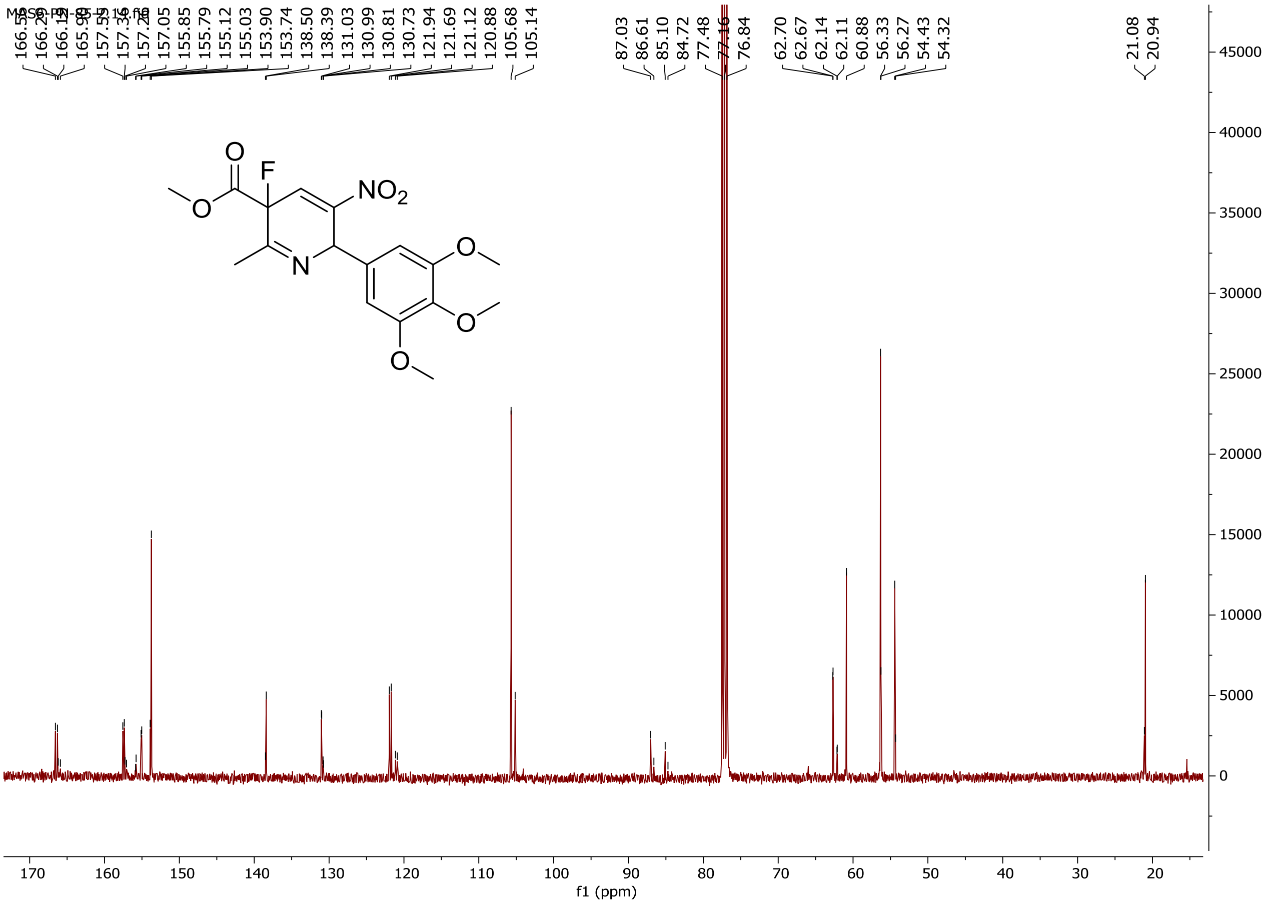

{^{1}H–^{1}H}COSY spectrum of compound **2d**

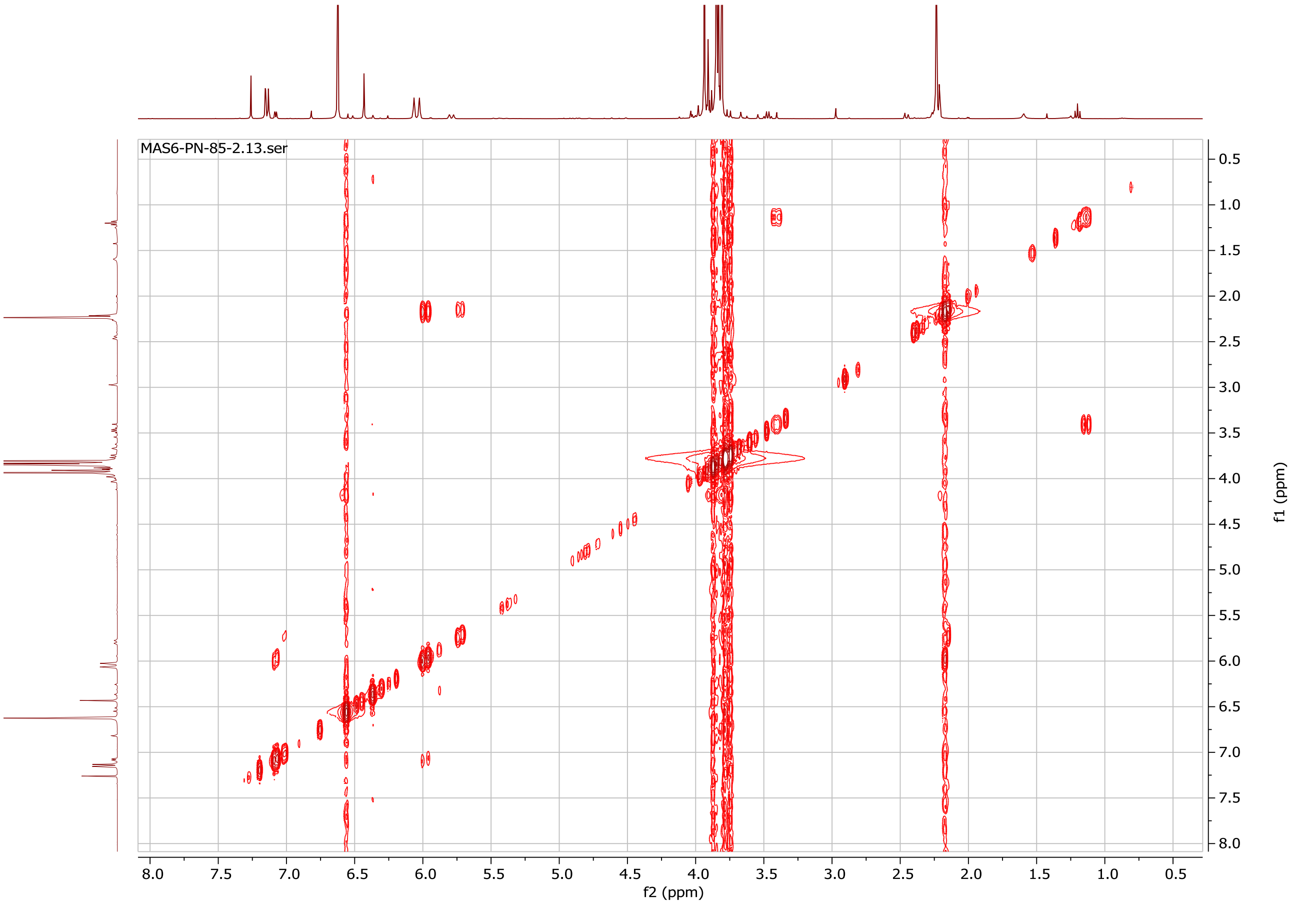

{^{13}C–^{1}H} HSQC spectrum of compound **2d**

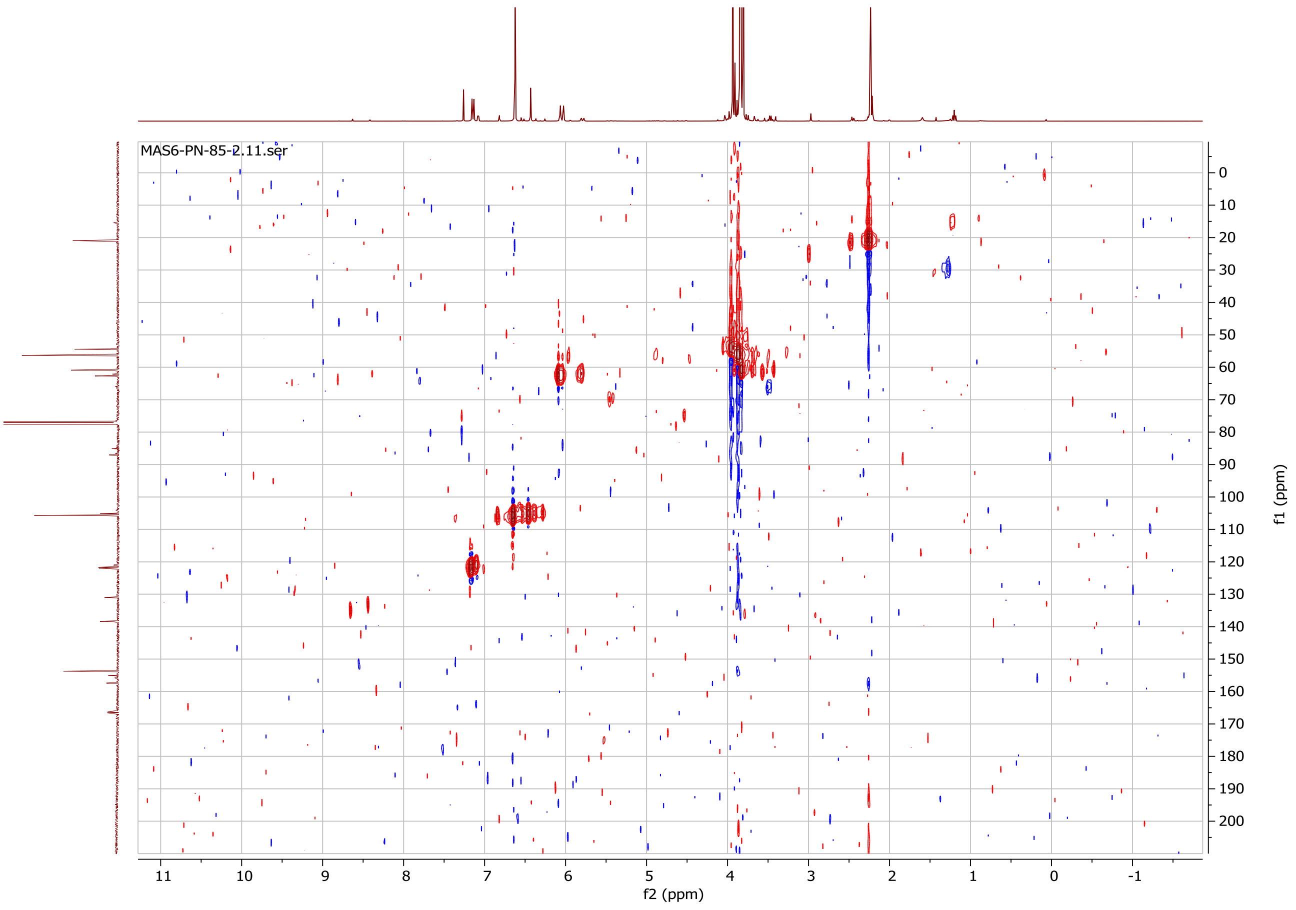

{^{13}C–^{1}H}HMBC spectrum of compound **2d**

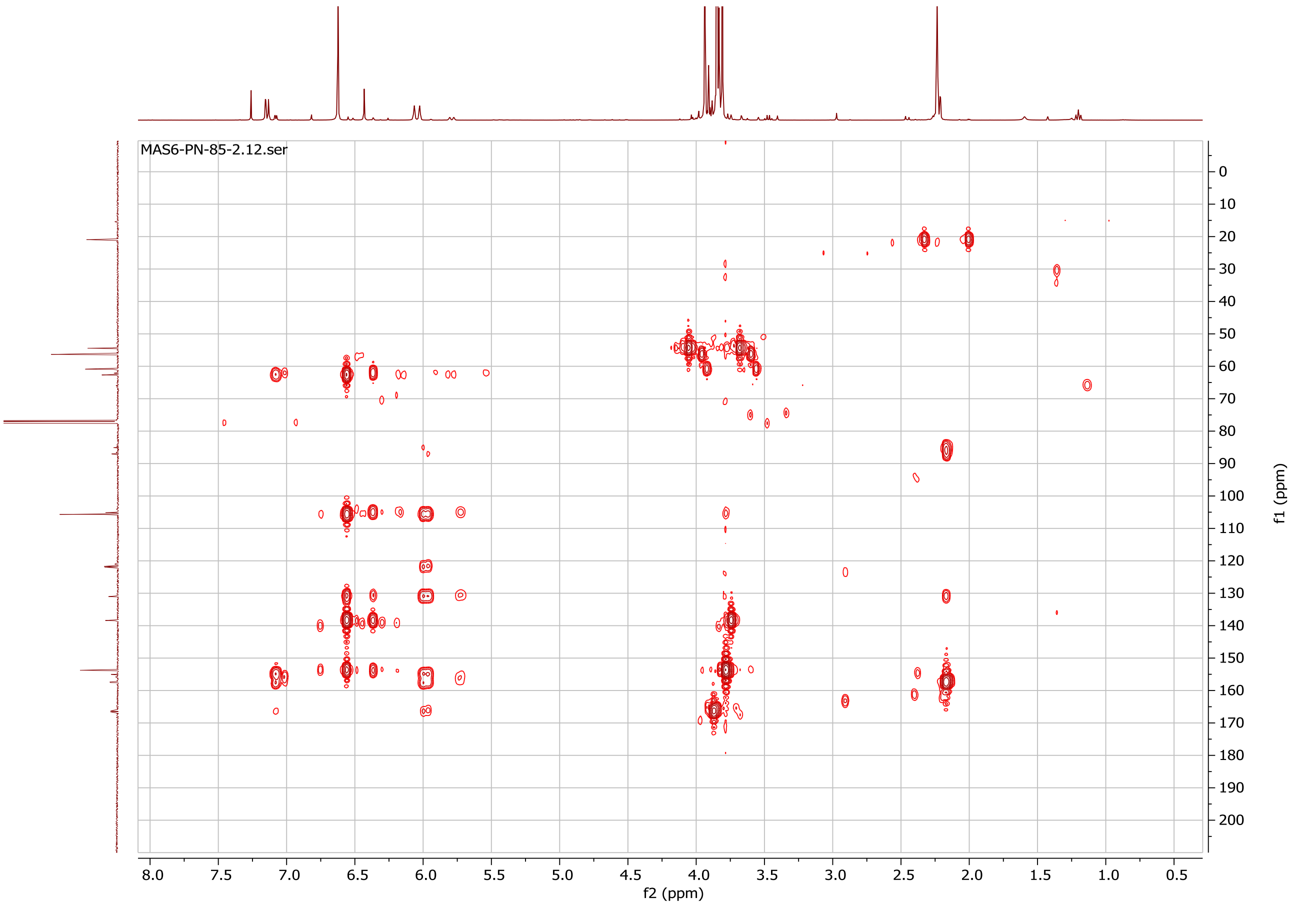

^{1}H NMR spectrum of compound **2e**

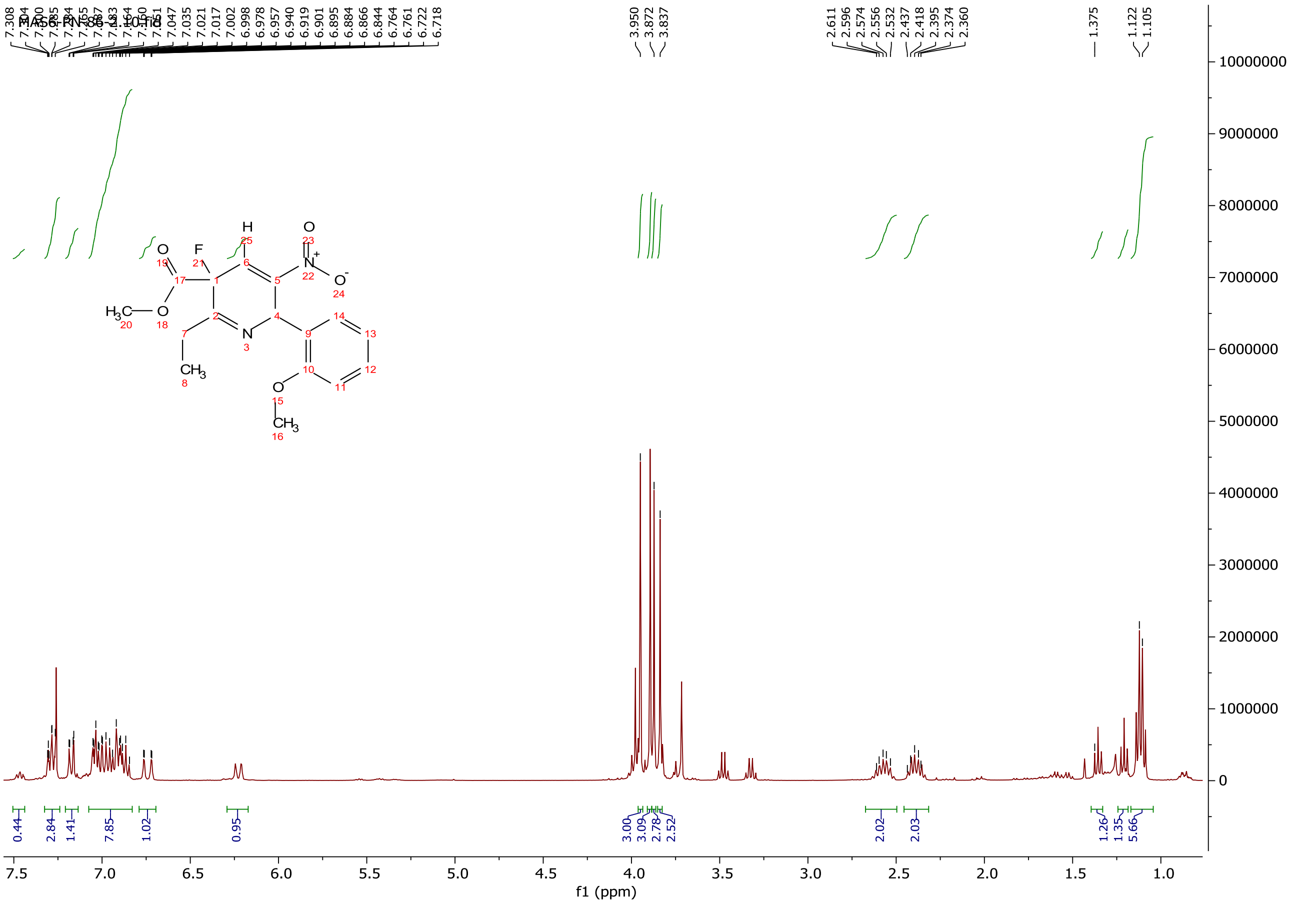

^{19}F NMR spectrum of compound **2e**

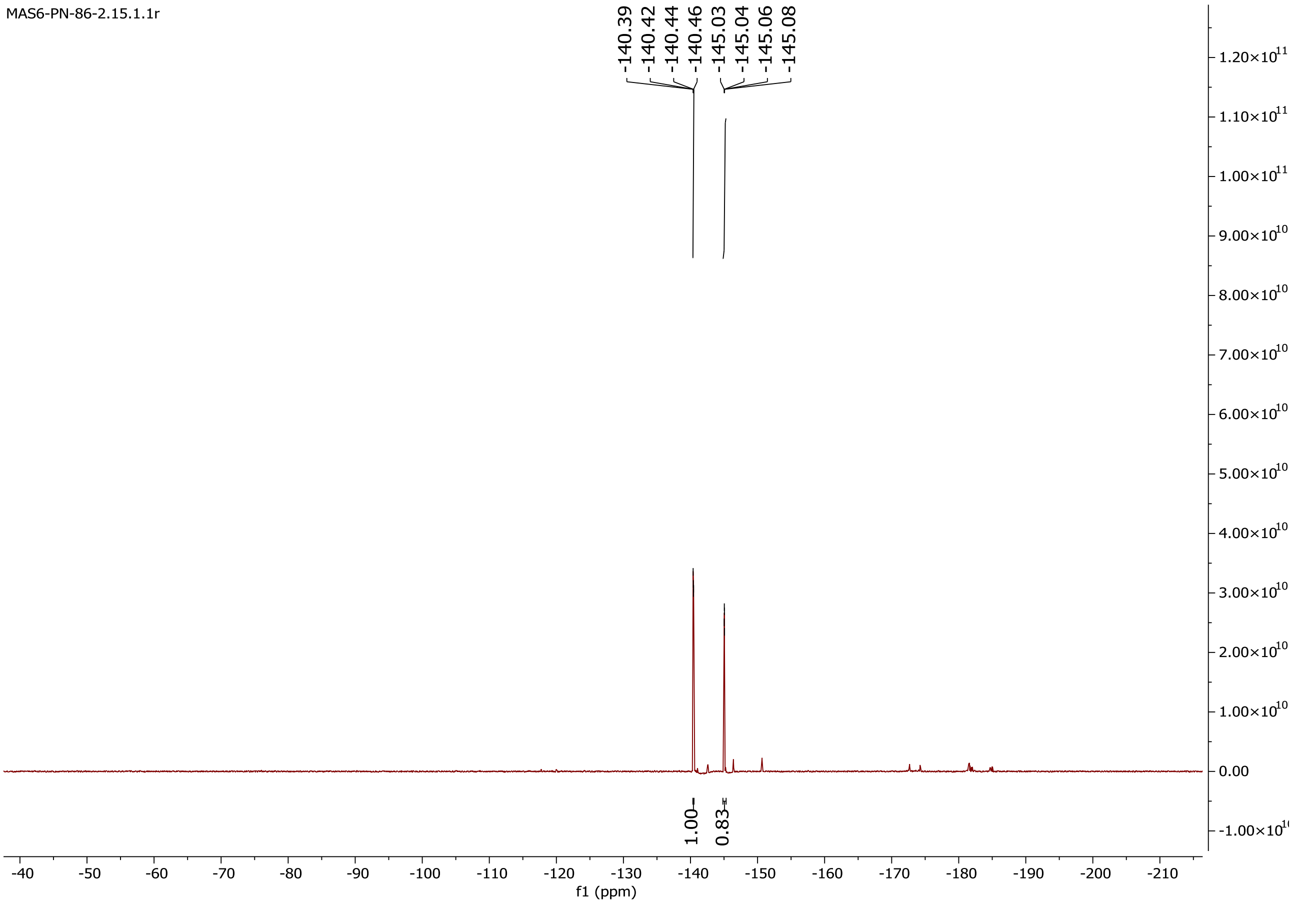

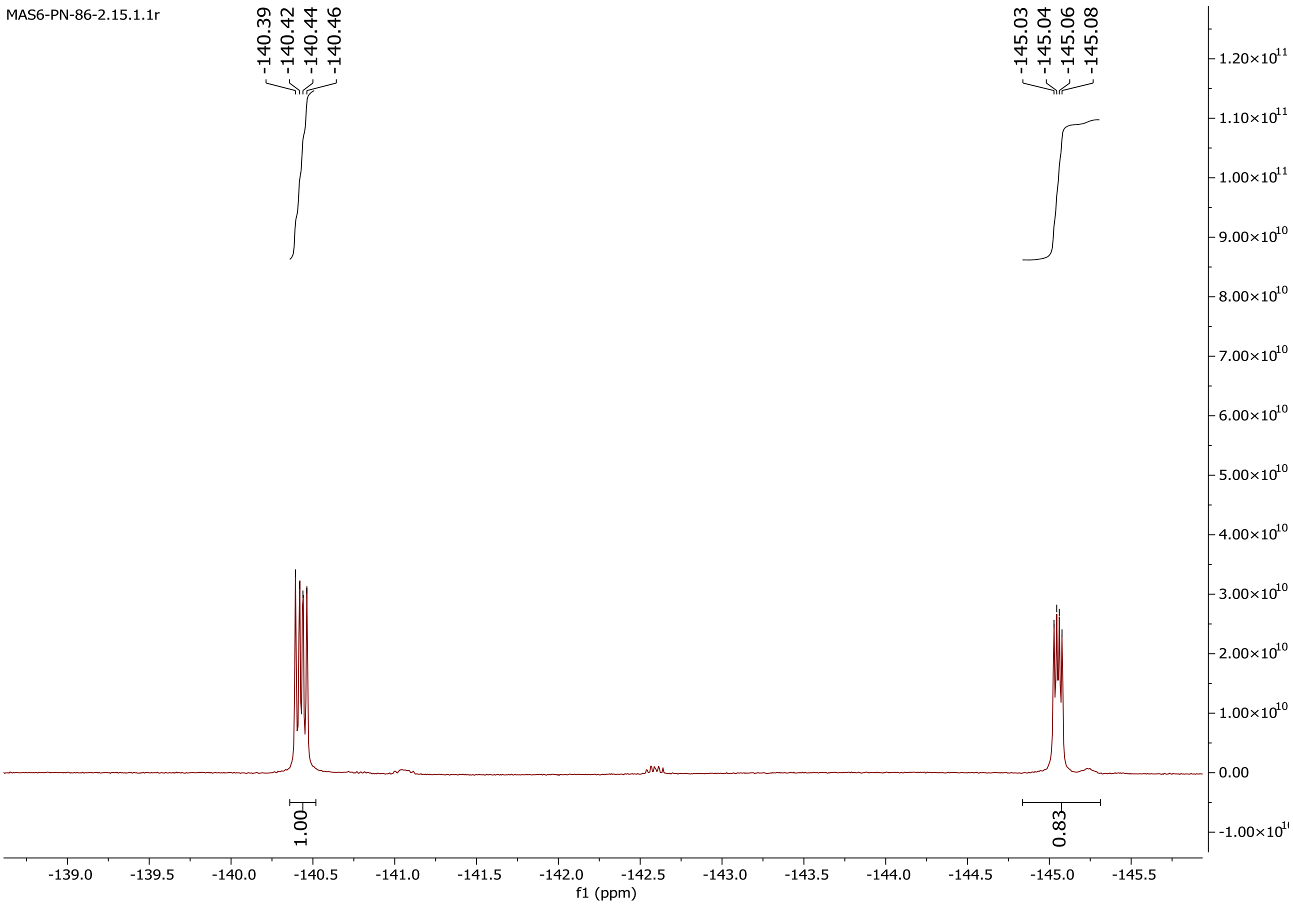

^{13}C NMR spectrum of compound **2e**

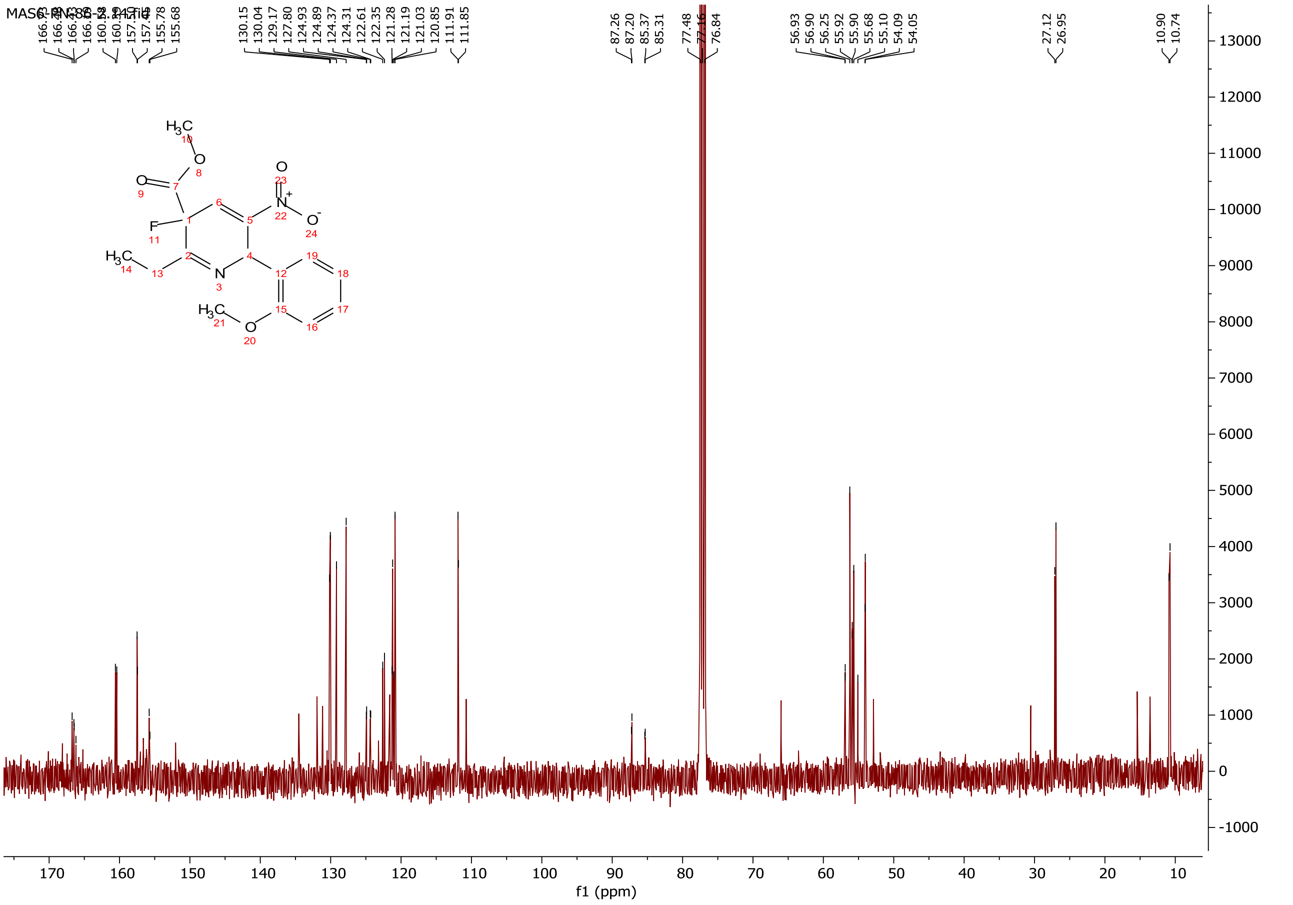

^{13}C NMR spectrum of compound **2e**

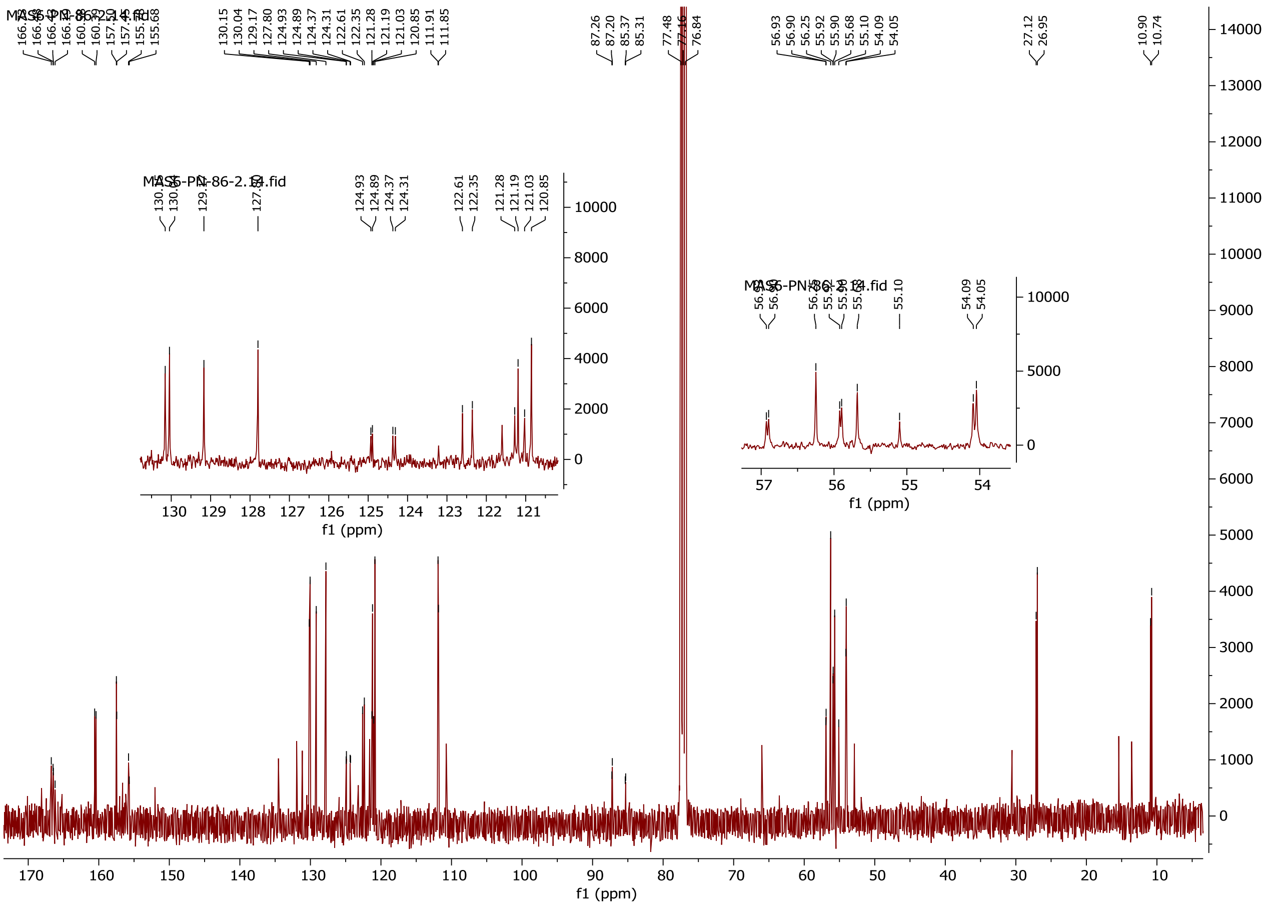

{^{1}H–^{1}H} COSY spectrum of compound **2e**

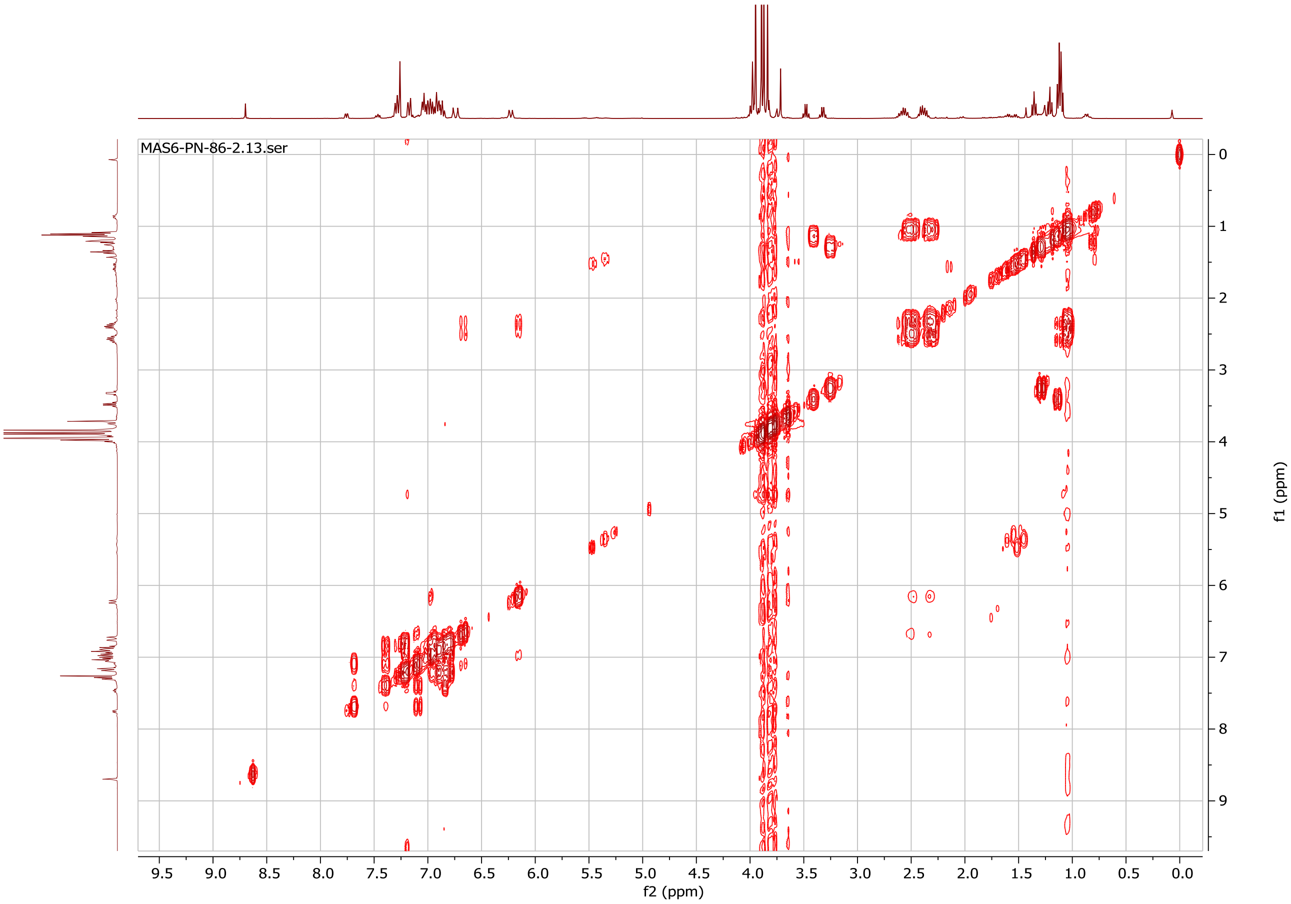

{^{13}C–^{1}H} HSQC spectrum of compound **2e**

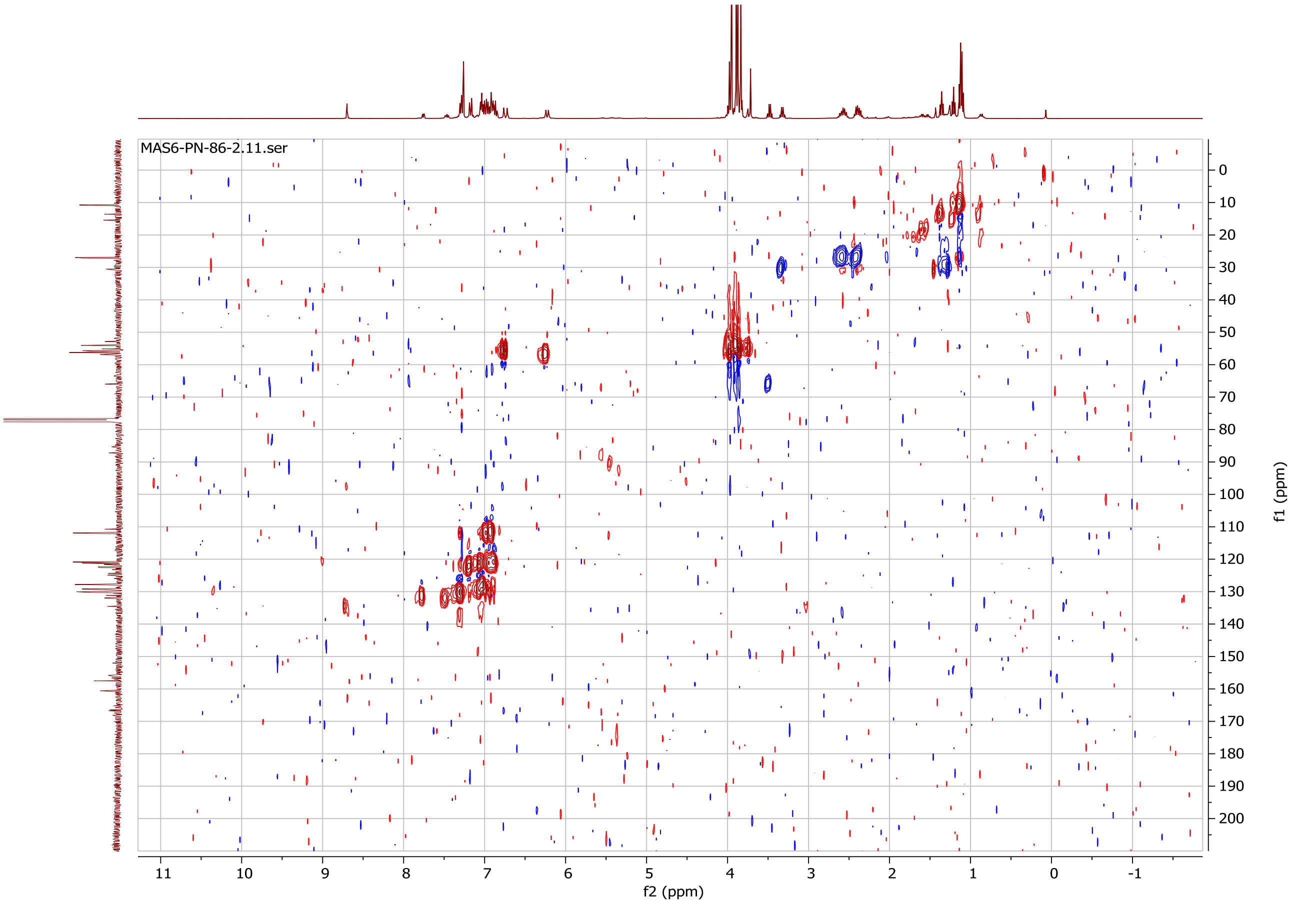

{^{13}C–^{1}H} HMBC spectrum of compound **2e**

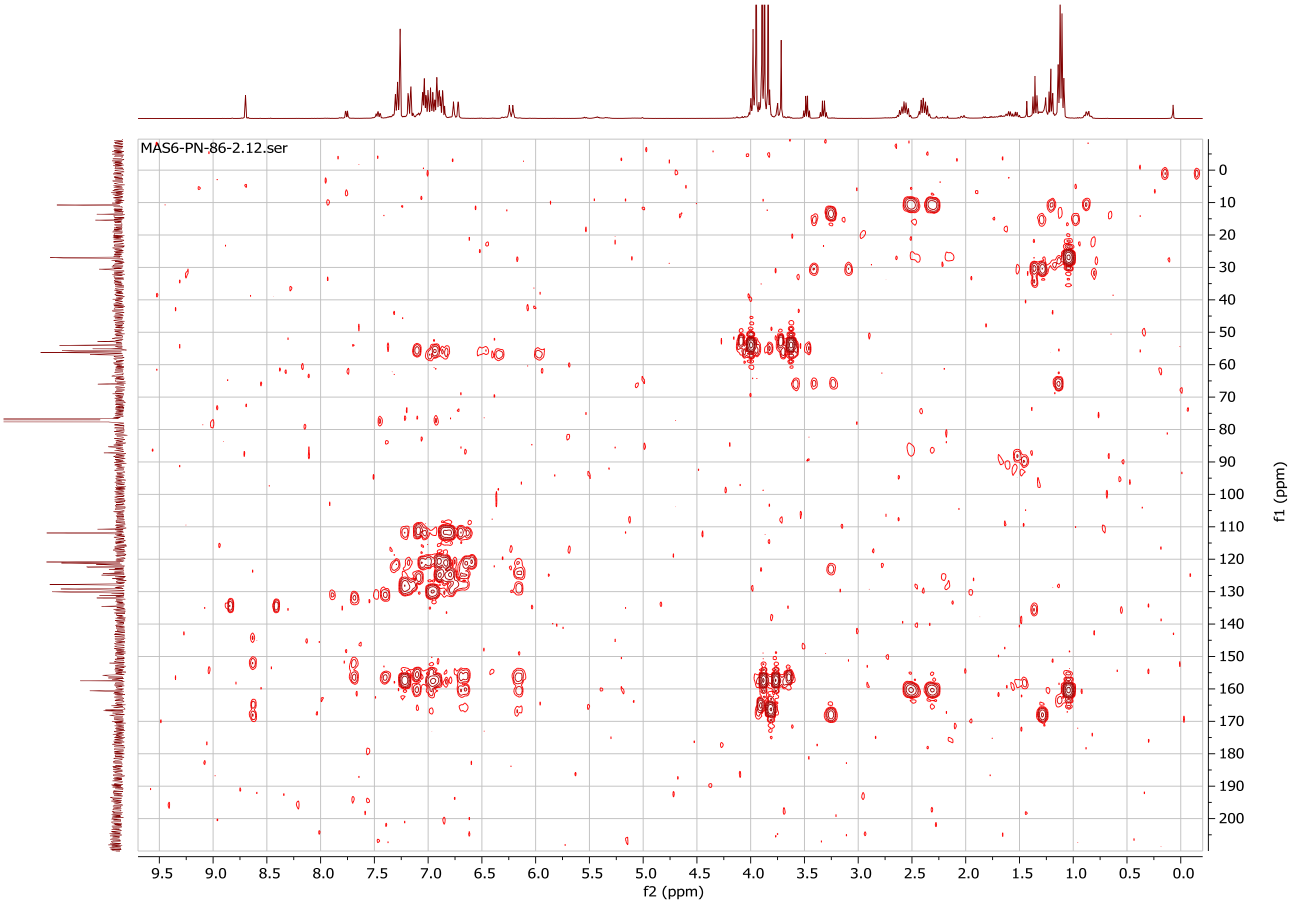

^{1}H NMR spectrum of compound **2f**

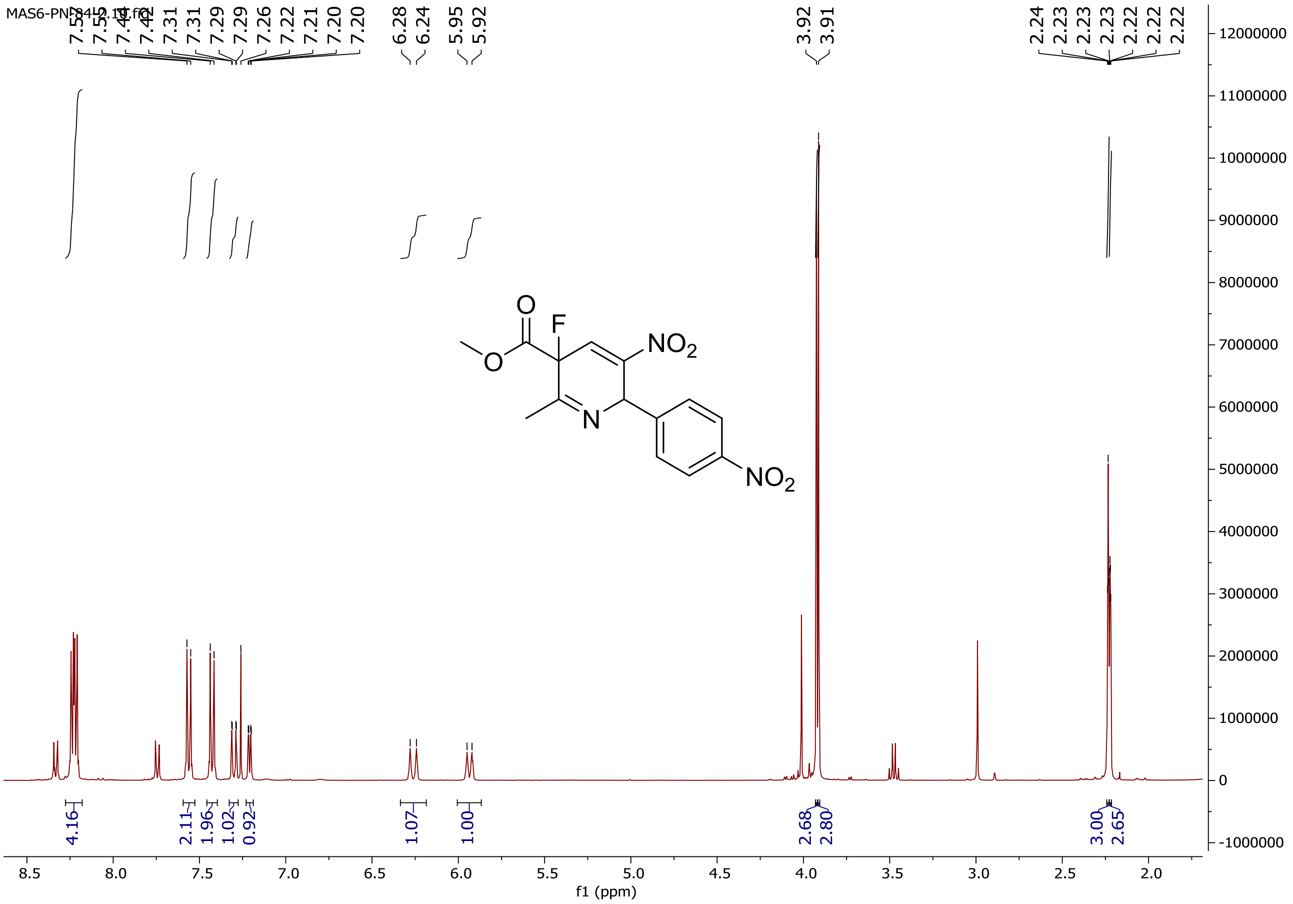

^{19}F NMR spectrum of compound **2f**

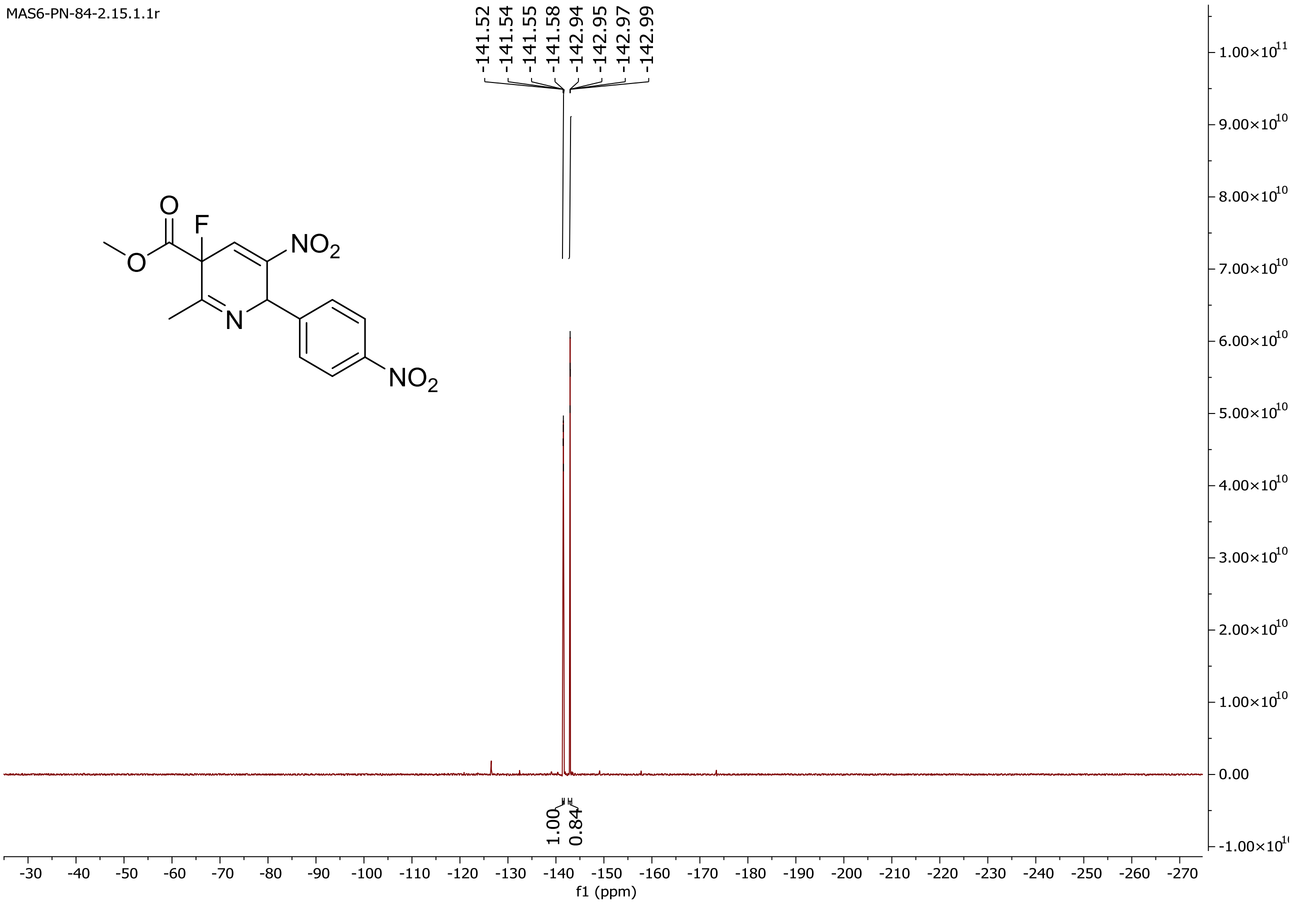

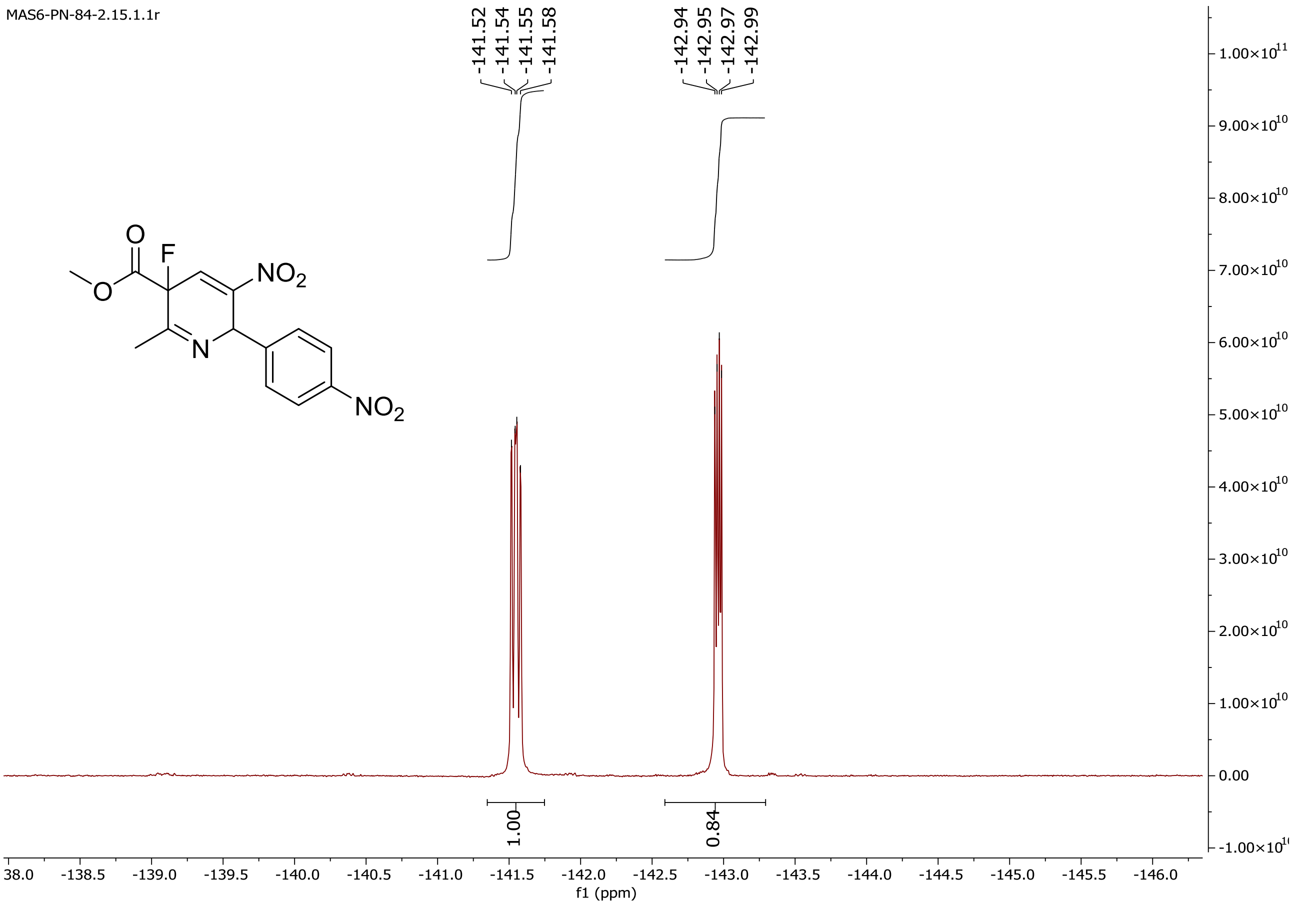

^{13}C NMR spectrum of compound **2f**

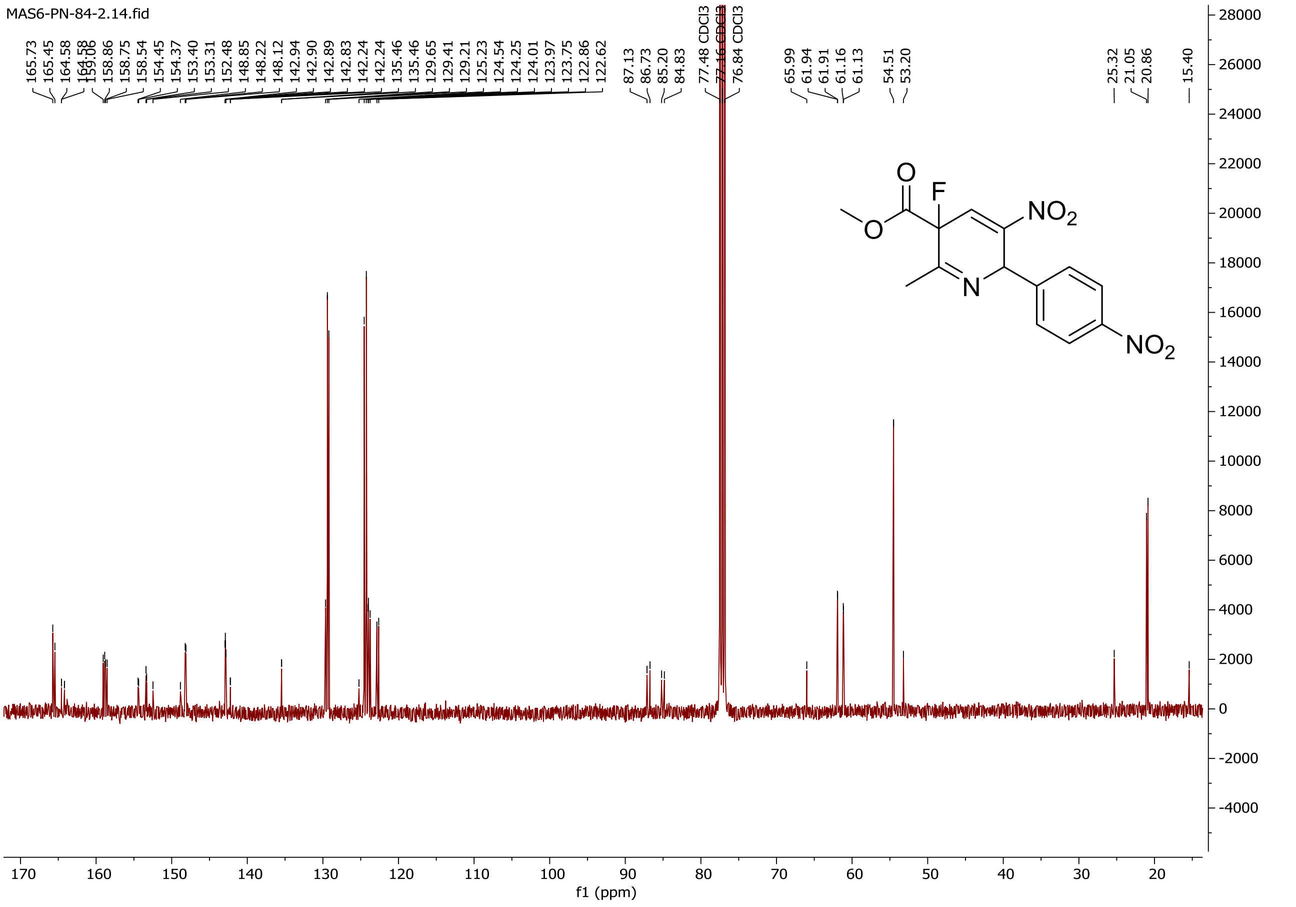

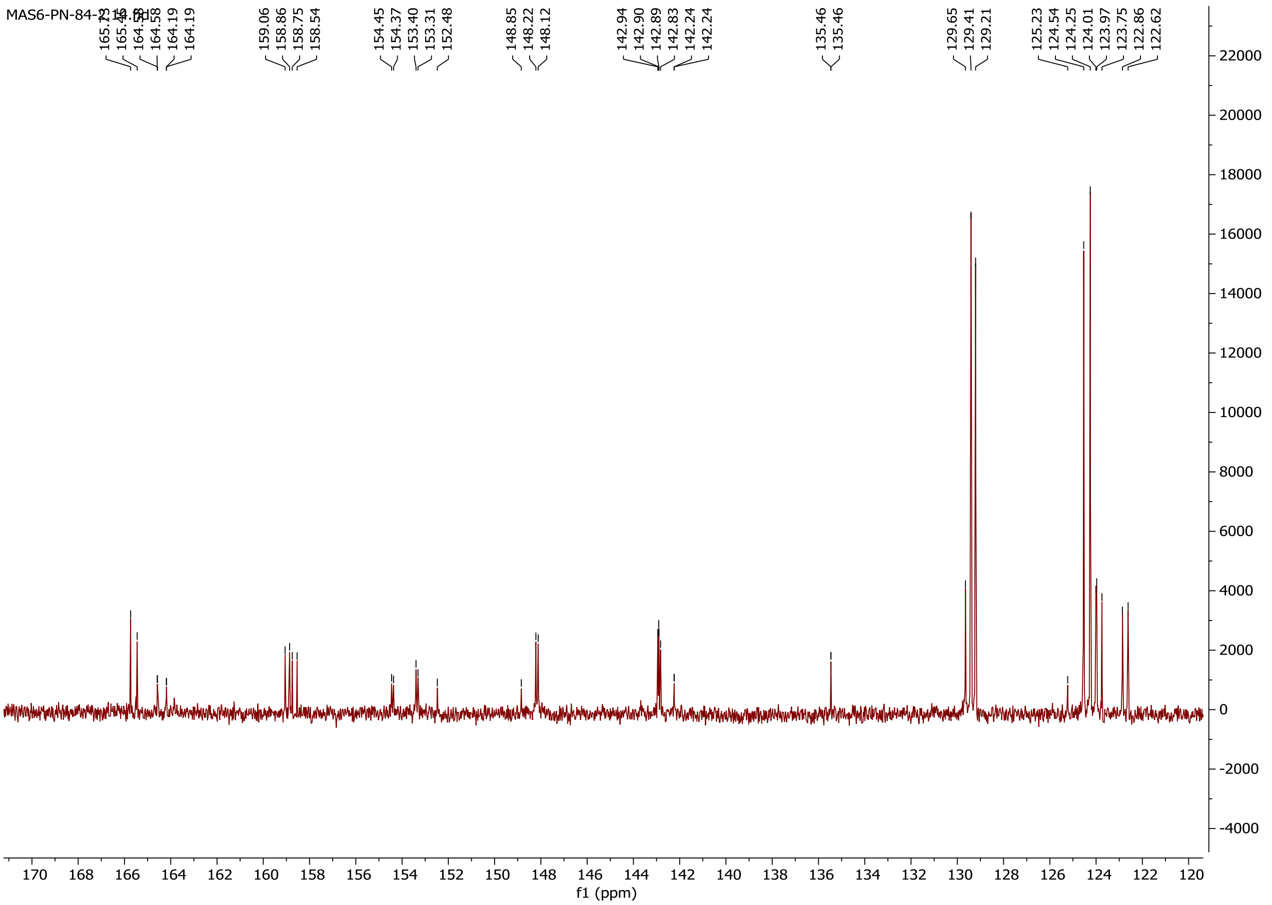

{^{1}H–^{1}H} COSY spectrum of compound **2f**

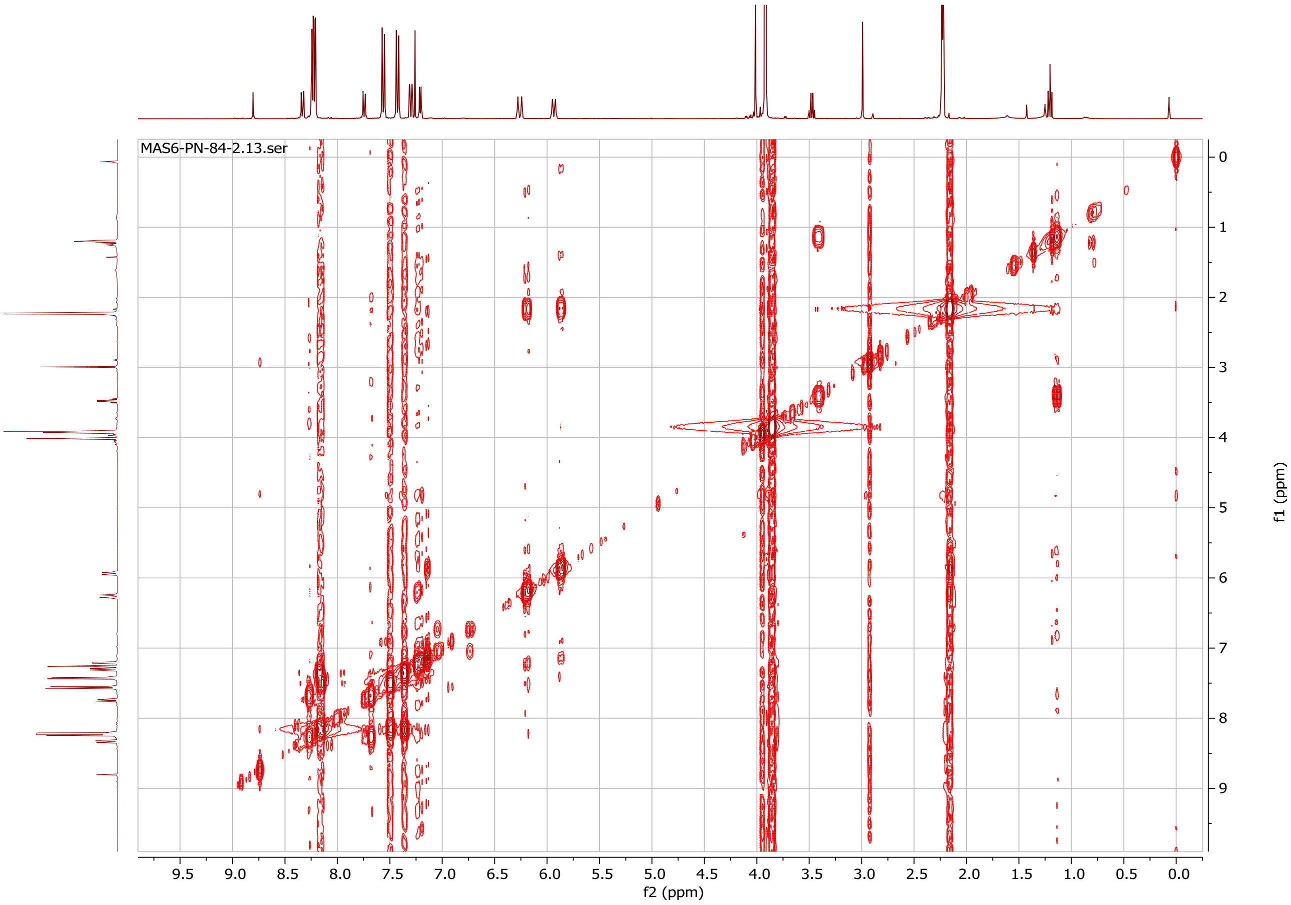

{^{13}C–^{1}H} HSQC spectrum of compound **2f**

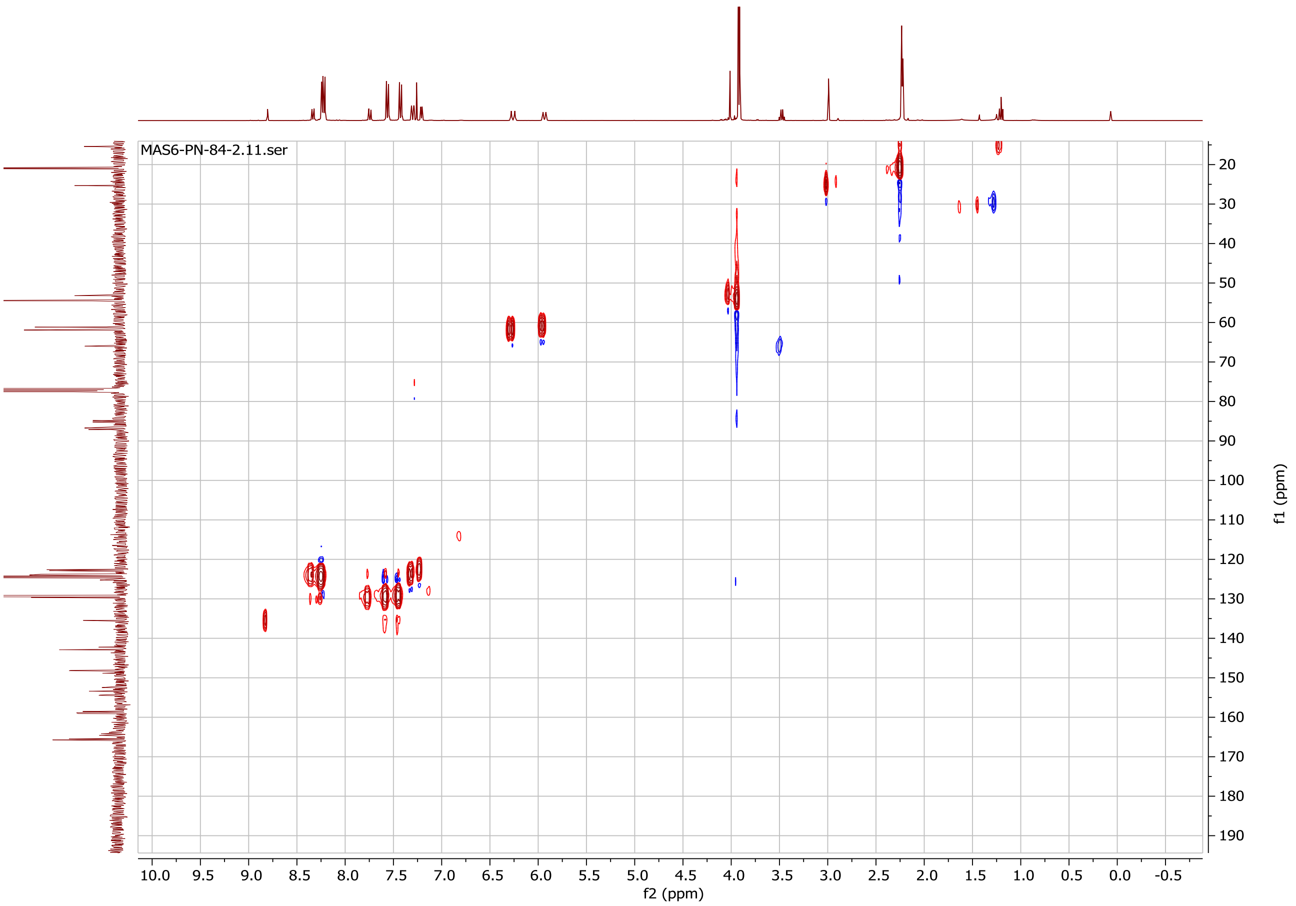

^{1}H NMR spectrum of compound **2g**

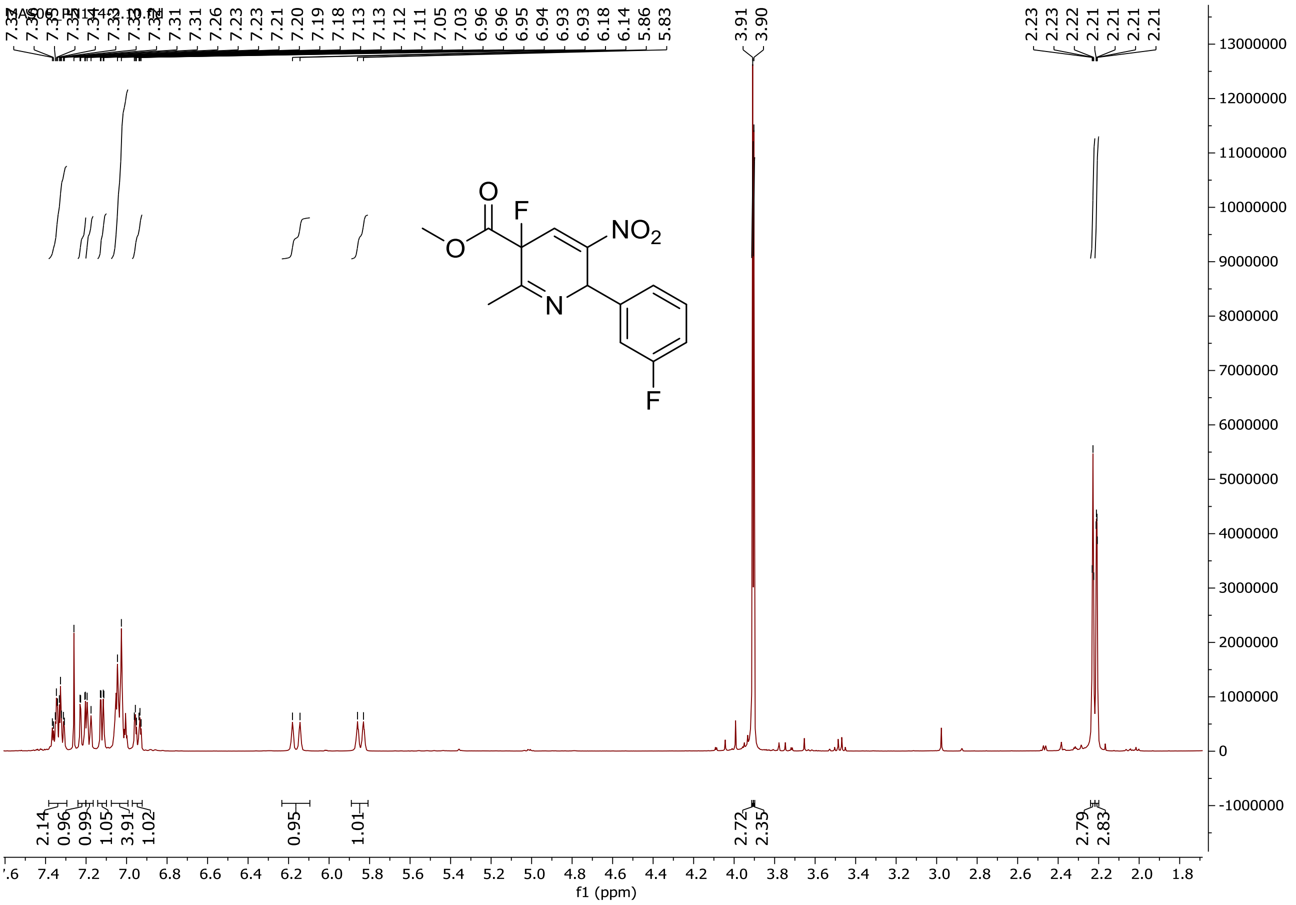

^{19}F NMR spectrum of compound **2g**

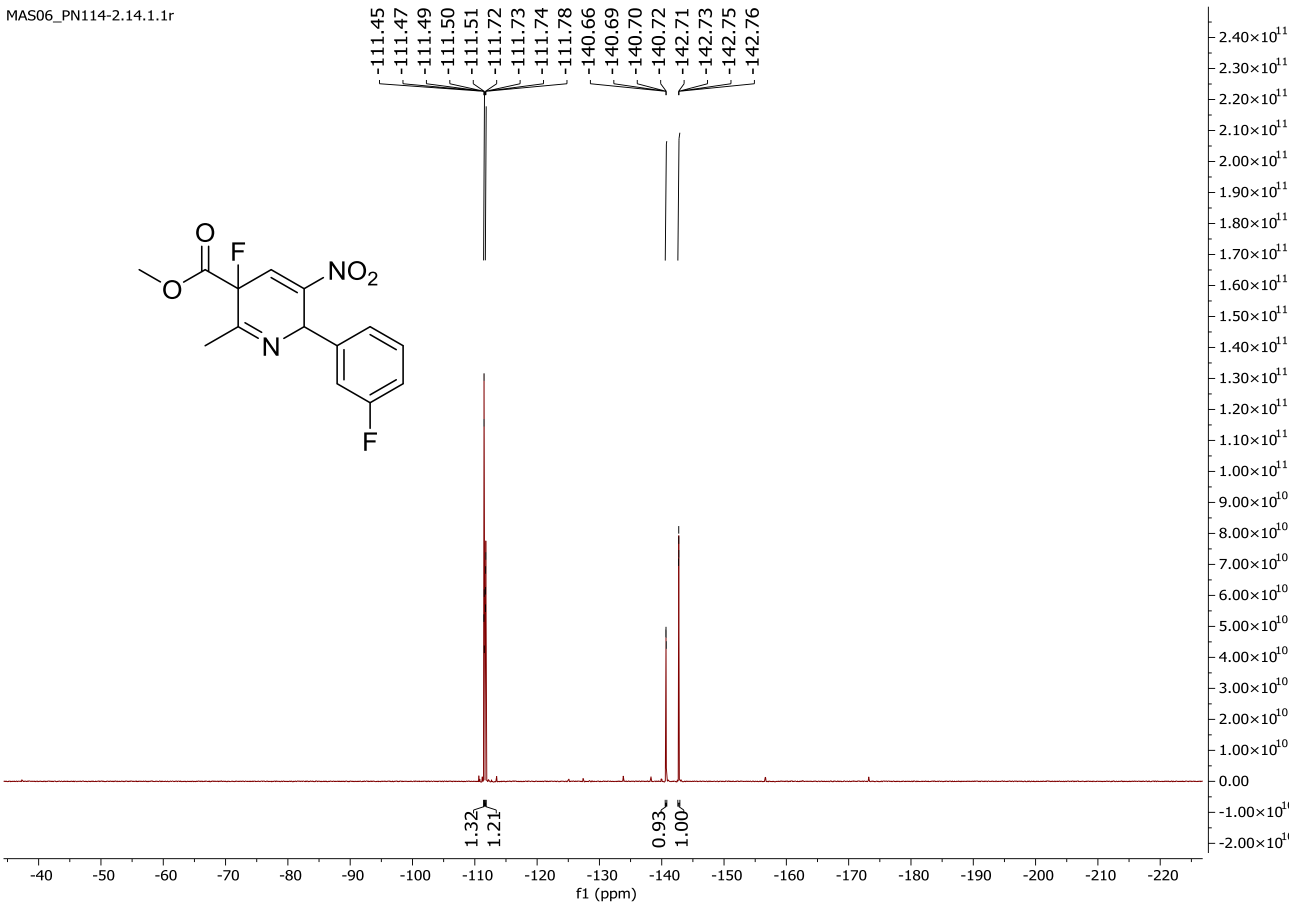

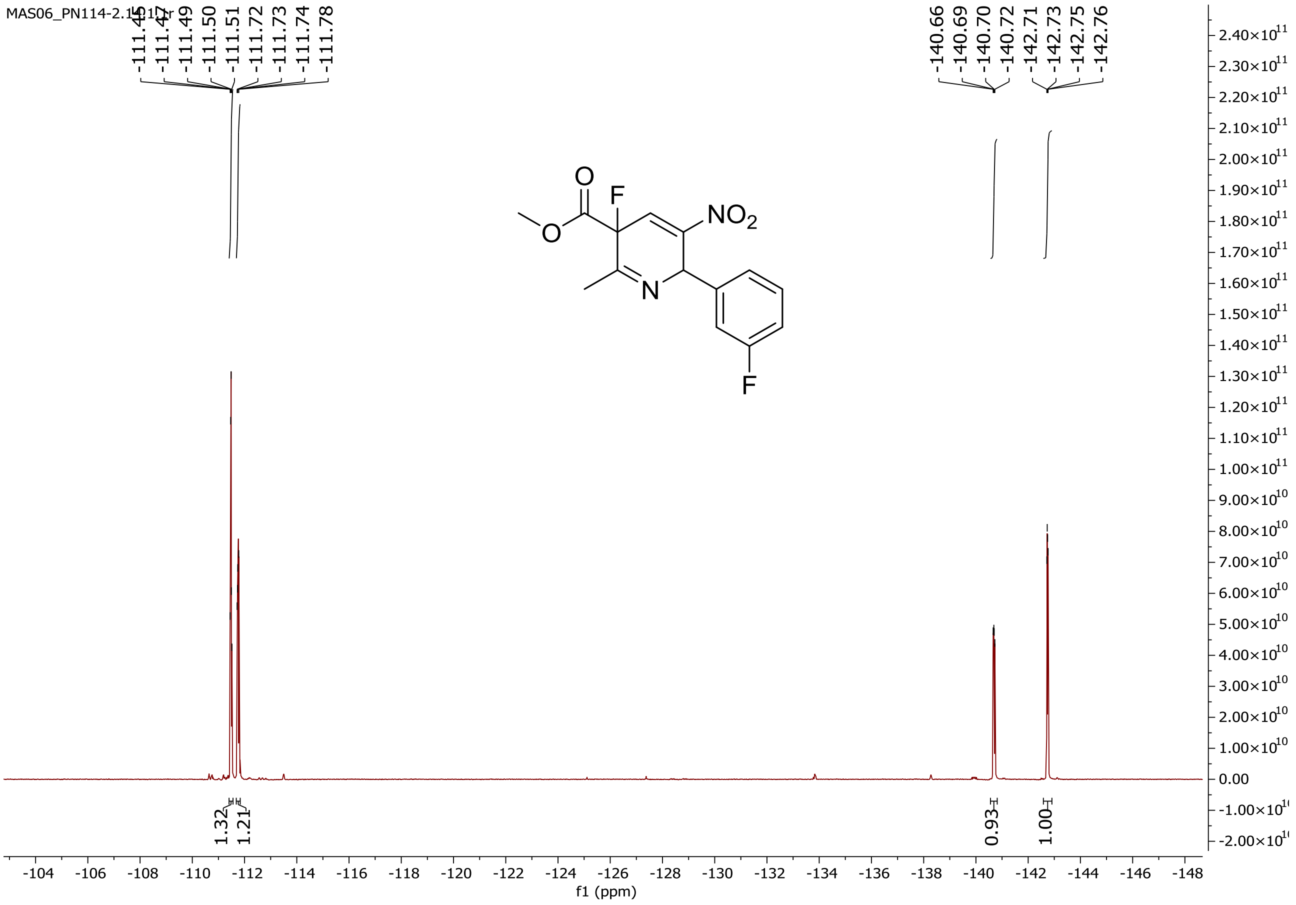

^{13}C NMR spectrum of compound **2g**

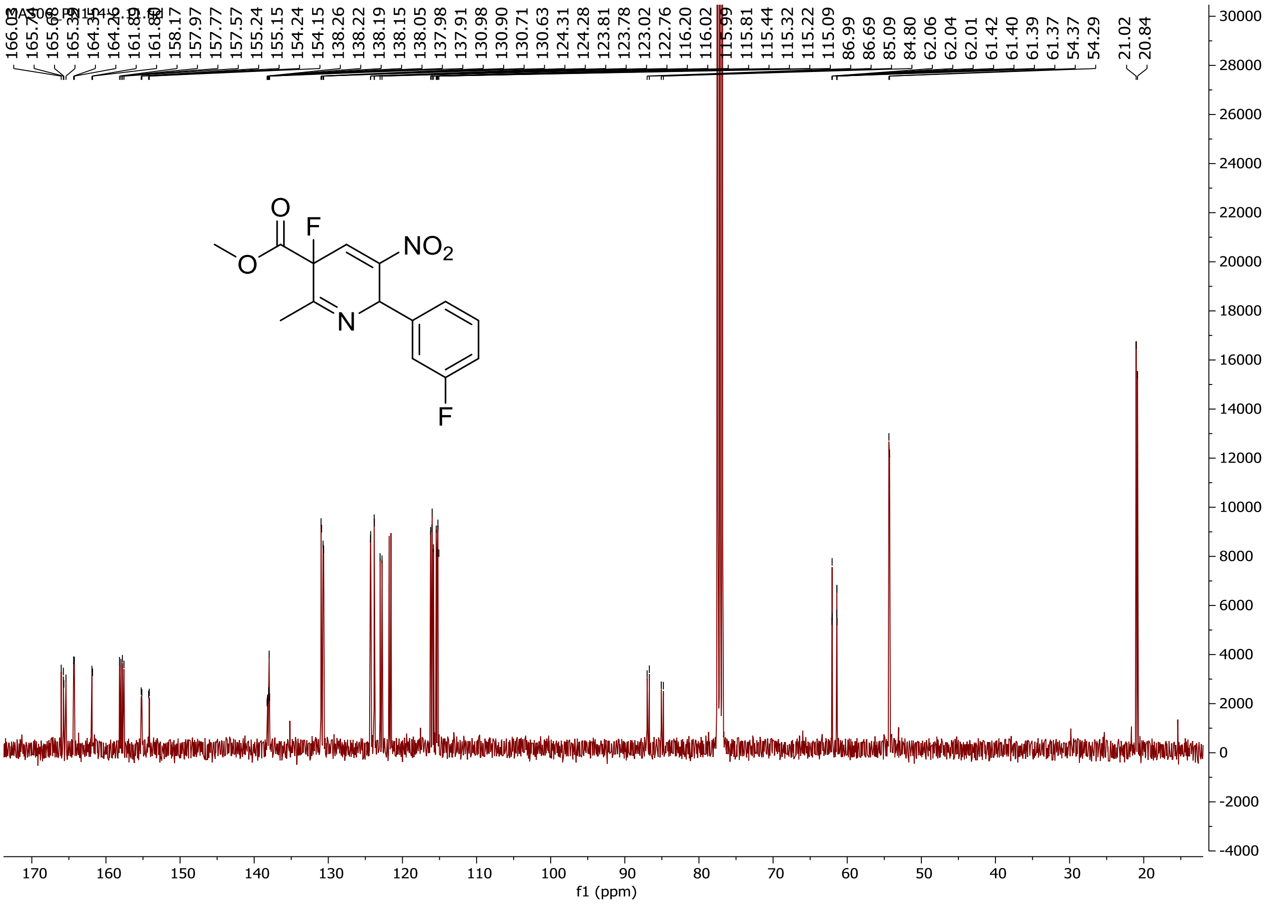

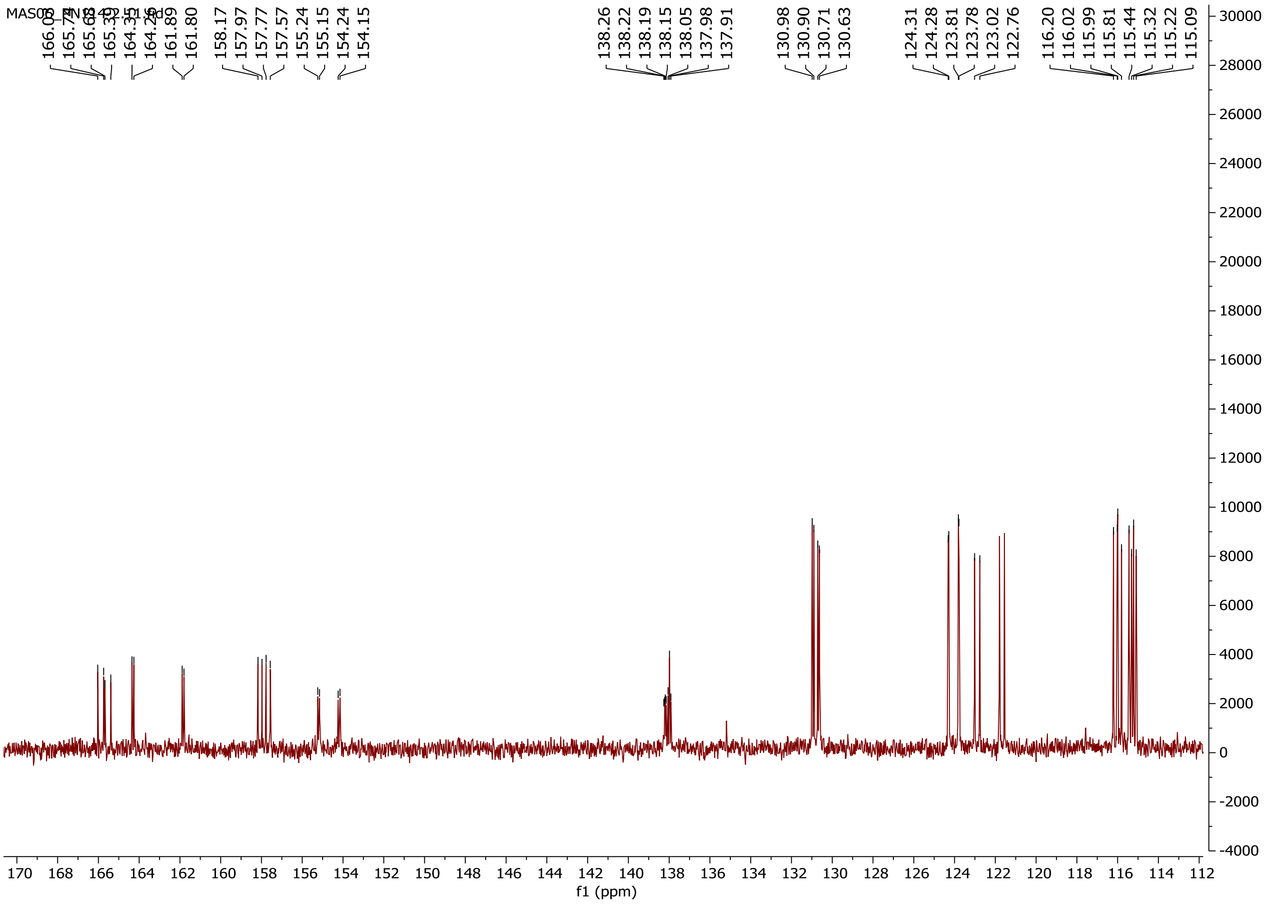

{^{13}C–^{1}H} HSQC spectrum of compound **2g**

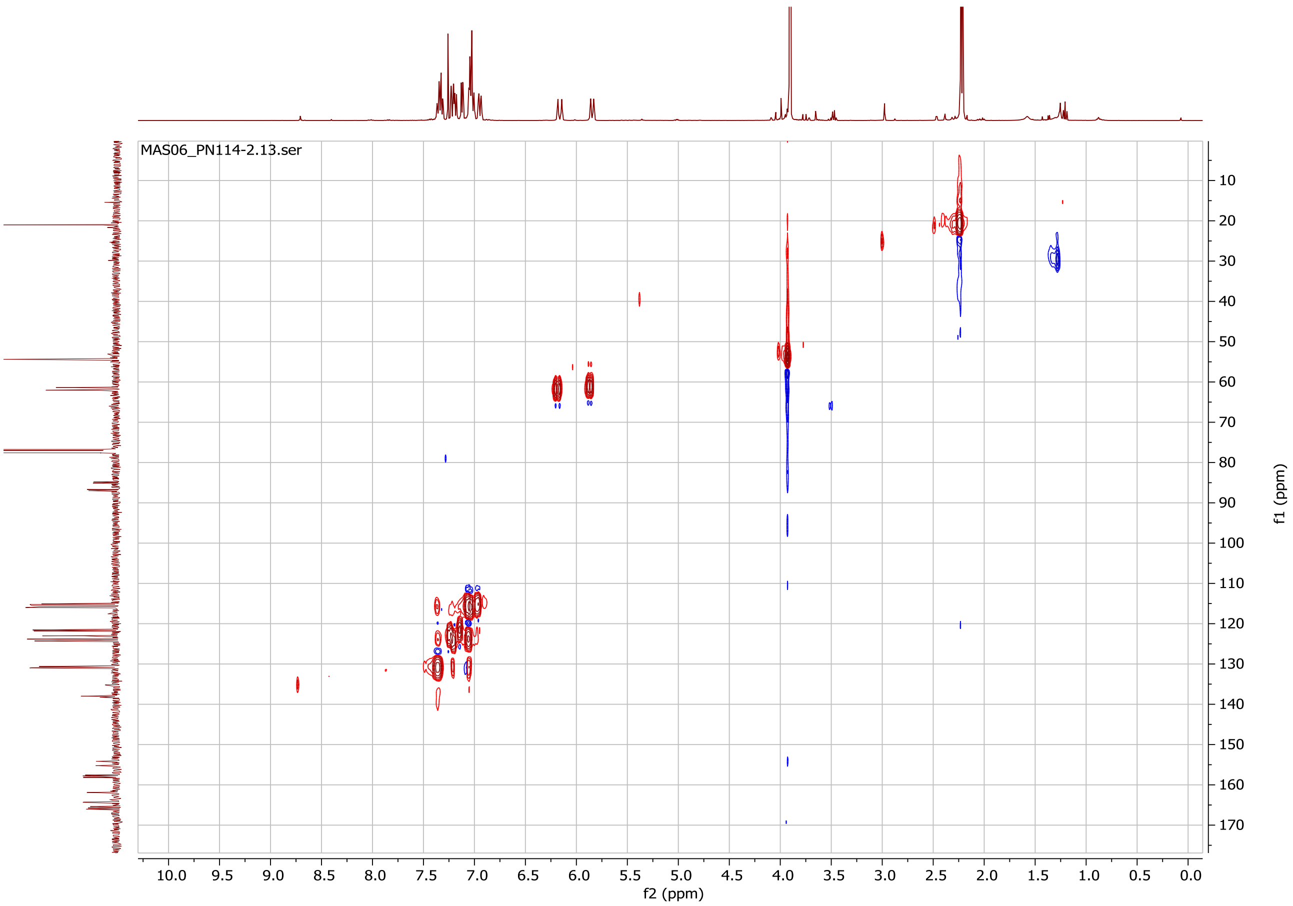

{^{13}C–^{1}H} HMBC spectrum of compound **2g**

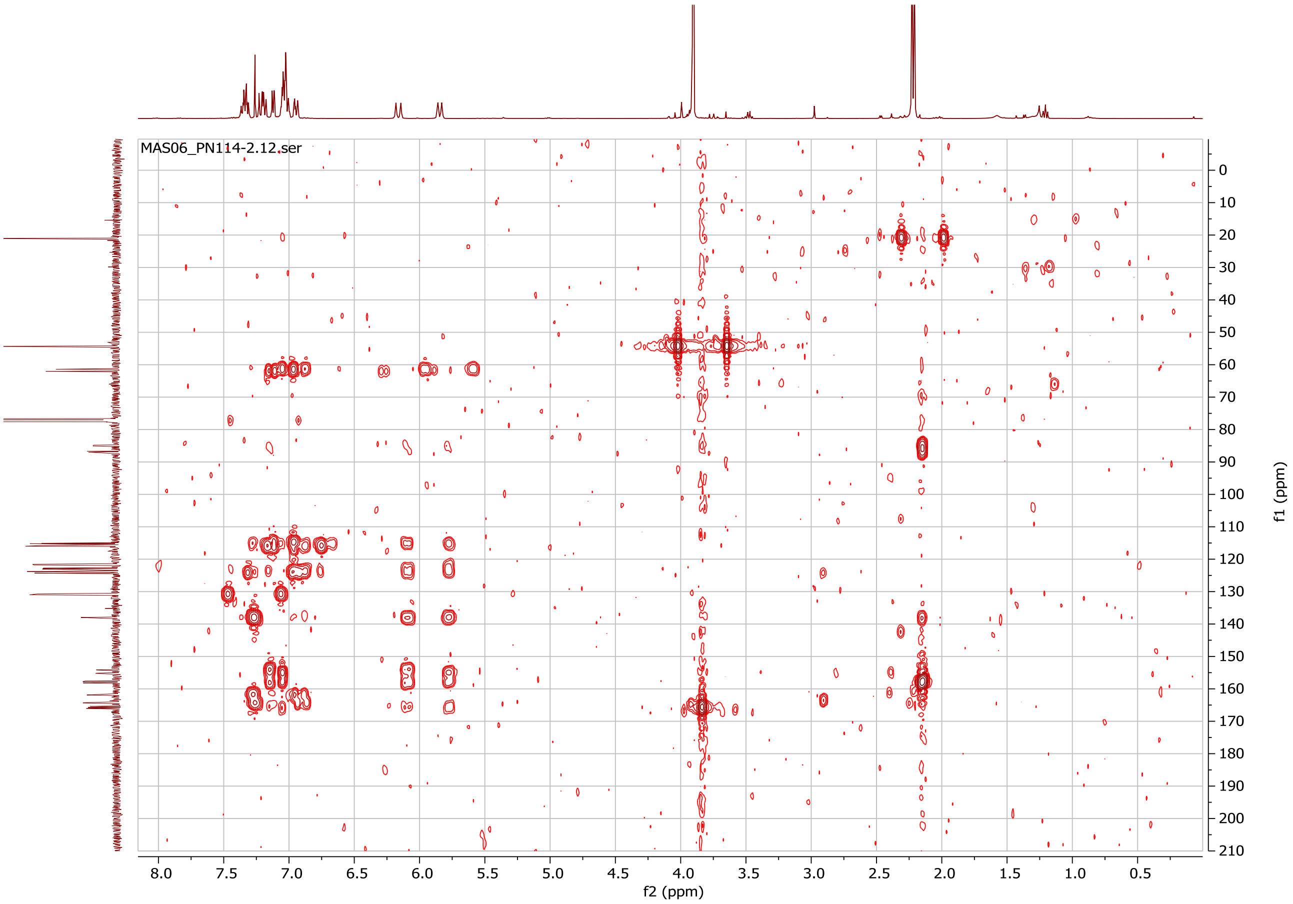

^{1}H NMR spectrum of compound **2h**

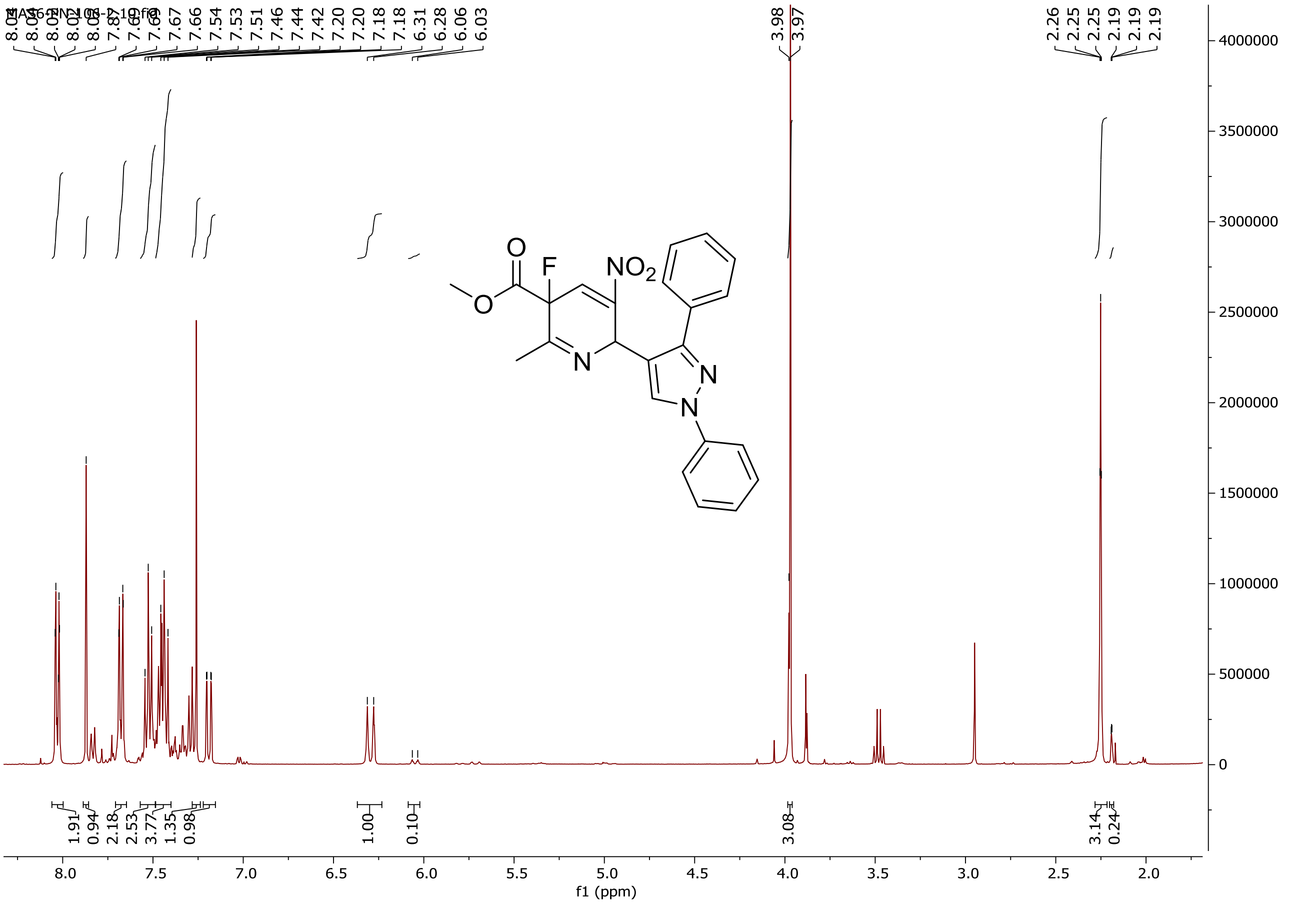

^{19}F NMR spectrum of compound **2h**

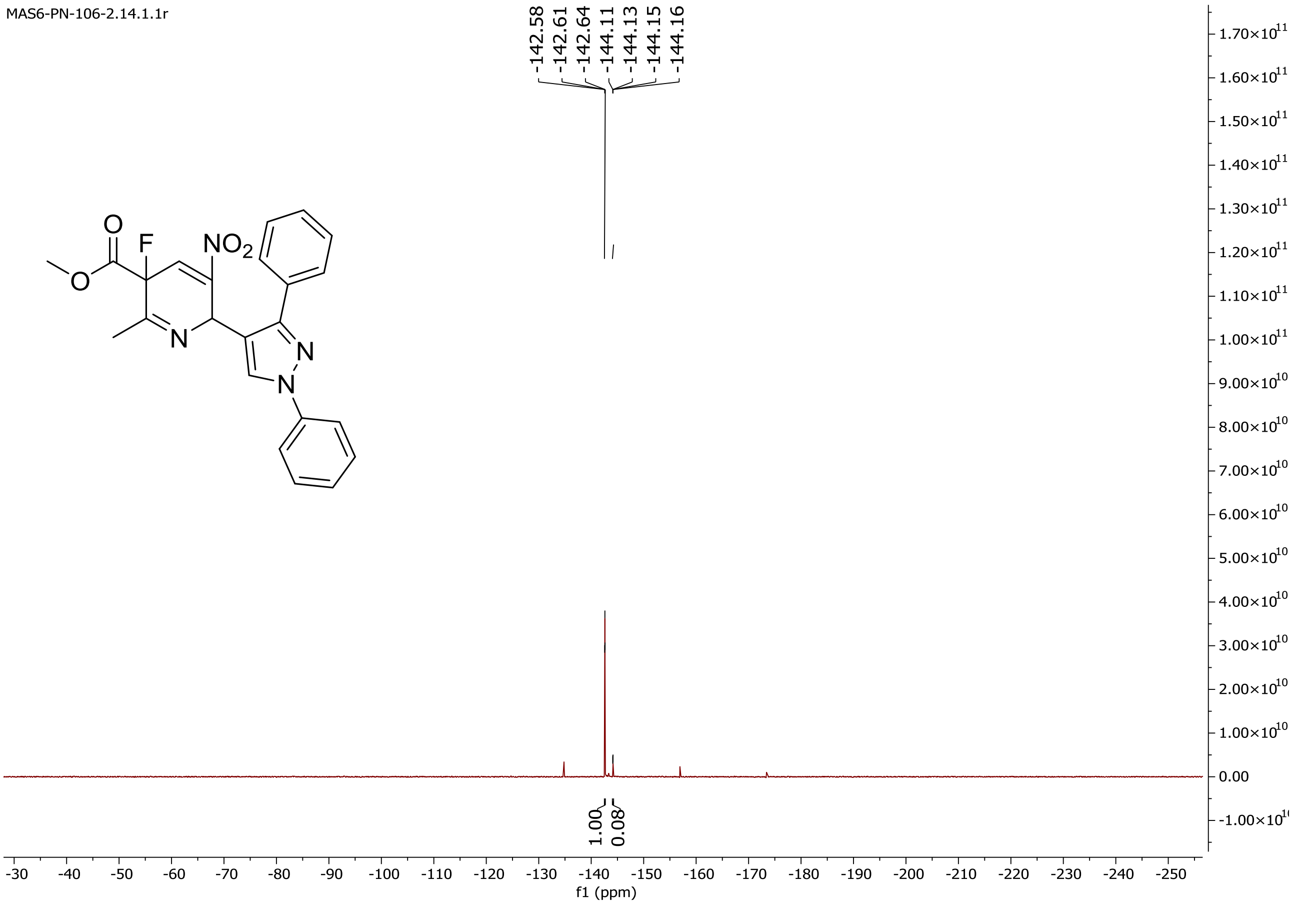

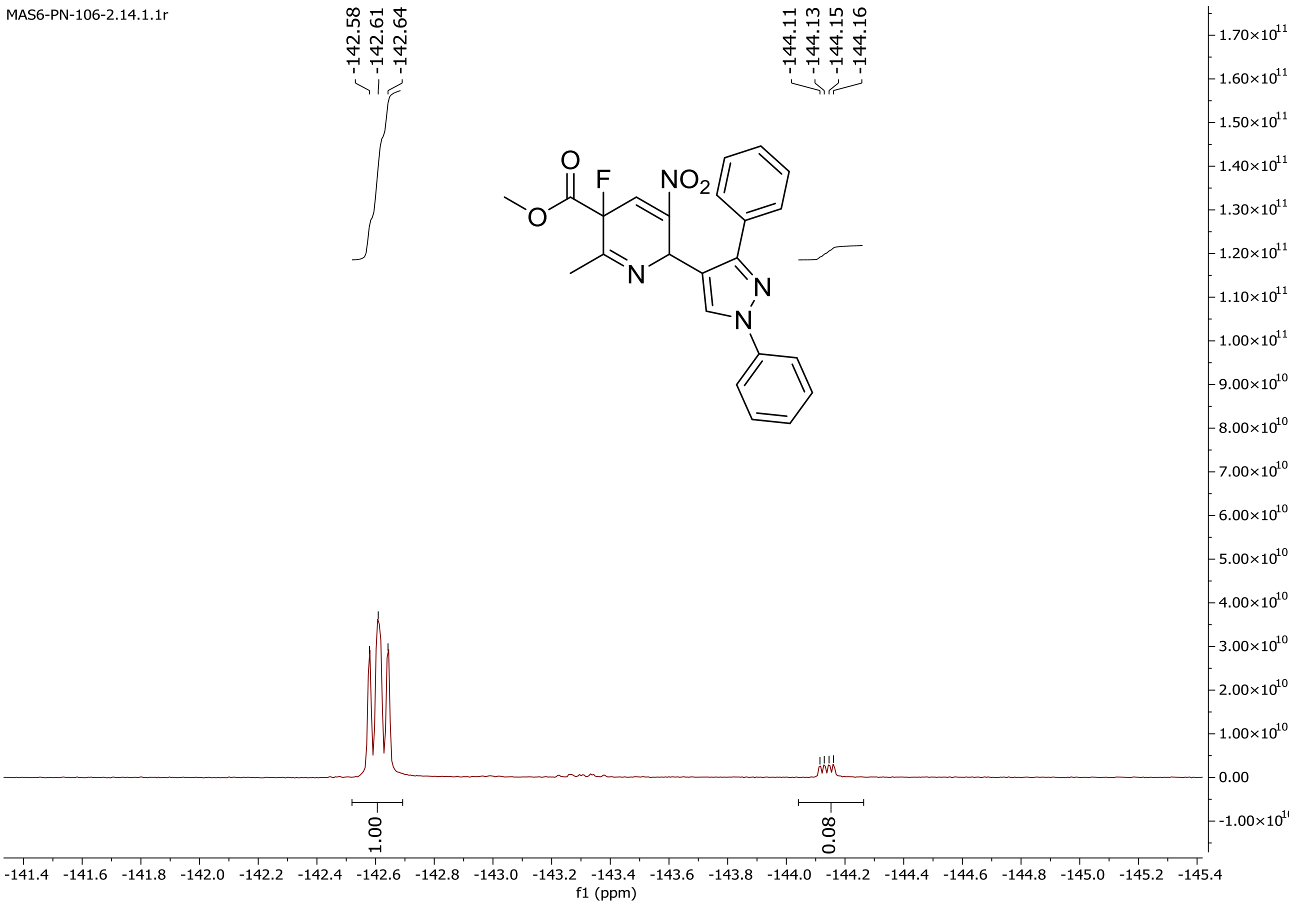

^{13}C NMR spectrum of compound **2h**

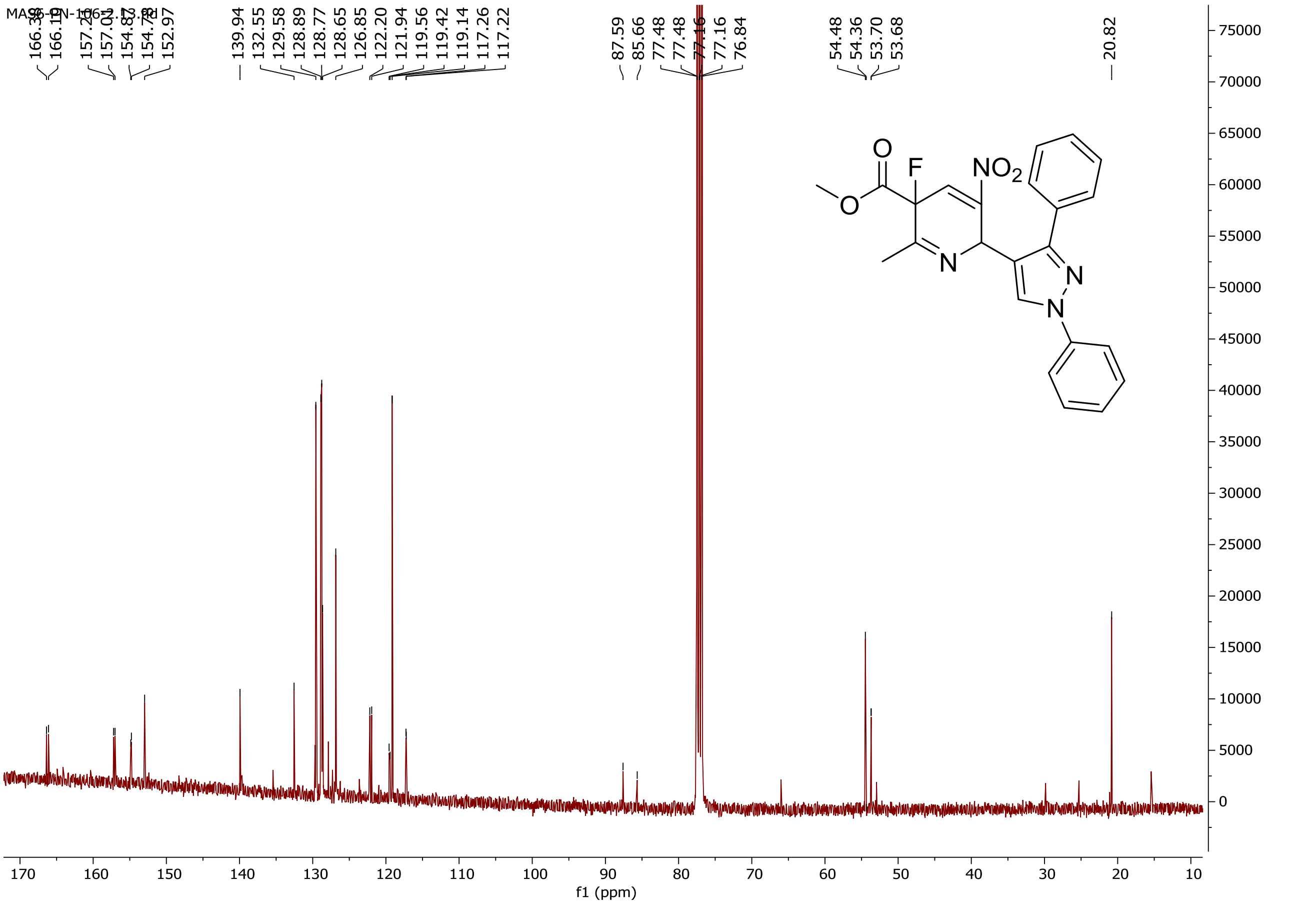

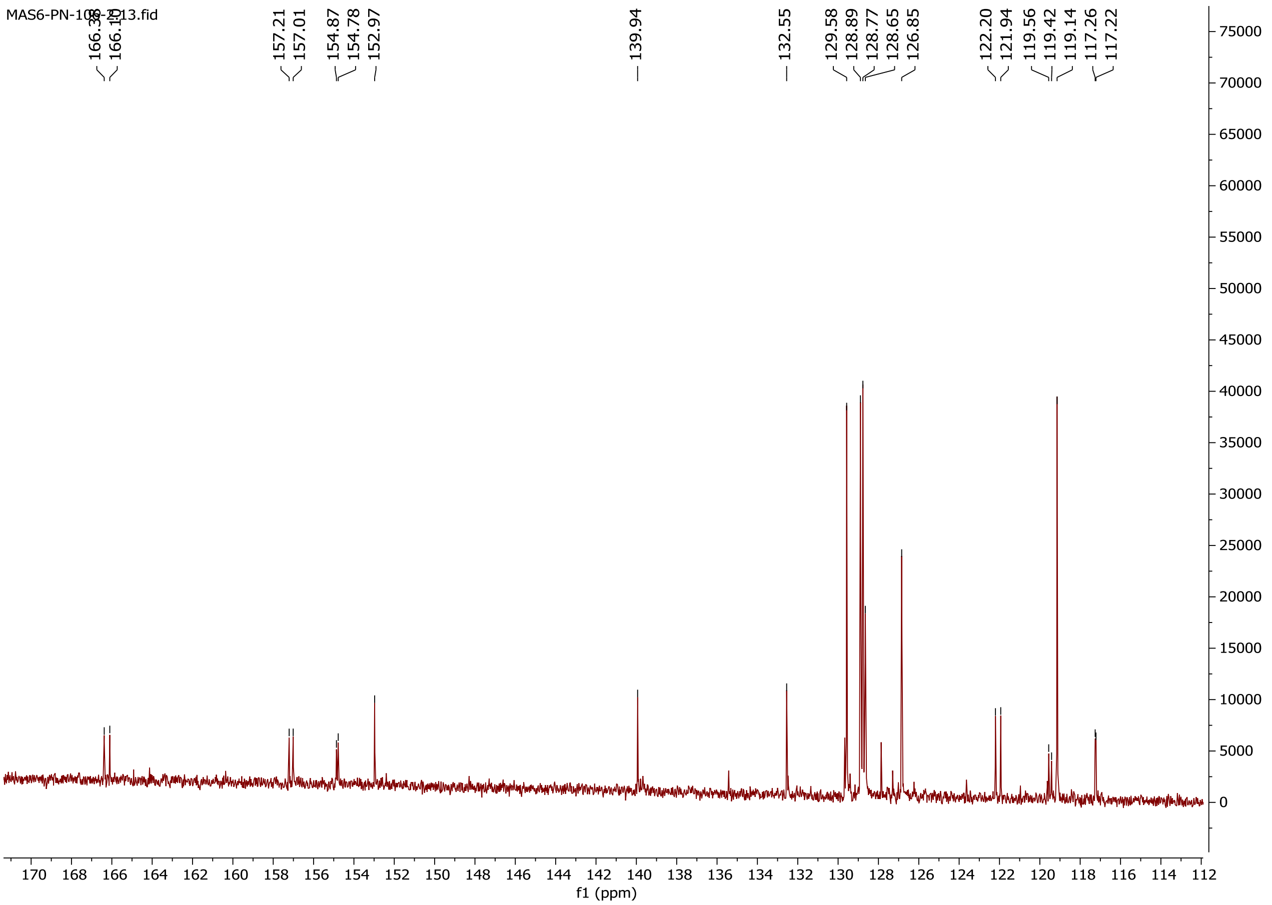

{^{13}C–^{1}H} HSQC spectrum of compound **2h**

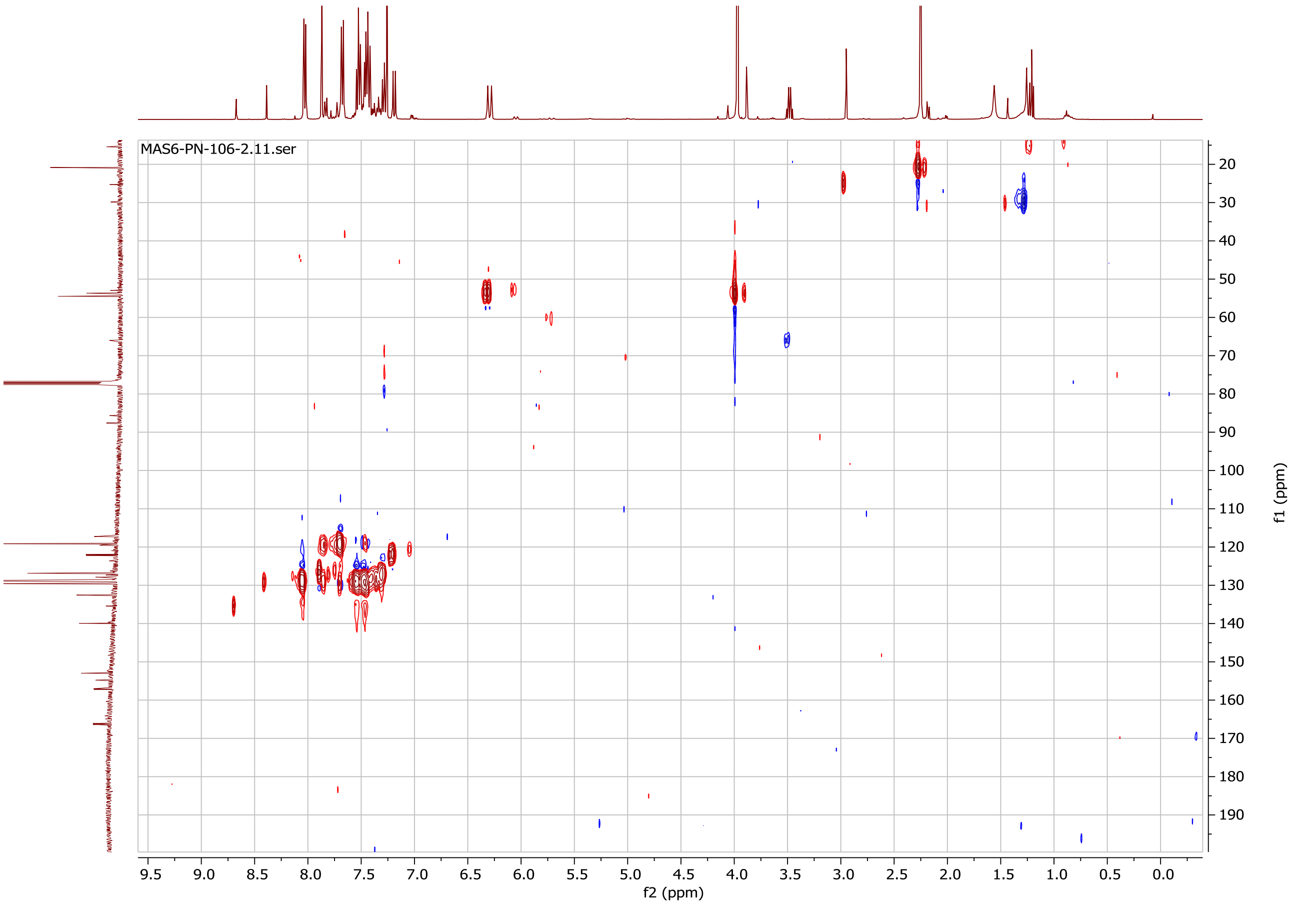

{^{13}C–^{1}H} HMBC spectrum of compound **2h**

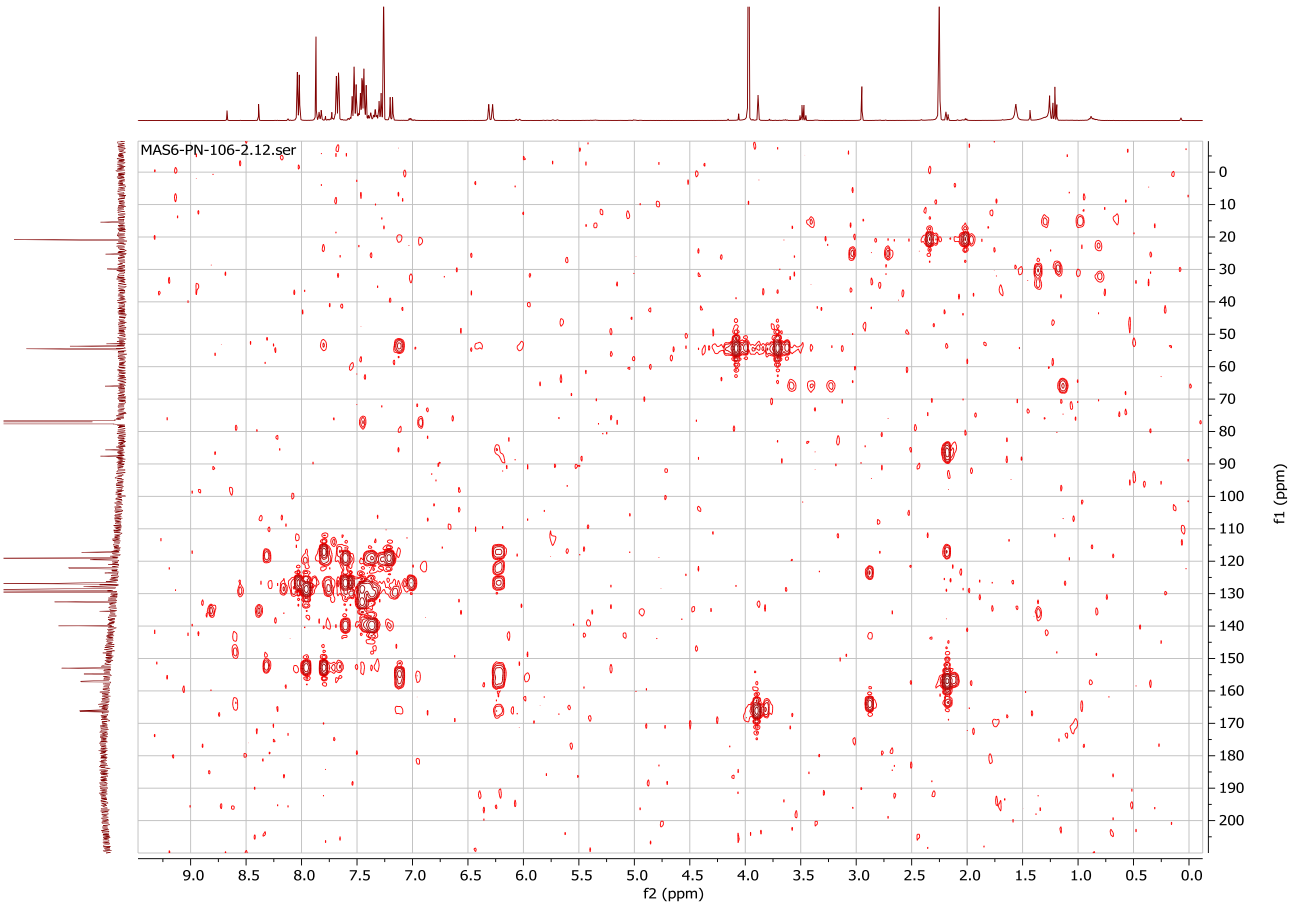

^{1}H NMR spectrum of compound **2k** (+ 12% pyridine **3k**)

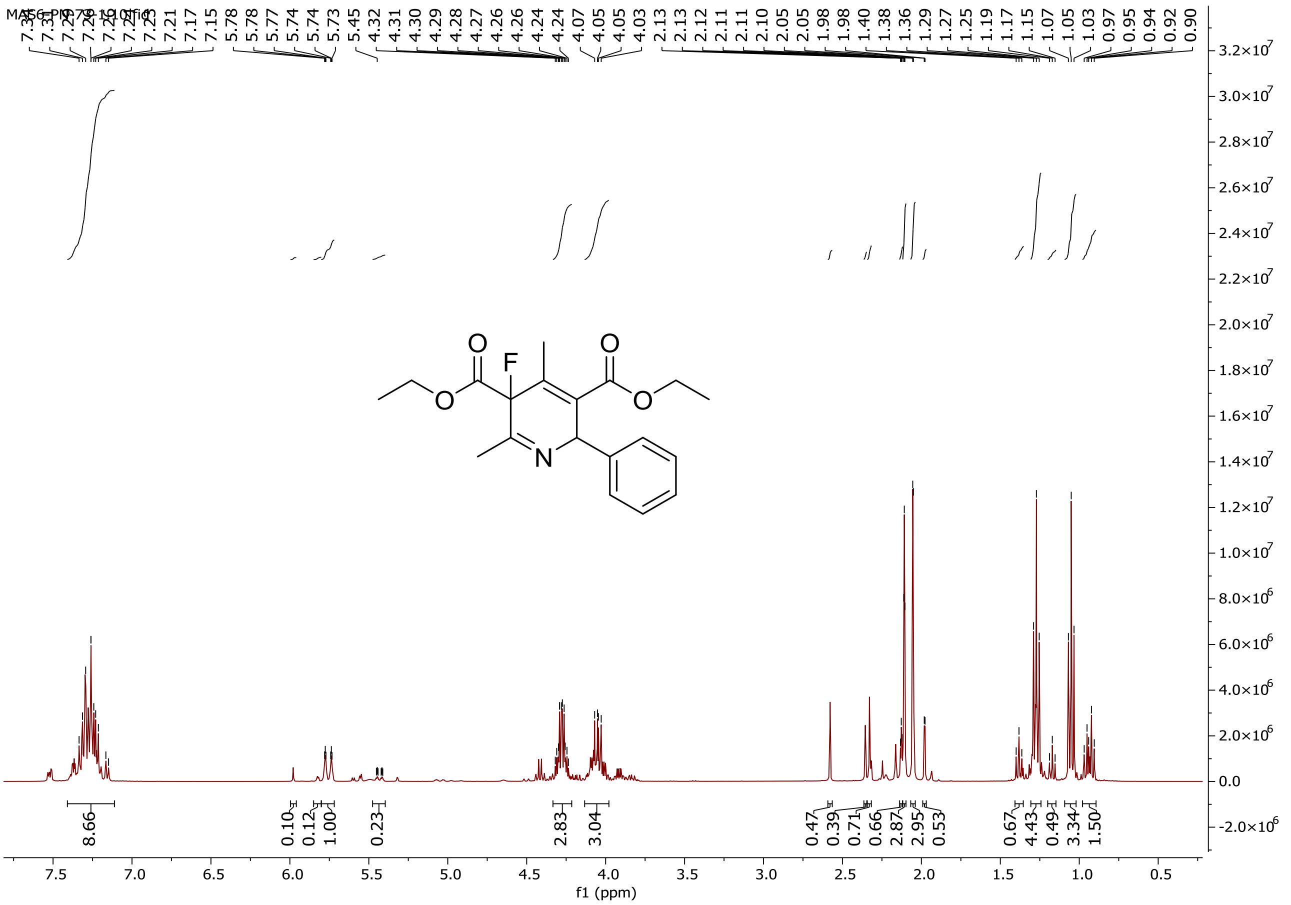

^{19}F NMR spectrum of compound **2k**

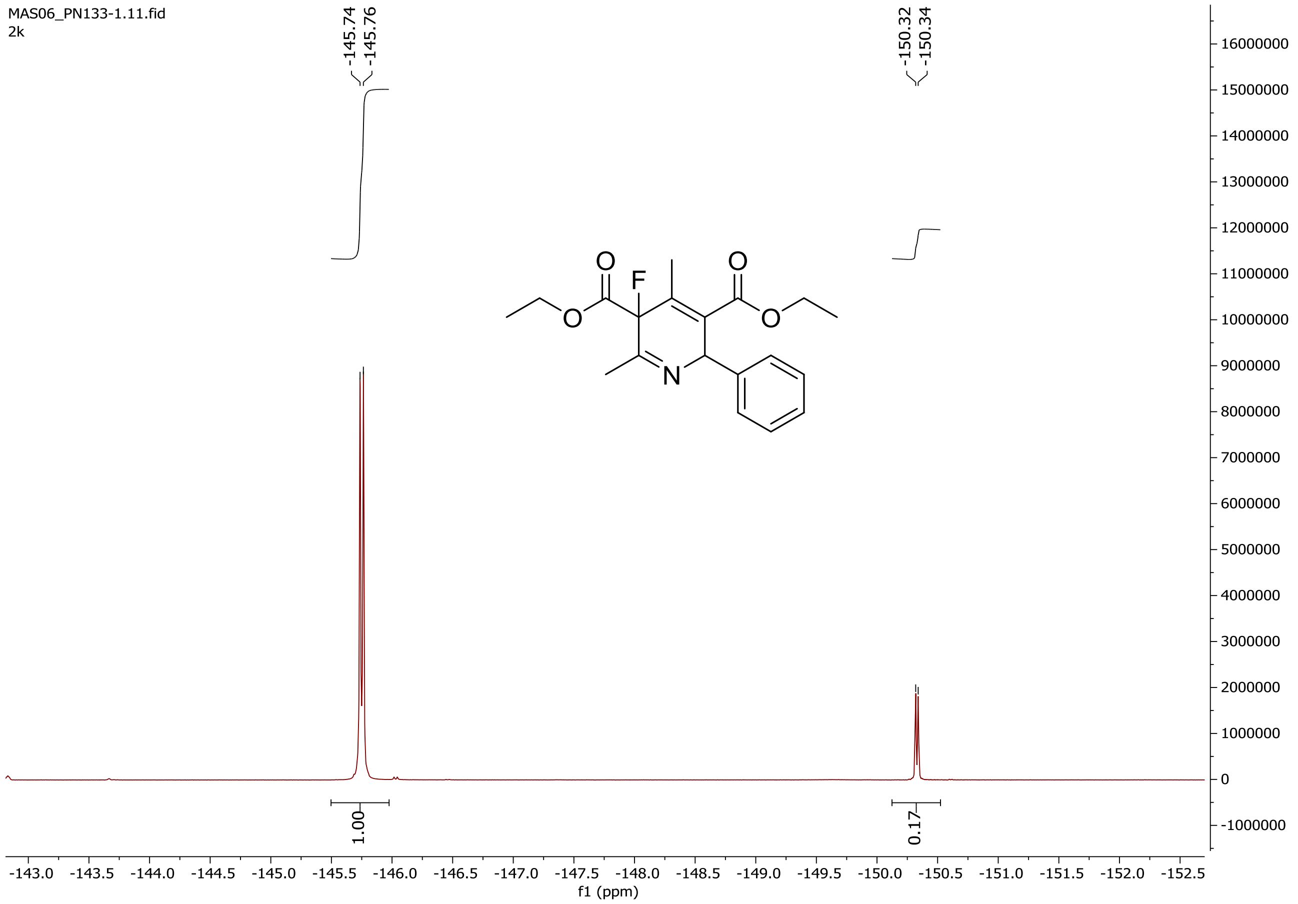

^{19}F NMR spectrum with ^{1}H decoupling of compound **2k**

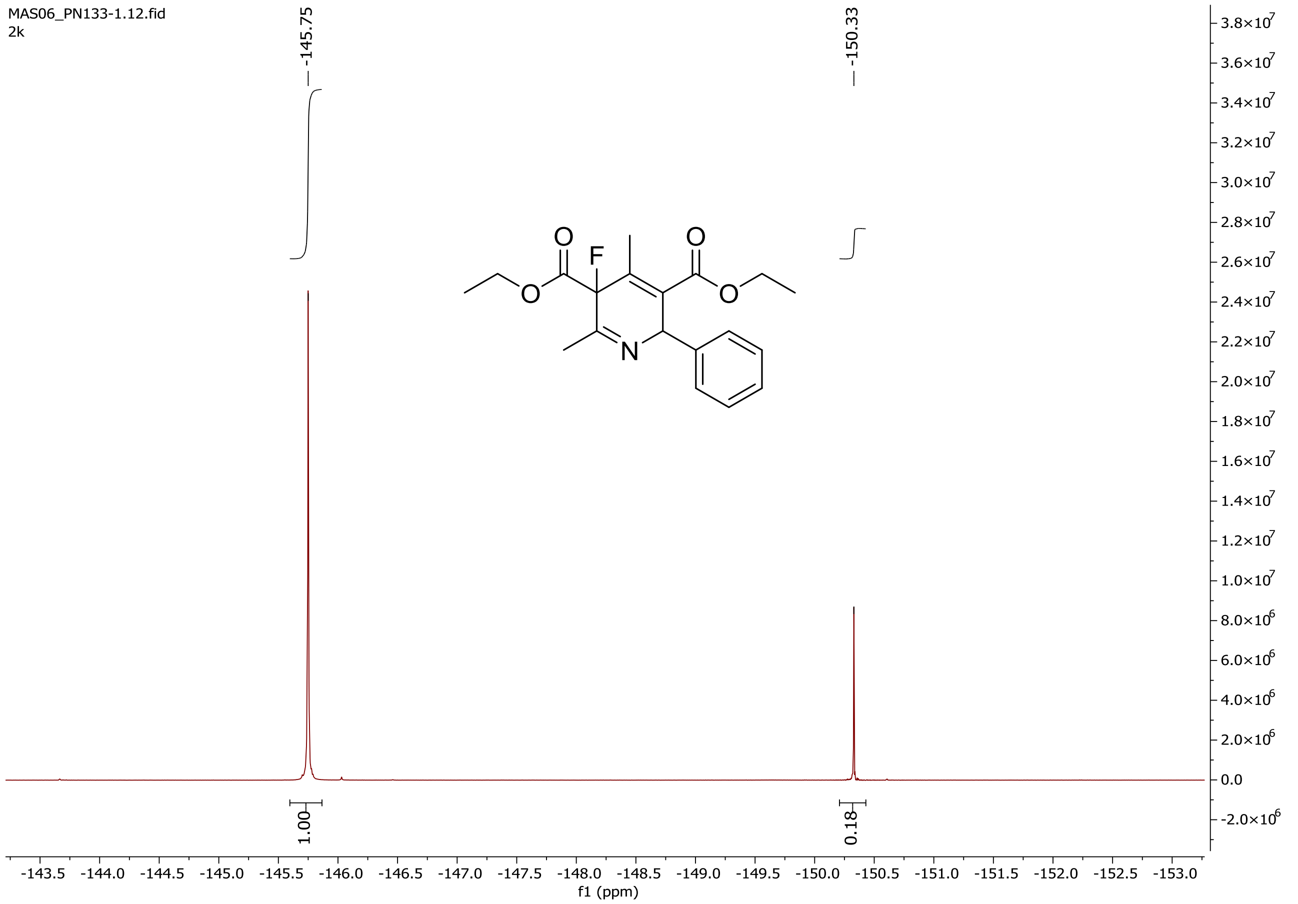

^{13}C NMR spectrum of compound **2k** (+ 12% pyridine **3k**)

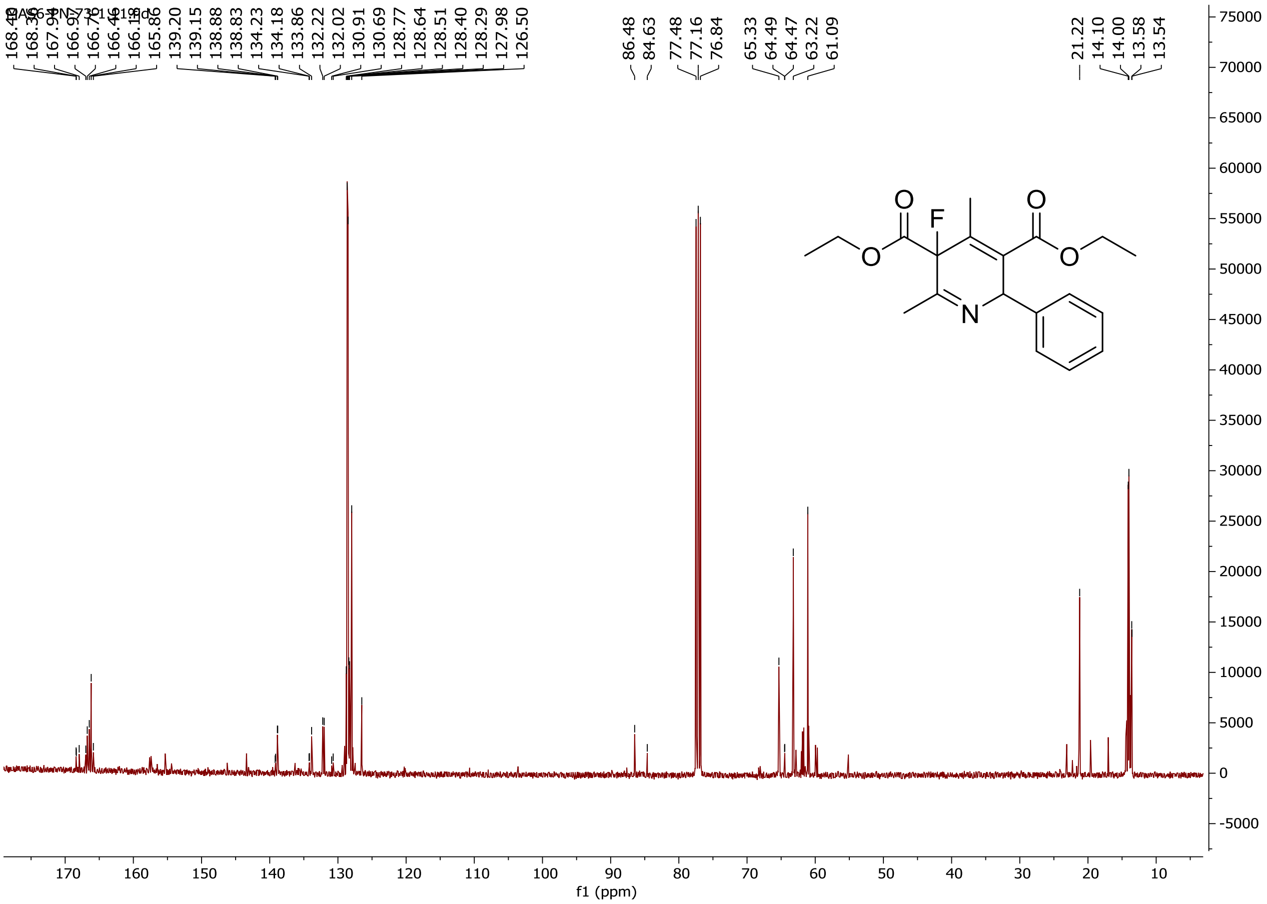

{^{1}H–^{1}H} COSY spectrum of compound **2k**

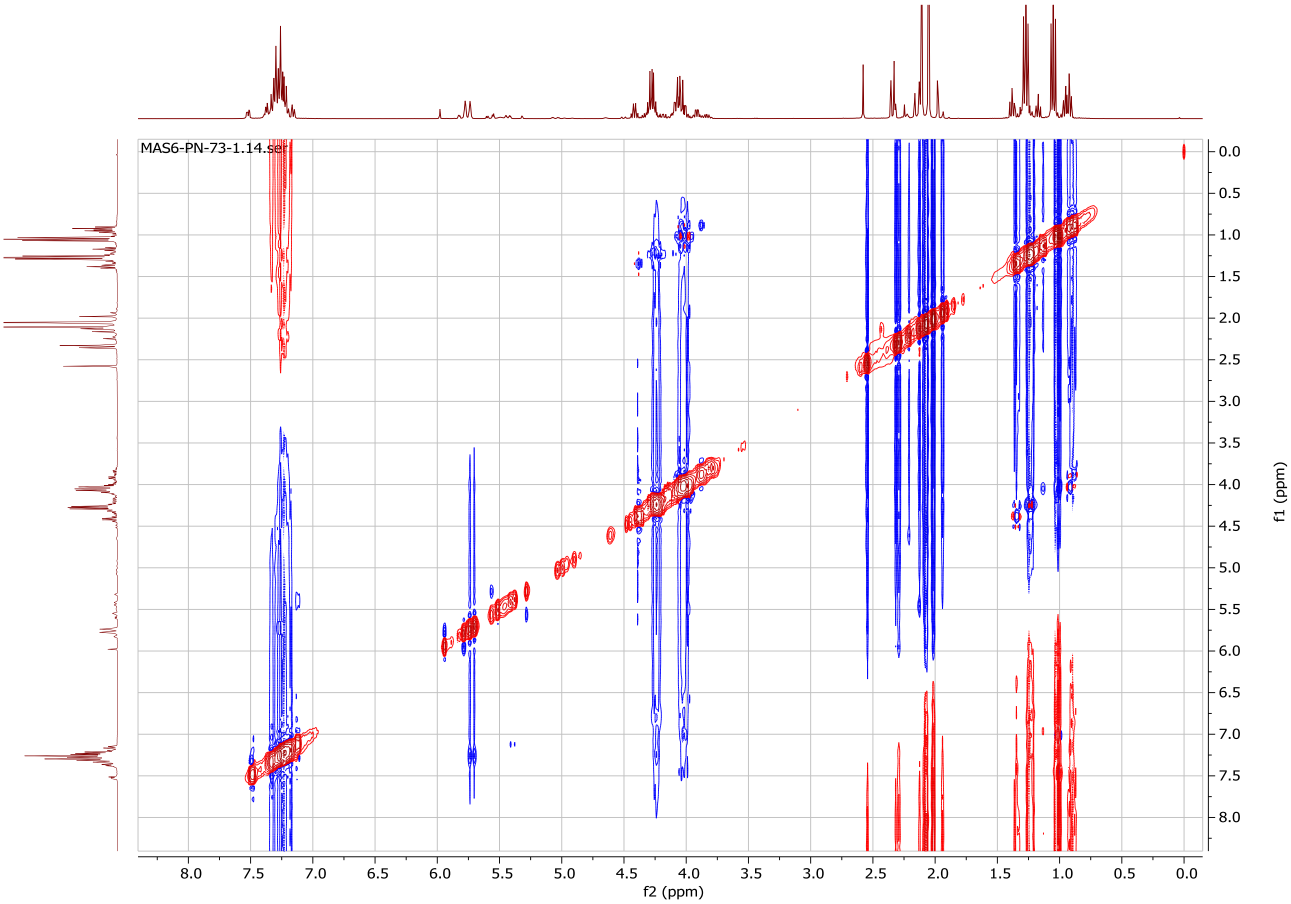

{^{13}C–^{1}H}HSQC spectrum of compound **2k**

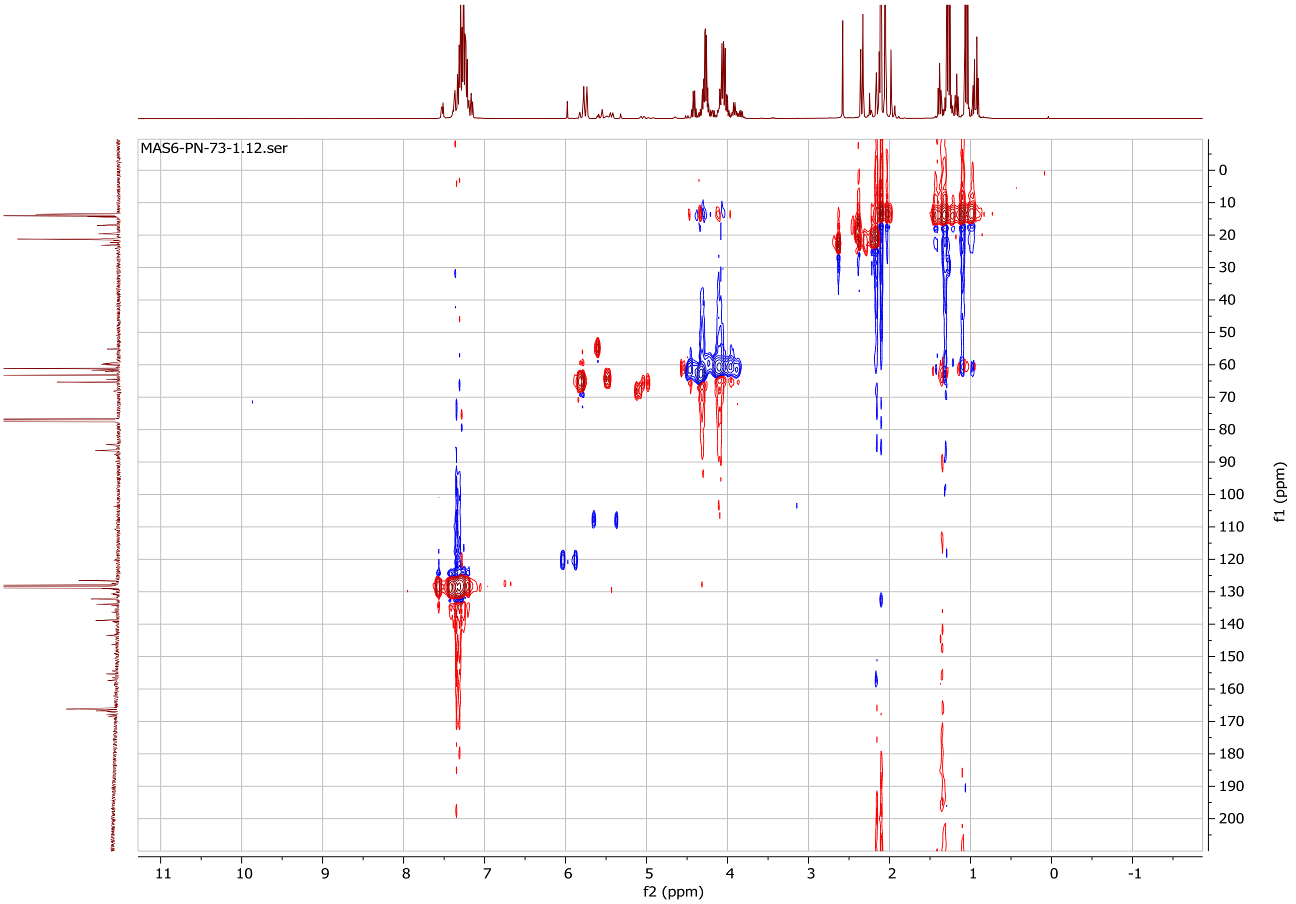

{^{13}C–^{1}H} HMBC spectrum of compound **2k**

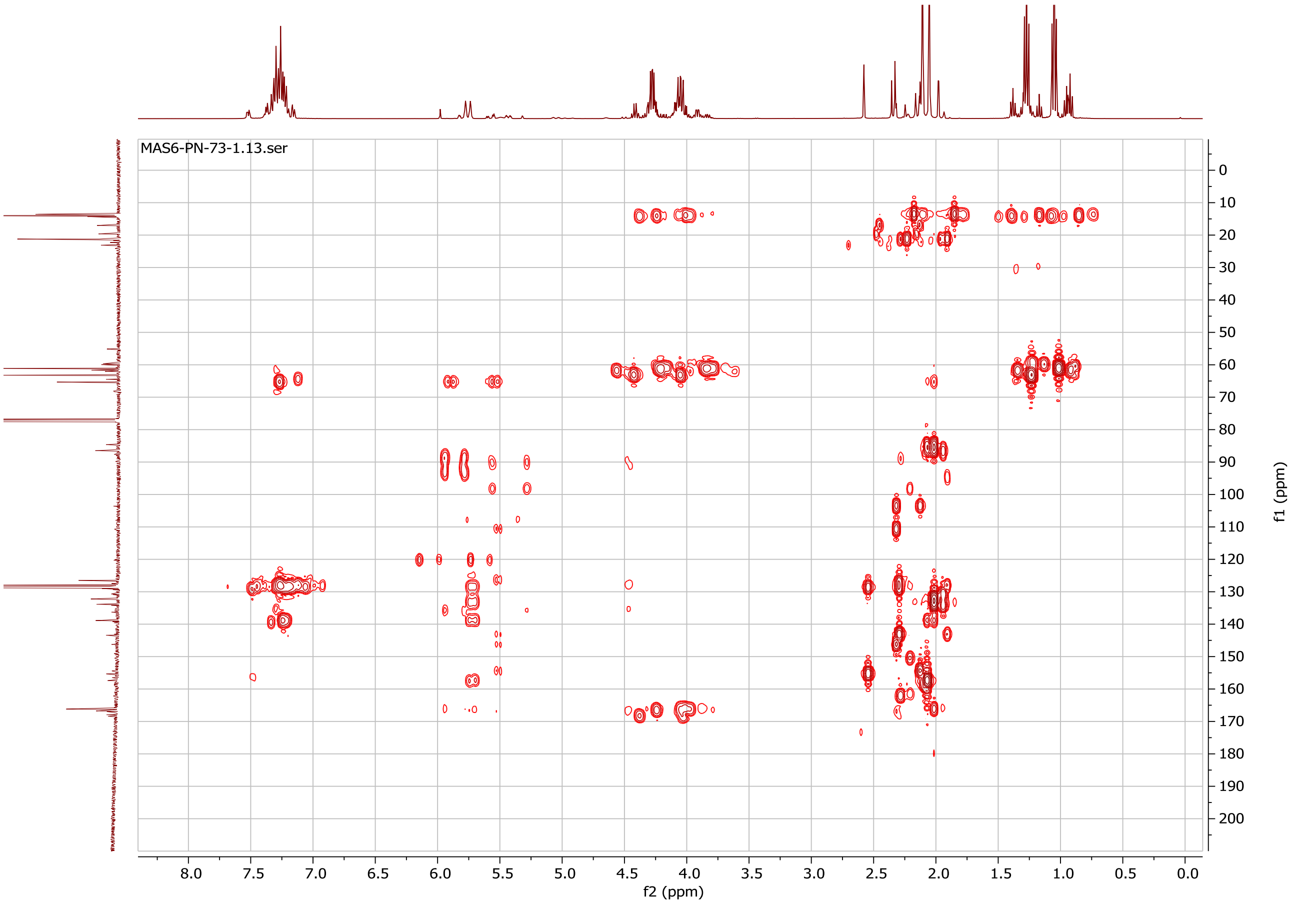

^{1}H NMR spectrum of compound **3a**

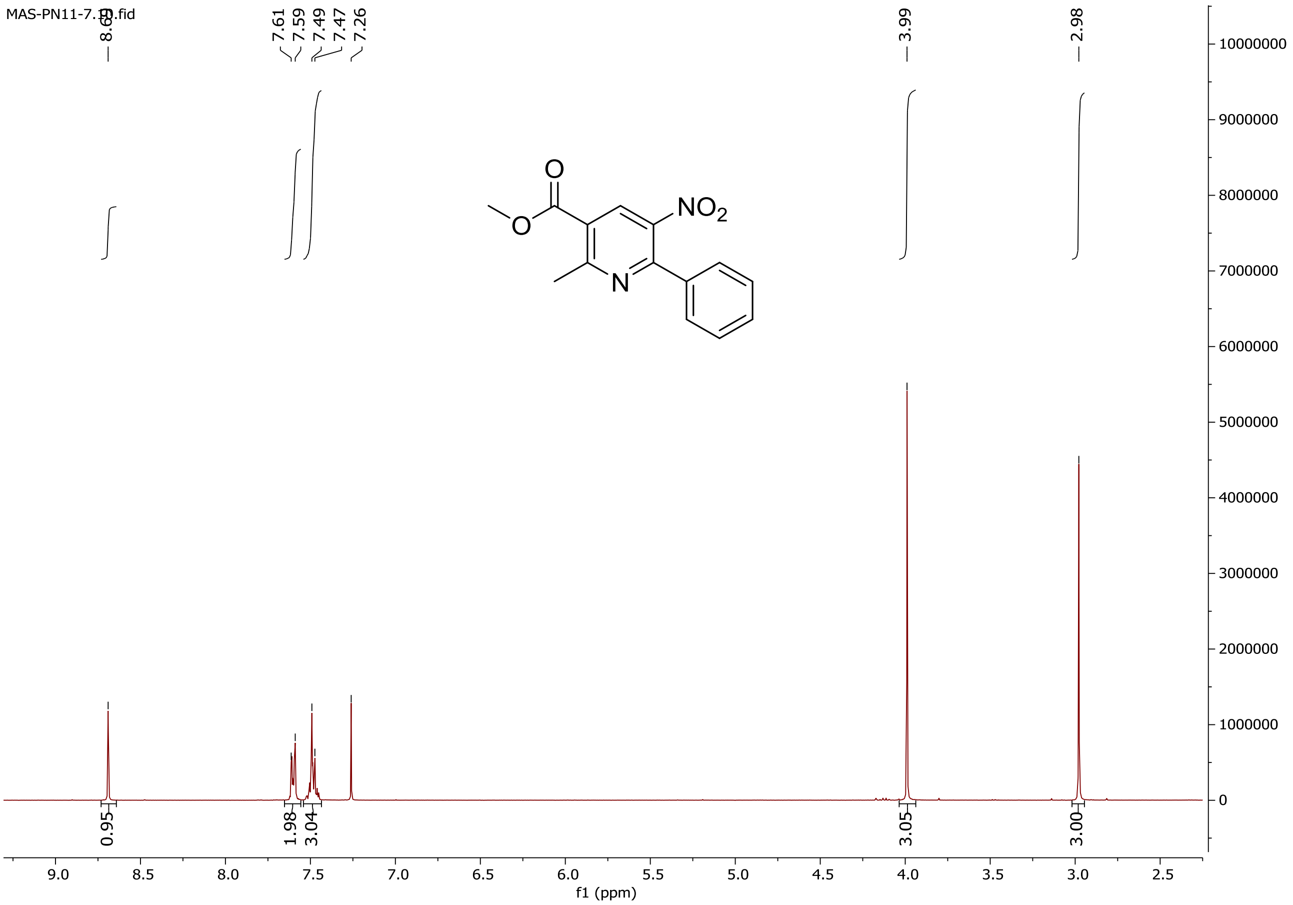

^{13}C NMR spectrum of compound **3a**

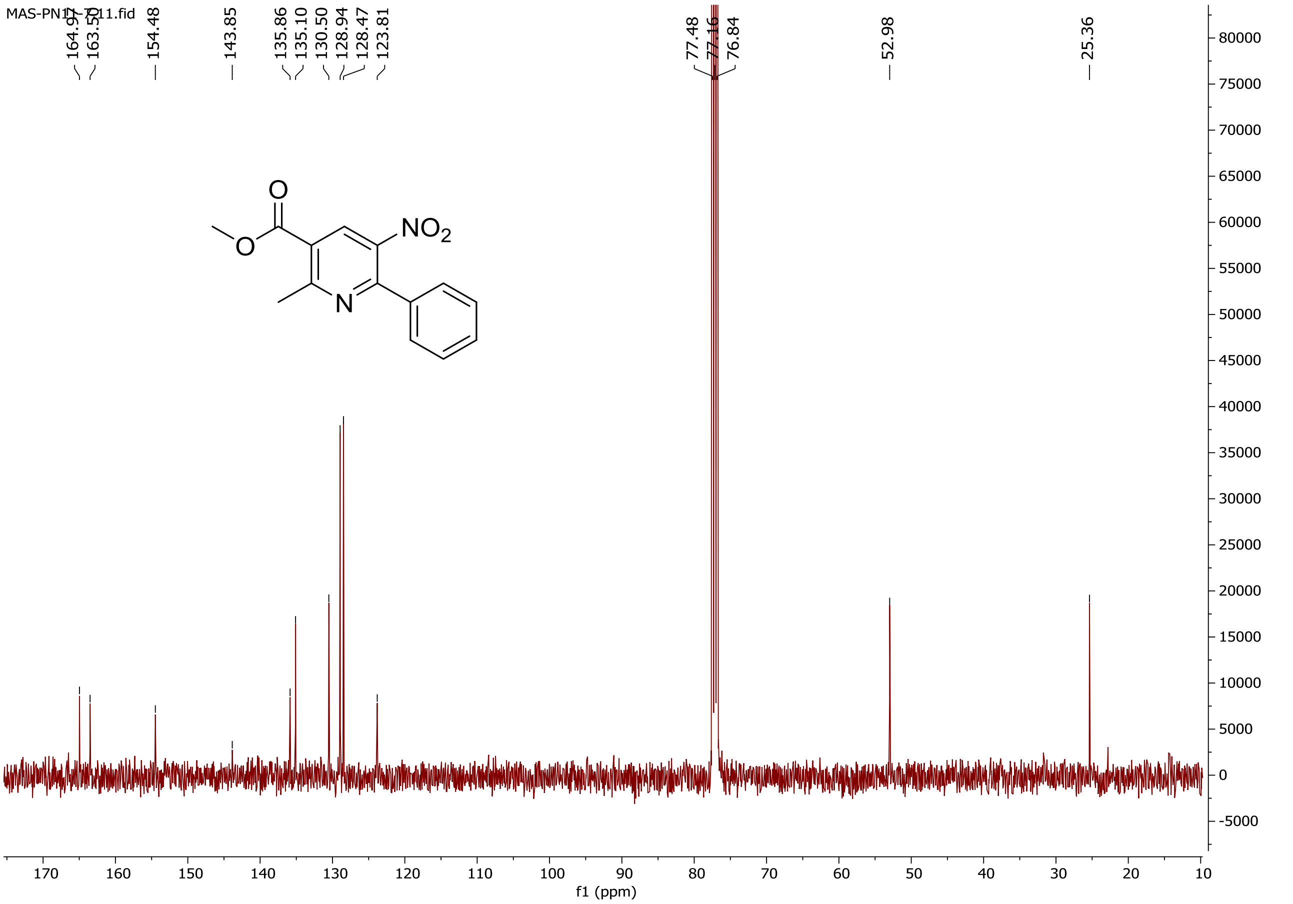

^{1}H NMR spectrum of compound **3b**

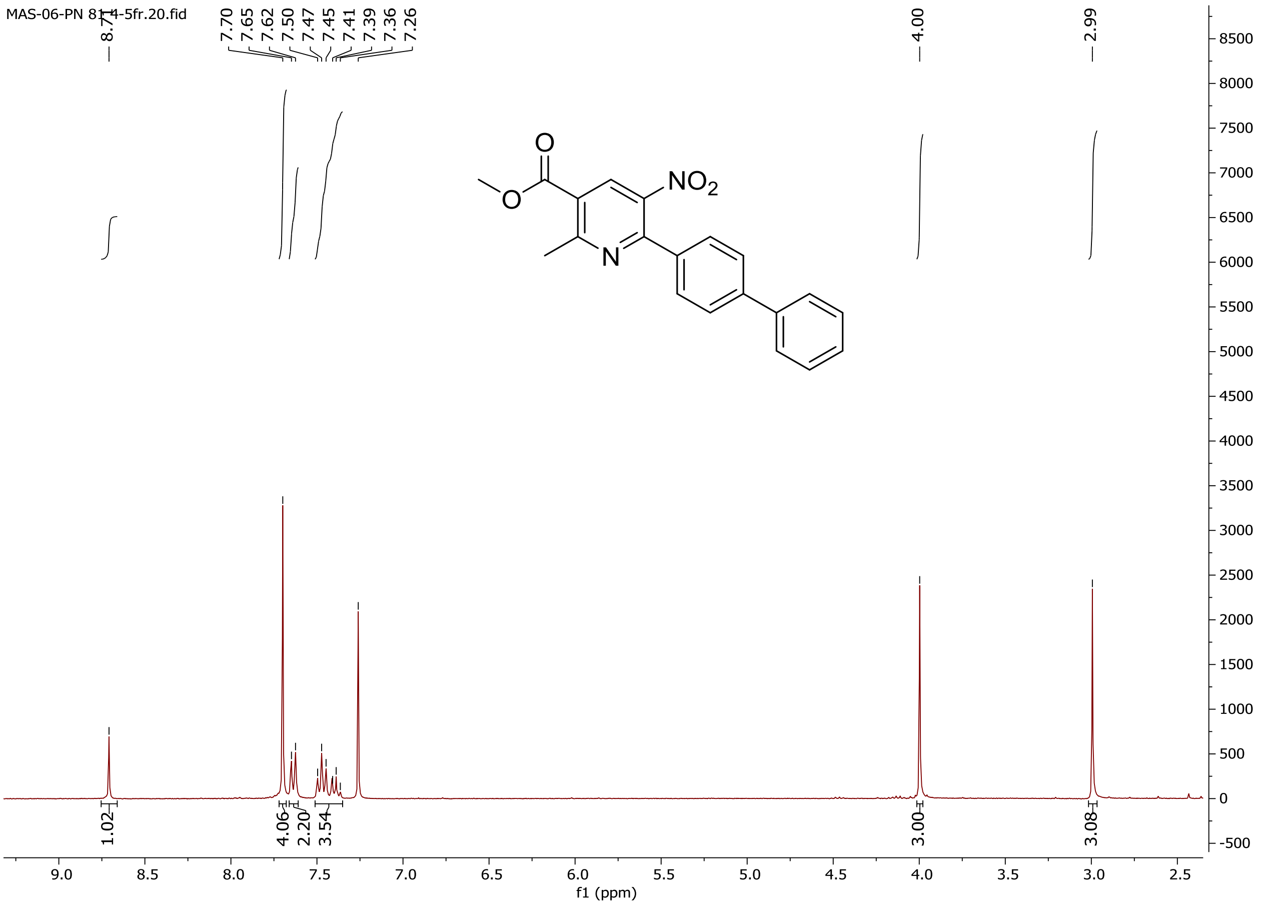

^{1}H NMR spectrum of compound **3c**

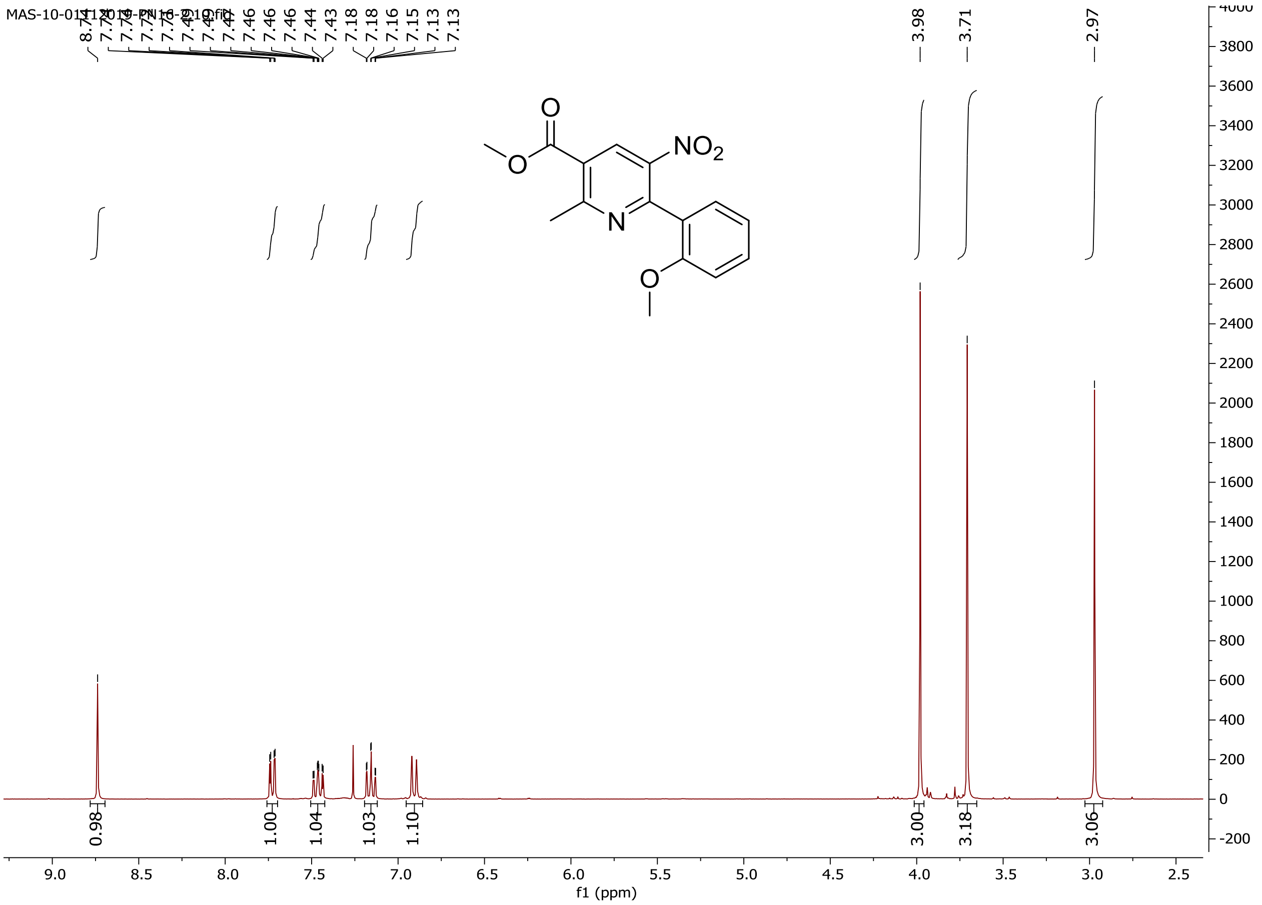

^{1}H NMR spectrum of compound **3d**

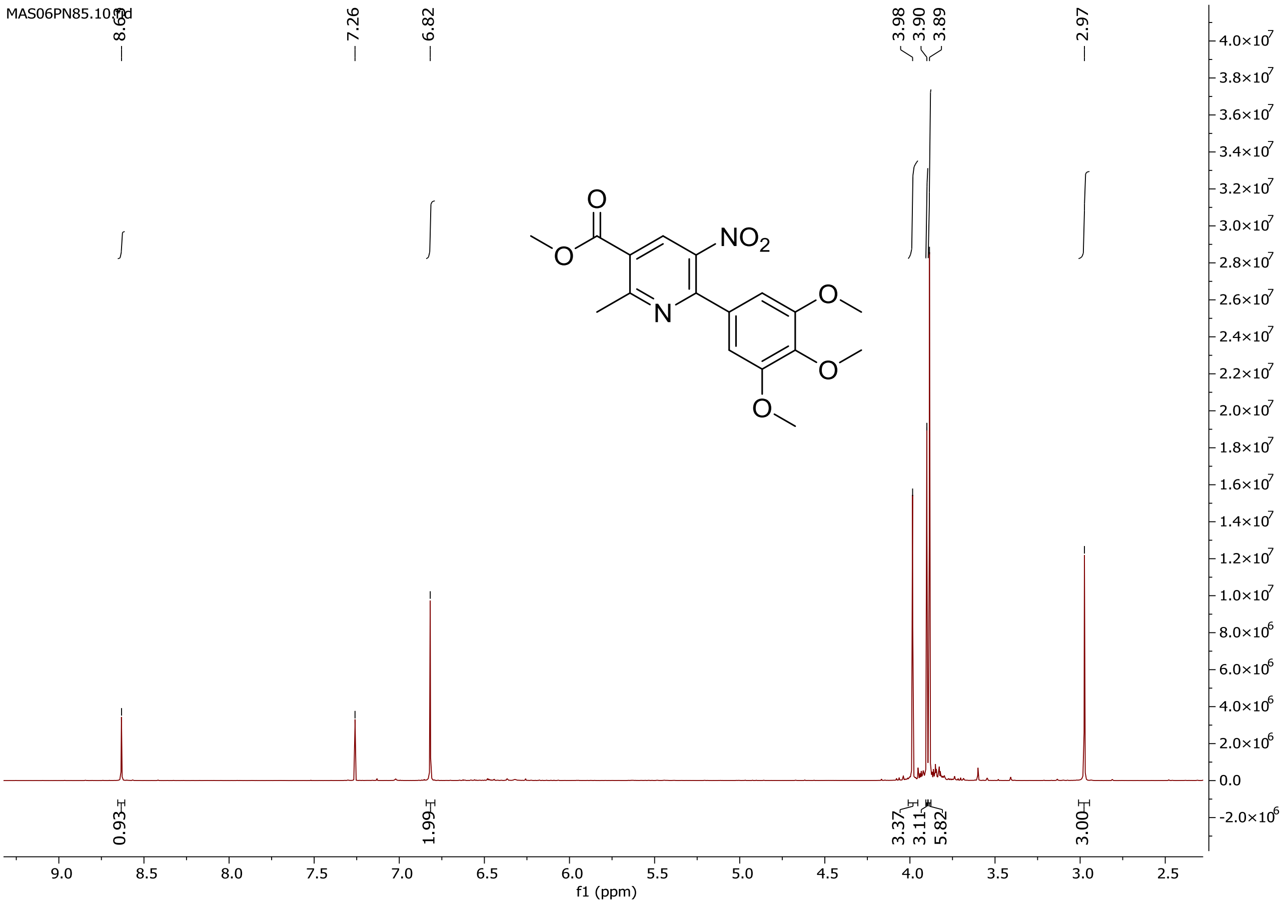

^{13}C NMR spectrum of compound **3d**

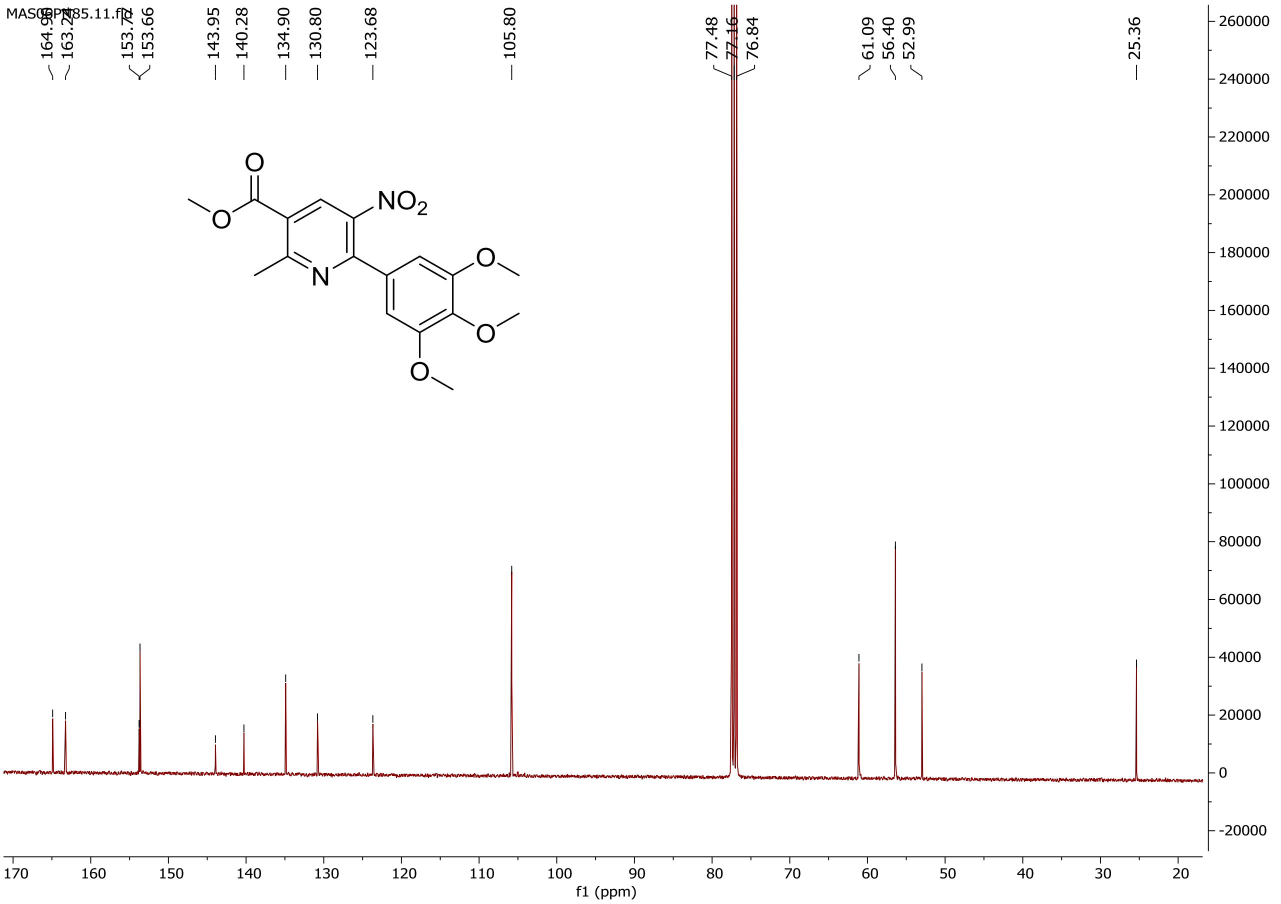

^{1}H NMR spectrum of compound **3e**

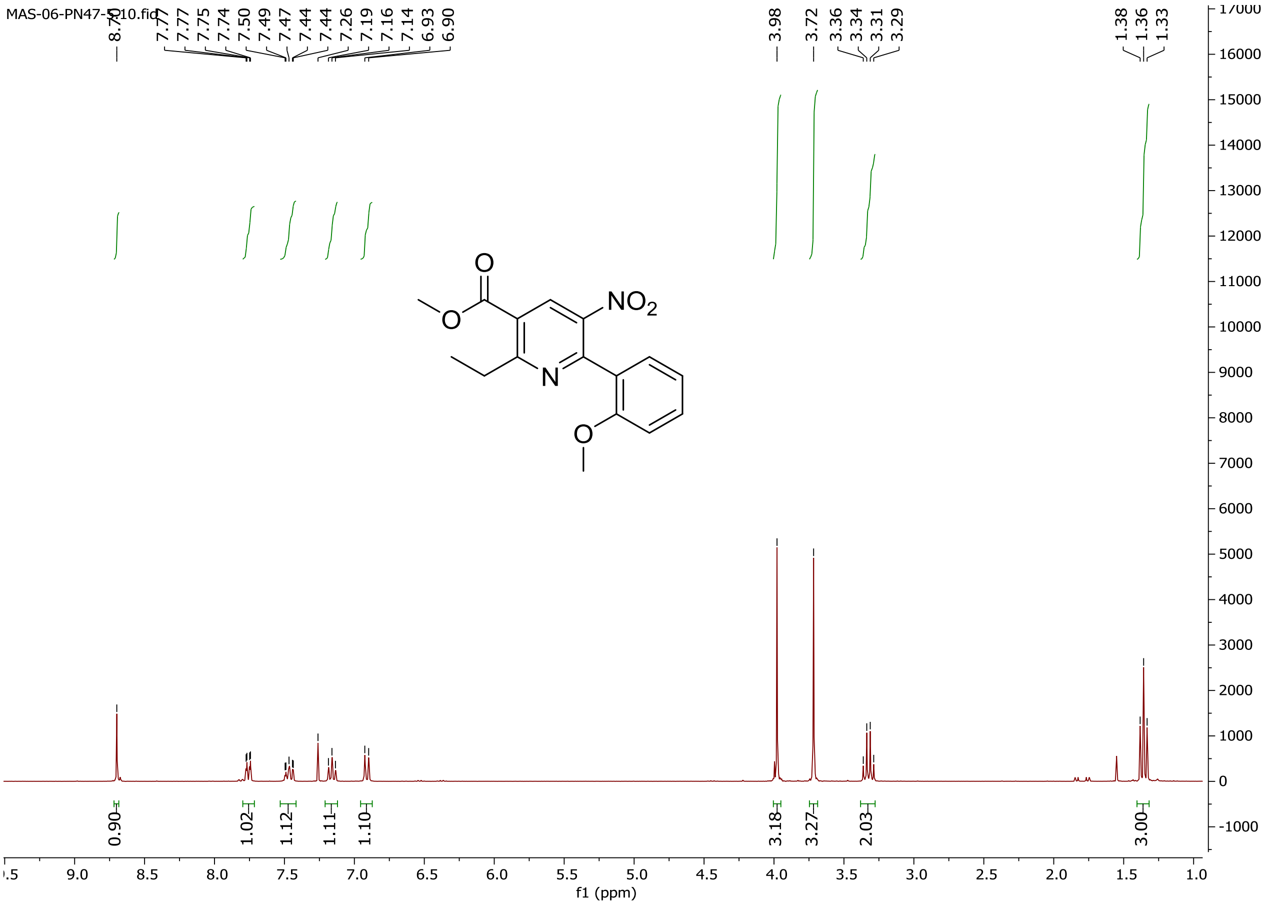

^{1}H NMR spectrum of compound **3f**

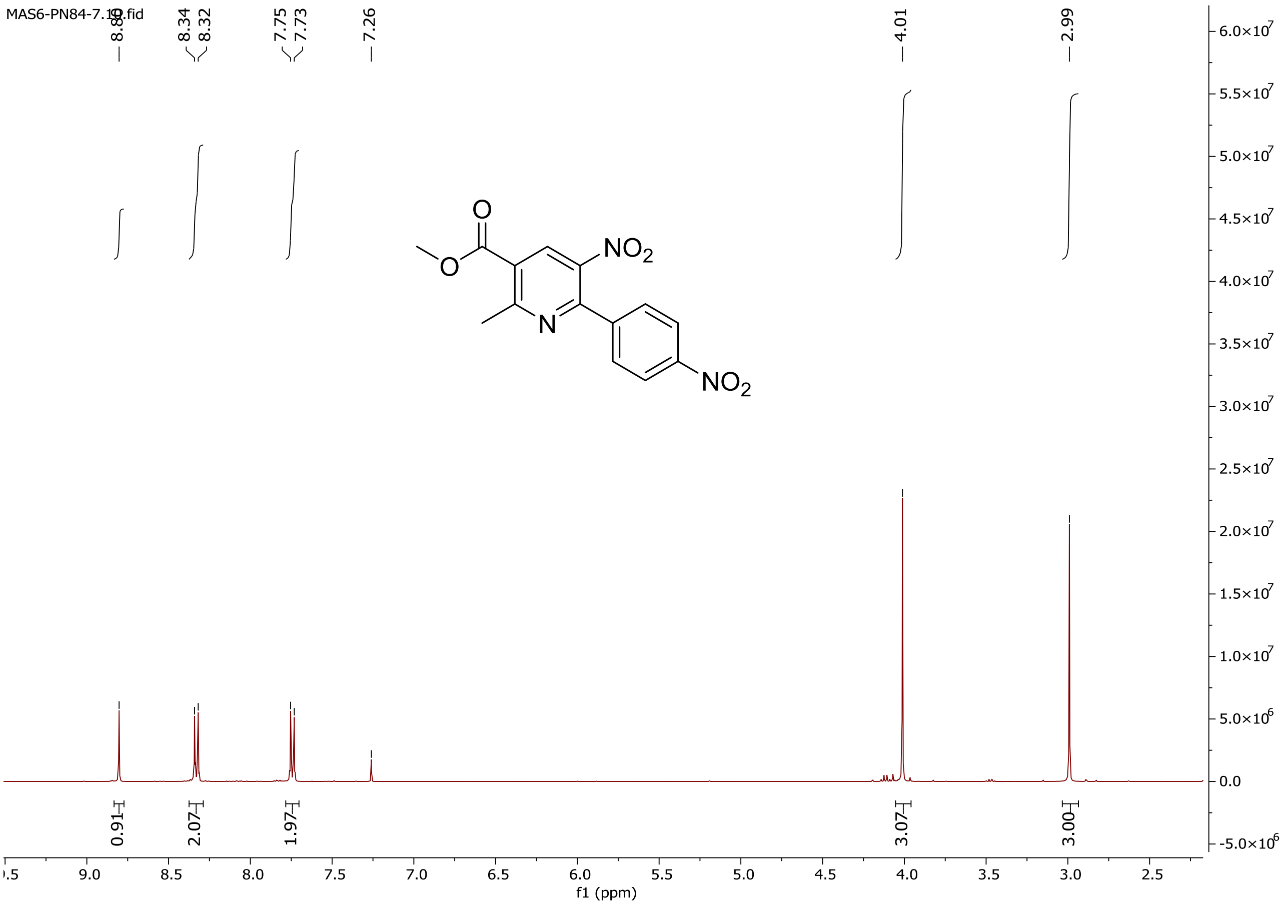

^{13}C NMR spectrum of compound **3f**

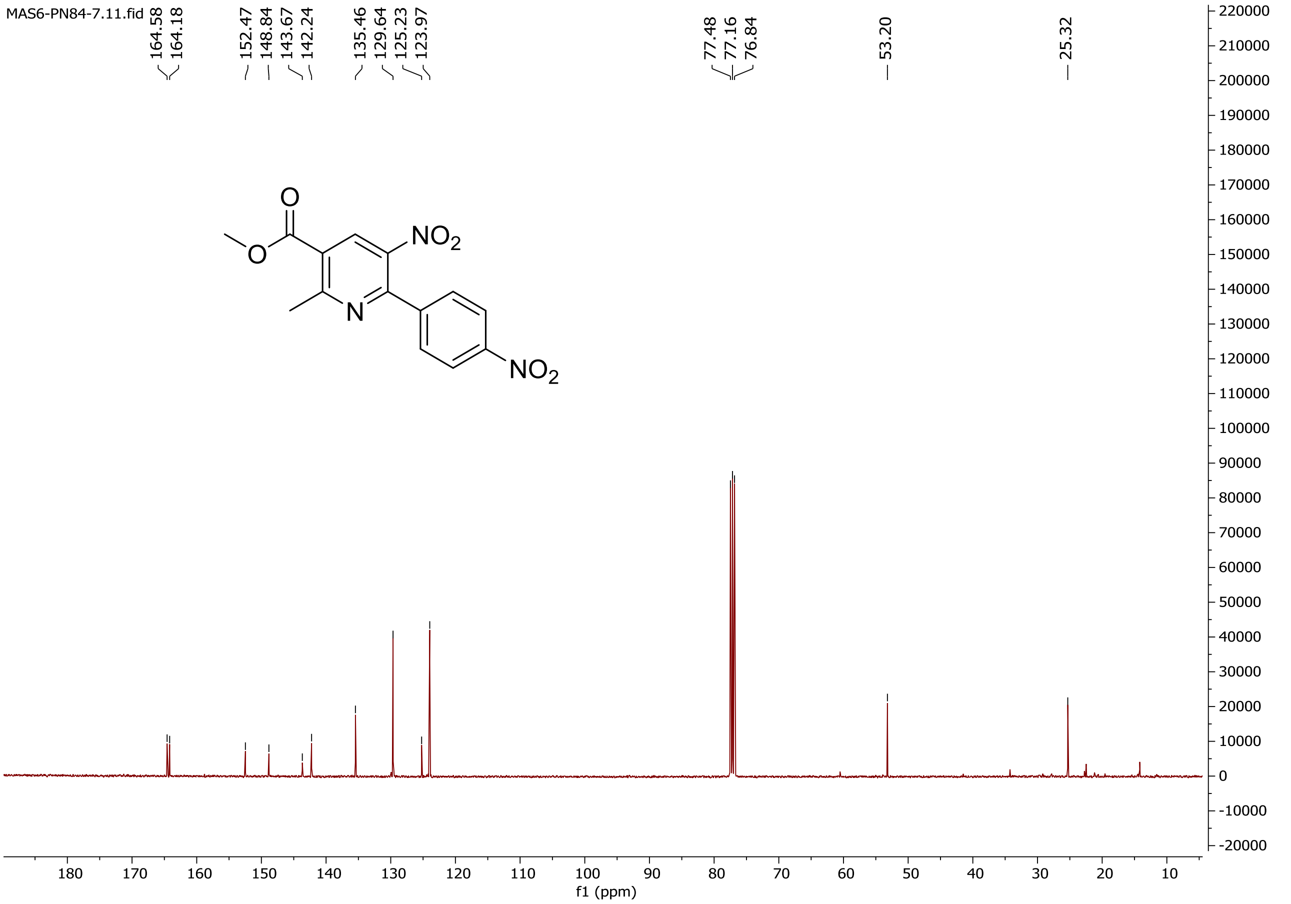

^{1}H NMR spectrum of compound **3g**

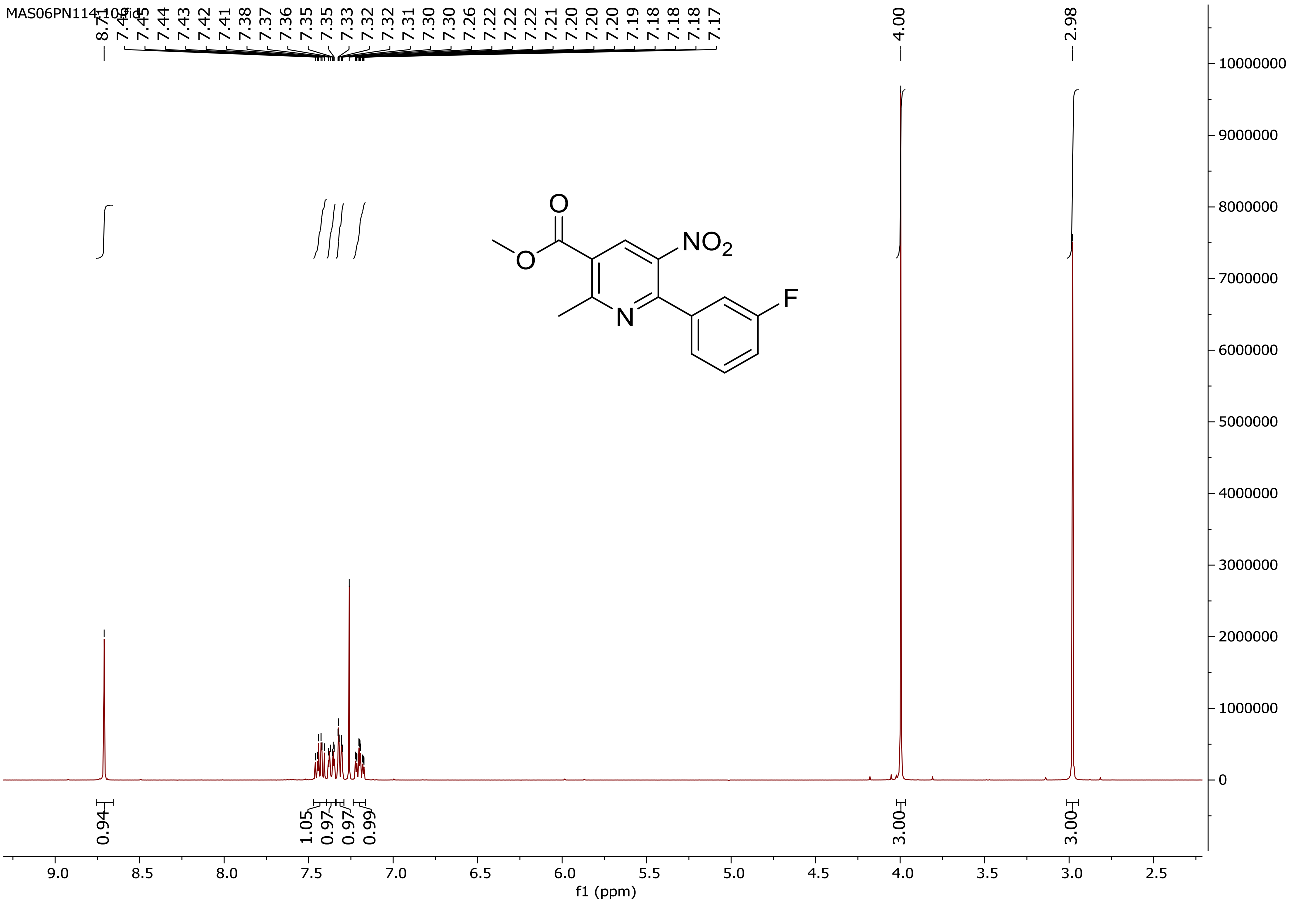

^{19}F NMR spectrum of compound **3g**

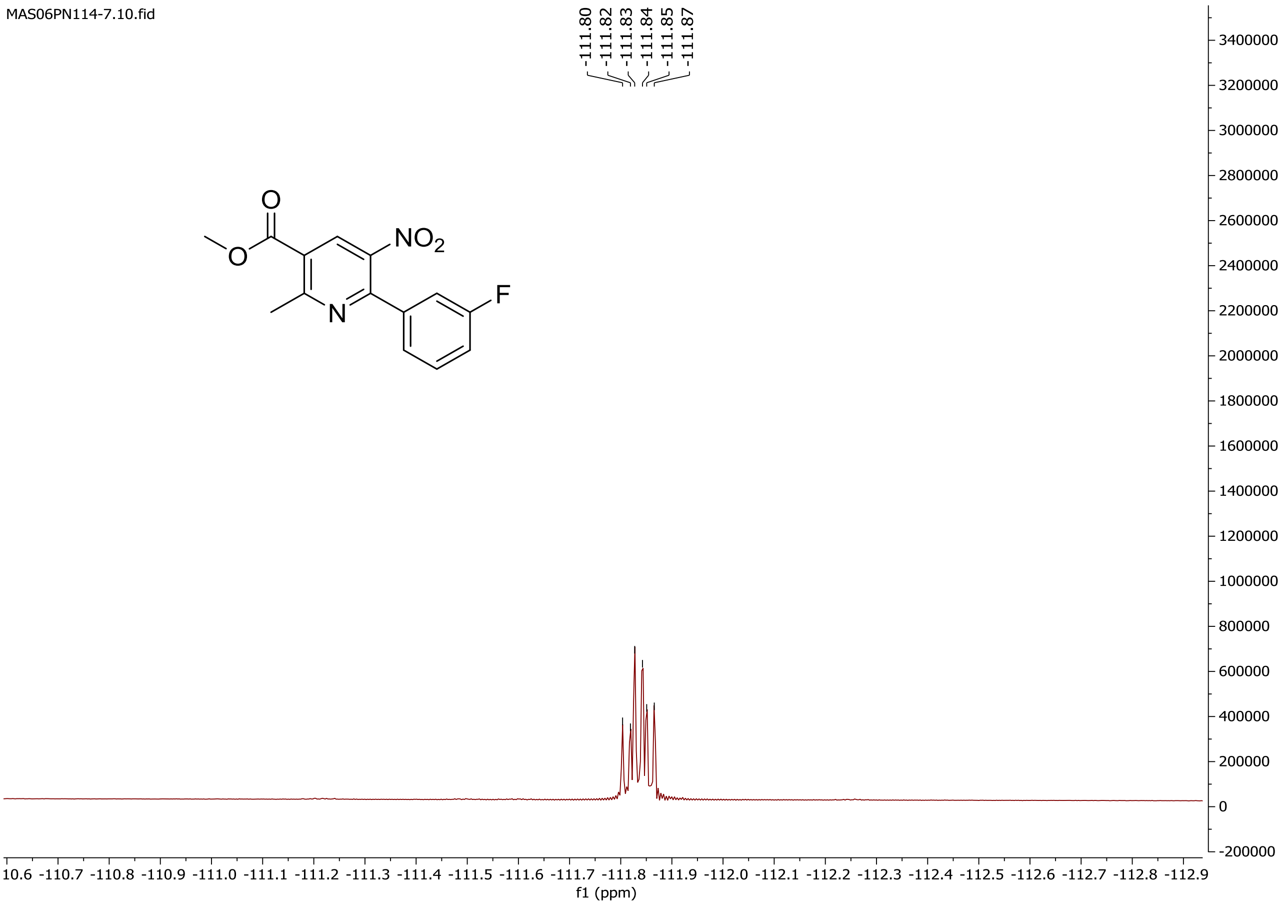

^{13}C NMR spectrum of compound **3g**

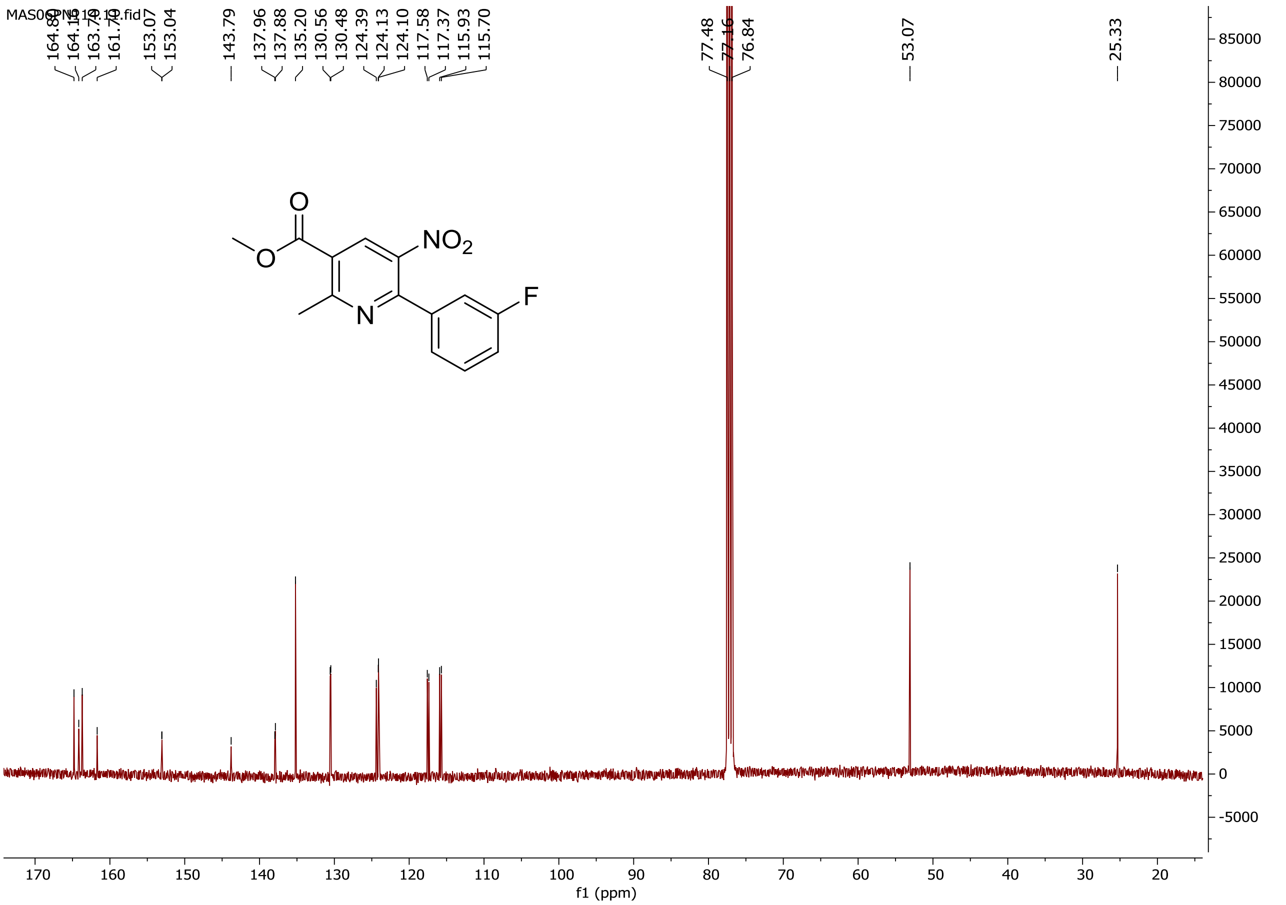

^{1}H NMR spectrum of compound **3h**

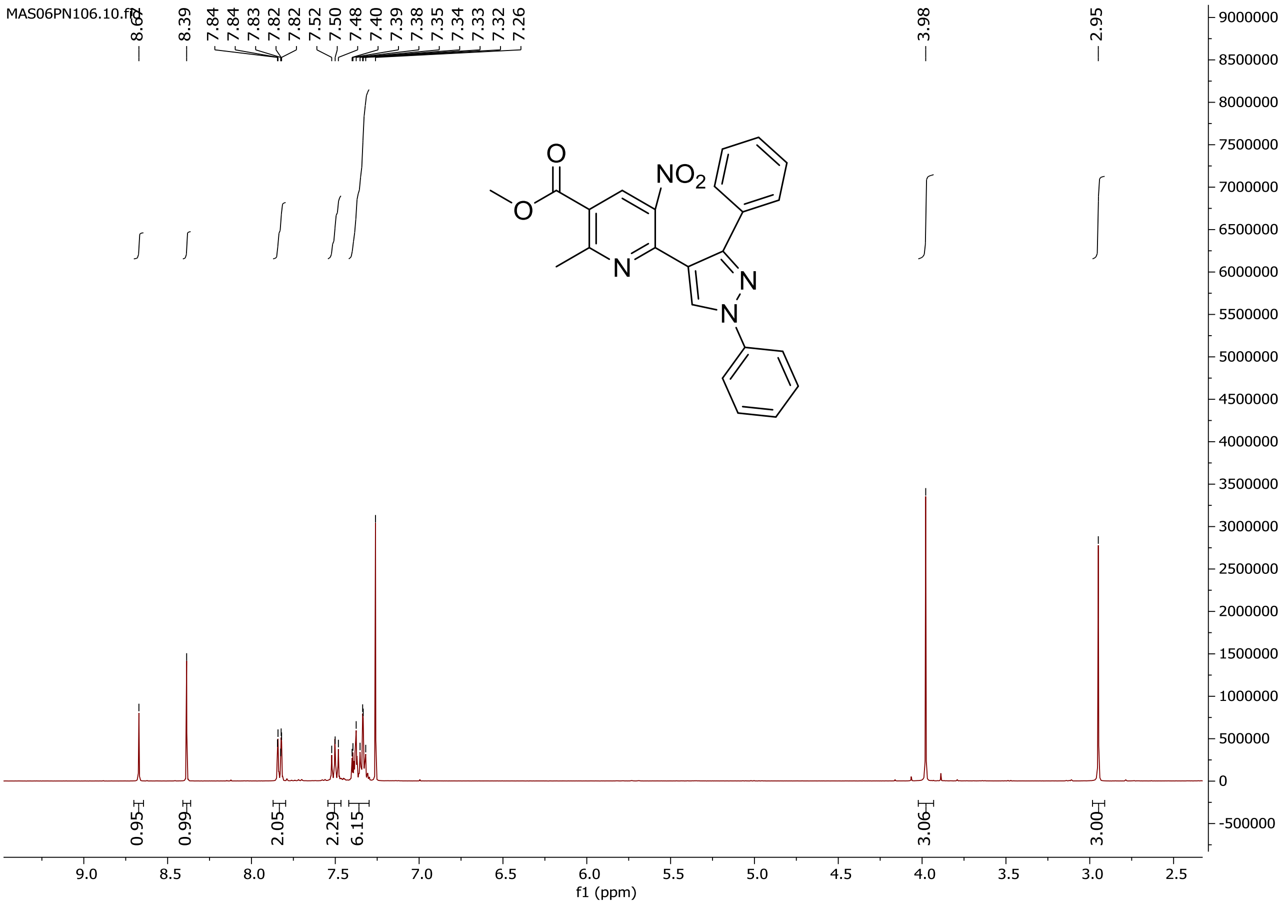

^{13}C NMR spectrum of compound **3h**

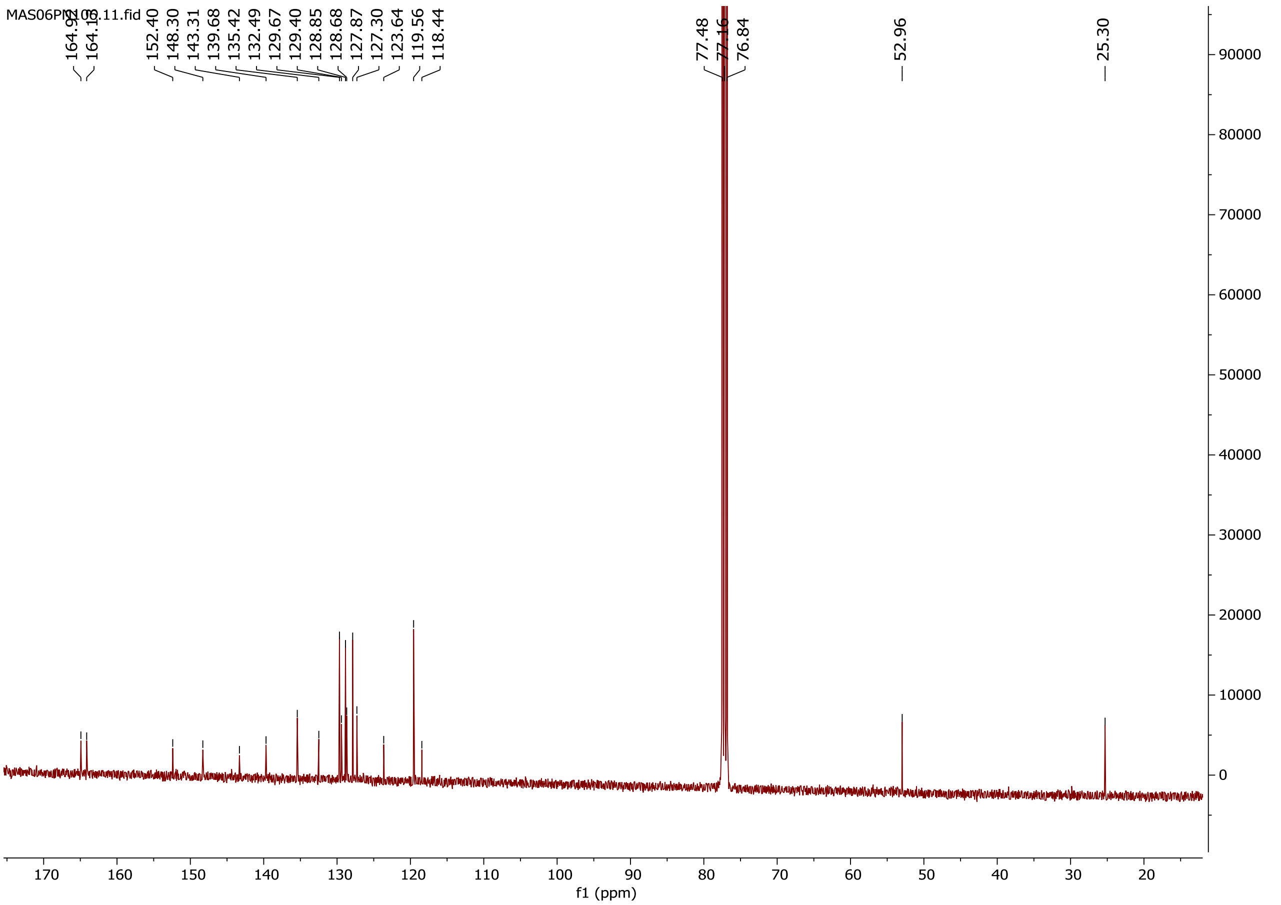

^{1}H NMR spectrum of compound **3k**

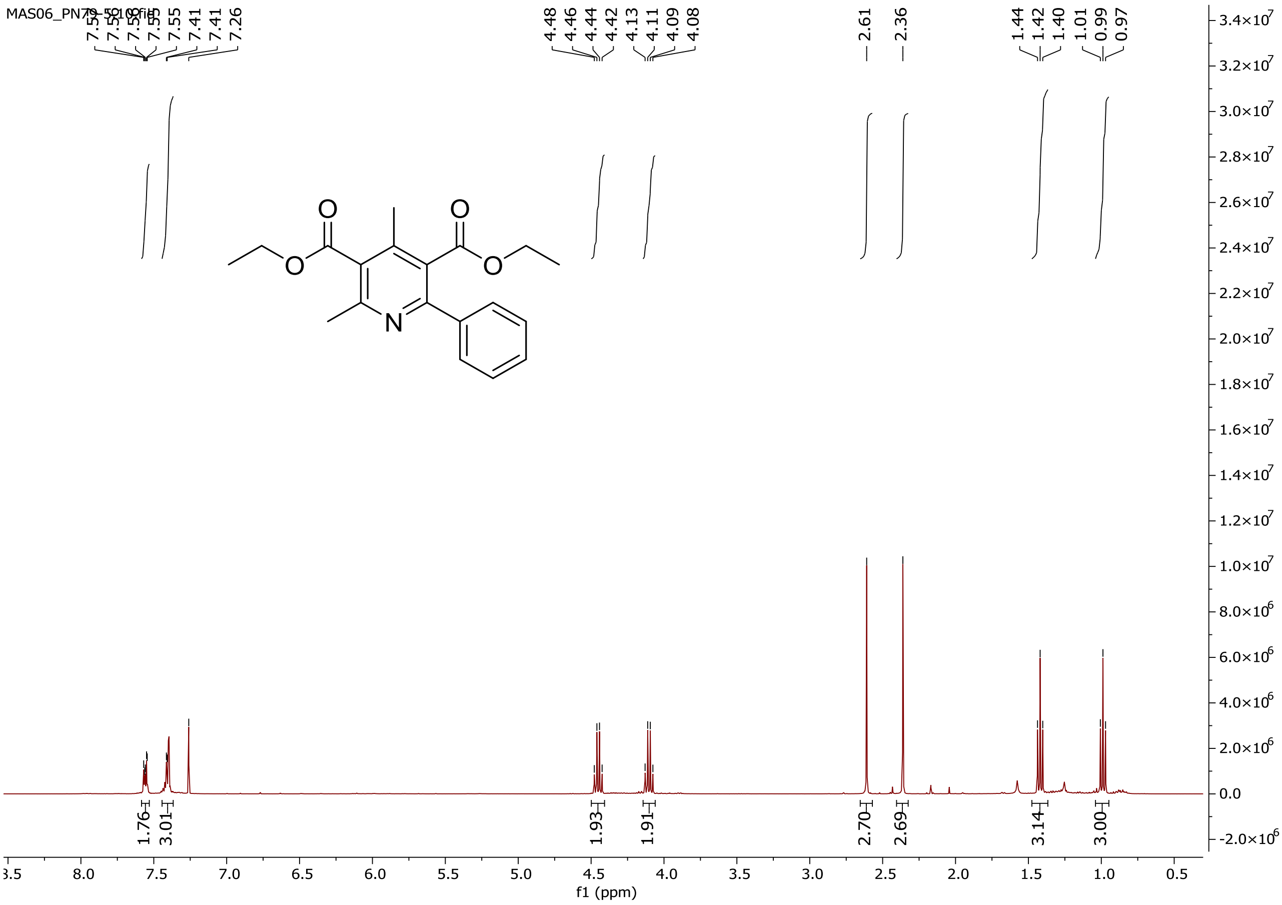

^{13}C NMR spectrum of compound **3k**

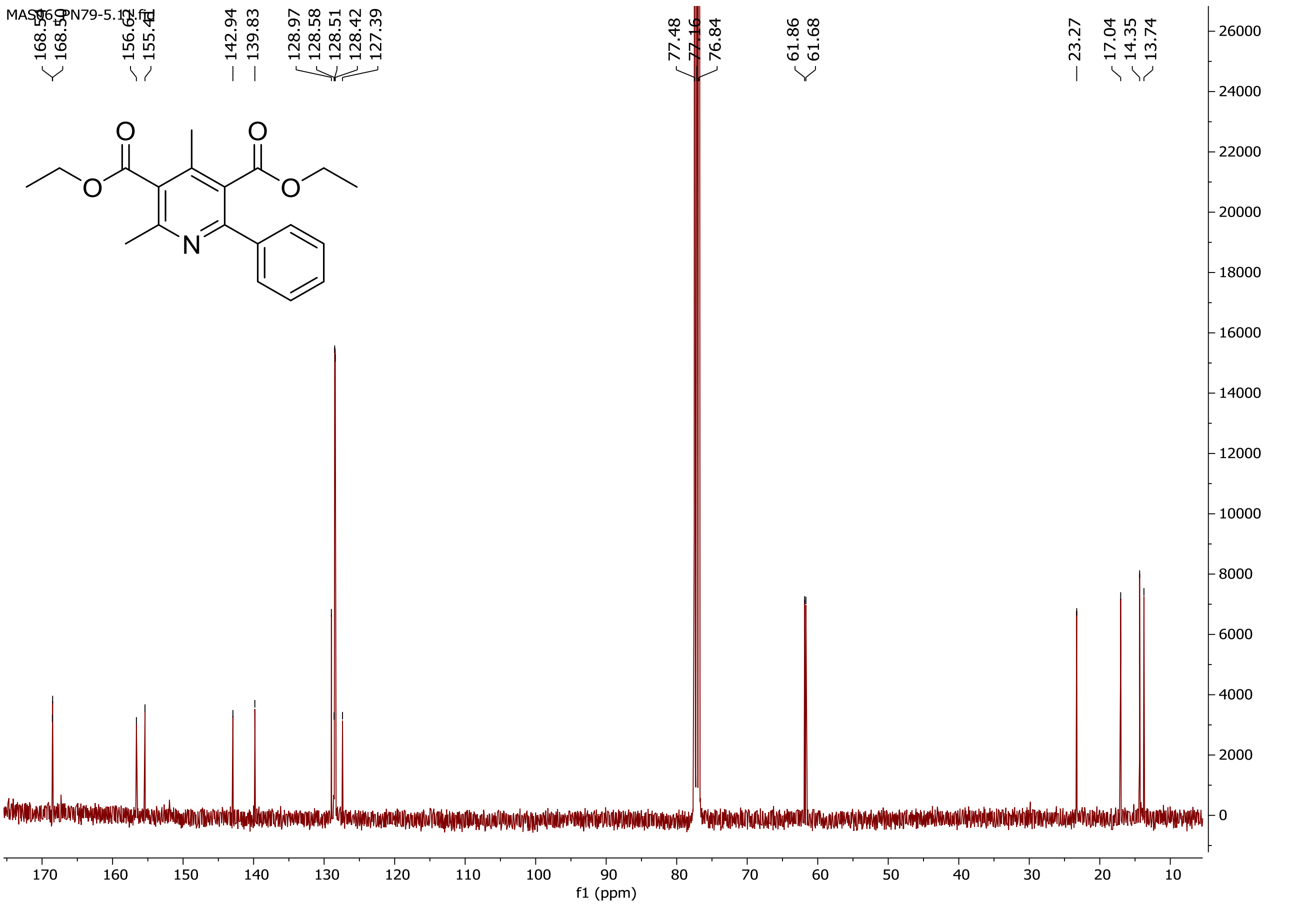

^{1}H NMR spectrum of compound **5a**

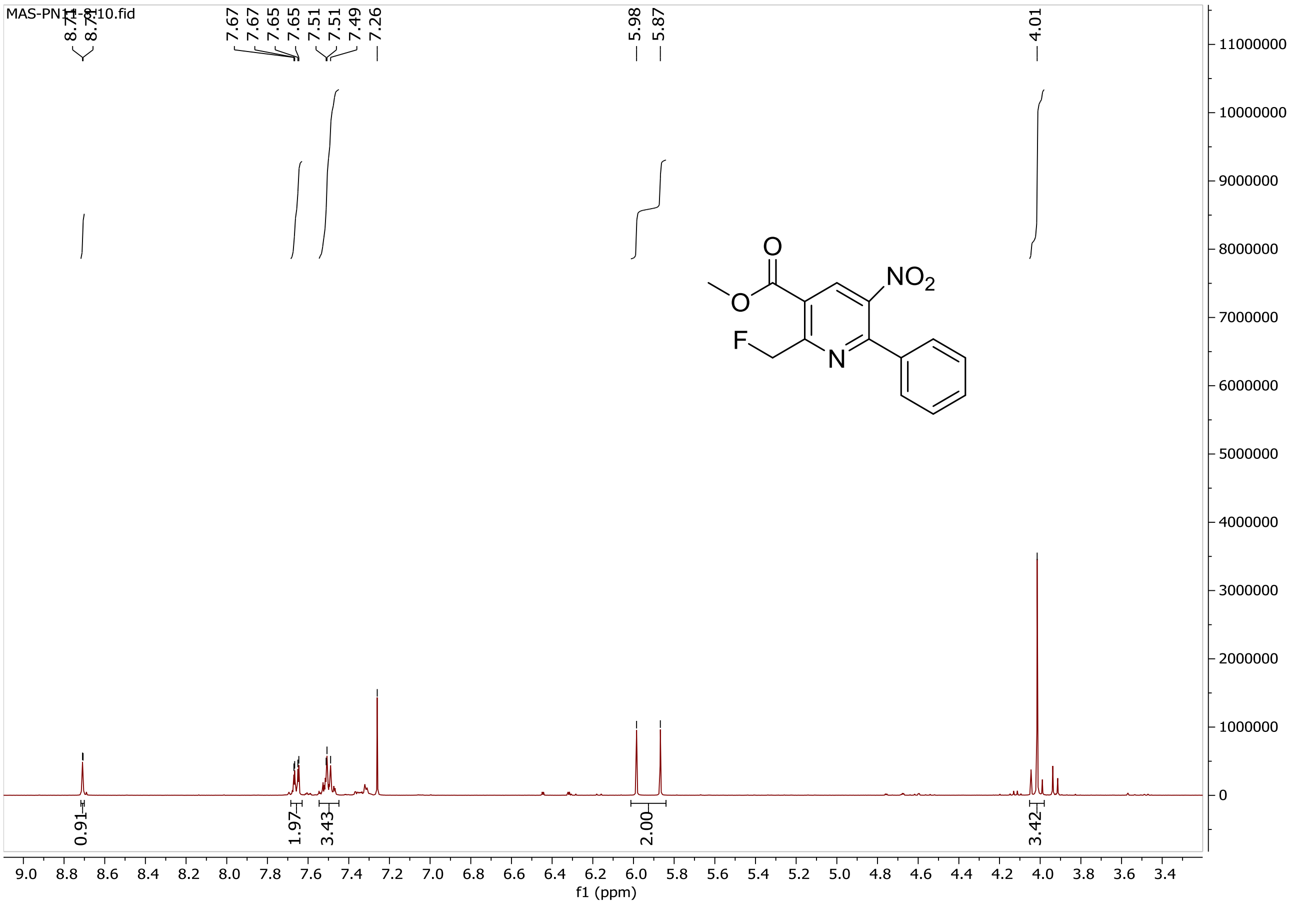

^{19}F NMR spectrum of compound **5a**

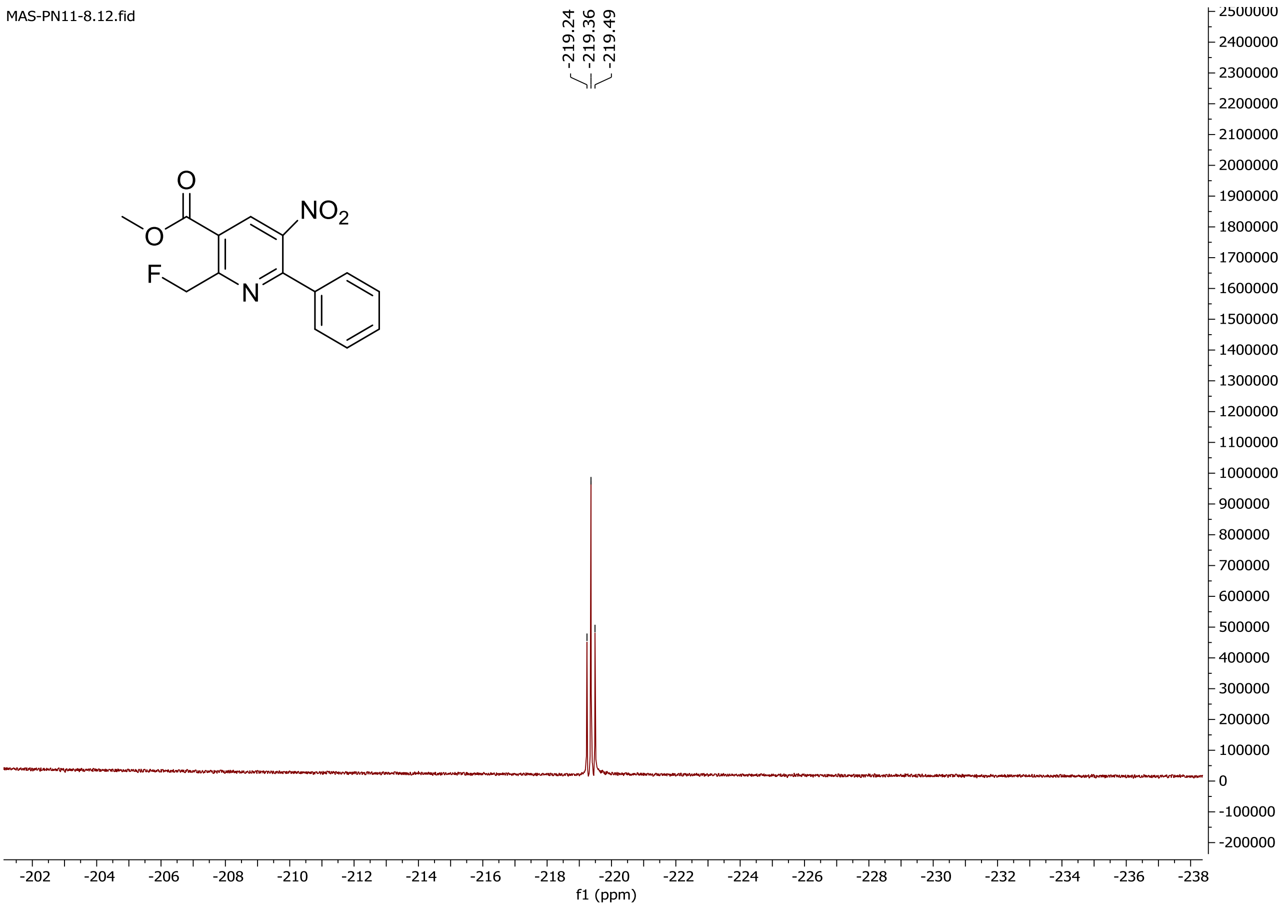

^{13}C NMR spectrum of compound **5a**

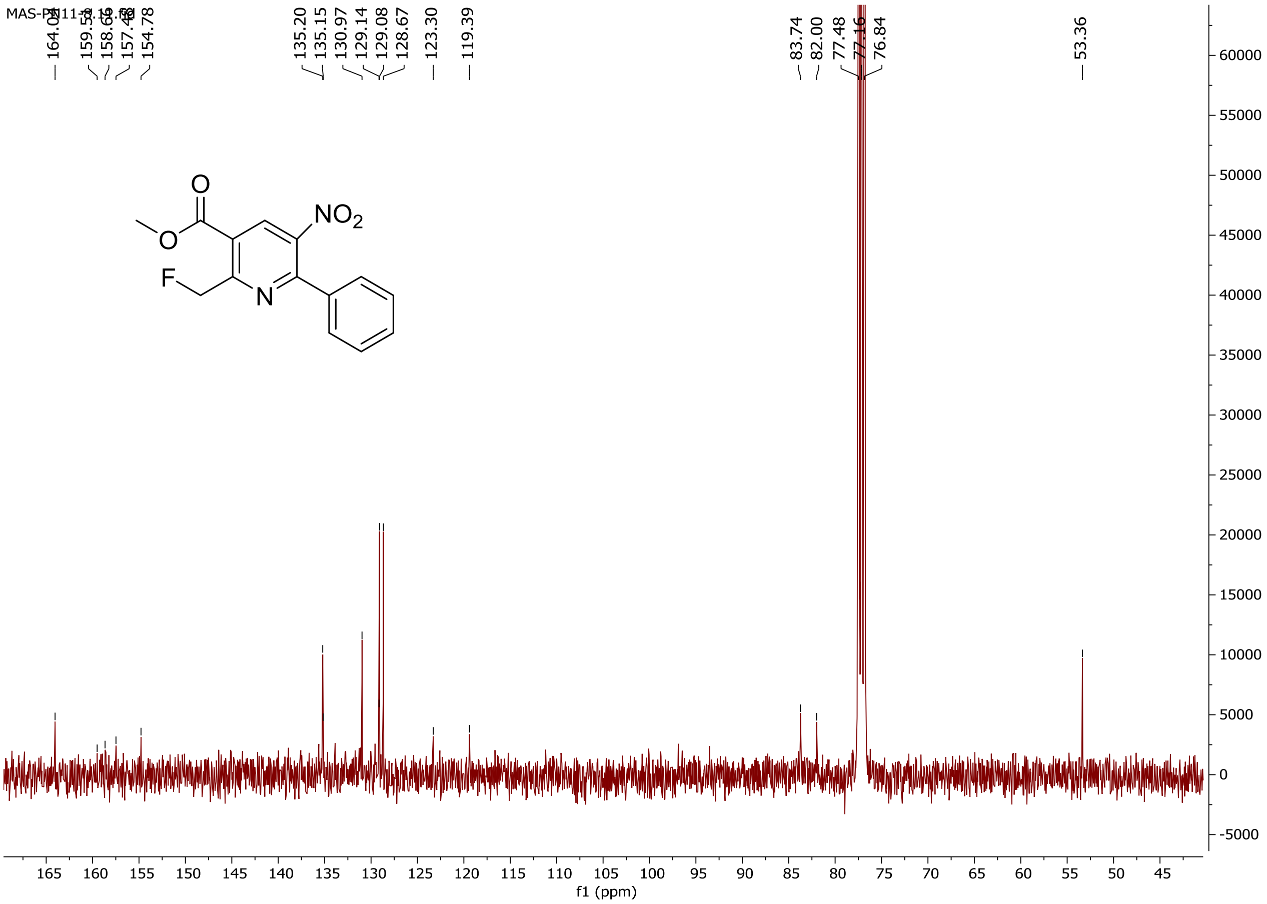

^{1}H NMR spectrum of compound **5b**

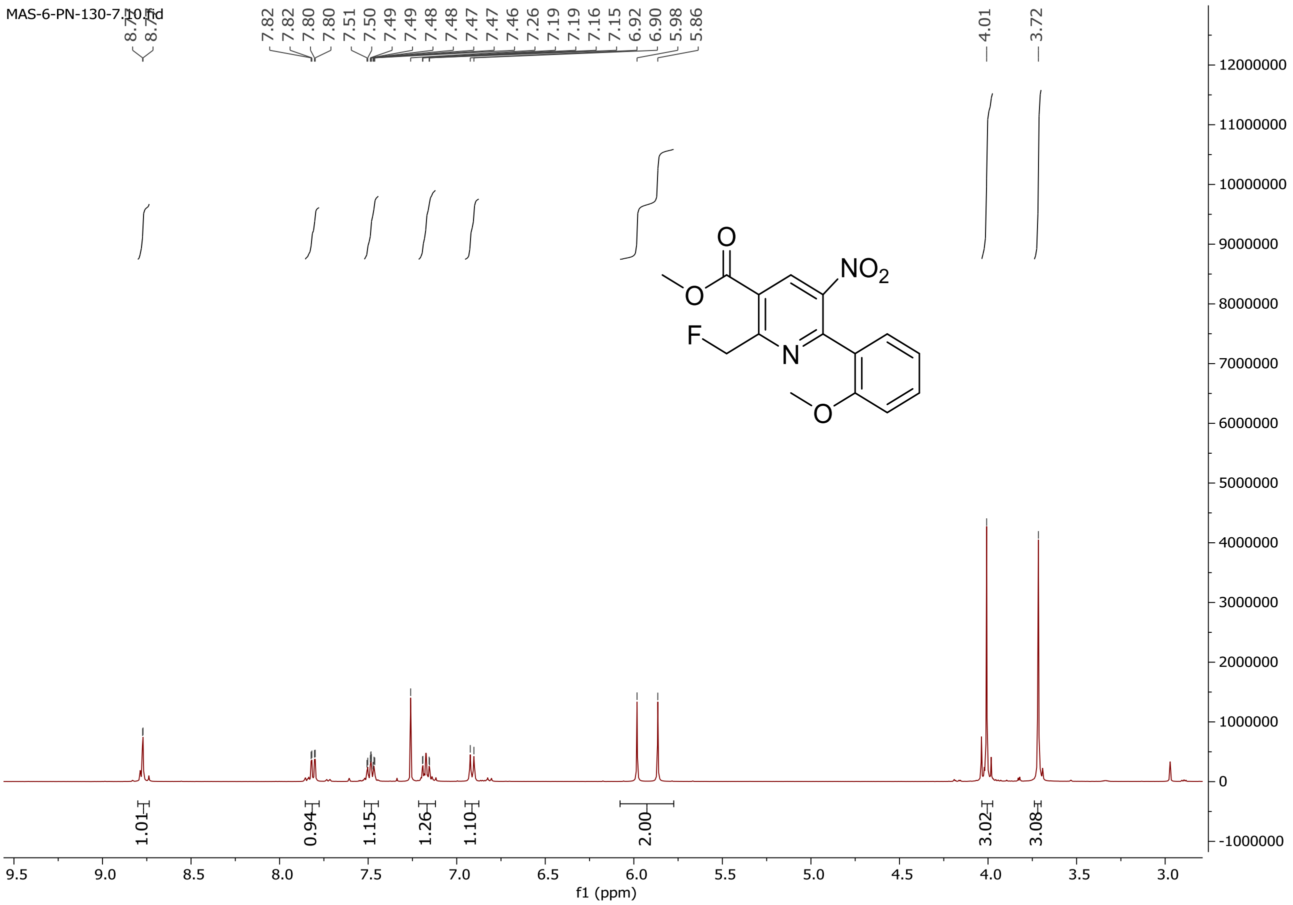

^{19}F NMR spectrum of compound **5b**

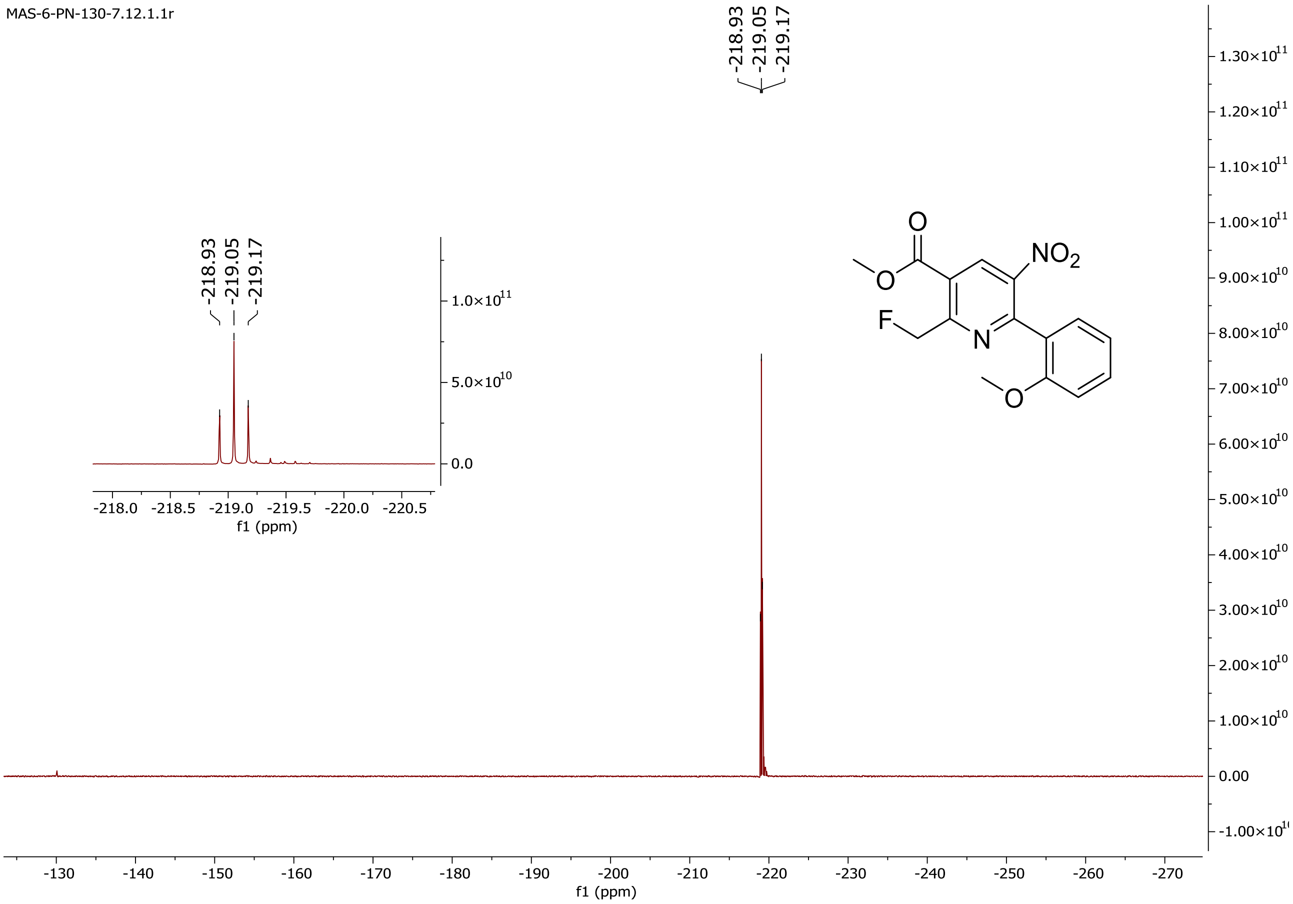

^{13}C NMR spectrum of compound **5b**

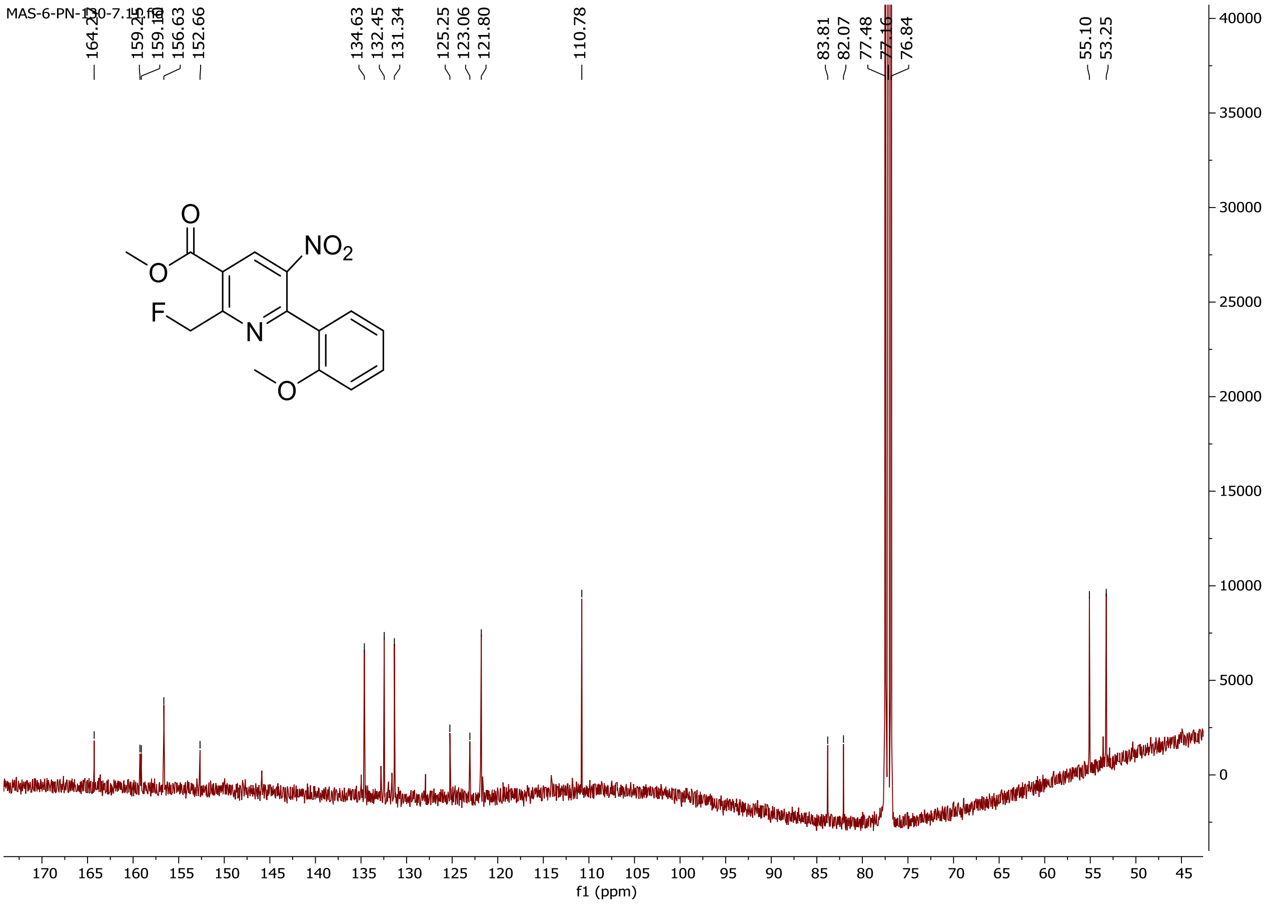

{^{1}H–^{1}H} COSY Spectrum of compound **5b**

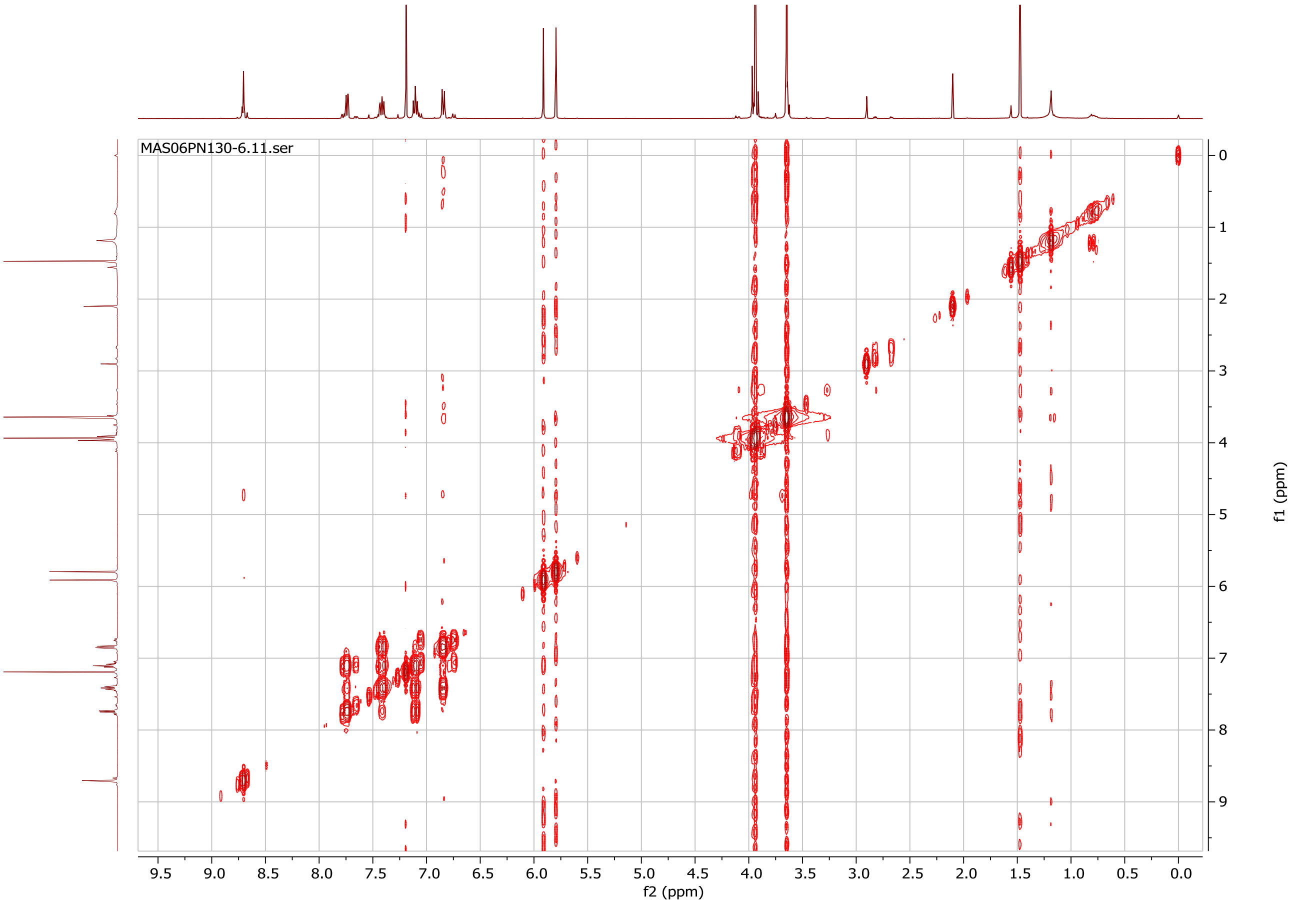

{^{13}C–^{1}H} HSQC spectrum of compound **5b**

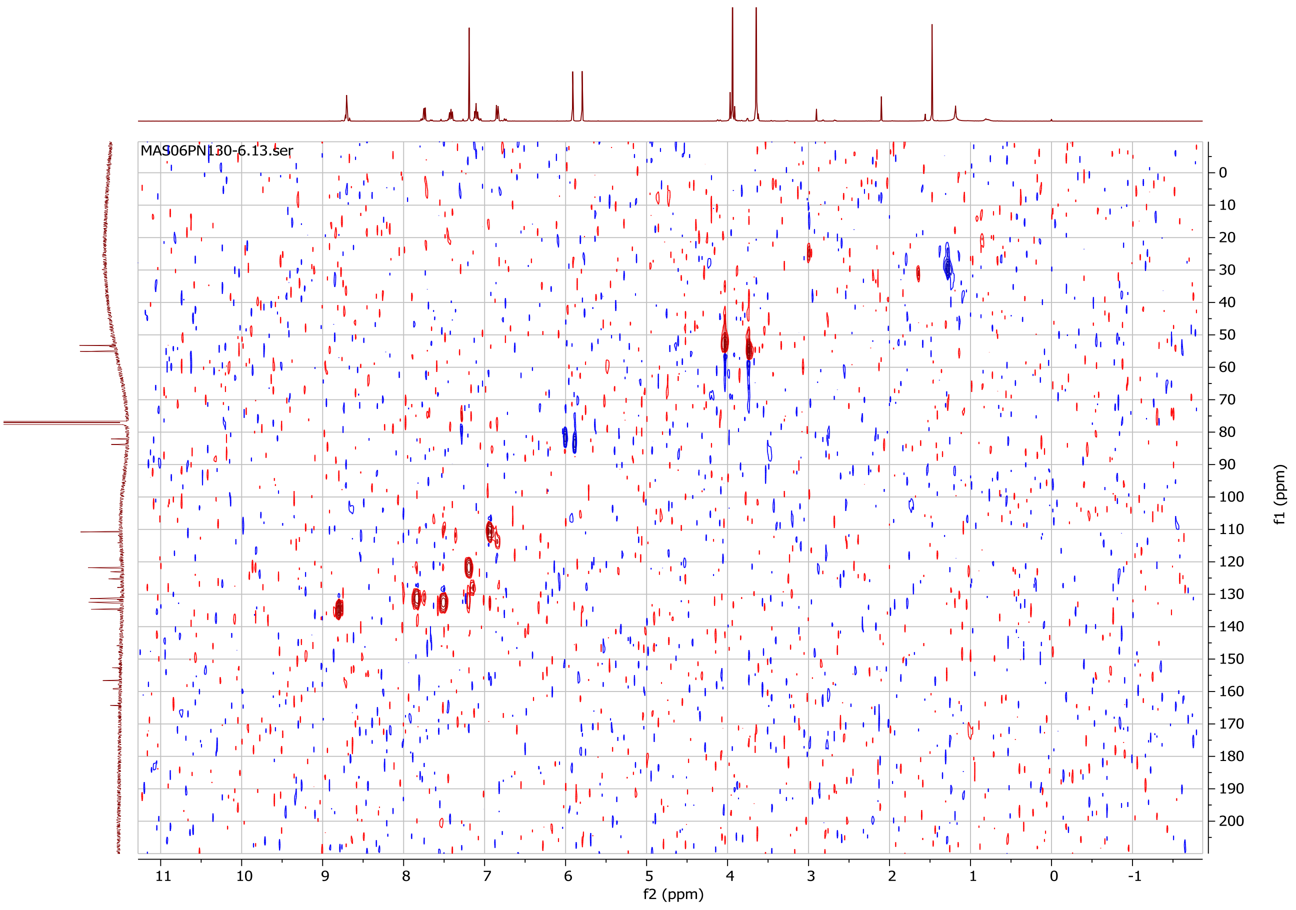

{^{13}C–^{1}H} HMBC spectrum of compound **5b**

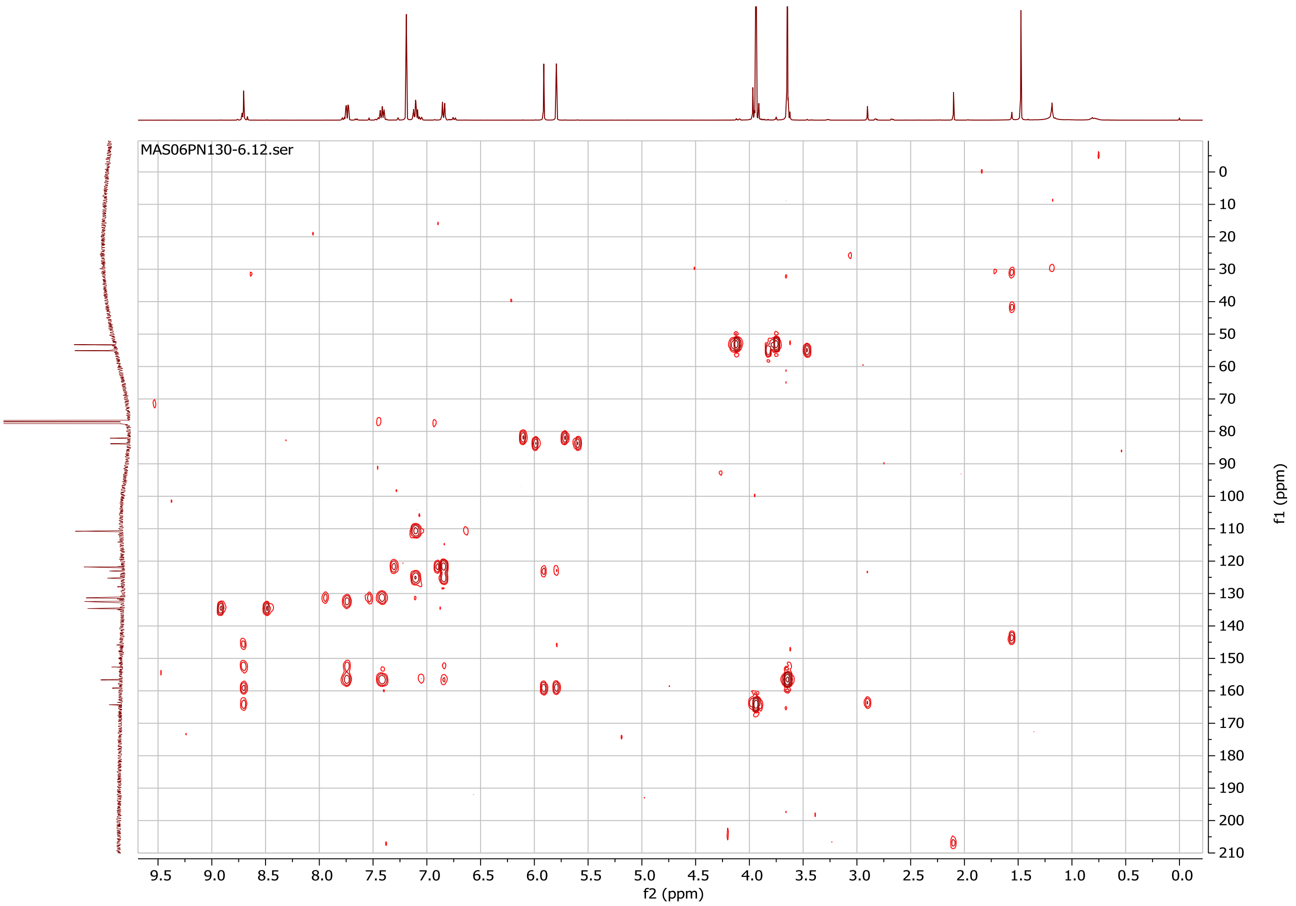

^{1}H NMR spectrum of compound **5c**

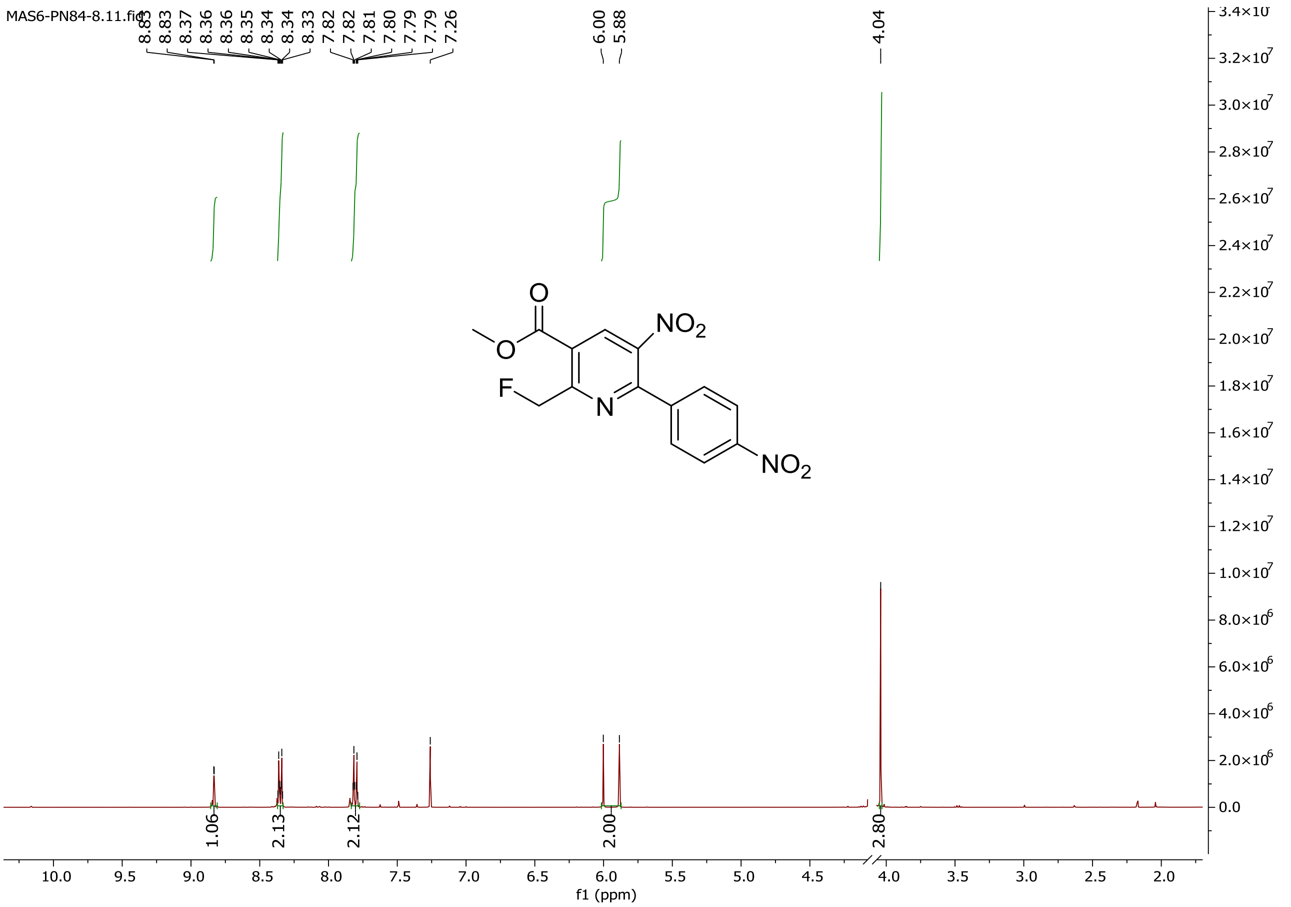

^{19}F NMR spectrum of compound **5c**

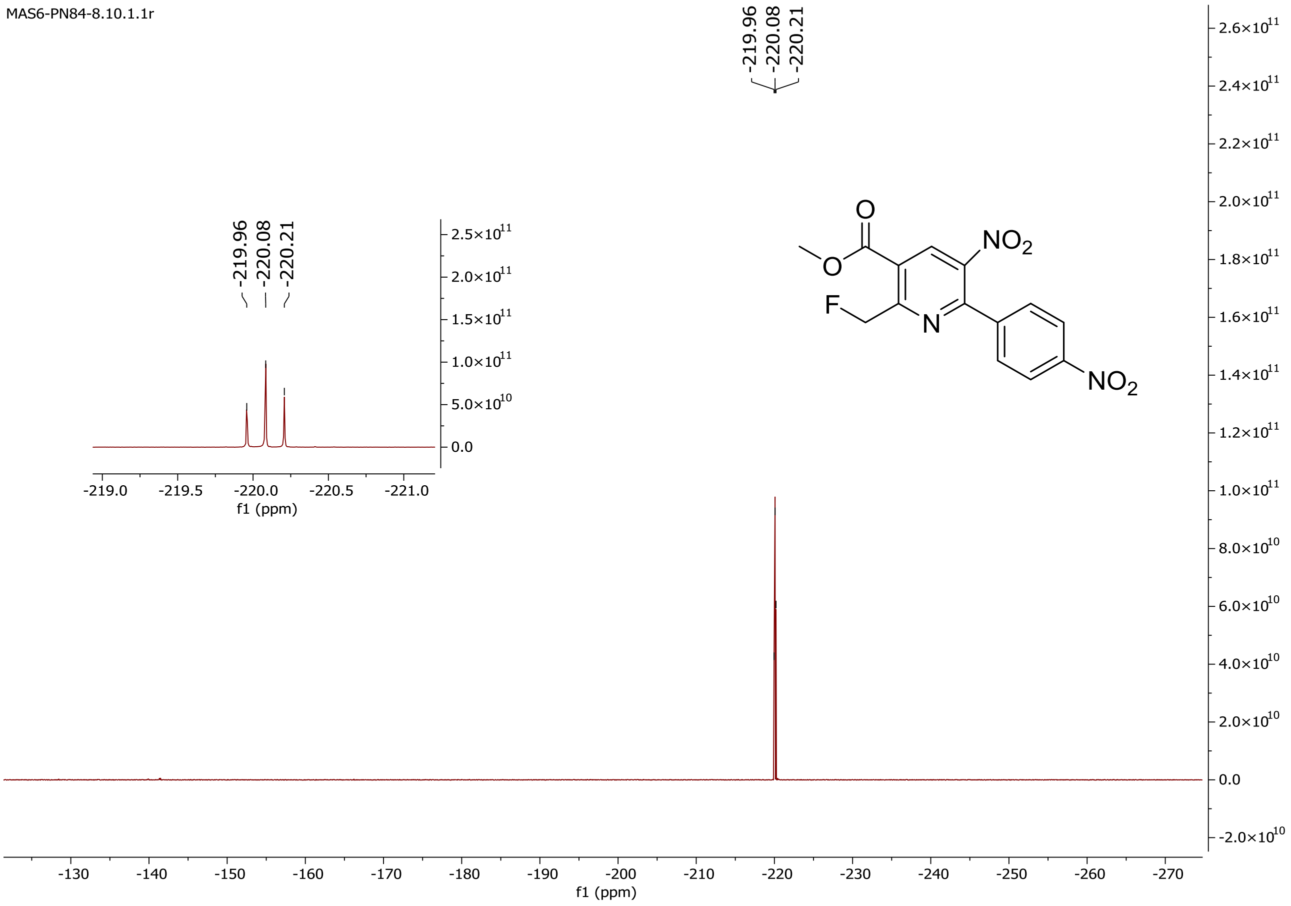

^{13}C NMR spectrum of compound **5c**

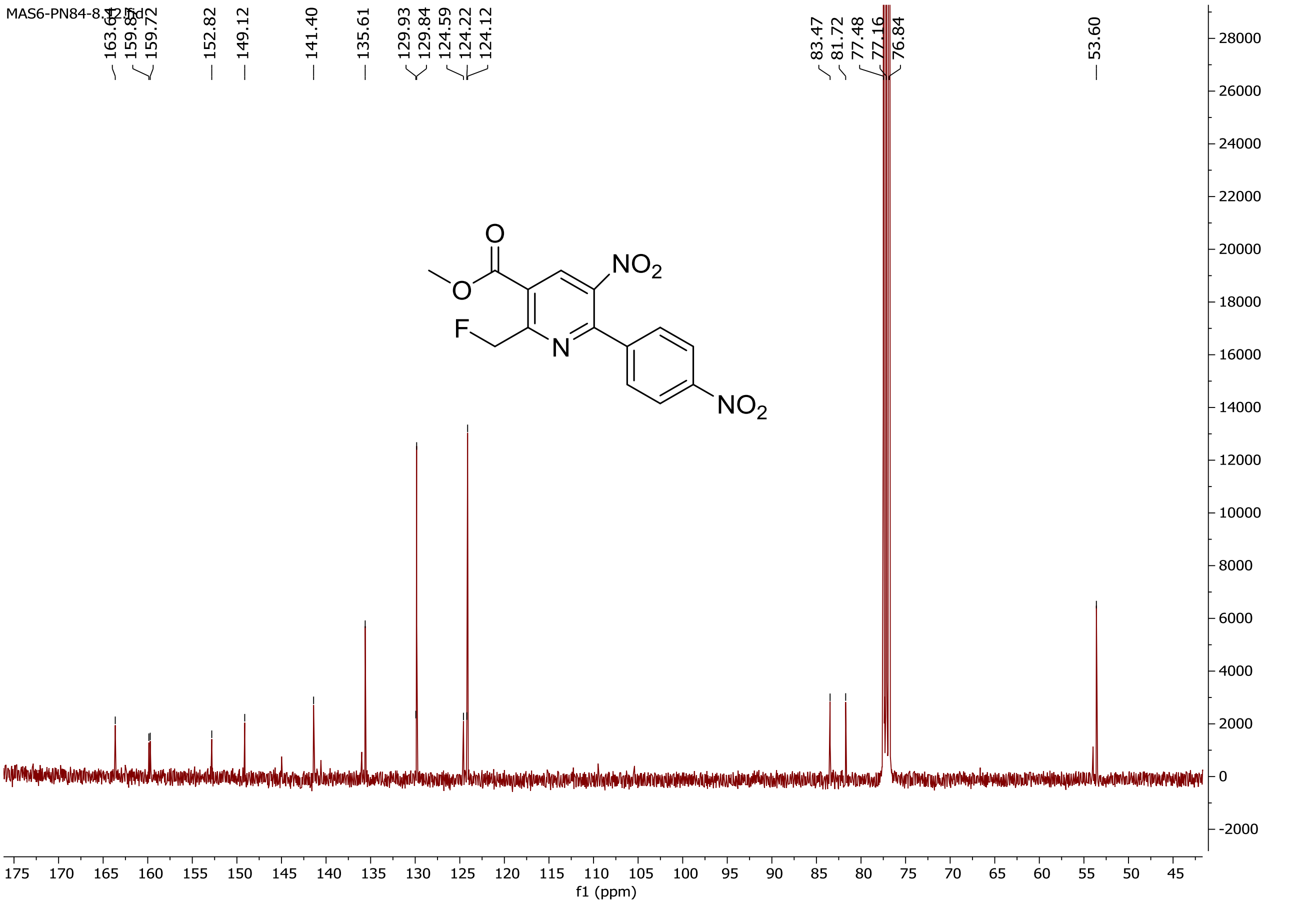

^{13}C NMR spectrum of compound **5d**

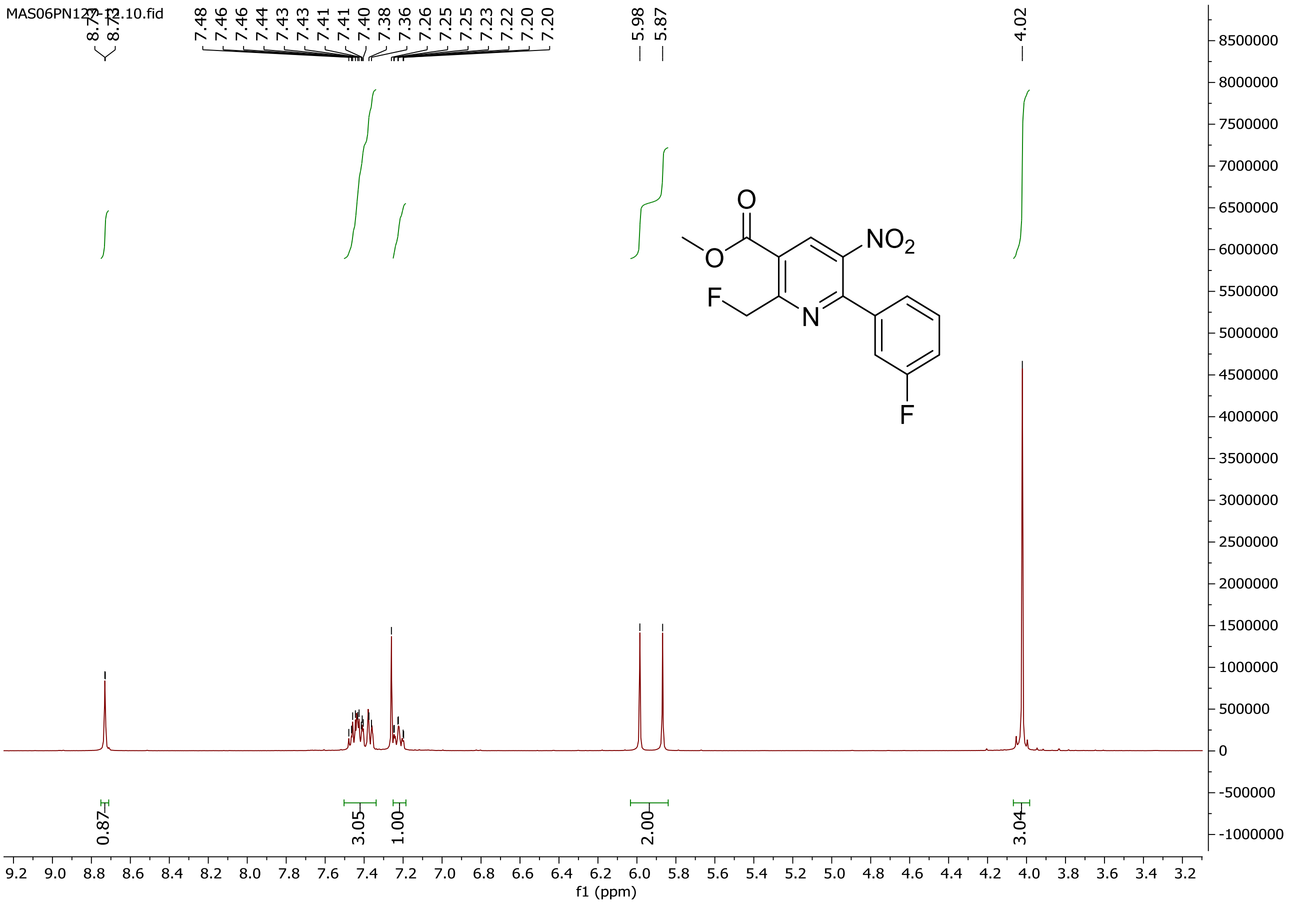

^{19}F NMR spectrum of compound **5d**

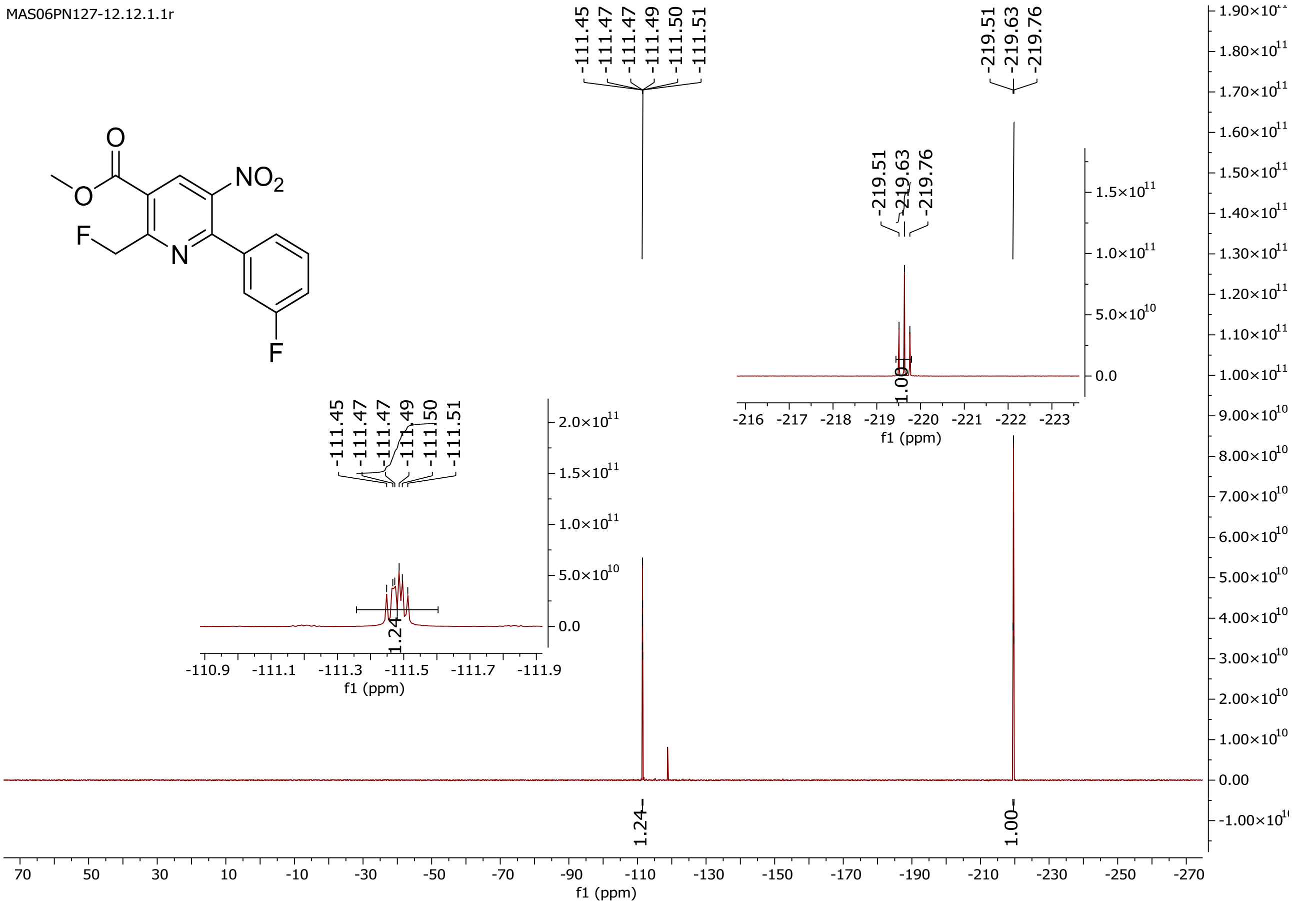

^{13}C NMR spectrum of compound **5d**

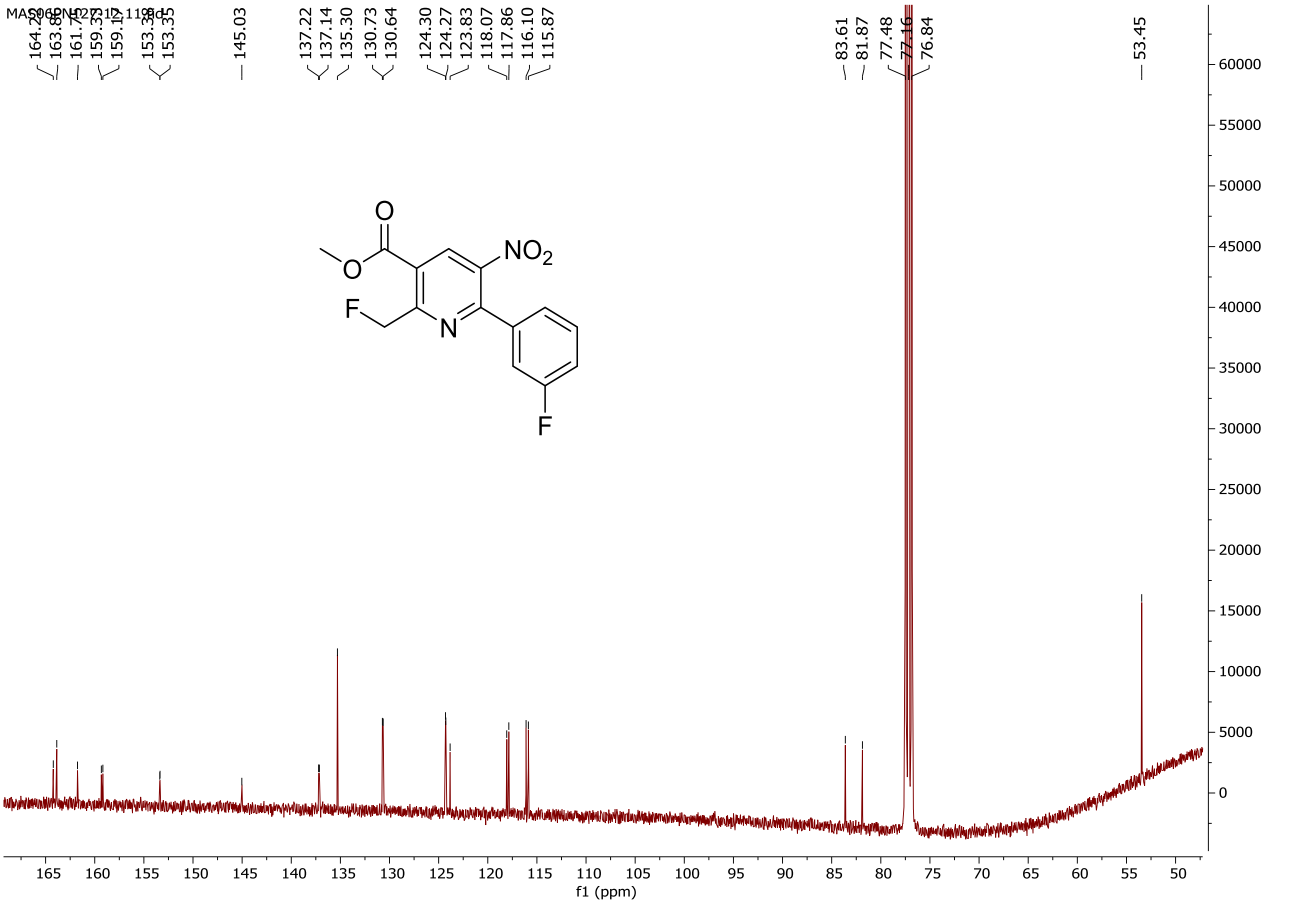

Supplement: Supplementary file 1 [file molecules-25-03143-s001.zip › Molecules_Rev1_Supporting_information.pdf]
